# Sequential Design of Activatable Photosensitizers for Ablation of Glioma Cells in Brain Cancer Tissues

**DOI:** 10.1002/anie.8453410

**Published:** 2026-09-30

**Authors:** Utsa Karmakar, Zhiming Cheng, Andrew D. M. Green, Sam Benson, Eleni Nestoros, Clarissa Lim, Justyna Cholewa‐Waclaw, Fabio de Moliner, Paula Lusarreta, Deborah Seah, Alsadeg Bilal, Neza Alfazema, Thibaud Aymoz‐Bressot, Gillian Morrison, Sonja Vermeren, Steven M. Pollard, Paul M. Brennan, Michael G. Tanner, Marc Vendrell

**Affiliations:** ^1^ Centre for Inflammation Research The University of Edinburgh Edinburgh UK; ^2^ IRR Chemistry Hub, Institute for Regeneration and Repair The University of Edinburgh Edinburgh UK; ^3^ Institute of Photonics and Quantum Sciences Heriot‐Watt University Edinburgh UK; ^4^ Center for Clinical Brain Sciences Institute for Neuroscience and Cardiovascular Research The University of Edinburgh Edinburgh UK; ^5^ Centre For Regenerative Medicine Institute for Regeneration and Repair The University of Edinburgh Edinburgh UK; ^6^ Cancer Research UK Scotland Centre The University of Edinburgh Edinburgh UK

**Keywords:** brain cancer, enzymes, PDT, phototherapeutics, probes

## Abstract

Photodynamic therapy (PDT) is a promising strategy to eradicate residual glioma cells in brain cancer patients; however, current photosensitizers (PS) show limitations on selective accumulation and controlled activation in glioma cells. In this work, we present the design, synthesis, and characterization of activatable PSs for selective PDT of glioma cells in mouse and human brain tissues. First, we designed and characterized a broad collection of photosensitive metabolite conjugates, from which we prepared **Gal‐SeNBD‐oleamide** as a β‐galactosidase‐responsive PS with efficient uptake in patient‐derived glioma cells and selectivity over immune cells. Furthermore, we optimized a whole‐tissue illumination strategy using **Gal‐SeNBD‐oleamide** in mouse brain slices, which led to efficient ablation of cancer cells with minimal damage to surrounding regions. Finally, the proof‐of‐concept application of **Gal‐SeNBD‐oleamide** directly in tissues from brain cancer patients supports its translational potential for PDT of glioblastoma.

## Graphical abstract

**Gal‐SeNBD‐oleamide** is an activatable photosensitizer (PS) with efficient uptake and selective, β‐galactosidase‐triggered activation in cancer cells, enabling efficient photodynamic ablation of glioma cells directly in brain tissue slices from mouse preclinical models and human glioblastoma patients.

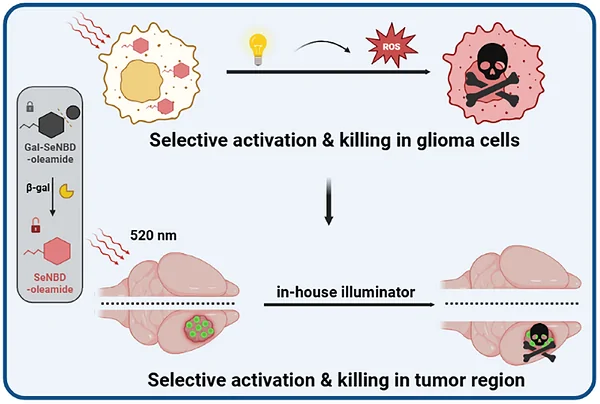

## 1 Introduction

Glioblastoma is the most aggressive form of all gliomas, accounting for nearly 70% of adult brain tumors [1]. Patients with glioblastoma have overall survival rates around 15 months from the time of diagnosis, and less than 10% of patients survive 5 years or longer [2, 3, 4, 5]. Current practice for glioblastoma treatment involves gross maximal resection of tumors followed by chemotherapy with temozolomide and radiotherapy [3, 4, 5]; however, this approach is rarely effective at eliminating glioma cells, and tumors can re‐grow at eloquent regions of the brain. Emerging modalities, including tumor‐treating fields and anti‐angiogenic agents such as bevacizumab, have provided only modest improvements in outcome [6]. As a result, recurrence within close distance to primary tumors occurs in many brain cancer patients [7, 8, 9]. Therefore, there is a need to develop safe approaches for effective elimination of glioma cells in brain tissues with reduced cross‐reactivity in surrounding immune cells.

Photodynamic therapy (PDT) is an effective therapeutic modality to ablate cancer cells after the surgical removal of tumors [10, 11, 12, 13, 14, 15]. PDT utilizes nontoxic light to enable the production of reactive oxygen species (ROS) by exogenous photosensitizers (PS) [16]. To date, 5‐aminolevulinic acid (5‐ALA) is the most common clinically‐used PS for PDT in patients with brain cancer [17]; however, the photodynamic activity of 5‐ALA cannot be modulated by tumor‐specific biomarkers and its therapeutic efficacy is hindered by tumor heterogeneity [18].

Over the past decade, our group and others have developed activatable PS whose optical properties are controlled in a biomarker‐dependent manner to enable cell‐selective activation [19, 20, 21, 22, 23, 24, 25, 26]. Activatable probes can respond to various physiological triggers (e.g., enzymatic action [27, 28, 29], pH [30, 31], redox changes [32], hypoxia [33, 34]); however, it is currently difficult to optimize their PDT illumination regimes because these biomarkers can vary across brain tissues. Some PDT strategies measure the production of singlet oxygen (e.g., via near‐infrared luminescence emission) [35, 36, 37] or indirect readouts associated with singlet oxygen generation (e.g., PS decay) [38, 39]. These readouts are difficult to measure in brain tissues because of the short lifetimes of singlet oxygen in cells and potential photobleaching. To overcome this shortcoming, we envisaged that real‐time monitoring of PS activation in brain tissues would lead to optimal PDT for ablation of glioma cells. To the best of our knowledge, there are no examples where the release of activatable PS has been directly monitored in human brain tissues.

In this work, we adopted a sequential design strategy comprising two stages: first, we synthesized and evaluated a collection of PS‐metabolite conjugates to identify new photosensitive agents with rapid uptake and high phototoxicity in patient‐derived glioma cells; second, we enhanced their selectivity by inclusion of β‐galactosidase‐responsive moieties to generate Gal‐SeNBD‐oleamide with confined PS activation to glioma cells. Furthermore, we built and employed a new on‐the‐fly illumination and spectroscopy device and used it to demonstrate the application of Gal‐SeNBD‐oleamide for direct PDT‐mediated ablation in mouse and human brain tumor tissues.

## 2 Results and Discussion

### 2.1 Rational Design and Synthesis of a Chemical Library of Photosensitive Metabolites

We started the design of targeted PS building on the benzoselenadiazole scaffold. Benzoselenadiazoles are modular and low molecular weight structures with favorable properties as PS, namely good singlet oxygen generation quantum yields, cell permeability, and chemical tunability [40, 41, 42, 43]. Given that the reduced size and neutral character of benzodiazoles can be utilized to generate analogues of small‐molecule metabolites [42], we designed a library whereby a functionalized benzoselenadiazole scaffold was coupled to multiple biomolecules associated with glioma metabolism (Figure 1). Among others, the metabolites included glucose due to the Warburg effect [44], amino acids that drive one‐carbon metabolism to support essential processes in cancer cells (e.g., alanine, serine, glycine) [45, 46], neurotransmitters (e.g., acetylcholine) whose transporters are linked to aggressive tumors [47], and fatty acids, which can be taken up by glioma cells for fatty acid oxidation as an alternative energy source [48, 49].

**FIGURE 1:**
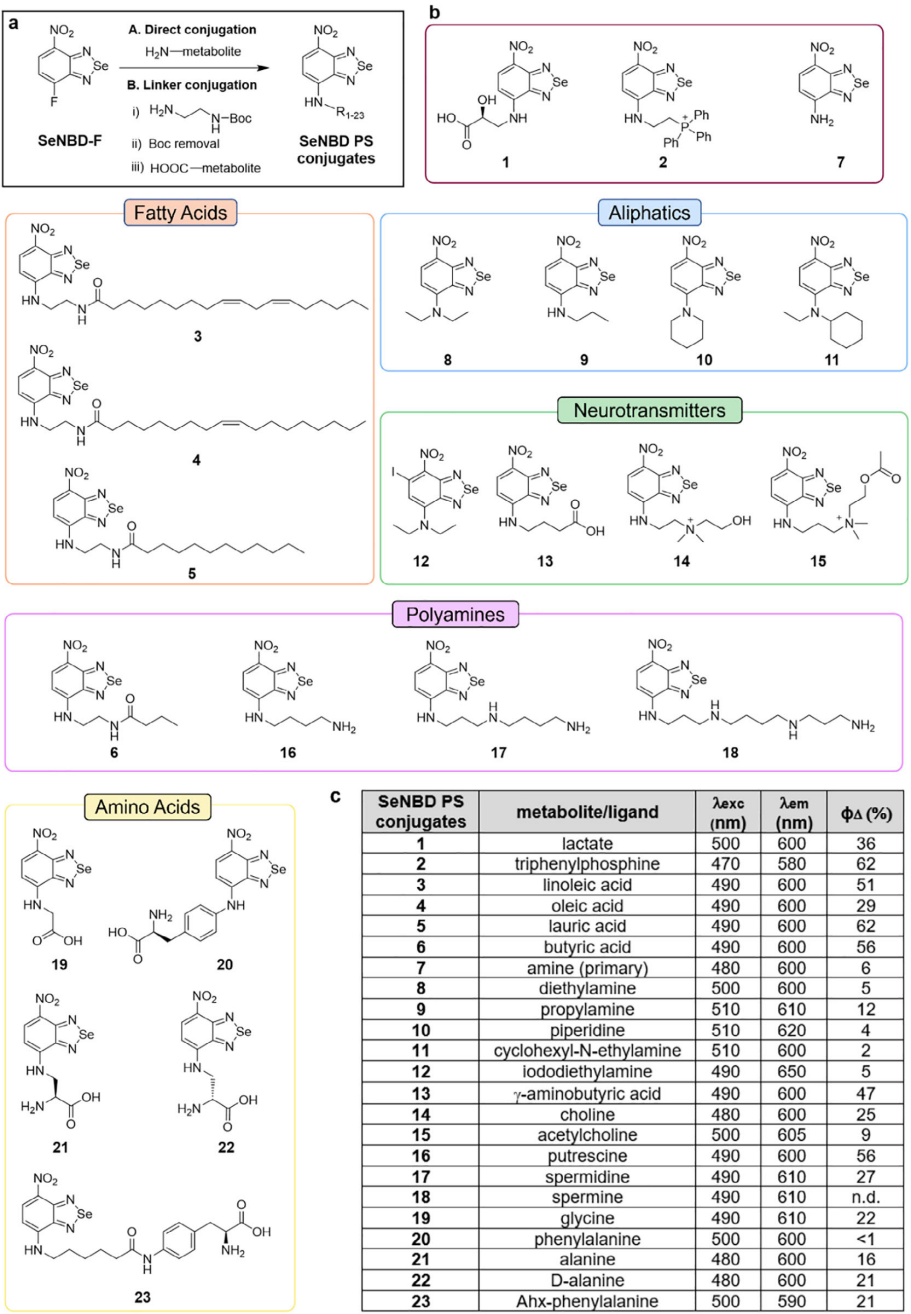
A collection of rationally‐designed, benzoselenadiazole‐metabolite conjugates. (a) Synthetic scheme of the PS collection from the common precursor **SeNBD‐F** using direct metabolite conjugation or linker‐mediated metabolite conjugation. (b) Chemical structures of SeNBD PS conjugates evaluated in this study. (c) Summary table including the photophysical properties of SeNBD PS conjugates, namely absorbance and emission maxima (200 µM, EtOH) and singlet oxygen generation quantum yields. Relative singlet oxygen generation quantum yields (ΦΔ) were determined for all compounds (5 µM) in ethanol using Rose Bengal (5 µM) as the reference.

A collection of 23 benzoselenadiazole compounds was synthesized in solution phase using the common precursor **SeNBD‐F** (Figure 1a,b), which was amenable to nucleophilic aromatic substitution with amino‐functionalized metabolites. For metabolites lacking nucleophilic functionalities (e.g., fatty acids), a short ethylenediamine linker was introduced between the benzoselenadiazole core and the metabolite structures. All compounds were prepared in less than 3 synthetic steps and isolated in high purities (full synthetic schemes and chemical characterization details in Supporting Information).

Next, we examined the optical and photodynamic properties of the entire collection of photosensitive metabolites. We determined their absorbance and emission spectra, extinction coefficients, fluorescence quantum yields, and generation of singlet oxygen ‐as one of the main effector ROS produced by benzoselenadiazole irradiation [50, 51] (Figure 1c and Table S1). Most metabolite conjugates exhibited typical absorbance and emission spectral profiles of amino‐functionalized benzoselenadiazoles (excitation ∼500 nm; emission ∼600 nm, Figure 1c). As expected for organic PS, fluorescence quantum yields were low, with values ranging between 1% and 5% (Table S1). On the other hand, singlet oxygen quantum yields were diverse, both including weak PS (e.g., compound **20** as a phenylalanine conjugate, <1%) as well as good PS (e.g., compound **5** as a lauric acid conjugate, 62%) (Figure 1c).

We found that linear aliphatic derivatives (e.g., compounds **8** and **9**) exhibited higher singlet oxygen production compared to their cyclic counterparts (e.g., compounds **10** and **11**), although the underlying reason remains unclear. Aromatic amines completely suppressed singlet oxygen production (e.g., compound **20** showed a singlet oxygen quantum yield below 1%), likely due to the quenching effect on the excited singlet and triplet states of the benzoselenadiazole core [52]. This effect was reversed when the benzoselenadiazole core and aromatic groups were separated by inclusion of an aminohexanoic acid spacer, as demonstrated by the higher photosensitivity of compound **23** (singlet oxygen quantum yield: 21%) when compared to compound **20** (singlet oxygen quantum yield: 1%). Finally, the incorporation of iodine atoms (i.e., compound **12**) led to red‐shifted emission but did not substantially increase singlet oxygen quantum yields (e.g., compounds **8** and **12** both showed values around 5%). Among the different classes of analogues, fatty acid derivatives (e.g., compounds **3–6**) showed the highest singlet oxygen quantum yields, ranging from 29% to 62%, with values ∼50% for each compound.

### 2.2 The Oleamide Derivative 4 is a Permeable PS with Phototoxicity in Patient‐Derived Glioma Cells

To characterize the photodynamic activity of benzoselenadiazole‐metabolite conjugates, we focused on 14 compounds with singlet oxygen quantum yields over 15%. For these experiments, we employed E17 cells as a representative patient‐derived glioblastoma stem cell (GSC) line, available from The Glioma Cellular Genetics Resource at the University of Edinburgh. The cellular toxicity of benzoselenadiazole compounds was assessed in the presence and absence of white light (10 mW cm^−2^) and their cell uptake was quantified by flow cytometry (Figure 2a). With these experiments, we aimed to identify new PS with good glioma cell uptake and high phototoxicity index (i.e., ratio between light‐mediated toxicity and dark toxicity).

**FIGURE 2:**
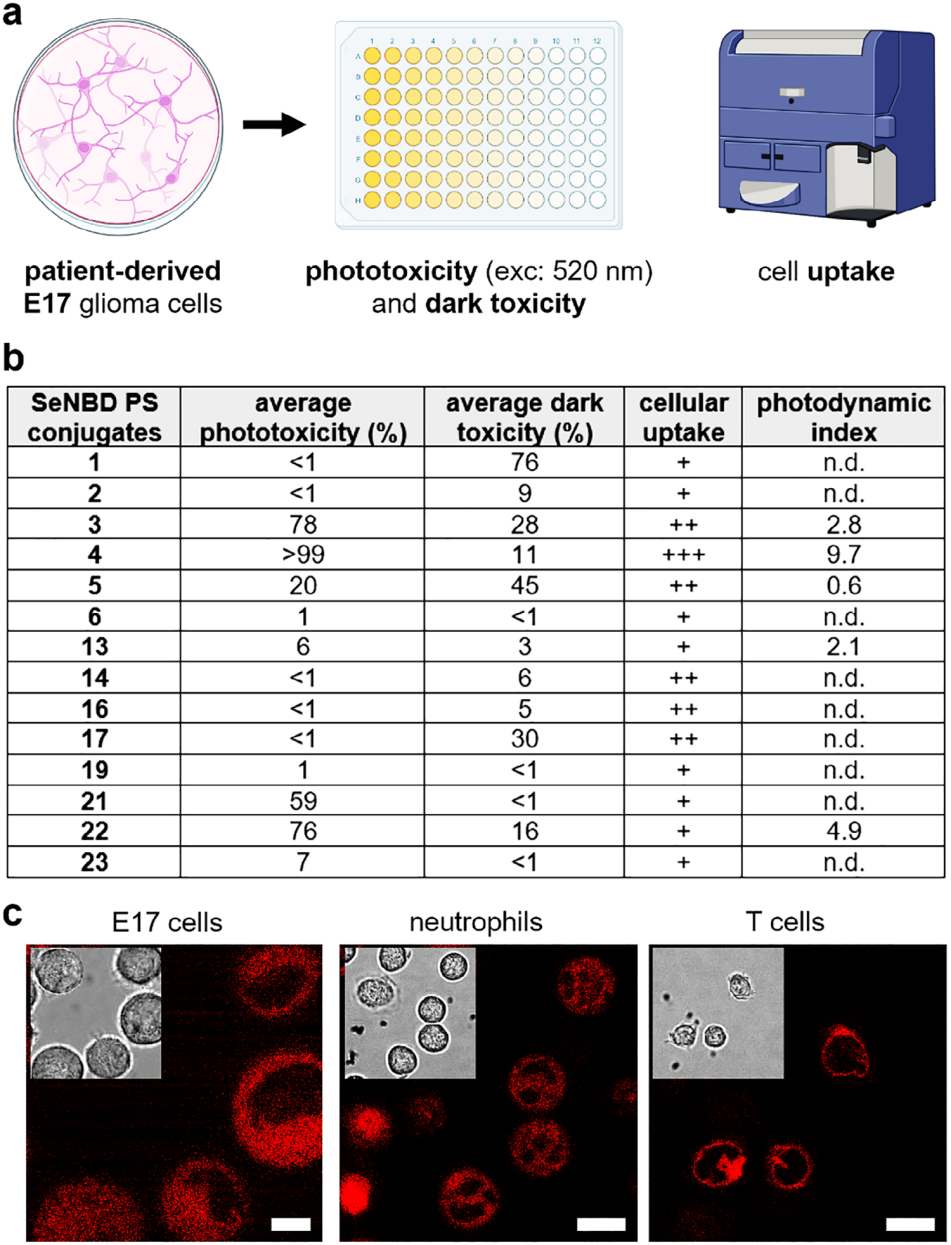
Evaluation of benzoselenadiazole PS conjugates in patient‐derived glioma cells. (a) Schematic illustration of the cellular assays to examine phototoxicity, dark toxicity, and cellular uptake in human brain cancer patient‐derived E17 cells. (b) Summary table of the 14 PS (all tested at 100 µM as a primary screen for hit identification) in the presence and absence of illumination (white light, 10 mW cm^−2^, 1 h), determined by resazurin assay in E17 cells (n = 3). Cellular uptake of all PS (1 h) in E17 cells (10,000 cells/well, n = 3) was measured by flow cytometry. Values presented as means ± SEM (n = 3). Semi‐quantitative MFI: mean fluorescence intensity (+++ for >5,000 fluorescence units, ++ for 1,000‐5,000 fluorescence units, + for <1,000 fluorescence units, n = 3). (c) Representative fluorescence and brightfield (inset) microscopy images of E17 glioma cells, human neutrophils and human T cells after incubation with **SeNBD‐oleamide** (10 µM, red) for 30 min at 37°C and 5% CO_2_. Experimental procedures detailed in the Supporting Information. Scale bars: 5 µm.

The 14 compounds exhibited diverse phototoxicity and dark toxicity values from 1% to 76%. Similarly, cellular uptake was variable, with fatty acids, choline, and polyamines showing the highest uptake in E17 cells. As shown in Figure 2b, cell uptake correlated well with light‐mediated cytotoxicity, with the exception of compounds **14**, **16** and **17**, which incorporated polyamines or ammonium salts. These compounds exhibited singlet oxygen quantum yields between 25% and 56% and reasonable cell uptake but were not phototoxic (i.e., below 1%), likely due to their differential intracellular distribution. Among the library, the fatty acid derivatives (compounds **3**–**6**) exhibited notable phototoxicity in E17 glioma cells and the highest photodynamic indexes (i.e., ratios of phototoxicity vs dark toxicity), and these compounds were selected for further biological analysis.

The fatty acid derivative **4** (hereinafter, **SeNBD‐oleamide**), which contains an 18‐carbon‐long chain with a cis double bond at the ninth carbon, showed the highest phototoxicity among all benzoselenadiazole‐metabolite conjugates. Meanwhile, the lauric acid analogue (compound **5**), a 12‐carbon medium‐chain saturated fatty acid, showed moderate phototoxicity (20%), while butyric acid (compound **6**) demonstrated lower phototoxicity (1%). In view of these results, we hypothesized that long and minimally unsaturated fatty acids could be taken up to a larger extent, and we verified this observation with cell microscopy experiments using fatty acid conjugates of the fluorescent benzoxadiazole (O‐NBD) (Figure S1; full synthetic and characterization details in Supporting Information). Fluorescence microscopy in E17 cells confirmed that **ONBD‐oleamide** was taken up to a larger degree than ONBD‐metabolite conjugates with shorter aliphatic chains (i.e., compounds **29** and **30**; Figure S1). We also confirmed that the incubation with **SeNBD‐oleamide** followed by light illumination generated ROS inside cells, as demonstrated by staining with the commercial dye 2ʼ,7ʼ‐dichlorodihydrofluorescein diacetate (DCFH‐DA, Figure S2).

Furthermore, we quantified the phototoxicity of **SeNBD‐oleamide** in three independent patient‐derived GSC lines [53] (e.g., E17: classical transcriptional subclass, E31: proneural transcriptional subclass, E57: mesenchymal transcriptional subclass). Notably, in all three GSC lines, we determined half‐maximal inhibitory concentrations in the high nanomolar‐low micromolar range (i.e., IC_50_ values: 0.6 ± 0.3 µM for E17, 3.1 ± 1.0 µM for E31, and 1.3 ± 0.1 µM for E57, Figure S3), which corroborates the utility of oleic acid as an effective moiety to deliver benzoselenadiazole PS into human glioblastoma cells. Finally, we employed fluorescence microscopy to compare the uptake of **SeNBD‐oleamide** in E17 cells and in immune cells (neutrophils and T cells) that are abundant in brain tumors. As shown in Figure 2c, we observed internalization of **SeNBD‐oleamide** in glioma as well as in noncancerous immune cells, highlighting the need for additional biomarker‐activatable groups to selectively release our activatable PS in glioma cells.

### 2.3 Fine‐Tuning the Synthesis of a Glioma‐Targeting Activatable PS

In order to improve the glioma‐targeting capabilities of **SeNBD‐oleamide**, we searched for targets with significantly higher expression in GSC lines than normal human neural stem cells (hNSCs). Our transcriptomic results indicated that *GLB1*, the gene encoding for the enzyme β‐galactosidase [54, 55, 56], was significantly increased in GSCs compared to hNSCs (Figure S4). In view of this, we envisioned that β‐galactosidase substrates could be utilized to develop a glioma‐targeted PS that incorporated a β‐galactosidase‐activatable caging group into the chemical structure of **SeNBD‐oleamide**. The rational design of the activatable **Gal‐SeNBD‐oleamide** consisted of four chemical moieties: 1) D‐galactose as a substrate for β‐galactosidase, 2) a carbamate caging unit to block the production of ROS in the absence of β‐galactosidase, 3) a self‐immolative linker [57] for PS release upon reaction with β‐galactosidase, and 4) **SeNBD‐oleamide** as a highly efficient PS (Figure 3a).

**FIGURE 3:**
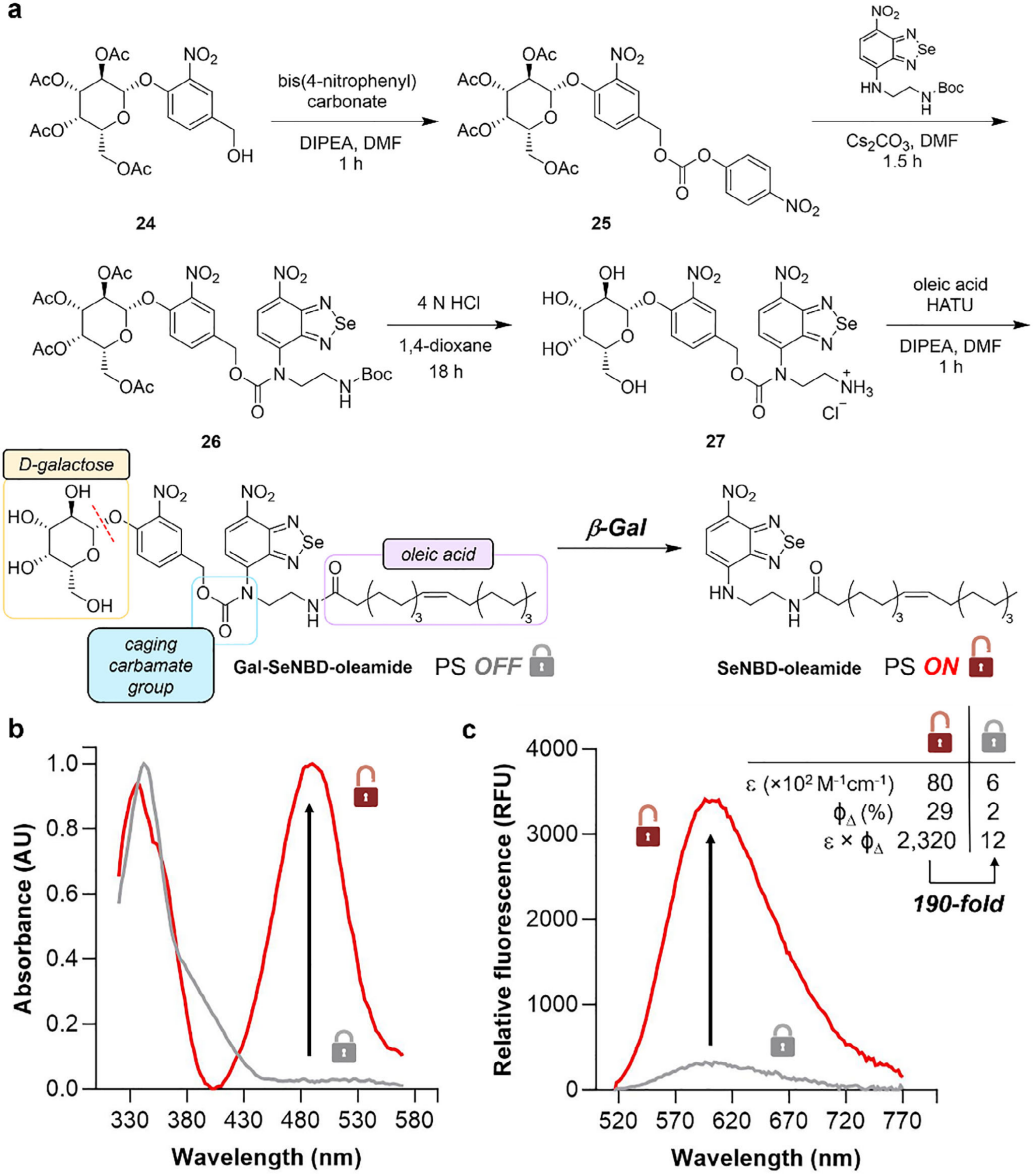
Chemical design, synthesis, and photophysical characterization of Gal‐SeNBD‐oleamide. (a) Synthetic scheme for the preparation of **Gal‐SeNBD‐oleamide**. The rational design of **Gal‐SeNBD‐oleamide**, comprising four key chemical moieties: 1) a D‐galactose as a substrate for β‐galactosidase, 2) a carbamate caging group to modulate ROS production, 3) a self‐immolative linker for release of the PS, and 4) **SeNBD‐oleamide** as the active PS. The red dotted line represents the bond cleaved upon activation by β‐galactosidase for concomitant 1,6‐elimination and **SeNBD‐oleamide** release. (b, c) Representative absorbance spectra (b) and fluorescence emission spectra (c) of **Gal‐SeNBD‐oleamide** (grey lines) and **SeNBD‐oleamide** (red lines) (both at 20 µM, *λ*_ex_ = 490 nm). Molar extinction coefficients (ε, 490 nm) were determined as 8 × 10^3^ M^−1^ cm^−1^ for **SeNBD‐oleamide** and 6 × 10^2^ M^−1^ cm^−1^ for **Gal‐SeNBD‐oleamide**. Relative singlet oxygen generation quantum yields (Φ_Δ_) were determined in ethanol with Rose Bengal (5 µM) as the reference.

We started the chemical synthesis of **Gal‐SeNBD‐oleamide** with the nucleophilic substitution reaction between a protected D‐galactose derivative (**24**) and bis(4‐nitrophenyl) carbonate to produce the p‐nitrophenyl carbonate **25**. Compound **25** was reacted with tert‐butyloxycarbonyl (Boc)‐protected ethylenediamine‐SeNBD to generate the corresponding caged carbamate **26**. The Boc and acetyl protecting groups of compound **26** were fully removed in one single treatment with 4 N HCl in 1,4‐dioxane, yielding the D‐galactose‐containing compound **27** with a free primary amine group for final derivatization with oleic acid. Compound **27** was conjugated to oleic acid using hexafluorophosphate azabenzotriazole tetramethyl uronium (HATU) as the coupling reagent [58, 59] (Figure 3a). The final **Gal‐SeNBD‐oleamide** was isolated after purification by preparative HPLC with purities >95% (full synthetic details and characterization data in the Supporting Information).

Having synthesized the β‐galactosidase activatable **Gal‐SeNBD‐oleamide**, we examined its optical properties and compared them to the noncaged **SeNBD‐oleamide** (Figure 3b,c). **Gal‐SeNBD‐oleamide** showed full blockage of absorbance at the maximum excitation wavelength of the benzoselenadiazole scaffold with extremely low extinction coefficients (e.g., 600 M^−1^ cm^−1^ at 490 nm) and minimal fluorescence emission in the red region of the spectrum, which is critical to monitor PS release with good signal‐to‐noise ratios. Furthermore, we also confirmed that ‐unlike its noncaged precursor **SeNBD‐oleamide**‐ **Gal‐SeNBD‐oleamide** did not produce high levels of singlet oxygen upon illumination, highlighting its value as an activatable PS. Specifically, **SeNBD‐oleamide** exhibited singlet oxygen generation quantum yields around 30%, while the caged **Gal‐SeNBD‐oleamide** showed singlet oxygen generation quantum yields around 2%. Altogether, these results confirmed that the introduction of D‐galactose to the benzoselenadiazole core via a carbamate self‐immolative linker represents an effective strategy to modulate its photodynamic activity. To the best of our knowledge, this is the first example of an enzyme‐activatable PS based on the benzoselenadiazole core and including a substrate for selective uncaging in glioma cells.

### 2.4 Gal‐SeNBD‐oleamide can be Activated in Human Glioma Cells but not in Human Immune Cells

We examined the time‐lapse reactivity between **Gal‐SeNBD‐oleamide** and human β‐galactosidase. After optimizing a suitable formulation for in vitro experiments (Figure S5), we observed that human β‐galactosidase reacted with **Gal‐SeNBD‐oleamide** to release **SeNBD‐oleamide**, as denoted by HPLC‐MS analysis (Figure S6) with a substantial increase in fluorescence emission around 600 nm (Figure 4a). Importantly, uncaging of **Gal‐SeNBD‐oleamide** was not detected in the presence of proteases that are commonly found in the tumor microenvironment, namely matrix metalloproteinases, cathepsins, neutrophil elastase, and granzymes [60, 61, 62, 63, 64] (Figure 4b) or in different intracellular environments (e.g., variable pH, oxidative or reducing environments) [65]. We also corroborated that the activation of **Gal‐SeNBD‐oleamide** by human β‐galactosidase was accompanied by a substantial increase in ROS generation due to the release of **SeNBD‐oleamide** (Figure 4b).

**FIGURE 4:**
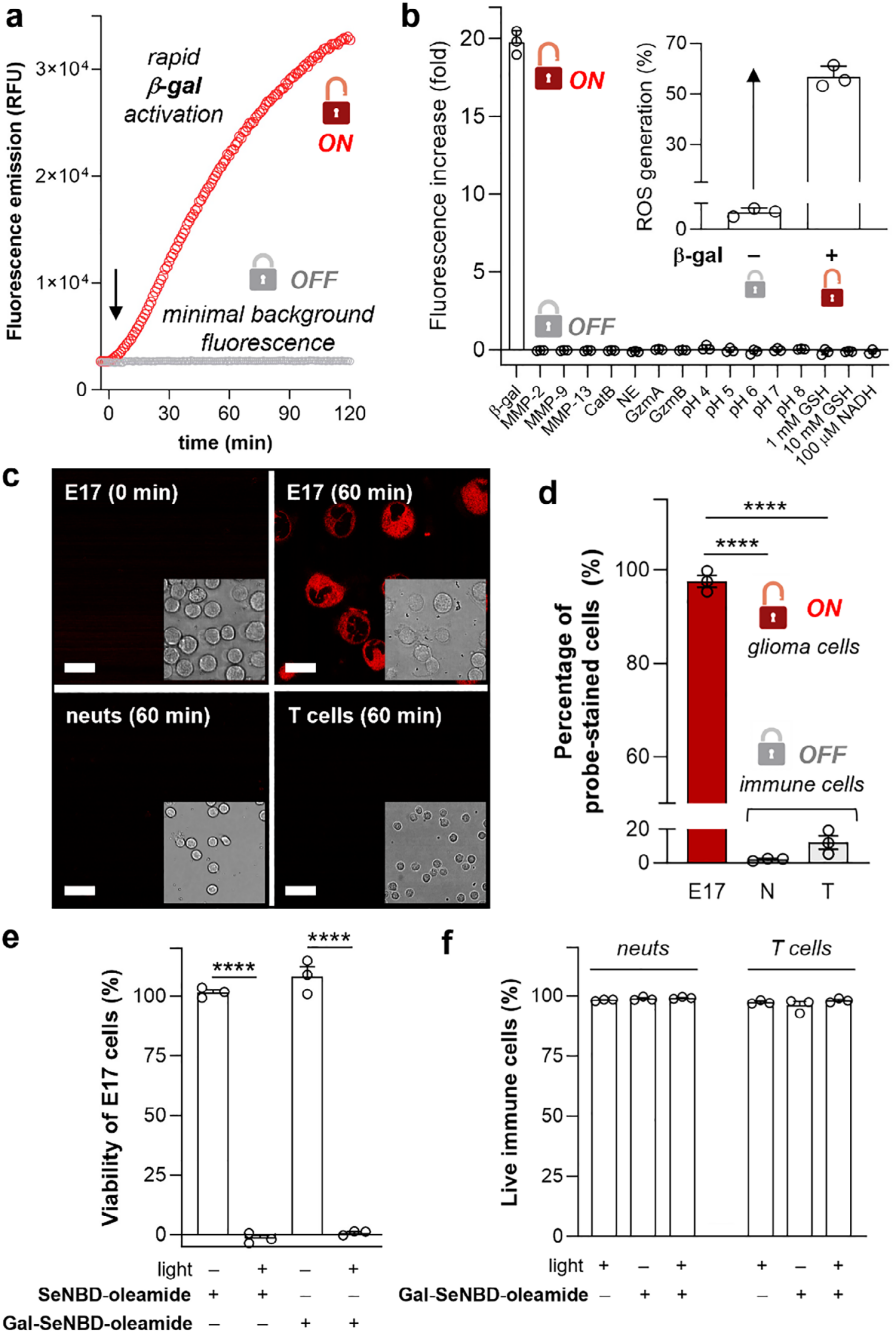
Gal‐SeNBD‐oleamide is activated in human glioma cells but not in human immune neutrophils or T cells. (a) Time‐lapse fluorescence emission (600 nm, 37 °C) of **Gal‐SeNBD‐oleamide** (10 µM) in the absence (grey circles) or presence (red circles) of β‐galactosidase (100 nM, aqueous buffer, 20% captisol, arrow indicates addition of enzyme). Values presented as means ± SD (n = 3). RFU: relative fluorescence units. (b) Relative fluorescence fold increase of **Gal‐SeNBD‐oleamide** (10 µM) after incubation at 37°C with enzymes (100 nM, 90 min), pH buffers and bioanalytes (exc.: 520 nm, em.: 590 nm). Values as means ± SD (n = 3). Inset) ROS production of **Gal‐SeNBD‐oleamide** (10 µM) in the absence/presence of β‐galactosidase (100 nM) by measuring the reduction of DPBF absorbance at 410 nm. Values as means ± SD (n = 3). (c) Representative fluorescence and brightfield (inset) microscopy images of E17 cells, human neutrophils and human T cells during incubation with **Gal‐SeNBD‐oleamide** (10 µM) for 60 min at 37°C (exc.: 520 nm, em.: 600 ± 50 nm, Movie S1). Scale bars: 10 µm. (d) Flow cytometry analysis of E17 cells (red bar), human neutrophils (N, grey) and human T cells (T, grey) after incubation with **Gal‐SeNBD‐oleamide** (10 µM) for 60 min at r.t. Values as means ± SD (n = 3). Statistical analysis using one‐way ANOVA. (e) Phototoxicity assays in E17 cells (resazurin assays) after incubation with **SeNBD‐oleamide** and **Gal‐SeNBD‐oleamide** (both at 10 µM) for 60 min at 37°C and absence/presence of light irradiation (15 mW cm^−2^, 520 nm, 60 min). Values as means ± SD (n = 3). Statistical analysis using one‐way ANOVA. **** for p < 0.0001. (f) Phototoxicity assays in neutrophils and T cells (flow cytometry) after incubation with **Gal‐SeNBD‐oleamide** (10 µM) for 60 min at 37°C and absence/presence of light irradiation (15 mW cm^−2^, 520 nm, 60 min). Values as means ± SD (n = 3).

Encouraged by these results, we examined the activation of **Gal‐SeNBD‐oleamide** in E17 glioma cells, first by time‐lapse fluorescence confocal microscopy under wash‐free conditions. **Gal‐SeNBD‐oleamide** exhibited very low fluorescence background and progressive increase in intracellular fluorescence (Figure 4c and Movie S1). Comparative experiments using fluorescence microscopy (Figure 4c) and flow cytometry (Figure 4d and Figure S7) confirmed that **Gal‐SeNBD‐oleamide** did not activate in these immune cells, which are commonly found in brain tumors [66, 67]. We also observed that **Gal‐SeNBD‐oleamide** can activate in small subpopulations of cells that express β‐galactosidase [68, 69, 70, 71], such as human monocyte‐derived macrophages (Figure S8). Next, we confirmed that the selective activation of **Gal‐SeNBD‐oleamide** in E17 cells led to targeted ablation of glioma cells versus immune cells. In these experiments, we measured the cytotoxicity in all cells and observed that the combination of **Gal‐SeNBD‐oleamide** and light irradiation selectively ablated E17 glioma cells but did not cause any significant cytotoxicity in neutrophils or T cells (Figure 4e,f). These results corroborate the preferential activation of **Gal‐SeNBD‐oleamide** in glioma cells and its potential for targeted PS release in brain tumors.

In order to study the importance of the oleic acid group for internalization to glioma cells, we synthesized compound 27 (hereinafter, **Gal‐SeNBD‐NH_2_**) as a control compound containing the same benzoselenadiazole PS and D‐galactose carbamate caging unit but lacking the fatty acid moiety (full synthetic and characterization details in Supporting Information). Notably, **Gal‐SeNBD‐NH_2_** was unable to enter E17 glioma cells, as shown by the lack of intracellular fluorescence signals (Figure S9), which indicated the critical role of the oleamide moiety for internalization to human glioma cells.

Finally, we confirmed that the activation of **Gal‐SeNBD‐oleamide** followed by light irradiation produced ROS (in experiments with 1,3‐diphenylisobenzofuran (DPBF) and DCFH‐DA, Figures 4b and S10, respectively), and we compared the photodynamic activity of **Gal‐SeNBD‐oleamide**, **SeNBD‐oleamide**, and the clinically approved 5‐ALA. For **Gal‐SeNBD‐oleamide** and **SeNBD‐oleamide**, we observed minimal dark toxicity and high levels of toxicity in E17 glioma cells after they had been incubated with the PS and exposed to light (520 nm, 15 mW cm^−2^, 60 min), with **Gal‐SeNBD‐oleamide** achieving near‐complete eradication of glioma cells at 10 µM and comparable efficacy to **SeNBD‐oleamide** (Figure 4e). On the other hand, the same experimental conditions showed limited phototoxicity in glioma cells when 5‐ALA was used as the PS (Figure S11). Altogether, these results highlight the potency of benzoselenadiazole PS at micromolar concentrations and highlight the potential application of **Gal‐SeNBD‐oleamide** as an enzyme‐activatable PS for photodynamic ablation of glioblastoma cells in brain tissues.

### 2.5 Real‐Time Monitoring of Activatable PS Release in Glioma Cells

The effective translation of activatable PS for PDT relies on the application of a suitable light dosage upon PS release; however, this step requires characterization of the kinetics in cell uptake and enzymatic activation, which can vary between tissues and patients. We envisioned that PDT illumination equipment integrating both spectroscopy modules and dosing of light in a single instrument would enhance the versatility of PDT systems, which typically do not incorporate spectroscopy modules for PS release [72].

To address this gap, we designed a component‐based system combining on‐the‐fly fluorescence spectroscopy (to monitor the release of **SeNBD‐oleamide**) and PDT illumination (to activate **SeNBD‐oleamide**) (Figure S12 and Table S2). The optics enabled fiber‐coupled, co‐aligned light collection for monitoring and dosimetry, both powered by the same light source. To match the excitation peak of **SeNBD‐oleamide**, we used a 520 nm laser diode with an available power of 50 mW and coupled it to a multimode optical fiber (400 µm diameter) via aspheric lenses with 70% efficiency. Using this design, the fluorescence signals of **SeNBD‐oleamide** were collected via a separate optical path to minimize background autofluorescence and enable direct monitoring of PS activation.

First, we evaluated the performance of the device by characterizing the illumination output and stability to confirm uniform irradiation over an adjustable area matching the well diameter (e.g., 7 mm), which was sufficient to flood illuminate the brain tissue slices (Figure 5a). We also confirmed the progressively brighter fluorescence signals of increasing concentrations of **SeNBD‐oleamide** using low‐power excitation (Figure S13). Next, we confirmed that the illumination of human glioma cells using our device (i.e., up to 30 mW cm^−^^2^ for 60 min) did not induce any toxicity in the absence of PS (Figure S14), thus indicating good biocompatibility. Finally, we were able to monitor the activation of **Gal‐SeNBD‐oleamide** in E17 glioma cells in real time using low‐power excitation (2.5 mW cm^−2^) to minimize any potential photobleaching. As shown in Figure 5b, these experiments confirmed very low background levels of **Gal‐SeNBD‐oleamide** at early time points and a gradual increase in fluorescence emission because of its intracellular activation.

**FIGURE 5:**
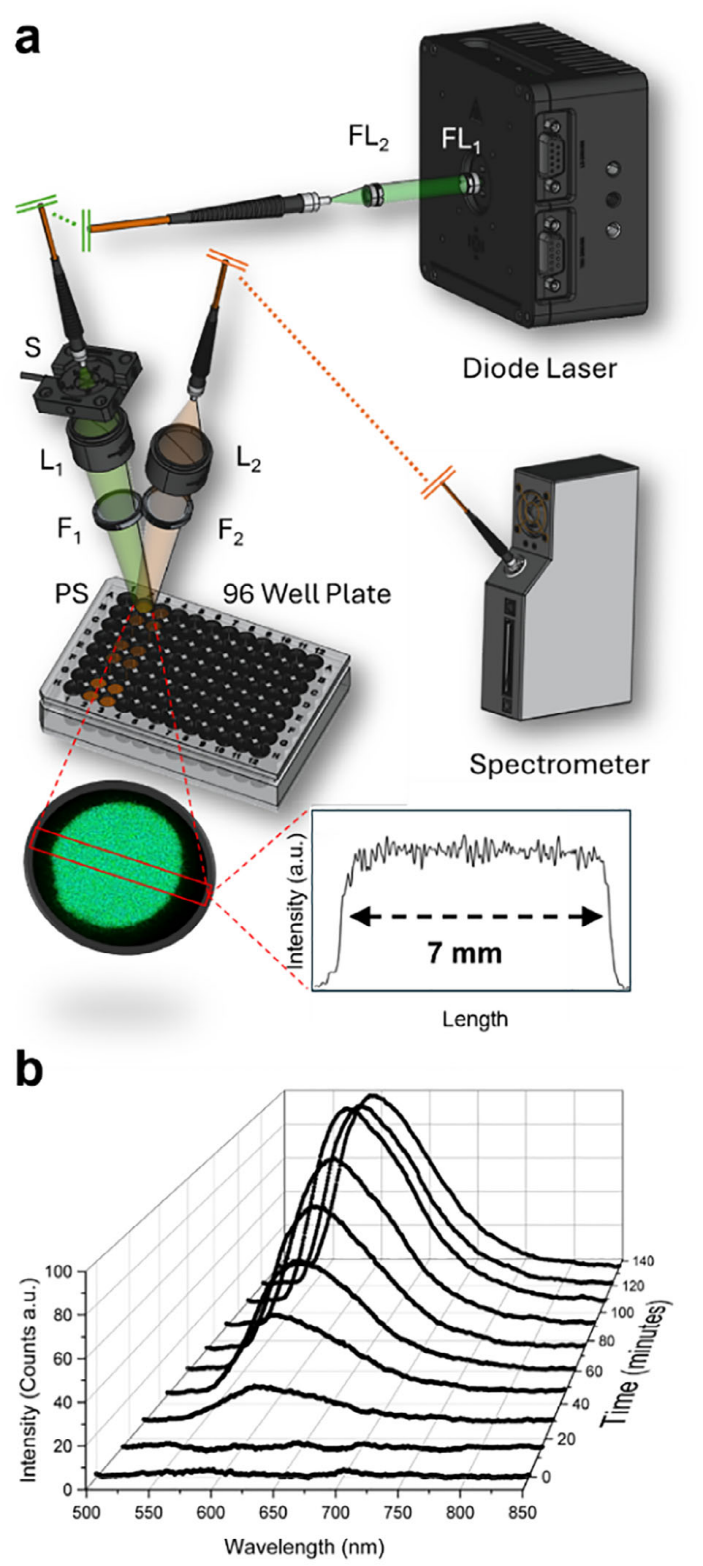
Real‐time monitoring of SeNBD‐oleamide release in human glioma cells. (a) Component based on‐the‐fly fluorescence spectroscopy and PDT dosimetry system. Emission from a TO‐Can laser diode via aspheric lenses *FL_1_* and *FL_2_* into a 400 µm diameter step index optical fiber. The fiber emission facet is reimaged onto the sample via lens *L_1_*, through band‐pass filter *F_1_*. A computer‐controlled shutter *S* blocks illumination when not required. Fluorescence emission is coupled (via *F_2_* and *L_2_*) to an identical fiber returning to a commercial spectrometer. Laser emission power, shutter, and spectrometer acquisition are automated for data collection via a graphical user interface giving real‐time observation of PDT progression. Diode and filters are interchangeable for use with other PS. Inset shows illumination profile across well‐defined exposure region. (b) Measurements of **Gal‐SeNBD‐oleamide** (10 µM) activation in E17 cells for 2 h (2.5 mW cm^−2^, exc.: 490 nm, em.: 500–850 nm).

### 2.6 Gal‐SeNBD‐oleamide Causes Cell Death in Tumor Regions of Mouse and Human Brain Tissues

Having optimized an illumination device for **Gal‐SeNBD‐oleamide**, we employed a genetically engineered mouse glioma model to investigate its selectivity and PDT activity in mouse brain tissues. We used a well‐established preclinical model whereby adult mouse neural stem cells efficiently induce Green Fluorescent Protein (GFP)‐expressing glioma‐like tumors following orthotopic transplantation [73]. Upon tumor formation, we harvested brain tissues, which included GFP‐expressing tumor regions (right hemisphere) and non‐tumorigenic regions (left hemisphere). Tissues were embedded in low‐melting agarose without any fixatives and sectioned to generate slices of ∼300 µm thickness (Figure 6a,b).

**FIGURE 6:**
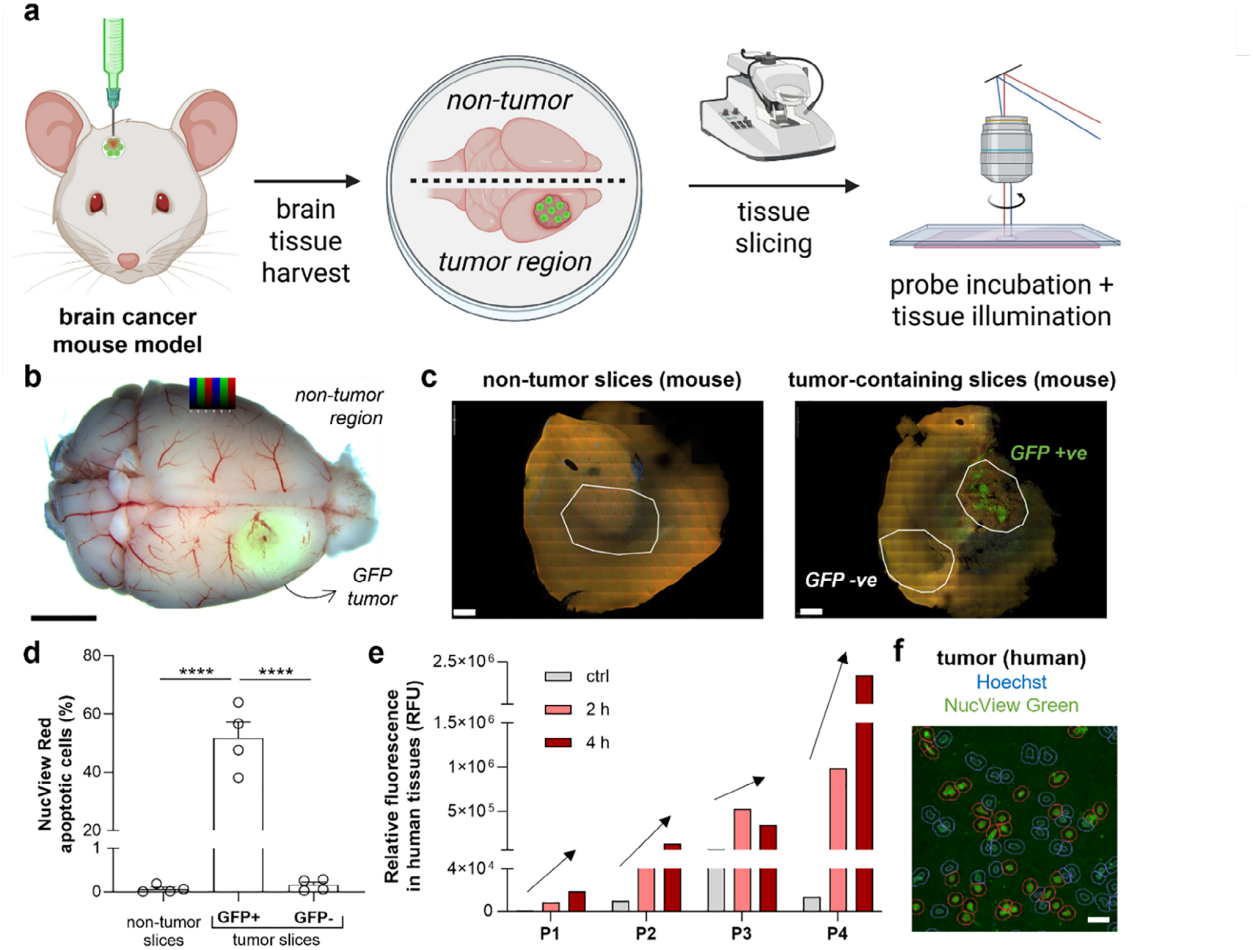
Gal‐SeNBD‐oleamide enables PDT‐mediated cell ablation in tumor regions of mouse and human brain tissues. (a) Schematic illustration of the preclinical mouse model of brain cancer, including neural stem cell implantation, tissue harvesting, and processing. (b) Representative whole brain tissue, highlighting both tumor and non‐tumor regions and GFP‐positive tumor cells. Scale bar: 3 mm. (c) Representative fluorescence confocal microscopy stitched images of non‐tumor mouse brain slices (left) and mouse brain slices (right) showing GFP‐expressing tumor cells. Fluorescence images were obtained for GFP (green, exc.: 488 nm, em.: 522 nm) and the apoptosis marker NucView Red (red, exc.: 561 nm, em.: 599 nm). Exemplar regions for image analysis are highlighted in white. Scale bars: 100 µm. (d) Quantification of NucView Red‐stained apoptotic cells in non‐tumor regions, GFP‐positive tumor regions and GFP‐negative regions. Results include 4 independent mouse experiments. Values presented as means ± SD (n = 4). P values determined using ANOVA (**** for *p*<0.0001). (e) Time‐course fluorescence emission obtained with the illuminator shown in Figure 5 of human brain tissue slices from 4 human patients (P1‐P4) upon incubation with **Gal‐SeNBD‐oleamide** (20 µM). Control grey bars correspond to the time point for the addition of **Gal‐SeNBD‐oleamide**, with pink and red bars corresponding to values after probe incubation for 2 h and 4 h, respectively. (f) Representative fluorescence confocal microscopy image of human brain tumor slices after treatment with **Gal‐SeNBD‐oleamide** (20 µM) and illumination (520 nm, 25 mW cm^−2^, 60 min). Cells counterstained with Hoechst 33342 (blue, exc.: 375 nm, em.: 456 nm) and apoptotic cells were labeled with NucView Green (green, exc.: 488 nm, em.: 522 nm).

In order to monitor the release of **SeNBD‐oleamide** directly in mouse brain slices, we incubated the tissue slices with **Gal‐SeNBD‐oleamide** (20 µM) and monitored the fluorescence emission in the illuminator at 37°C in the dark. Under these conditions, we observed a gradual increase of emission in all brain tumor slices, with fluorescence emission plateauing after 4 h (Figure S15). In line with this result, we decided to incubate non‐tumor and tumor brain tissue slices with **Gal‐SeNBD‐oleamide** for a maximum of 4 h, followed by PDT illumination at 25 mW cm^−^^2^ for 60 min. In addition to the GFP signals from cancer cells, we stained all mouse brain tissues with Hoechst 33342 (for nuclei counterstaining) and NucView Red (for quantification of apoptotic cells).

We determined the photodynamic activity of **Gal‐SeNBD‐oleamide** after illumination by image analysis. First, we identified tumor regions using the GFP signals found in tumor cells, then we compared the levels of apoptosis in both tumor and non‐tumor regions of the same tissue slice as well as in its corresponding non‐tumor brain tissue slice (Figures 6c and S16 for detailed step‐by‐step image analysis). Notably, we observed significantly higher levels of NucView Red‐stained apoptotic cells in GFP‐positive tumor regions compared to neighboring GFP‐negative non‐tumor regions and GFP‐negative non‐tumor slices (Figures 6d and S17; Table S3). These results indicate that direct incubation of **Gal‐SeNBD‐oleamide** in brain tissues from a preclinical mouse model of brain cancer leads to selective PDT‐mediated toxicity in tumor regions with minimal effect in non‐tumor areas.

Finally, we evaluated the translational potential of **Gal‐SeNBD‐oleamide** in human brain tissues, which were resected from a small cohort of glioblastoma patients. Material provision and use was covered by Lothian NRS BioResource RTB approval (REC ref: 20/ES/0061; project reference: SR2216), whereby written informed consent was obtained from all participants. First, brain tissue slices (sectioned at ∼300 µm thickness and cultured as indicated above) were incubated with **Gal‐SeNBD‐oleamide** (20 µM), and their fluorescence signals were monitored over time using our illuminator. Despite patient‐to‐patient variability, most brain tumors showed notable fluorescence increase after a few hours, indicating successful activation of **Gal‐SeNBD‐oleamide** (Figure 6e). Having released the active PS, we performed PDT illumination (i.e., 25 mW cm^−^^2^ for 60 min) followed by Hoechst 33342 and NucView Green staining for image analysis under fluorescence microscopy. As shown in Figures 6f and S18, we found high levels of apoptosis in brain tumor slices that had been treated with both **Gal‐SeNBD‐oleamide** and light ‐as opposite to untreated tumor slices‐ which indicates the potential of **Gal‐SeNBD‐oleamide** for tumor ablation directly in brain cancer tissues.

## 3 Conclusions

In summary, we have synthesized a chemical collection of 23 benzoselenadiazole‐metabolite conjugates and evaluated their photodynamic activity in patient‐derived glioblastoma cell lines. Derivatives of fatty acids (in particular, the oleic acid analogue **SeNBD‐oleamide**) showed the best photodynamic index with excellent uptake in E17 glioma cells; however, they showed nonselective internalization in immune cells like neutrophils and T cells. The selectivity of **SeNBD‐oleamide** for glioma cells was enhanced by inclusion of a β‐galactosidase‐responsive moiety to generate **Gal‐SeNBD‐oleamide**. This caged PS showed a 190‐fold decrease in photodynamic ability and rapid release of the PS after reaction with β‐galactosidase in glioma cells but not in immune cells. Finally, we employed an illuminator for direct PDT in whole‐brain tissue slices and demonstrated the utility of **Gal‐SeNBD‐oleamide** (summarized in Table S4) for selective PDT‐mediated killing in tumor regions using a preclinical mouse model of brain cancer and in tissue biopsies from human glioblastoma patients.

## Author Contributions

**Utsa Karmakar**: investigation, writing – review and editing, data curation, validation. **Zhiming Cheng**: investigation, writing – original draft, data curation, validation. **Andrew D M Green**: investigation, data curation. **Sam Benson**: investigation, data curation. **Eleni Nestoros**: investigation and data curation. **Clarissa Lim**: investigation. **Justyna Cholewa‐Waclaw**: investigation. **Fabio de Moliner**: investigation. **Paula Lusarreta**: investigation. **Deborah Seah**: investigation. **Alsadeg Bilal**: resources. **Neza Alfazema**: resources. **Thibaud Aymoz‐Bressot**: resources. **Gillian Morrison**: resources. **Sonja Vermeren**: resources. **Steven M Pollard**: resources. **Paul M Brennan**: resources. **Michael G Tanner**: conceptualization, writing – review and editing, supervision. **Marc Vendrell**: conceptualization, funding acquisition, writing – original draft, writing – review and editing, supervision.

## Conflicts of Interest

The authors declare no conflicts of interest.

## Supporting information



Materials and methods; experimental details; synthetic procedures and full chemical characterization data (^1^H NMR spectra, ^13^C NMR spectra and HRMS data); supplementary data including: microscopy cell images of benzoxadiazole analogues of fatty acid conjugates (Figure S1), DCFH‐DA experiments with **SeNBD‐oleamide** (Figure S2), half‐maximal inhibitory concentrations in different glioma cell lines (Figure S3), transcriptomic analysis of human β‐galactosidase gene (*GLB1*, Figure S4), optimization of **Gal‐SeNBD‐oleamide** formulation (Figure S5), HPLC‐MS analysis of **Gal‐SeNBD‐oleamide** (Figure S6), flow cytometry gating strategy (Figure S7), experiments in human monocytes (Figure S8), fluorescence experiments with **Gal‐SeNBD‐NH_2_** in E17 cells (Figure S9), DCFH‐DA experiments with **Gal‐SeNBD‐oleamide** (Figure S10), cytotoxicity experiments with 5‐ALA (Figure S11), in‐house illumination and spectroscopy system (Figure S12) with fluorescence emission data (Figure S13) and cytotoxicity analysis (Figure S14), fluorescence emission in mouse brain tissue slices (Figure S15), image data analysis in mouse brain tissue slices (Figure S16), NucView Red quantification in control slices (Figure S17) and apoptosis imaging in human tumor tissues from brain cancer patients (Figure S18).**Supporting File 1**: anie75034‐sup‐0001‐Movie1‐Vendrell.avi.

**Supporting File 2**: anie75034‐sup‐0002‐SuppMat.pdf.

## Data Availability

The data that support the findings of this study are available on request from the corresponding author. The data are not publicly available due to privacy or ethical restrictions.
