# Supplementary material for: Sequential Design of Activatable Photosensitizers for Ablation of Glioma Cells in Brain Cancer Tissues
Source: Angew Chem Int Ed Engl. 2026 Sep 30;65(46):e8453410. doi: 10.1002/anie.8453410 (PMC13749316; doi:10.1002/anie.8453410)
Supplement: Supplementary file 1 [file ANIE-65-e8453410-s002.pdf]

Electronic Supporting Information

Sequential Design of Activatable Photosensitizers for Ablation of Glioma Cells in Brain Cancer Tissues

Table of Contents

1. Materials and Methods
2. Experimental Procedures
3. Supplementary Figures and Tables
4. Supplementary Movie Legends
5. NMR Spectra
6. Supplementary References

1. Materials and Methods

Commercially available reagents were used without further purification. All solvents were commercially available and used without purification. Thin-layer chromatography was conducted on Merck silica gel 60 F254 sheets and visualized by UV (254 and 365 nm). Silica gel Merck 60 (particle size 40-63 μm) or Teledyne ISCO (eluent systems as indicated) were used for column chromatography. Preparative HPLC was performed using an Agilent Technologies 1260 system with a Kinetex C_{18} 150 × 21.2 mm column. Eluents: H_2O (0.1% CF_3CO_2H) and ACN (0.1% CF_3CO_2H). Flow: 20.0 mL min^{-1}. Synthesis reactions and products were monitored by HPLC-MS using a HPLC Agilent Technologies 1200 with a Kinetex C_{18} 50 × 4.6 mm column and a diode array detector. Eluents: H_2O (0.1% HCOOH) and ACN (0.1% HCOOH). Flow: 1.0 mL min^{-1}. The MS detector was configured with an electrospray ionization source and nitrogen was used as the nebuliser gas. cLogP values were estimated using the software Molinspiration. ^{1}H-NMR and ^{13}C-NMR spectra were recorded using Bruker Avance 500 spectrometer (at 500 and 126 MHz, respectively) equipped with a 5 mm helium-cooled BBO cryoprobe. NMR data was processed using Mestrenova version 16.0.0. Chemical shifts were reported in ppm on the δ scale relative to a residual solvent ($CDCl_3$: δ = 7.26 for ^{1}H-NMR and 77.2 for ^{13}C-NMR, CD_3OD: δ = 3.31 for ^{1}H-NMR and 49.0 for ^{13}C-NMR, DMSO-d_6: δ = 2.50 for ^{1}H-NMR and 39.5 for ^{13}C-NMR, D_2O: δ = 4.79 for ^{1}H-NMR) Multiplicities are referred to by the following abbreviations: s = singlet, d = doublet, t = triplet, dd = doublet doublets, ddd = double double doublet, dt = double triplet, q = quartet, p = pentet and m = multiplet.

2. Experimental Procedures

Photophysical measurements. Spectroscopic data were recorded on a Synergy HT spectrophotometer and data analysis was performed using GraphPad Prism 5.0. Compounds were dissolved at the indicated concentrations and spectra were recorded at r.t. Molar absorbance readings were taken for samples (6.25-100 μM) at 490 nm using a NanoDrop™ One/OneC Microvolume UV-Vis Spectrophotometer. Readings (3 replicates) were plotted against concentration and the extinction coefficient was calculated using the Beer-Lambert law:

$$A = \epsilon c l$$

where A is the absorbance at a specified wavelength, ϵ is the extinction coefficient (molar absorbance M^{-1} cm^{-1}), c is the sample concentration and l is the path length (in cm).

Singlet oxygen quantum yields. SOQY measurements were taken for **SeNBD-oleamide** and **Gal-SeNBD-oleamide** at a concentration of 5 μM (2% DMSO in EtOH) with Rose Bengal (5 μM, 2% DMSO in EtOH) as the reference and 1,3-diphenylisobenzofuran (DPBF, 300 μM, 2% DMSO in EtOH) as the $^1O_2$ scavenger. Absorbance readings were taken at 410 nm and 520 nm before and after illumination of the samples (520 nm, 0.35 mW cm^{-2}) for 240, 120, 60, and 30 seconds. The change in absorbance of DPBF at 410 nm ($A_{DPBF,t}$) was plotted as a function of time and the gradient of the graph was used to calculate the rate of singlet oxygen production, k, using the equation below:

$$kt = \left(\frac{\ln A_{DPBF,t}}{\ln A_{DPBF,t=0}}\right) \ln A_{520nm,PS}$$

where $A_{DPBF,t}$ represents the absorbance of the PS at 520 nm. To obtain the SOQY, Φ_Δ relative to that of the reference, the following relationship is used:

$$\Phi_{\Delta PS} = \Phi_{\Delta ref} \times \left(\frac{k_{PS}}{k_{ref}}\right)$$

Enzymatic assays. Assays with β-galactosidase were performed in either phosphate-buffered saline (PBS) or water with additional supplementation or changes to pH as indicated. Human cathepsin B (CatB) assays were performed in acetate buffer (100 mM sodium acetate, 100 mM sodium chloride, 10 mM DTT, 1 mM EDTA, pH 5.5). Human granzyme A (GzmA) assays were performed in Tris buffer (0.05 M TRIS containing 0.15 M NaCl and 0.01% Triton X-100, pH 8.0). Human granzyme B (GzmB) assays were performed in Tris buffer (0.05 M Tris-HCl, 0.1 M NaCl, pH 7.4). Human neutrophil elastase (NE) assays were performed in Tris buffer (0.05 M Tris, 1 M NaCl, 0.01% m/v Triton X-100, pH 8). Assays using matrix metalloproteinases (MMP-2, MMP-9 and MMP-13) were performed in Tris buffer (0.05 M Tris, 0.2 M NaCl, 2 mM $CaCl_2$, pH 7.5). All reagents were warmed to 37 °C prior to the start of the experiments. **Gal-SeNBD-oleamide** was then incubated with β-galactosidase at the concentrations indicated in PBS unless otherwise indicated at 37 °C. Fluorescence readings (λ_{ex} = 520 nm, λ_{em} = 590 nm) were simultaneously taken every 1 min during the incubation period using a Biotek Synergy Cytation 3 spectrophotometer.

ROS generation post-enzymatic cleavage. Gal-SeNBD-oleamide (10 μM) was incubated with or without β-galactosidase (100 nM) using 20% captisol in water as the enzyme buffer at 37 °C for 2 h. ROS measurements were performed in solvent-resistant 96-wells in triplicate. DPBF (300 μM) was then added and absorbance at 410 nm was measured before and after illumination (520 nm, 3 min, 10 mW cm^{-2}). The decrease in absorbance was calculated and corrected for any decrease observed in associated controls (blank: 20% captisol in water) using the following formula:

$$DPBF\ decrease\ (\%) = \left[\left(\frac{A_S - A_{S0}}{A_{S0}}\right) - \left(\frac{A_B - A_{B0}}{A_{B0}}\right)\right] \times\ 100\ \%$$

Where A_{S0} and A_S represent the absorbance of the sample at 410 nm before and after illumination respectively, while A_{B0} and A_B represent the absorbance of the blank at 410 nm before and after illumination respectively.

Cell culture. Patient-derived glioma stem cell lines were kindly provided by the University of Edinburgh Glioma Cellular Genetics Resource. Cells were grown in DMEM/HAMS-F12 supplemented with glucose, MEM NEAA, Pen- Strep, BSA, 0.1 mM β-mercaptoethanol, B27 Supplement (1x), N_2 Supplement (1x), 10 ng mL^{-1} murine EGF (peprotech 315-09), 10 ng mL^{-1} human FGF, and 2 μg mL^{-1} laminin. All cells were grown at 37 °C and 5% CO_2 and passaged at 70-80 % confluency. THP1 monocytes were kindly provided by the Anderson lab at the University of Edinburgh. Cells were grown in RPMI supplemented with 10% fetal bovine serum (FBS), Pen-strep (100 U mL^{-1}) and L-glutamine (2 mM). All cells were grown at 37 °C and 5% CO_2 and passaged at 70-80 % confluency. In order to differentiate the THP1 to macrophages, 5×10^5 cells were plated in 6-well plates and cultured with 10 ng mL^{-1} (PMA) for 72 hours followed by washing and 48 h of resting phase. The cells were then detached with accutase (15 min at 37 °C) and treated with the indicated probe for flow cytometry analysis.

Confocal microscopy. E17 cells (50,000 cells per well) were plated into 18-well glass bottom μ-slides (Ibidi) and incubated at 37 °C and 5% CO_2. Cells were then treated with the indicated compounds and imaged in phenol red-free DMEM at 37 °C and 5% CO_2. Confocal microscopy images were acquired on a Leica Stellaris FALCON microscope (λ_{ex} = 520 nm, λ_{em} = 620±50 nm, 63x oil objective) and analyzed using LAS X software. For 2',7'-dichlorodihydrofluorescein diacetate (DCFH-DA) assays, cells were treated with the indicated compounds with or without light irradiation, followed by DCFH-DA treatment (10 μM, 30 min at 37 °C and 5% CO_2). Images were then acquired on a Leica Stellaris FALCON microscope (λ_{ex} = 490 nm, λ_{em} = 525±25 nm, 63x oil objective) and analyzed using LAS X software.

Phototoxicity assays. E17 cells (10,000 cells per well) were plated into 96-well plates and incubated at 37 °C and 5% CO_2 for 24 h. Cells were then incubated with their respective compounds at the indicated concentrations in phenol red-free DMEM at 37 °C and 5% CO_2 for 1 h. Next, cells were either irradiated (15 mW cm^{-2}) or kept in the dark for another 1 h before the cell media was replaced with fresh supplemented DMEM/HAMS-F12 media. 0.5x

resazurin solution was added to the cells 2 h post-treatment and incubated at 37 °C and 5% CO_2 for 12 h. Fluorescence values were then taken at 590 nm (λ_{ex} = 560 nm) and analyzed. Cell viability and phototoxicity values were normalized against non-treated cells and cells incubated with 30% DMSO (dead) and vice-versa using the following equations:

$$Cell\ viability\ (\%) = \frac{Fl_{PDT-treated} - Fl_{dead}}{Fl_{non-treated} - Fl_{dead}} \times 100\%$$

$$Phototoxicity\ (\%\ cell\ deaths) = 100\% - Cell\ viability\ (\%)$$

where $Fl_{PDT\text{-}treated}$ represents the average fluorescence intensity from the PDT-treated cells, Fl_{dead} represents the average fluorescence intensity from the dead control, and $Fl_{non\text{-}treated}$ represents the average fluorescence intensity of the non-treated control.

Isolation of human primary immune cells and flow cytometry. Whole blood was collected from human healthy volunteers following informed written consent with ethical approval (25-EMREC-023) at the University of Edinburgh. Granulocytes and non-granulocytes were isolated from the peripheral blood by dextran sedimentation and Percoll gradient separation.[1] Isolated immune cells, along with E17 cells (250,000 cells per condition) were incubated with **Gal-SeNBD-oleamide** (10 μM) for 1 h at r.t., then with Zombie Violet Live/Dead stain for 20 min at 4 °C followed by washes with PBS. The cells were resuspended in FACS buffer (PBS + 0.1% BSA) and taken to the flow cytometer (Novocyte), gated according to their distinct size and shape and histograms were plotted using FCS express 7 (De Novo Software). For cytotoxicity assays, immune cells were treated with indicated compounds followed by light irradiation (15 mW cm^{-2}, 60 min). Cells were then washed and stained with Zombie Violet Live/Dead stain for 20 min at 4 °C before performing flow cytometry experiments (Novocyte). Data were analyzed using FCS express 7 (De Novo Software).

Optical system design. Traditional clinical PDT light sources include bulk systems (e.g., dye lasers, metal vapor lasers) and more recently diode lasers at high costs for a single wavelength unit. In contrast, we built a component-based system (for versatility), combining

fluorescence spectroscopy (to monitor PS release) and PDT dosimetry (to activate the PS). We implemented optical fibre coupling between the light source (laser diode) and illumination head to ensure freedom of movement in a crowded clinical environment. Separation of source, delivery and spectroscopy units enabled component interchangeability. The illumination and collection head had adjustable optical tuning for alignment to macroscopic samples (e.g., tissue slices). As such, in combination with bespoke visualization software, we performed monitoring of biomarker-triggered PS activation. Furthermore, in order to potentially employ the device with multiple PS, we employed low-cost laser diode components that could be easily substituted to match the spectral profiles of different PS.

The component laser diodes (~5.6 mm in diameter) were housed in standard TO-can packaging to deliver power above 1 W. The device was designed to operate with a moderate current supply (~1 A) and included adequate mounting for efficient heat dissipation through a thermoelectric cooler. The device emitted multimode light, which was captured using standard multimode optical fibres with a simple lensing arrangement, resulting in only moderate power loss (approximately 30% depending on diode). The flexibility of the component-based system enabled co-aligned light collection for monitoring and dosimetry, all powered by the same light source providing the PDT fluence.

To match the excitation peak of our PS, a 520 nm laser diode was chosen with an available power of 50 mW, coupled to a multimode optical fibre (400 μm diameter) via a pair of aspheric lenses with 70% efficiency. Sample illumination and PS emission collection was achieved through reimaging both fibre facets onto the sample with 30 mm focal length achromatic doublet lenses. Light illumination was matched to 7 mm as the well size of a 96-well plate. For fluorescence measurements, interchangeable filters were used to allow adaption to different PS. The illumination path included a band-pass filter to remove and unwanted long wavelength emission from the diode. The fluorescence signals of the PS were collected via a separate optical path to prevent detection of any background fibre fluorescence, filtered to remove

unwanted illumination laser signal, such that only fluorescence was monitored and recorded by a spectrometer. An electrically actuated shutter was placed after the fibre output before the laser clean up filter. This shutter enabled stable continuous laser operation. During experiments the shutter was remotely opened to allow controllable illumination of the sample and closed to prevent photobleaching or unintended prolonged sample exposure. A graphical user interface (GUI) was developed for real time monitoring of PS activation. This stand-alone application (Matlab) displays data from the spectrometer automatically acquired when the shutter is activated. The application runs in parallel with the spectrometer software to display fluorescence spectra and PS activation.

Mouse brain tissue experiments. All animal procedures were approved by the University of Edinburgh Animal Welfare and Ethical Review Body (AWERB) and conducted under UK Home Office licence [PPL number: PP8631583], in accordance with the Animals (Scientific Procedures) Act 1986 and ARRIVE guidelines. Mice were housed in a pathogen-free facility with standard husbandry. Tumor development was confirmed by IVIS™ bioluminescence imaging of live mice. Young adult mouse brains (5–6 weeks old) were then removed, sliced (300 μm thickness) and cultured on tissue membrane in complete culture media for a maximum of 7 days. On the days of experiments, slices were transferred to a black 96-well plate and incubated with or without **Gal-SeNBD-oleamide** (20 μM) at 37 °C and in the dark (200 μL per well). Fluorescence emission was recorded at every hour using our illuminator. After 4 hours, slices were illuminated at 520 nm for 60 min (25 mW cm^{-2}). Tissue slices were then stained with culture media containing Hoechst (1:1,000) and NucView Red (1:200) for 3 h at 37 °C, followed by fixation with 4% PFA for 2 h at r.t and washes with PBS. On the following day, slices were treated with Rapiclear and imaged using an Opera Phenix microscope (40× lens).

Human brain tissue experiments. Human brain tissue slices were obtained following all relevant ethical regulations. Material provision and use was covered by Lothian NRS

BioResource RTB approval (REC reference: 20/ES/0061), and biosamples were collected as per existing approval (reference: SR826/SR1319; Paul Brennan). Slices of 300 μm thickness were cultured in tissue membranes and in E17 complete growth media. On the days of experiments, slices were transferred to a black 96-well plate and incubated with or without **Gal-SeNBD-oleamide** (20 μM) at 37 °C and in the dark (200 μL per well). Fluorescence emission was recorded at every hour using our illuminator. After 4 hours, slices were illuminated at 520 nm for 60 min (25 mW cm^{-2}). Tissue slices were then stained with culture media containing Hoechst (1:1,000) and NucView Green (1:200) for 3 h at 37 °C, followed by fixation with 4% PFA for 2 h at r.t and washes with PBS. On the following day, slices were treated with Rapiclear and imaged using an Opera Phenix microscope (40×).

Chemical Synthesis

The synthesis and characterization of compounds **1**, **8**, **9**, **10**, **12**, **21**, **22** and **SeNBD-F** have been previously described.[2,3]

2-((7-nitrobenzo[c][1,2,5]selenadiazol-4-yl)amino)ethyl)triphenylphosphonium formate (2)

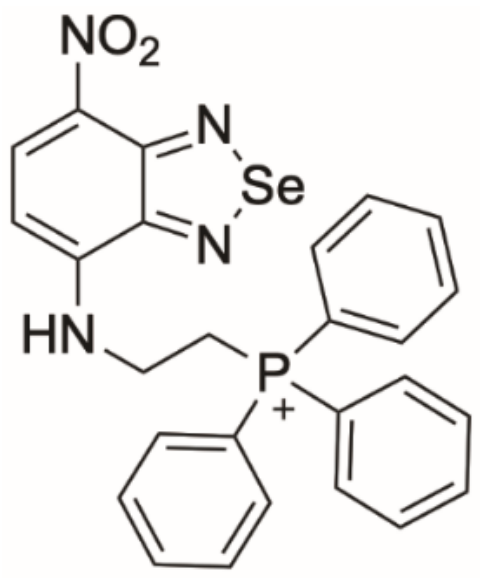

SeNBD-F (20.7 mg, 0.08 mmol) was dissolved in MeCN (3 mL). (2-aminoethyl)triphenylphosphonium bromide (38.3 mg, 0.09 mmol) and triethylamine (34 µL, 0.24 mmol) were added and the reaction was stirred for 24 h at r.t. The reaction mixture was concentrated *in vacuo* and the product was purified by semi-preparative HPLC (MeCN:H_2O 5:95 to 95:5 (+0.1% HCOOH)) to give compound **2** as an orange salt (12 mg, 28%).

^{1}H NMR (500 MHz, D_2O) δ 8.46 (d, *J* = 8.4 Hz, 1H), 8.38 (d, *J* = 0.7 Hz, 1H), 7.72 – 7.65 (m, 6H), 7.64 – 7.58 (m, 3H), 7.47 (td, *J* = 7.9, 3.4 Hz, 6H), 6.13 (d, *J* = 8.7 Hz, 1H), 4.13 (d, *J* = 18.7 Hz, 2H), 3.85 (dt, *J* = 13.0, 6.6 Hz, 2H).

^{13}C NMR (126 MHz, MeOD) δ 152.0, 152.0, 146.5, 134.9, 134.9, 134.4, 133.5, 133.4, 130.2, 130.1, 129.9, 118.3, 117.6, 97.7, 36.3, 21.8, 21.4.

HRMS (ESI+): m/z calculated for $C_{26}H_{22}N_4O_2PSe$: 533.0640, found: 533.0628 $[M]^+$.

(9Z,12Z)-*N*-(2-((7-nitrobenzo[c][1,2,5]selenadiazol-4-yl)amino)ethyl)octadeca-9,12-dienamide (3)

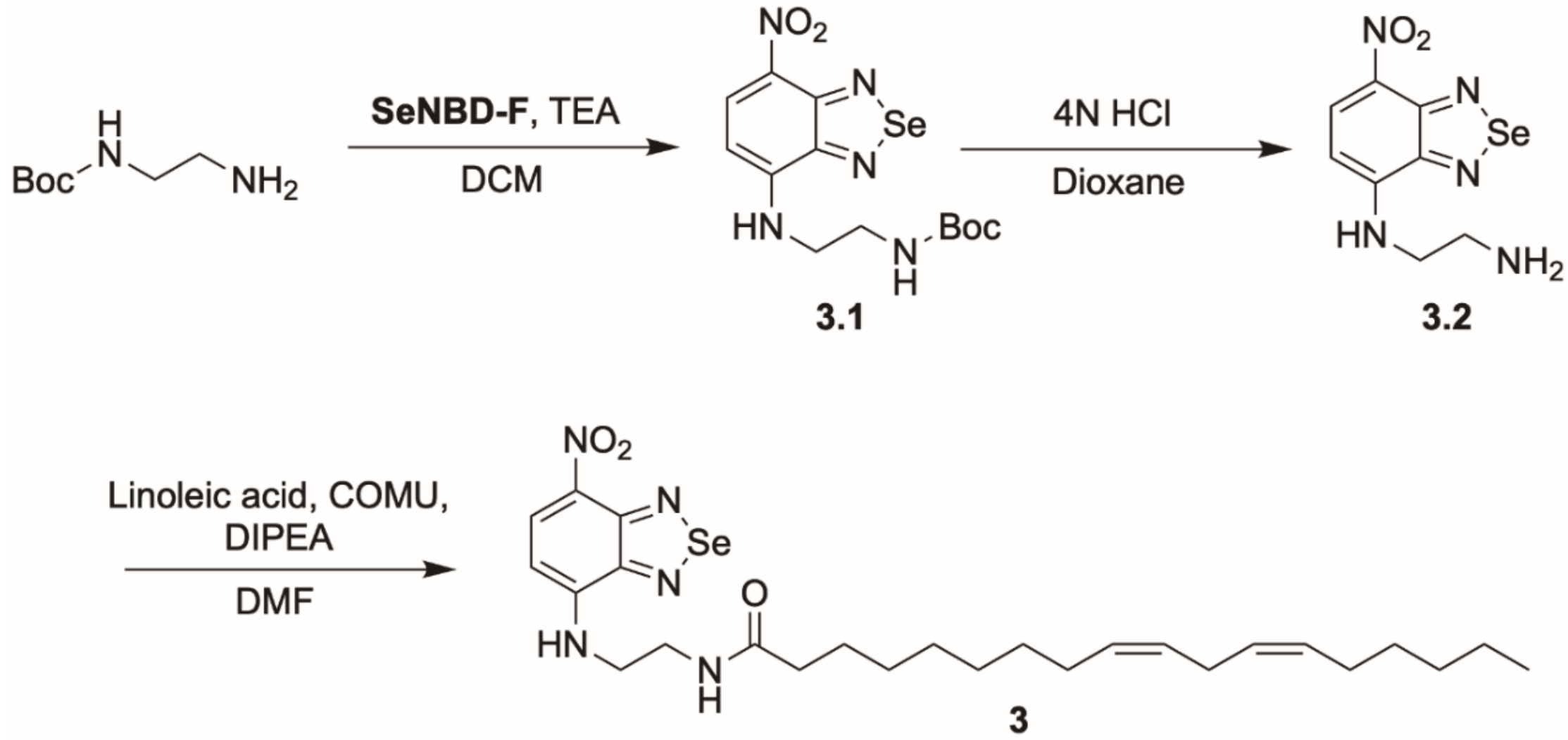

Compound 3.1. To a solution of **SeNBD-F** (1.0 g, 4.1 mmol) and N-Boc-ethylenediamine (1.3 g, 8.1 mmol) in CH_2Cl_2 (30 mL), triethylamine (1.1 mL, 8.1 mmol) was added and stirred at 50 °C for 2 h. The reaction was monitored using HPLC-MS and TLC. The reaction mixture was concentrated *in vacuo* and purified using silica gel chromatography (50% EtOAc in CH_2Cl_2). Compound **3.1** was obtained as a bright orange powder (1.3 g, 82%).

^{1}H NMR (500 MHz, DMSO-d_6) δ 8.57 (d, *J* = 8.9 Hz, 1H), 8.37 (t, *J* = 6.3 Hz, 1H), 7.03 (t, *J* = 5.8 Hz, 1H), 6.41 (d, *J* = 9.0 Hz, 1H), 3.51 (q, *J* = 6.3 Hz, 2H), 3.24 (q, *J* = 6.1 Hz, 2H), 1.34 (s, 9H).

^{13}C NMR (126 MHz, DMSO-d_6) δ 155.8, 151.9, 151.9, 148.6, 135.1, 128.1, 97.1, 77.9, 42.8, 28.1.

MS (ESI+): m/z calculated for $C_{13}H_{17}N_5O4Se$: 388.1, found: 388.1 $[M+H]^+$.

Compound 3.2. Compound **3.1** (73 mg, 0.19 mmol) was dissolved in a solution of HCl (4 N, dioxane, 4 mL). Dioxane (1 mL) was used to dissolve any remaining starting material, and the reaction was stirred at r.t. for 2.5 h. The reaction mixture was concentrated *in vacuo* and the residue diluted in water before lyophilization to yield compound **3.2** as an orange powder (48 mg, 88%), which was used without purification.

Compound 3. Compound **3.2** (20 mg, 0.07 mmol), linoleic acid (19.7 mg, 0.07 mmol) and COMU (60 mg, 0.14 mmol) were dissolved in CH_2Cl_2 (2 mL). DIPEA (240 μL, 1.40 mmol) was added incrementally and the reaction was stirred under mild heating for 4 h. The reaction was concentrated *in vacuo* and the crude product purified by silica gel chromatography (2% MeOH in CH_2Cl_2) to afford compound **3** as an orange solid (22 mg, 58%).

^{1}H NMR (500 MHz, $CDCl_3$) δ 8.63 (d, *J* 8.7 Hz, 1H), 7.57 (t, *J* 4.9 Hz, 1H), 6.19 (d, *J* 8.8 Hz, 1H), 6.07 (t, *J* 6.2 Hz, 1H) 5.48 – 5.26 (m, 4H), 3.74 (q, *J* 5.9 Hz, 2H), 3.59 (q, *J* 5.3 Hz, 2H), 2.84 – 2.69 (m, 2H), 2.34 – 2.20 (m, 2H), 2.11 – 1.94 (m, 4H), 1.74 – 1.50 (m, 2H), 1.42 – 1.18 (m, 14H), 0.96 – 0.80 (m, 3H).

^{13}C NMR (126 MHz, $CDCl_3$) δ 175.4, 152.5, 147.6, 135.3, 130.2, 130.1, 129.9, 128.1, 127.8, 97.0, 45.1, 38.5, 36.6, 31.5, 29.5, 29.3, 29.2, 29.1, 27.2, 27.1, 25.6, 25.6, 22.5, 14.0.

HRMS (ESI+): m/z calculated for $C_{26}H_{40}N_5O_3Se$: 550.2291, found: 550.2307 $[M+H]^+$.

***N*-(2-((7-nitrobenzo[c][1,2,5]selenadiazol-4-yl)amino)ethyl)oleamide (SeNBD-oleamide, 4)**

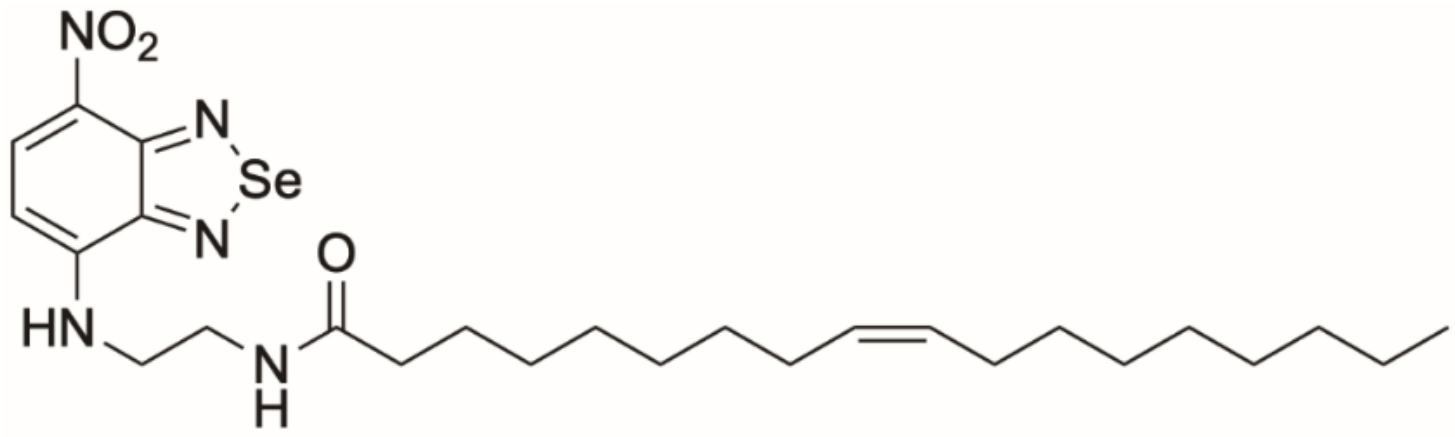

To a solution of oleic acid (0.92 g, 3.2 mmol) in DMF (5 mL), DIPEA (565 µL, 3.2 mmol) was added and stirred for 5 min. Hexafluorophosphate azabenzotriazole tetramethyl uranium (HATU, 1.48 g, 3.9 mmol) was then added to the reaction mixture and stirred for another 10 min. In a separate flask, DIPEA (565 µL, 3.2 mmol) was added to a solution of compound **3.2** (0.77 g, 2.7 mmol) dissolved in DMF (50 mL). The two mixtures were combined and stirred at 50 °C for 30 min. The reaction mixture was concentrated *in vacuo* and purified using flash column chromatography (30-100% EtOAc in hexane). Compound **4** (hereinafter **SeNBD-oleamide**) was obtained as a bright orange powder (1.4 g, 79%).

^{1}H NMR (500 MHz, DMSO-d_6) δ 8.57 (d, *J* = 9.0 Hz, 1H), 8.37 (t, *J* = 6.0 Hz, 1H), 8.04 (t, *J* = 5.8 Hz, 1H), 6.41 (d, *J* = 9.0 Hz, 1H), 5.34 – 5.23 (m, 2H), 3.50 (q, *J* = 6.1 Hz, 2H), 3.37 (q, *J* = 6.1 Hz, 2H), 2.04 (t, *J* = 7.4 Hz, 2H), 1.98 – 1.89 (m, 4H), 1.44 (p, *J* = 7.4 Hz, 2H), 1.31 – 1.10 (m, 20H), 0.83 (t, *J* = 6.9 Hz, 3H).

^{13}C NMR (126 MHz, DMSO-d_6) δ 172.9, 151.9, 151.8, 148.6, 135.1, 129.5, 129.5, 128.2, 97.2, 43.0, 37.4, 35.3, 31.2, 29.0, 29.0, 28.7, 28.6, 28.6, 28.5, 28.4, 26.5, 26.5, 25.1, 22.0, 13.9.

HRMS (ESI+): m/z calculated for $C_{26}H_{42}N_5O_3Se$: 552.2447, found: 552.2427 $[M+H]^+$.

***N*-(2-((7-nitrobenzo[c][1,2,5]selenadiazol-4-yl)amino)ethyl)dodecanamide (5)**

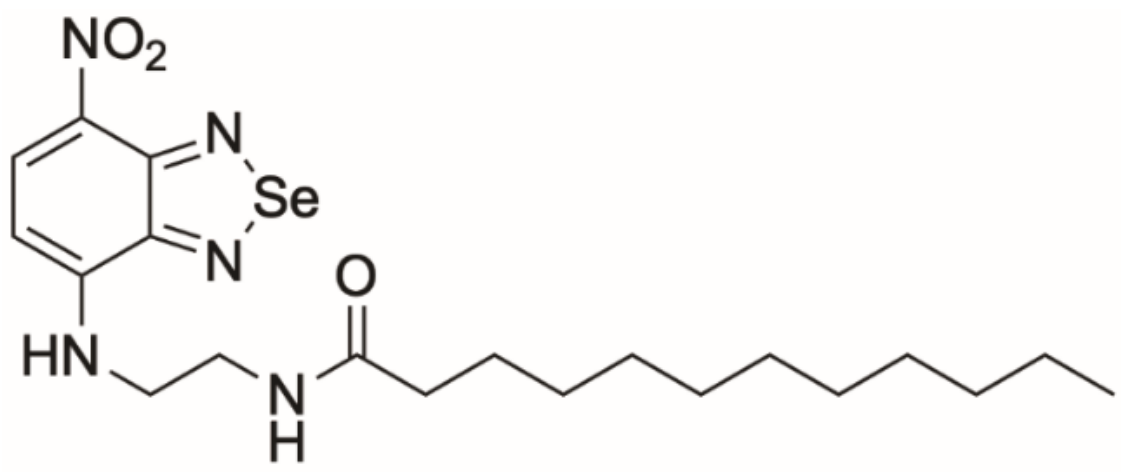

Compound **3.2** (18.4 mg, 0.06 mmol) and lauric acid (13.6 mg, 0.07 mmol) were dissolved in CH_2Cl_2 (3 mL) with DMF (0.5 mL). DIC (65 µL, 0.42 mmol) and DIPEA (73 µL, 0.42 mmol) were added and the reaction stirred at 50 °C for 96 h. The reaction mixture was concentrated *in vacuo* and the crude product was purified by flash column chromatography (EtOAc) to give compound **5** as an orange solid (13 mg, 46%).

^{1}H NMR (500 MHz, DMSO-d_6) δ 8.58 (dd, *J* = 8.9, 0.5 Hz, 1H), 8.38 (t, *J* = 6.0 Hz, 1H), 8.05 (t, *J* = 5.8 Hz, 1H), 6.42 (d, *J* = 9.0 Hz, 1H), 3.51 (q, *J* = 6.1 Hz, 2H), 3.38 (q, *J* = 6.0 Hz, 2H), 2.04 (t, *J* = 7.3 Hz, 2H), 1.44 (p, *J* = 6.9 Hz, 2H), 1.25 (d, *J* = 7.9 Hz, 16H), 0.85 (t, *J* = 7.0 Hz, 3H).

^{13}C NMR (126 MHz, DMSO-d_6) δ 173.5, 152.4, 152.2, 149.1, 135.6, 128.7, 97.6, 43.5, 41.1, 37.9, 35.8, 31.7, 29.4, 29.3, 29.1, 29.0, 25.6, 23.7, 22.5, 14.4.

HRMS (ESI+): m/z calculated for $C_{20}H_{32}N_5O_3Se$: 470.1665, found: 470.1656 $[M+H]^+$.

***N*-(2-((7-nitrobenzo[c][1,2,5]selenadiazol-4-yl)amino)ethyl)butyramide (6)**

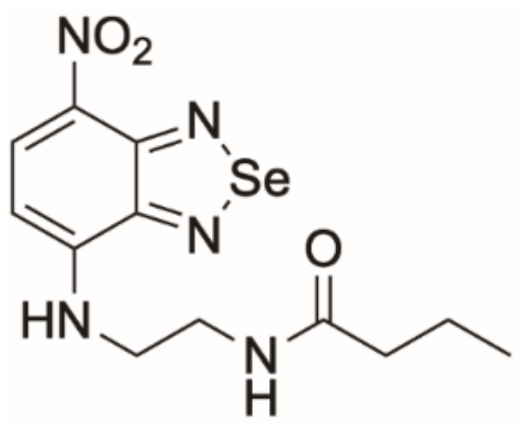

Compound **3.2** (17.1 mg, 0.06 mmol) and butyric acid (6 μL, 0.07 mmol) were dissolved in CH_2Cl_2 (3 mL) with added DMF (0.5 mL). DIC (65 μL, 0.42 mmol) and DIPEA (73 μL, 0.42 mmol) were added and the reaction was stirred at 50 °C for 96 h. The reaction mixture was concentrated *in vacuo* and the crude product purified by flash column chromatography (100% EtOAc). The organic product was washed with water (3 × 20 mL) and concentrated *in vacuo* to afford compound **6** as an orange solid (9 mg, 42%).

^{1}H NMR (500 MHz, DMSO-d_6) δ 8.59 (d, *J* = 8.9, 0.5 Hz, 1H), 8.42 (t, *J* = 6.1 Hz, 1H), 8.05 (t, *J* = 5.8 Hz, 1H), 6.43 (d, *J* = 9.0 Hz, 1H), 3.52 (q, *J* = 6.2 Hz, 2H), 3.41 – 3.36 (m, 2H), 2.04 (t, *J* = 7.3 Hz, 2H), 1.50 (h, *J* = 7.4 Hz, 2H), 0.81 (t, *J* = 7.4 Hz, 3H).

13C NMR (126 MHz, DMSO-d_6) δ 173.3, 152.4, 152.3, 149.0, 135.6, 128.6, 97.7, 43.4, 37.8, 37.7, 18.9, 14.0.

HRMS (ESI+): m/z calculated for $C_{12}H_{16}N_5O_3Se$: 358.0413, found: 358.0418 $[M+H]^+$.

7-nitrobenzo[*c*][1,2,5]selenadiazol-4-amine (7)

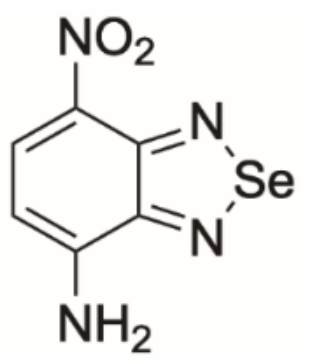

SeNBD-F (50 mg, 0.20 mmol) was dissolved in a 4N NH_3 solution in MeOH (3 mL) at r.t. for 6 h. The final product was purified by flash column chromatography (1% MeOH in CH_2Cl_2) to afford compound **7** as an orange solid (40 mg, 82%).

^{1}H NMR (500 MHz, DMSO-d_6) δ 8.55 (d, *J* = 8.8 Hz, 1H), 8.09 (br, s, 2H), 6.44 (d, *J* = 8.8 Hz, 1H).

^{13}C NMR (126 MHz, DMSO-d_6) δ 152.9, 152.1, 150.9, 135.7, 128.3, 101.2.

HRMS (ESI+): m/z calculated for $C_6H_5N_4O_2Se$: 244.9572, found: 244.9579 $[M+H]^+$.

***N*-cyclohexyl-*N*-ethyl-7-nitrobenzo[*c*][1,2,5]selenadiazol-4-amine (11)**

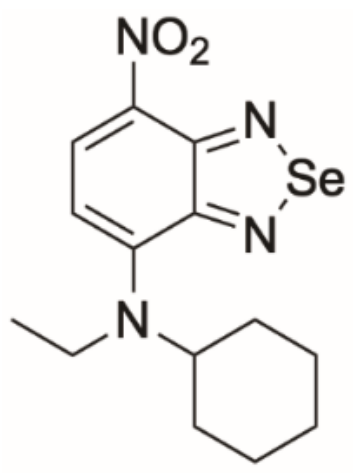

SeNBD-F (50 mg, 0.2 mmol), N-cyclohexylethylamine (60 μL, 0.4 mmol) and Et_3N (57 μL, 0.4 mmol) were combined in MeCN (2 mL) at r.t. and stirred for 1 h. The final product was purified by flash column chromatography (0-1% MeOH in CH_2Cl_2) to give compound **11** as an orange solid (56 mg, 79%).

^{1}H NMR (500 MHz, $CDCl_3$) δ 8.61 (d, *J* = 9.3 Hz, 1H), 6.34 (d, *J* = 9.3 Hz, 1H), 5.27 – 5.12 (m, 1H), 3.86 (q, *J* = 7.0 Hz, 2H), 2.09 – 1.98 (m, 2H), 1.97 – 1.88 (m, 2H), 1.83 – 1.73 (m, 1H), 1.72 – 1.60 (m, 2H), 1.55 – 1.43 (m, 2H), 1.36 (t, *J* = 7.0 Hz, 3H), 1.27 – 1.17 (m, 1H).

^{13}C NMR (126 MHz, $CDCl_3$) δ 154.5, 153.3, 149.0, 133.9, 129.3, 102.5, 61.1, 41.4, 31.2, 25.8, 25.5, 14.6.

HRMS (ESI+) m/z calculated for $C_{14}H_{19}N_4O_2Se$: 355.0668, found: 355.0668 $[M+H]^+$.

4-((7-nitrobenzo[c][1,2,5]selenadiazol-4-yl)amino)butanoic acid (13)

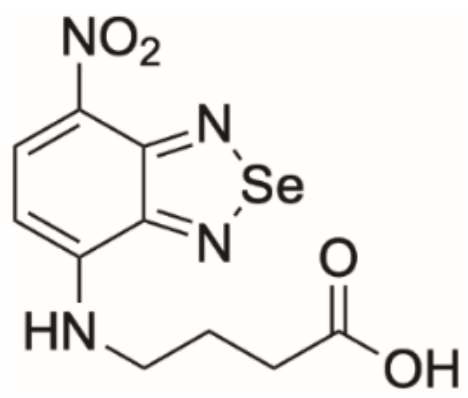

SeNBD-F (24 mg, 0.09 mmol, 1 eq), γ-aminobutyric acid (12 mg, 0.11 mmol, 1.2 eq) and triethylamine (20 μL, 0.14 mmol, 1.4 eq) were combined in MeCN:H_2O (3:1, 4 mL) at r.t.and stirred for 16 h. The reaction mixture was washed with EtOAc (20 mL) and $NaHCO_3$ (20 mL). The aqueous layer was acidified with 1 N HCl (aq.) and the product extracted into EtOAc (3 × 10 mL). The final product was purified by flash column chromatography (5-10% MeOH in CH_2Cl_2 + 0.1% AcOH) to give compound **13** as a red solid (15 mg, 51%).

^{1}H NMR (500 MHz, DMSO-d_6) δ 8.60 (d, *J* = 9.0 Hz, 1H), 6.40 (d, *J* = 9.0 Hz, 1H), 3.48 (q, *J* = 6.5 Hz, 2H), 2.35 (t, *J* = 7.2 Hz, 2H), 1.96 – 1.81 (m, 2H).

^{13}C NMR (126 MHz, DMSO-d_6) δ 174.8, 152.5, 152.4, 149.0, 135.7, 128.5, 97.7, 42.7, 31.6, 23.8.

HRMS (ESI+): m/z calculated for $C_{10}H_{11}N_4O_4Se$: 330.9940, found: 330.9943 $[M+H]^+$.

2-hydroxy-*N,N*-dimethyl-*N*-(2-((7-nitrobenzo[*c*][1,2,5]selenadiazol-4-yl)amino)ethyl)ethan-1-aminium (14)

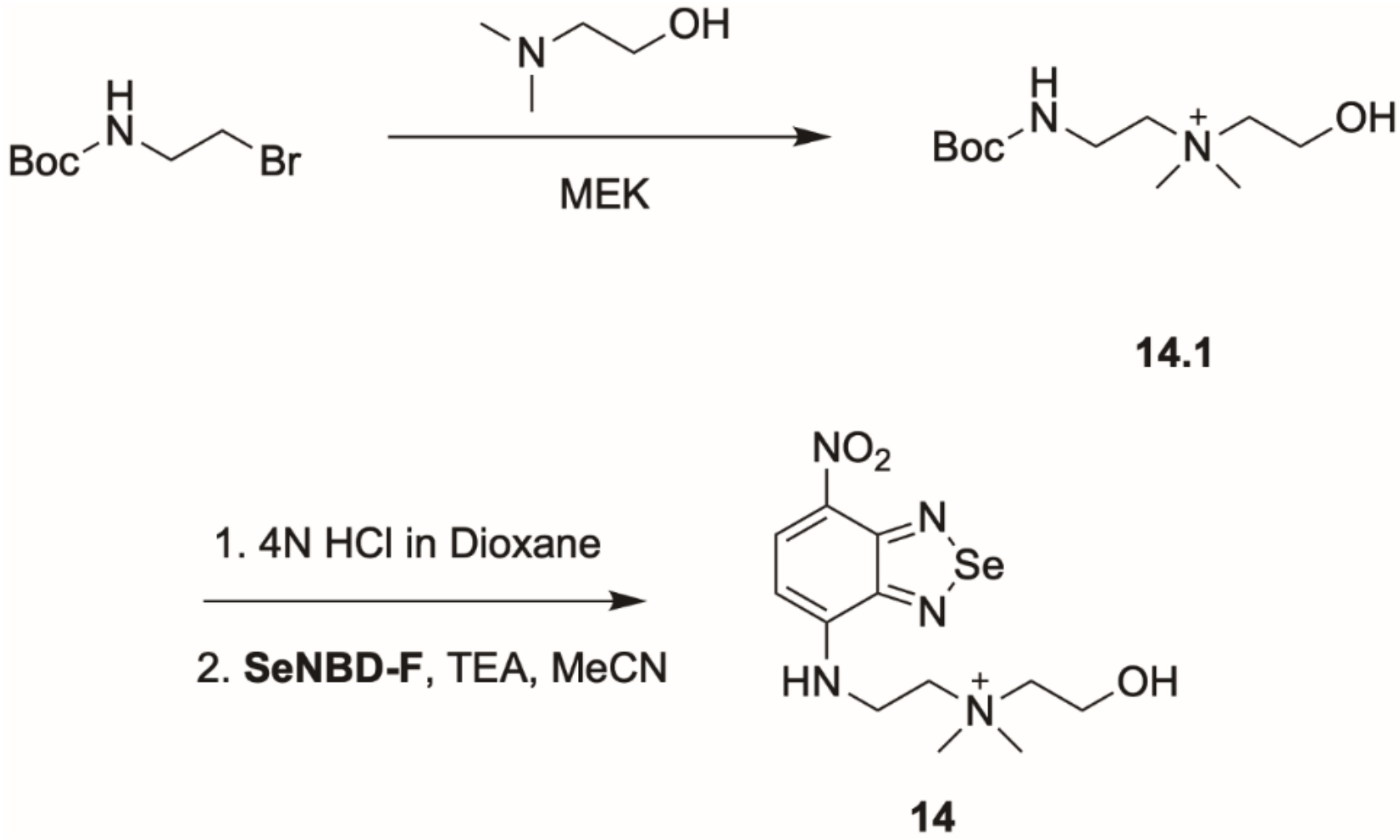

Compound 14.1. Boc-aminoethylbromide (25.1 mg, 0.11 mmol) and 2-dimethylaminoethanol (16 µL, 0.11 mmol) were dissolved in methyl ethyl ketone (0.5 mL) and stirred at 40 °C for 16 h. Additional 2-dimethylaminoethanol (8 µL, 0.06 mmol) was added and the reaction left to stir at 40 °C for an additional 24 h. After the reaction had plateaued, the mixture was allowed to cool to r.t. before precipitating the product in ice-cold Et_2O (50 mL). The precipitate was centrifuged to allow supernatant removal, and the resultant colourless oil was dried under high vacuum to afford compound **14.1** (17 mg, 66%).

^{1}H NMR (500 MHz, D_2O) δ 4.06 – 3.98 (m, 2H), 3.58 – 3.44 (m, 6H), 3.16 (s, 6H), 2.84 (d, *J* = 0.8 Hz, 1H), 1.39 (s, 9H).

^{13}C NMR (126 MHz, D_2O) δ 157.7, 81.7, 65.7, 63.3, 58.8, 55.3, 51.8, 42.8, 34.3, 27.6.

HRMS (ESI+): m/z calculated for $C_{11}H_{25}N_2O_3$: 233.1860, found: 233.1864 $[M]^+$.

Compound 14. The compound **14.1** (17.3 mg, 0.07 mmol) was dissolved in a 4 N HCl solution (dioxane, 3 mL) and stirred at r.t. for 20 min. The reaction was quenched with water (20 mL) and excess dioxane removed *in vacuo*. The aqueous layer was washed with CH_2Cl_2 (2 × 20 mL) before lyophilization to give a crude intermediate which was used without further purification. The crude intermediate (11.9 mg, 0.09 mmol) and **SeNBD-F** (21.7 mg, 0.08 mmol) were dissolved in MeCN (3 mL). Triethylamine (12.4 µL, 0.08 mmol) was added and the

reaction stirred at r.t. for 48 h. The reaction was adjusted to pH 9 by addition of saturated aqueous $NaHCO_3$ (1 mL) and stirred at r.t. overnight. The mixture was concentrated *in vacuo* and the product was redissolved in water (10 mL) before lyophilization. The crude product was washed with EtOAc. Final purification step with semi-preparative HPLC yielded compound **14** as a red solid (2.6 mg).

^{1}H NMR (500 MHz, MeOD) δ 8.68 (d, *J* = 8.7 Hz, 1H), 8.56 (s, 1H), 6.53 (d, *J* = 8.8 Hz, 1H), 4.12 – 4.05 (m, 4H), 3.86 (t, *J* = 6.8 Hz, 2H), 3.68 – 3.63 (m, 2H), 3.34 – 3.33 (m, 6H).

13C NMR (126 MHz, MeOD) δ 152.4, 152.2, 147.0, 134.6, 130.1, 97.5, 65.8, 62.1, 55.4, 51.4, 36.7.

HRMS (ESI+): calculated for $C_{12}H_{18}N_5O_3Se$: 360.0569, found: 360.0569 $[M]^+$.

2-acetoxy-*N*,*N*-dimethyl-*N*-(2-((7-nitrobenzo[*c*][1,2,5]selenadiazol-4-yl)amino)propyl)ethan-1-aminium (15)

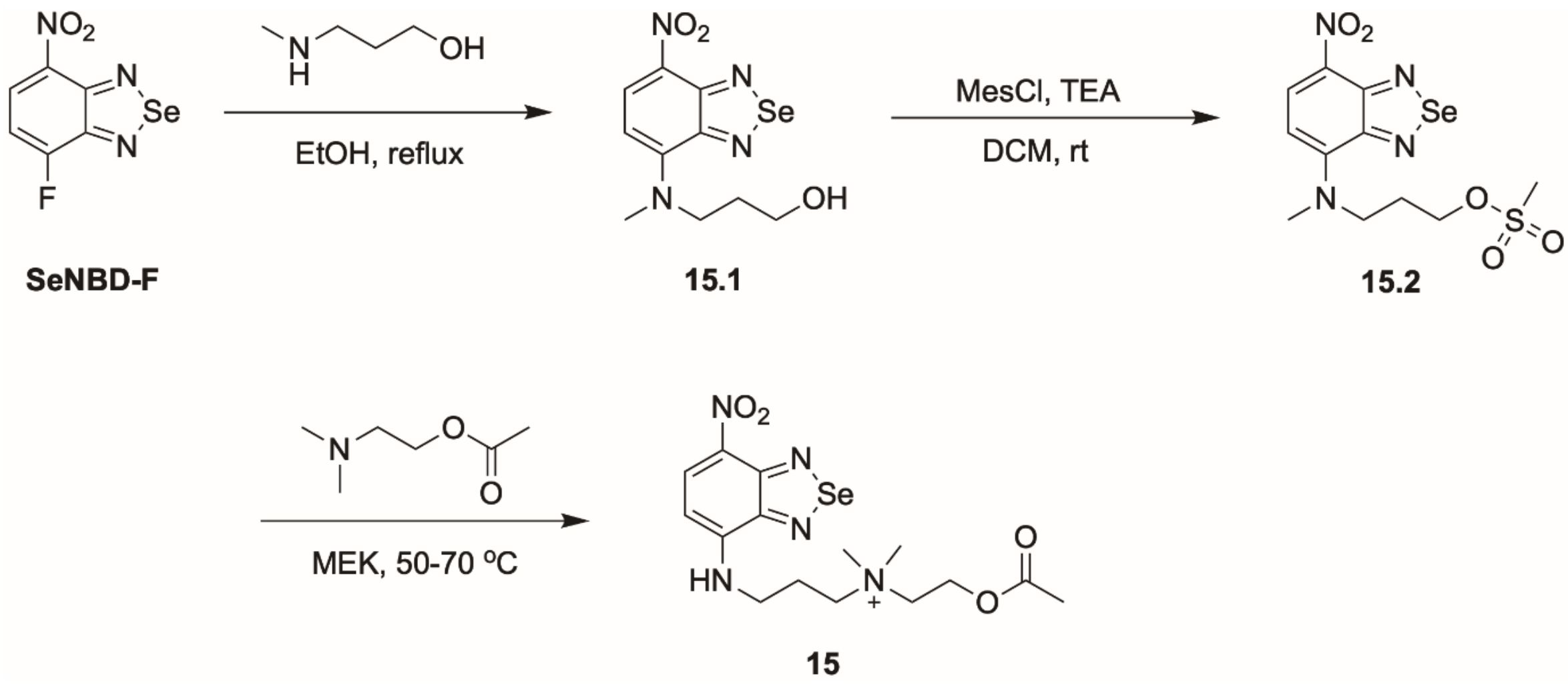

Compound 15.1. SeNBD-F (50.8 mg, 0.2 mmol) and 3-methylamino-1-propanol (20 µL, 0.2 mmol) were dissolved in EtOH (2 mL) and stirred at 55 °C for 2 h. The reaction was poured into ice-cold EtOH (10 mL) and stored at 4 °C overnight. The solid was filtered off,washed with EtOH and dried *in vacuo*. The filtrates were recrystallized in EtOH to afford compound **15.1** as a red solid (49 mg, 78%).

^{1}H NMR (500 MHz, MeOD) δ 8.62 (d, *J* = 9.3 Hz, 1H), 6.48 (d, *J* = 9.3 Hz, 1H), 4.34 – 4.29 (m, 2H), 3.73 (t, *J* = 6.1 Hz, 2H), 3.54 (s, 3H), 2.11 – 2.02 (m, 2H).

^{13}C NMR (126 MHz, MeOD) δ 153.9, 152.7 149.7, 133.9, 101.7, 58.9, 52.5, 41.1, 30.5.

HRMS (ESI+): m/z calculated for $C_{10}H_{13}N_4O_3Se$: 317.0147, found: 317.0157 $[M+H]^+$.

Compound 15.2. SeNBD-(methyl)aminopropanol (20.7 mg, 0.06 mmol) was dissolved in CH_2Cl_2 (2 mL) at r.t. Triethylamine (37 µL, 0.26 mmol) and mesyl chloride (25 µL, 0.32 mmol) were added and the reaction was stirred at r.t. for 30 min. The reaction was monitored by HPLC-MS and stopped at 85% conversion. The reaction mixture was concentrated *in vacuo*. The crude product was purified by flash column chromatography (2% MeOH in CH_2Cl_2) to give compound **15.2** as a red solid (13 mg, 55%).

^{1}H NMR (500 MHz, DMSO-d$_6$) δ 8.52 (d, *J* = 9.2 Hz, 1H), 6.46 (d, *J* = 9.3 Hz, 1H), 4.34 (t, *J* = 6.2 Hz, 2H), 4.29 (t, 2H), 3.43 (s, 3H), 3.18 (s, 3H), 2.21 – 2.10 (m, 2H).

^{13}C NMR (126 MHz, DMSO-d$_6$) δ 153.5, 152.7, 149.6, 133.7, 129.1 102.5, 68.7, 51.9, 42.1, 37.1, 27.9.

HRMS (ESI+): m/z calculated for $C_{11}H_{15}N_4O_5SSe$: 394.9923, found: 394.9927 $[M+H]^+$.

Compound 15. Compound **15.2** (12.9 mg, 0.033 mmol) was dissolved in methyl ethyl ketone (2 mL) before addition of 2-dimethylaminoethylacetate (47 μL, 0.33 mmol). The mixture was stirred at 50 °C for 24 h, then 60 °C for another 24 h. 2-dimethylaminoethylacetate (47 μL, 0.33 mmol) was added and the reaction mixture was heated to 70 °C and stirred for 72 h. Reaction monitoring by HPLC-MS showed the starting material had been consumed. The reaction mixture was concentrated *in vacuo* and purification by preparative HPLC afforded compound **15** as a red solid (6 mg, 45%).

^{1}H NMR (500 MHz, DMSO-d6) δ 8.54 (d, *J* = 9.2 Hz, 1H), 6.48 (d, *J* = 9.4 Hz, 1H), 4.44 (t, *J* = 4.9 Hz, 2H), 4.20 (t, *J* = 7.4 Hz, 2H), 3.67 – 3.61 (m, 2H), 3.55 – 3.46 (m, 2H), 3.39 (s, 3H), 3.10 (s, 6H), 2.24 – 2.15 (m, 2H), 2.05 (s, 3H).

^{13}C NMR (126 MHz, DMSO-d6) δ 170.3, 153.4, 152.6, 149.4, 133.7, 129.3, 102.5, 62.0, 61.7, 58.0, 52.1, 51.2, 41.8, 22.0, 21.1.

HRMS (ESI+): m/z calculated for $C_{16}H_{24}N_5O_4Se$: 430.0988, found: 430.0972 $[M]^+$.

N^1-(7-nitrobenzo[*c*][1,2,5]selenadiazol-4-yl)butane-1,4-diamine (16)

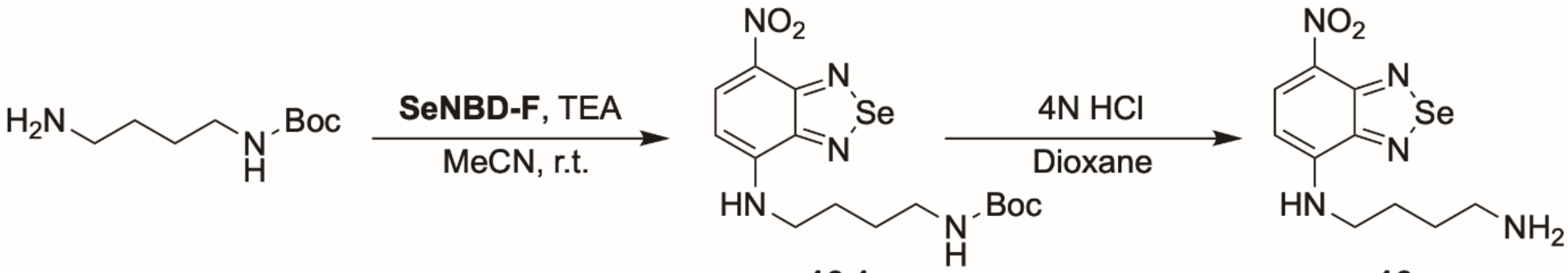

Compound 16.1. SeNBD-F (20.9 mg, 0.08 mmol) and Boc-putrescine (16.8 mg, 0.08 mmol) were combined in MeCN (2 mL) containing triethylamine (12.4 µL, 0.08 mmol). The reaction was stirred at r.t. for 2 h and concentrated *in vacuo*. The crude product was purified by column chromatography (30-60% EtOAc in hexane) to afford compound **16.1** as an orange solid (25 mg, 75%).

^{1}H NMR (500 MHz, $CDCl_3$) δ 8.73 – 8.62 (m, 1H), 6.54 (s, 1H), 6.25 (d, *J* = 8.8 Hz, 1H), 4.62 (s, 1H), 3.52 (t, *J* = 7.1 Hz, 2H), 3.24 (t, *J* = 6.9 Hz, 2H), 1.87 (tt, *J* = 7.8, 6.4 Hz, 2H), 1.74 – 1.66 (m, 2H), 1.47 (s, 9H).

^{13}C NMR (126 MHz, $CDCl_3$) δ 156.0, 152.5, 152.4, 147.2, 135.5, 130.1, 97.2, 79.5, 43.1, 39.9, 28.4, 27.8, 25.9.

HRMS (ESI+): m/z calculated for $C_{15}H_{22}N_5O_4Se$: 416.0832, found: 416.0819 $[M+H]^+$.

Compound 16. Compound **16.1** (16.8 mg, 0.04 mmol) was added to 4 N HCl in Dioxane (2 mL) for 1 h. The reaction was quenched with aqueous NaOH (2 N, 4 mL) and lyophilized prior to purification by preparative HPLC. Compound **16** was isolated as an orange solid (12 mg, 95%).

^{1}H NMR (500 MHz, MeOD) δ 8.70 (d, *J* = 8.9 Hz, 1H), 6.42 (d, *J* = 8.9 Hz, 1H), 3.61 (t, *J* = 6.7 Hz, 2H), 3.02 (t, *J* = 7.7, 7.3 Hz, 2H), 1.94 – 1.79 (m, 4H).

^{13}C NMR (126 MHz, MeOD) δ 152.5, 152.3, 148.4, 135.4, 128.8, 96.9, 42.1, 39.0, 25.1, 24.7.

HRMS (ESI+): m/z calculated for $C_{10}H_{14}N_5O_2Se$: 316.0307, found: 316.0308 $[M+H]^+$.

N^1-(3-((7-nitrobenzo[*c*][1,2,5]selenadiazol-4-yl)amino)propyl)butane-1,4-diamine (17)

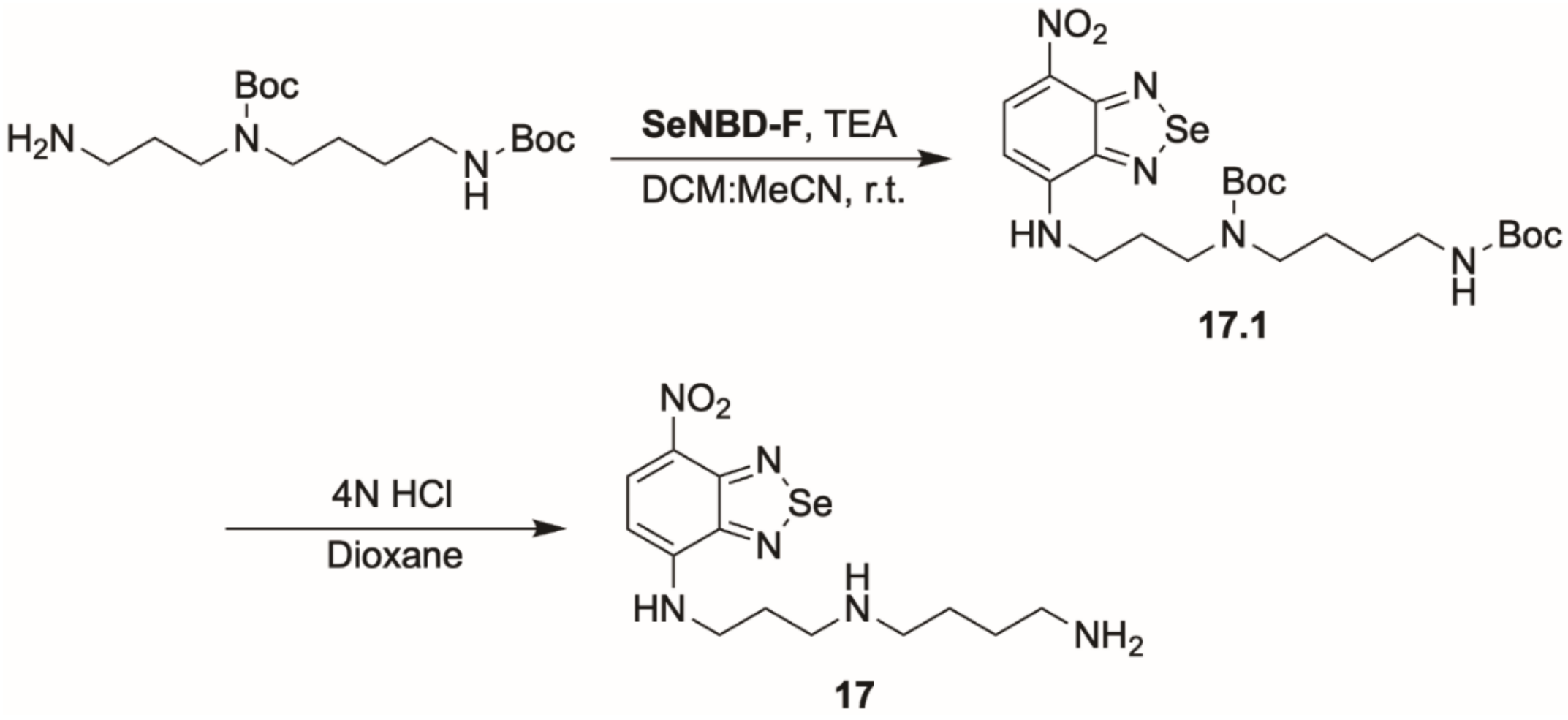

Compound 17.1. SeNBD-F (48.5 mg, 0.19 mmol) and N^1,N^5-bis-Boc-spermidine (65.5 mg, 0.19 mmol) were dissolved in a CH_2Cl_2:MeCN mixture (1:1, 4 mL) at r.t. Triethylamine (70 μL, 0.50 mmol) was added after 2 h, and the reaction was left to stir for further 14 h. The reaction mixture was concentrated *in vacuo* and the crude product was purified by silica gel column chromatography (0-80% EtOAc in CH_2Cl_2) to yield compound **17.1** as a red solid (76 mg, 70%).

^{1}H NMR (500 MHz, $CDCl_3$) δ 8.68 (d, *J* = 8.8 Hz, 1H), 7.45 (s, 1H), 6.21 (d, *J* = 8.8 Hz, 1H), 4.58 (s, 1H), 3.49 (q, *J* = 6.4 Hz, 2H), 3.39 (t, *J* = 6.4 Hz, 2H), 3.23 (t, *J* = 7.2 Hz, 2H), 3.15 (q, *J* = 6.7 Hz, 2H), 1.96 (d, *J* = 19.3 Hz, 2H), 1.63 – 1.55 (m, 2H), 1.49 (s, 9H), 1.45 (s, 9H).

^{13}C NMR (126 MHz, $CDCl_3$) δ 156.0, 152.7 152.56, 147.5, 135.5, 96.9, 80.0, 79.2, 56.7, 46.9, 43.7, 40.3, 40.1, 28.4, 28.4, 27.5, 27.2, 25.7.

Compound 17. Compound **17.1** (26.7 mg, 0.04 mmol) was dissolved in HCl (4 N in dioxane, 1.5 mL) at r.t. and stirred for 1 h. The reaction was then quenched with 2 N NaOH (aq., 3 mL) and dissolved in H_2O before lyophilization. The crude product was purified by preparative HPLC to afford compound **17** as an orange solid (6 mg, 34%).

^{1}H NMR (500 MHz, MeOD) δ 8.65 (d, *J* = 8.8 Hz, 1H), 6.41 (d, *J* = 8.9 Hz, 1H), 3.67 (t, *J* = 6.8 Hz, 2H), 3.27 – 3.17 (m, 2H), 3.17 – 3.06 (m, 2H), 3.06 – 2.92 (m, 2H), 2.20 (dt, *J* = 14.7, 7.0 Hz, 2H), 1.88 – 1.71 (m, 4H).

^{13}C NMR (126 MHz, MeOD) δ 152.3, 148.1, 135.4, 128.9, 97.1, 45.3, 44.5, 39.7, 38.6, 36.4, 24.9, 24.2, 22.9.

HRMS (ESI+): m/z calculated for $C_{13}H_{21}N_6O_2Se$: 373.0886, found: 373.0898 $[M+H]^+$.

***N*[1]-(3-aminopropyl)-*N*[4]-(3-((7-nitrobenzo[*c*][1,2,5]selenadiazol-4-yl)amino)propyl)butane-1,4-diamine (18)**

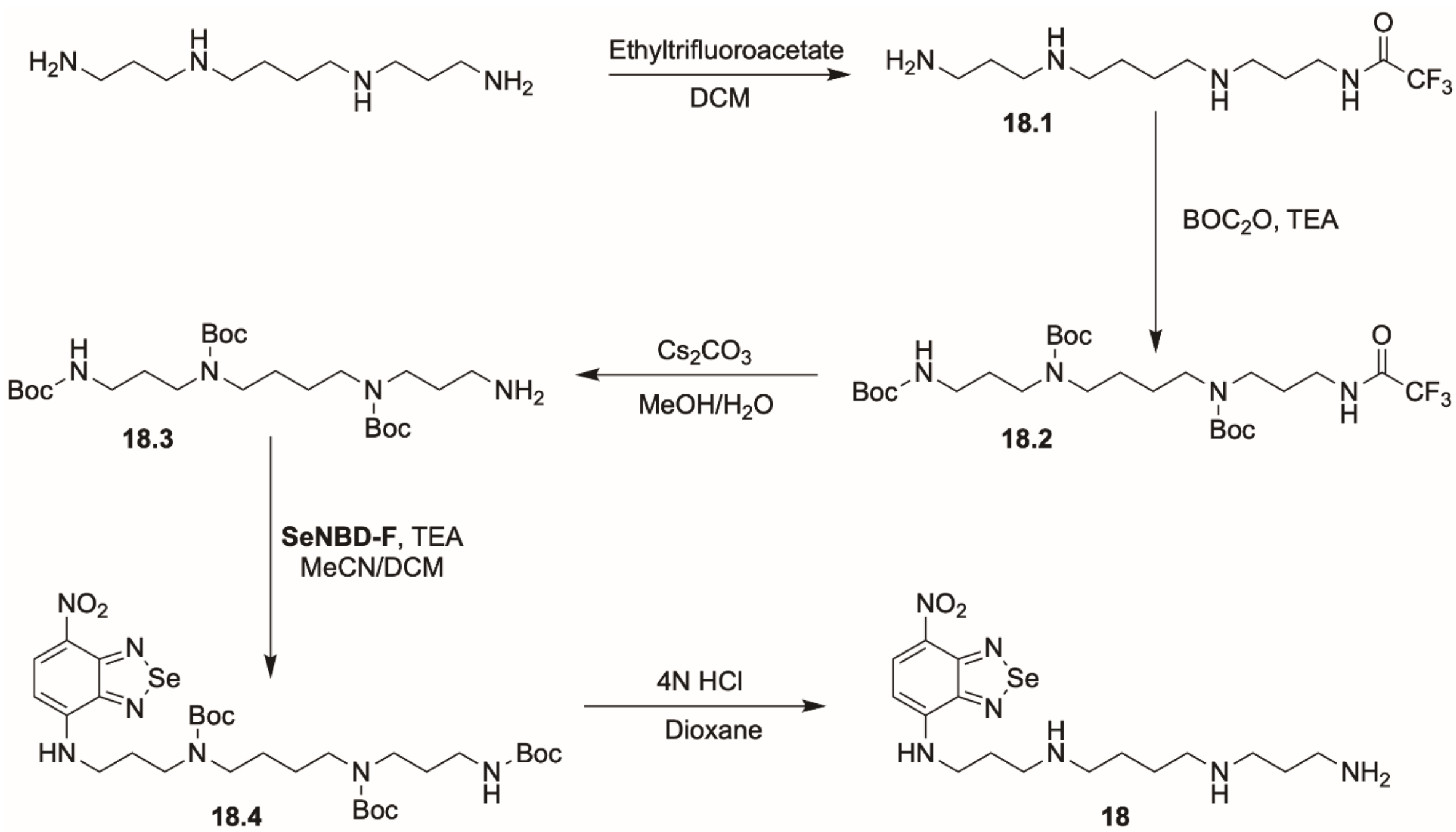

Compound 18.3. Compound **18.3** was synthesized following reported methods.[4] Spermine (102 mg, 0.49 mmol) was dissolved in MeOH (7 mL) and cooled over dry ice. Ethyltrifluoroacetate (58.8 µL, 0.49 mmol) was added dropwise over 3 min. The reaction was monitored over 1 h. Boc_2O (431 mg, 1.97 mmol) was added, and the reaction was allowed to warm to r.t. and triethylamine (275 µL, 1.97 mmol) was also added. Upon reaction completion, an aqueous solution of Cs_2CO_3 (322 mg, 0.98 mmol) was added to raise the pH to 11. The reaction mixture was stirred for 16 h. An additional portion of Cs_2CO_3 was added and the reaction stirred at r.t. for 24 h. The organic product was extracted into CH_2Cl_2 and concentrated *in vacuo* prior to purification by flash column chromatography (5-18% MeOH in CH_2Cl_2 + 0.1% 2 N NH_3) to render compound **18.3** (12 mg, 34%).

^{1}H NMR (500 MHz, MeOD) δ 3.37 (s, 1H), 3.24 (q, *J* = 6.7, 6.0 Hz, 10H), 3.05 (t, *J* = 6.7 Hz, 2H), 1.54 (s, 4H), 1.51 – 1.44 (m, 31H).

^{13}C NMR (126 MHz, MeOD) δ 157.0, 155.9, 117.2, 115.0, 79.7, 79.5, 78.5, 48.4, 44.3, 37.5, 27.3, 27.3, 25.2.

Compound 18.4. SeNBD-F (6.2 mg, 0.025 mmol, 1 eq.) and compound **18.3** (12 mg, assumed 0.024 mmol, 1 eq.) were dissolved in MeCN:DCM (1:1, 2 mL). To this mixture, TEA was added (50 µL, 0.358 mmol, 14 eq.) and stirred at 40 ºC. Upon completion, excess solvent was removed *in vacuo* and the crude product was purified by silica gel chromatography (30-70% EtOAc in hexane) to afford compound **18.4** as an orange oil (6 mg, 35%).

^{1}H NMR (500 MHz, MeOD) δ 8.69 (d, J = 8.9 Hz, 1H), 6.38 (d, J = 8.9 Hz, 1H), 3.55 (t, J = 6.8 Hz, 2H), 3.39 (t, J = 7.1 Hz, 2H), 3.25 (dt, J = 25.4, 7.1 Hz, 10H), 3.07 – 3.01 (m, 2H), 1.60 – 1.41 (m, 29H), 1.27 (s, 2H).

^{13}C NMR (126 MHz, MeOD) δ 152.5, 152.3, 148.4, 135.6, 96.9, 79.7, 79.5, 78.5, 69.2, 54.6, 40.4, 30.6, 28.1, 27.4, 27.3.

Compound 18. Compound **18.4** (6.3 mg, 0.01 mmol) was dissolved in CH_2Cl_2 (500 µL) and a solution of HCl (4 N in dioxane, 500 µL). The resultant mixture was stirred at r.t. until completion. The crude mixture was diluted in H_2O (10 mL) and lyophilized prior to purification by preparative HPLC to afford compound **18** as an orange solid (0.6 mg, 14%).

HRMS (ESI+): calculated for $C_{16}H_{28}N_7O_2Se$: 430.1464, found: 430.1460 $[M+H]^+$.

7-nitrobenzo[c][1,2,5]selenadiazol-4-yl)glycine (19)

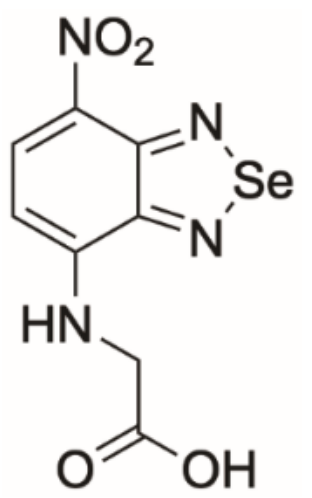

SeNBD-F (50 mg, 0.20 mmol), glycine (31 mg, 0.40 mmol) and $NaHCO_3$ (34 mg, 0.40 mmol) were combined in MeOH:H_2O (1:1, 3 mL) at 55 °C for 15 min. A precipitate was formed which was collected by filtration and rinsed with MeOH to give compound **19** as a red solid (56 mg, 93%).

^{1}H NMR (500 MHz, D_2O) δ 8.41 (d, J = 9.1 Hz, 1H), 5.99 (d, J = 9.1 Hz, 1H), 3.99 (s, 2H).

^{13}C NMR (126 MHz, D_2O) δ 175.4, 151.9, 151.4, 149.0, 137.3, 127.3, 98.9, 46.6.

HRMS (ESI+) m/z calculated for $C_8H_7N_4O_4Se$: 302.9627, found: 302.9619 $[M+H]^+$.

(*S*)-2-amino-3-(4-((7-nitrobenzo[c][1,2,5]selenadiazol-4-yl)amino)phenyl)propanoic acid (20)

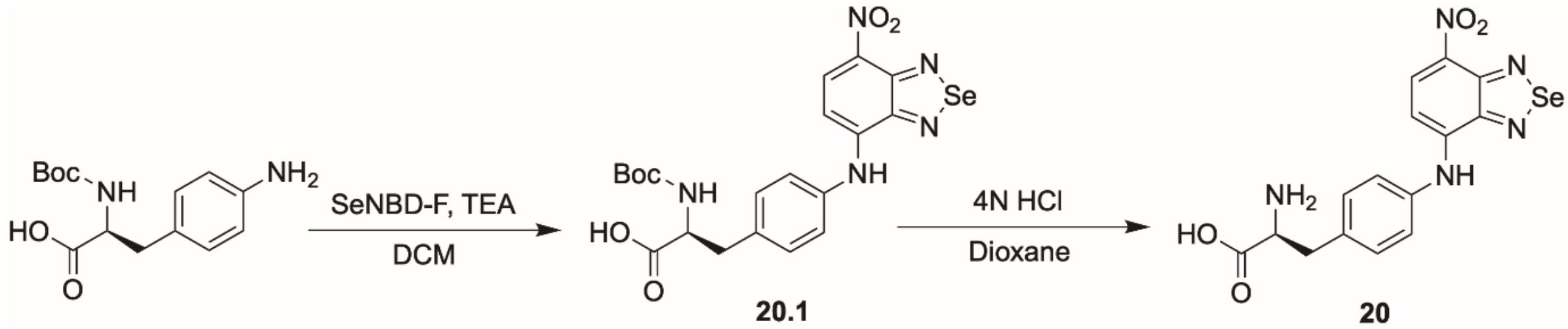

SeNBD-F (19.8 mg, 0.08 mmol), Boc-Phe(4-NH_2)-OEt (29.1 mg, 0.09 mmol) and triethylamine (23 μL, 0.16 mmol) were combined in CH_2Cl_2 (1.5 mL) and stirred at 40 °C for 16 h. The reaction mixture was concentrated *in vacuo* and the crude product purified by column chromatography (0-1% MeOH in CH_2Cl_2) to afford compound **20.1** (31 mg, 77%). Compound **20.1** (16.0 mg, 0.03 mmol) was dissolved in a solution of HCl (4 N, dioxane, 2 mL) and allowed to stir for 1 h at r.t. until complete deprotection. The reaction was quenched with aqueous NaOH (2 N, 4 mL) and the resultant mixture was diluted with H_2O (5 mL) and lyophilized before purification by preparative HPLC to isolate compound **20** as a red solid (12 mg, 98%).

^{1}H NMR (500 MHz, D_2O) δ 7.37 (d, *J* = 10.5 Hz, 1H), 6.93 (d, *J* = 8.0 Hz, 2H), 6.64 (d, *J* = 8.0 Hz, 2H), 5.92 (d, *J* = 10.6 Hz, 1H), 3.26 (dd, *J* = 7.5, 5.4 Hz, 1H), 2.72 (dd, *J* = 13.5, 5.4 Hz, 1H), 2.54 (d, *J* = 7.6 Hz, 1H).

^{13}C NMR (126 MHz, D_2O) δ 182.2, 168.3, 157.5, 154.8, 147.4, 134.4, 129.9, 122.1, 121.2, 57.3, 48.5.

HRMS (MALDI+): calculated for $C_{15}H_{13}N_4O_6SeNa$: 430.0026, found: 430.0028 $[M+Na]^+$.

(*S*)-2-amino-3-(4-(6-((7-nitrobenzo[*c*][1,2,5]selenadiazol-4-yl)amino)hexanamido)phenyl)propanoic acid (23)

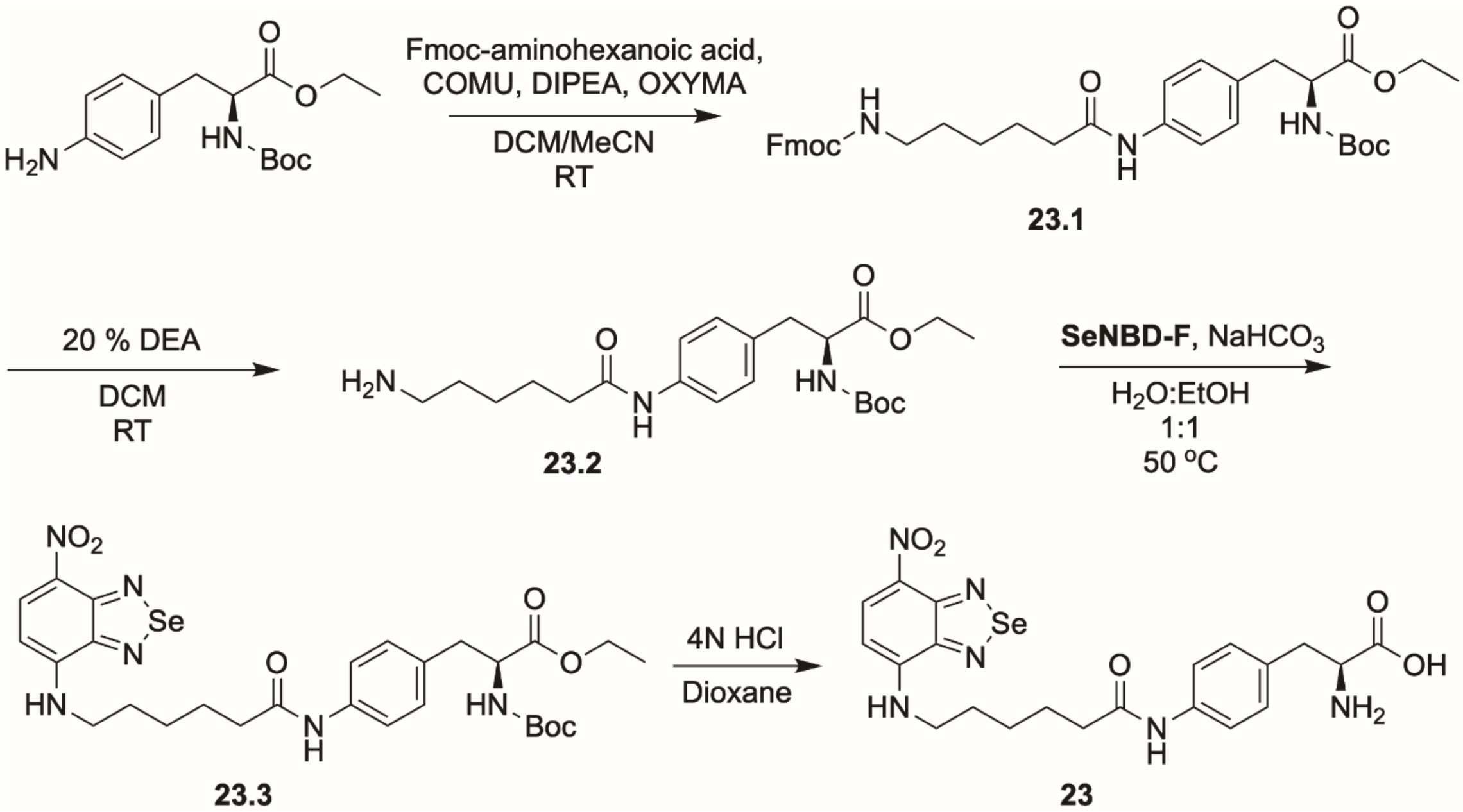

Compound 23.1. Boc-Phe(4-NH_2)-OEt (50 mg, 0.16 mmol), Fmoc-aminohexanoic acid (57.6 mg, 0.16 mmol), COMU (106 mg, 0.24 mmol) and Oxyma (48.7 mg, 0.34 mmol) were all dissolved in a 1:1 CH_2Cl_2:MeOH (2 mL). The resultant mixture was stirred at r.t. for 1 h. DIPEA (42.3 uL, 0.24 mmol) was added and the reaction was stirred for 4 h. The reaction mixture was concentrated *in vacuo* and the crude product was purified by flash column chromatography (2% MeOH in CH_2Cl_2) to afford compound **23.1** as a white solid (49 mg, 48%).

^{1}H NMR (500 MHz, $CDCl_3$) δ 7.78 (dt, *J* = 7.6, 0.9 Hz, 2H), 7.61 (dd, *J* = 7.5, 1.0 Hz, 2H), 7.46 (t, *J* = 8.2 Hz, 2H), 7.41 (dt, *J* = 7.5, 0.9 Hz, 2H), 7.33 (td, *J* = 7.5, 1.2 Hz, 2H), 7.09 (d, *J* = 8.1 Hz, 2H), 4.91 (s, 2H), 4.56 – 4.51 (m, 1H), 4.41 (d, *J* = 6.9 Hz, 2H), 4.23 (t, *J* = 7.0 Hz, 1H), 4.17 (q, *J* = 7.1 Hz, 2H), 3.22 (s, 2H), 3.06 (qd, *J* = 14.0, 13.4, 4.9 Hz, 2H), 2.38 (t, *J* = 7.4 Hz, 2H), 1.77 (s, 2H), 1.62 – 1.53 (m, 2H), 1.49 (dd, *J* = 15.2, 7.0 Hz, 2H), 1.44 (s, 9H), 1.26 (t, *J* = 7.2 Hz, 3H).

^{13}C NMR (126 MHz, $CDCl_3$) δ 171.7, 171.2, 156.5, 155.1, 143.9, 141.3, 136.8, 131.9, 129.9, 127.6, 127.0, 125.0, 119.9, 119.8, 79.9, 66.6, 61.4, 54.4, 54.3, 47.2, 42.5, 40.6, 37.6, 37.3, 29.6, 28.3, 26.1, 24.9, 18.5, 17.2, 14.1, 12.2.

HRMS (ESI+): m/z calculated for $C_{37}H_{46}N_3O_7$: 644.3303, m/z found: 644.3318 $[M+H]^+$.

Compound 23.2. Compound **23.1** (46.8 mg, 0.07 mmol) was dissolved in diethanolamine (1 mL, 20% in CH_2Cl_2) and stirred at r.t. for 24 h. The reaction mixture was concentrated *in vacuo* and the crude product was washed with Et_2O (2 x 12 mL). Compound **23.2** was used without further purification (32 mg, quant.).

^{1}H NMR (500 MHz, $CDCl_3$) δ 7.58 (d, J = 8.5 Hz, 2H), 7.06 (d, J = 8.1 Hz, 2H), 5.02 (d, J = 8.1 Hz, 1H), 4.49 (d, J = 8.1 Hz, 1H), 4.16 (q, J = 7.0 Hz, 2H), 2.43 (t, J = 7.5 Hz, 2H), 1.61 (s, 2H), 1.47 (dd, J = 7.3, 1.1 Hz, 9H), 1.45 (d, J = 1.0 Hz, 4H), 1.42 (s, 9H).

^{13}C NMR (126 MHz, $CDCl_3$) δ 171.8, 155.2, 129.6, 120.1, 79.9, 61.4, 54.6, 54.6, 39.6, 37.6, 36.5, 30.9, 28.3, 26.3, 25.3, 24.5, 14.1.

HRMS (ESI+): m/z calculated for $C_{22}H_{36}N_3O_5$: 422.2650, found: 422.2629 $[M+H]^+$.

Compound 23.3. Compound **23.2** (29.1 mg, 0.07 mmol) and $NaHCO_3$ (11.6 mg, 0.13 mmol) were dissolved in H_2O (1.5 mL) and warmed to 50 °C. **SeNBD-F** (17 mg, 0.07 mmol) was dissolved in EtOH (1.5 mL) and added to the mixture. The reaction was then stirred at 50 °C for 1 h. The reaction mixture was concentrated *in vacuo* and the crude product purified by flash column chromatography (3% MeOH in CH_2Cl_2) to afford compound **23.3** as an orange solid (12 mg, 26%).

^{1}H NMR (500 MHz, $CDCl_3$) δ 8.67 (d, J = 8.7 Hz, 1H), 7.45 (d, J = 8.0 Hz, 2H), 7.25 (s, 1H), 7.10 (d, J = 8.0 Hz, 2H), 6.58 (s, 1H), 6.21 (d, J = 8.8 Hz, 1H), 5.07 – 4.97 (m, 1H), 4.60 – 4.47 (m, 1H), 4.19 (q, J = 7.1 Hz, 2H), 3.49 (t, J = 7.4 Hz, 2H), 3.07 (qd, J = 14.1, 13.3, 5.4 Hz, 2H), 2.43 (t, J = 7.1 Hz, 2H), 1.87 (dp, J = 14.3, 7.3 Hz, 4H), 1.59 (p, J = 7.7, 7.2 Hz, 2H), 1.44 (s, 9H), 1.27 (q, J = 6.9, 5.2 Hz, 3H).

^{13}C NMR (126 MHz, $CDCl_3$) δ 171.7, 170.7, 155.1, 152.5, 152.4, 147.3, 136.7, 135.5, 132.1, 129.9, 119.7, 97.2, 79.9, 61.4, 54.4, 43.2, 37.7, 37.2, 29.7, 28.4, 28.3, 26.5, 24.8, 14.1.

HRMS (ESI+): calculated for $C_{28}H_{37}N_6O_7Se$: 649.1884, found: 649.1893 $[M+H]^+$.

Compound 23. Compound **23.3** (12.4 mg, 0.02 mmol) was dissolved in a 4 N HCl (dioxane, 4 mL). The reaction was left to stir at r.t. overnight. The reaction was stirred at 60 °C for 3 h, then concentrated *in vacuo*. The crude product was diluted in water and washed with CH_2Cl_2 (4 × 15 mL). The aqueous layer was preserved and lyophilized. The crude product was purified by preparative HPLC to give compound **23** as a red solid (5.5 mg, 53%).

^{1}H NMR (500 MHz, DMSO-d_6) δ 9.81 (s, 1H), 8.57 (d, *J* = 9.0 Hz, 1H), 8.50 (t, *J* = 6.3 Hz, 1H), 7.46 (d, *J* = 8.4 Hz, 2H), 7.12 (d, *J* = 8.5 Hz, 2H), 6.38 (d, *J* = 9.1 Hz, 1H), 4.02 (s, 1H), 3.09 – 2.94 (m, 2H), 2.30 (t, *J* = 7.3 Hz, 2H), 1.77 – 1.68 (m, 2H), 1.67 – 1.59 (m, 2H), 1.40 (q, *J* = 7.6 Hz, 2H).

^{13}C NMR (126 MHz, DMSO-d_6) δ 171.5, 170.8, 152.4, 152.3, 149.0, 138.7, 135.9, 130.1, 128.2, 119.4, 97.7, 54.1, 43.1, 36.5, 35.9, 28.0, 26.3, 25.2.

HRMS (ESI+): calculated for $C_{21}H_{25}N_6O_5Se$: 521.1046, found: 521.1058 $[M+H]^+$.

(2R,3S,4S,5R,6S)-2-(acetoxymethyl)-6-(2-nitro-4-((((4-nitrophenoxy)carbonyl)oxy)methyl)phenoxy)tetrahydro-2H-pyran-3,4,5-triyl triacetate (25)

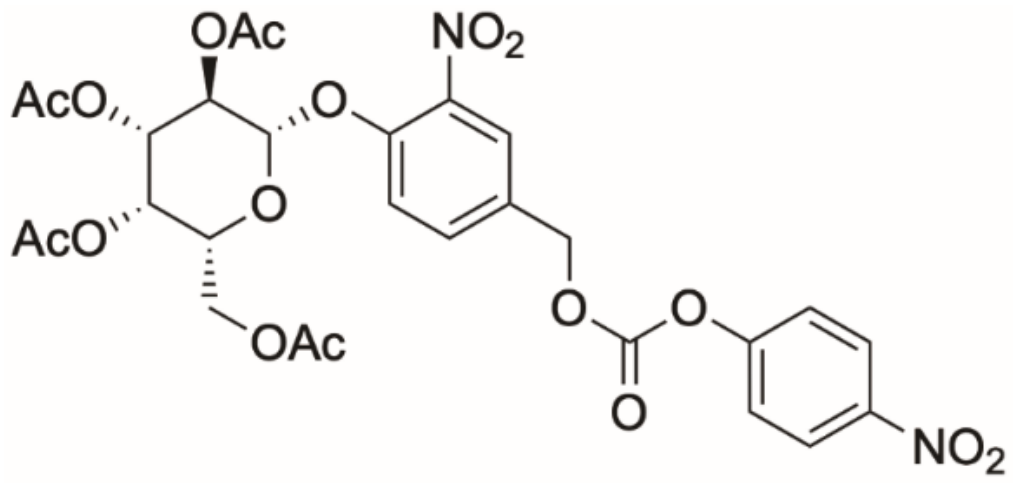

(2R,3S,4S,5R,6S)-2-(acetoxymethyl)-6-(4-(hydroxymethyl)-2-nitrophenoxy)tetrahydro-2H-pyran-3,4,5-triyltriacetate **24** (250 mg, 0.50 mmol) and bis(4-nitrophenyl) carbonate (304 mg, 1.00 mmol) were dissolved in dry DMF (3 mL) and stirred until fully dissolved. DIPEA (196 µL, 1.13 mmol) was added and stirred under N_2 for 1 h. The reaction was monitored using HPLC-MS and TLC. The reaction mixture was evaporated to dryness by azeotropic distillation with heptane and purified using flash chromatography (0-70% EtOAc in hexane) to afford compound **25** as a colorless oil (300 mg, 90%).

^{1}H NMR (500 MHz, DMSO-d_6) δ 8.32 (d, *J* = 9.2 Hz, 2H), 8.04 (d, *J* = 2.2 Hz, 1H), 7.83 (dd, *J* = 8.7, 2.2 Hz, 1H), 7.58 (d, *J* = 9.2 Hz, 2H), 7.46 (d, *J* = 8.7 Hz, 1H), 5.63 (d, *J* = 7.5 Hz, 1H), 5.38 (dd, J = 3.3, 1.2 Hz, 1H), 5.33 (s, 2H), 5.30 – 5.22 (m, 2H), 4.50 (ddd, *J* = 6.9, 5.5, 1.2 Hz, 1H), 4.18 – 4.09 (m, 2H), 2.15 (s, 3H), 2.04 (s, 3H), 2.03 (s, 3H), 1.95 (s, 3H).

^{13}C NMR (126 MHz, DMSO-d_6) δ 169.9, 169.8, 169.5, 168.8, 155.2, 151.7, 148.4, 145.2, 140.1, 134.3, 130.1, 125.4, 122.5, 117.7, 98.4, 70.8, 69.8, 68.5, 67.6, 67.0, 61.2, 20.4, 20.3, 20.3, 20.2.

HRMS (ESI+) calculated for $C_{28}H_{28}N_2O_{17}Na^+$: 687.1280, found: 687.1283 $[M+Na]^+$.

(2R,3S,4S,5R,6S)-2-(acetoxymethyl)-6-(4-(10,10-dimethyl-4-(7-nitrobenzo[c][1,2,5] selenadiazol-4-yl)-3,8-dioxo-2,9-dioxa-4,7-diazaundecyl)-2-nitrophenoxy)tetrahydro-2H-pyran-3,4,5-triyl triacetate (26)

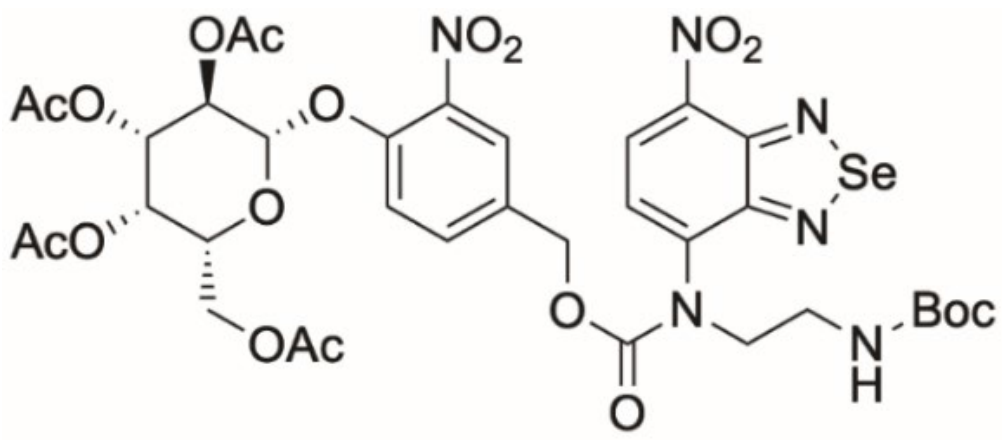

Compound **25** (300 mg, 0.45 mmol), compound **3.1** (209 mg, 0.54 mmol) and Cs_2CO_3 (441 mg, 1.35 mmol) were dissolved in dry DMF (20 mL) and stirred under N_2 for 1.5 h. The reaction was monitored using HPLC-MS and TLC. The reaction mixture was concentrated *in vacuo* by azeotropic distillation with heptane and purified using flash column chromatography (0-80% MeOH in CH_2Cl_2) to afford compound **26** as an orange oil (364 mg, 89%).

^{1}H NMR (500 MHz, DMSO-d$_6$) δ 8.55 (d, *J* = 7.9 Hz, 1H), 7.75 (d, *J* = 7.9 Hz, 1H), 7.67 (d, *J* = 2.2 Hz, 1H), 7.52 (dd, *J* = 8.7, 2.1 Hz, 1H), 7.33 (d, *J* = 8.7 Hz, 1H), 6.77 (t, *J* = 5.9 Hz, 1H), 5.55 (d, *J* = 7.7 Hz, 1H), 5.36 (dd, *J* = 3.3, 1.2 Hz, 1H), 5.30 – 5.18 (m, 2H), 5.10 (s, 2H), 4.46 (ddd, *J* = 6.9, 5.6, 1.2 Hz, 1H), 4.18 – 4.07 (m, 2H), 3.84 (t, *J* = 6.3 Hz, 2H), 3.13 (q, *J* = 6.2 Hz, 2H), 2.14 (s, 3H), 2.02 (s, 3H), 2.01 (s, 3H), 1.94 (s, 3H), 1.26 (s, 9H).

^{13}C NMR (126 MHz, DMSO-d$_6$) δ 169.9, 169.8, 169.4, 168.8, 156.5, 155.3, 154.2, 150.8, 147.9, 140.0, 140.0, 139.5, 133.1, 131.7, 127.4, 124.9, 123.6, 117.7, 98.5, 77.6, 70.7, 69.8, 67.6, 67.0, 65.3, 61.1, 49.4, 38.7, 28.0, 20.4, 20.3, 20.2, 20.2.

HRMS (ESI+): m/z calculated for $C_{35}H_{40}N_6O_{18}Se^+$: 935.1637, found: 935.1623 $[M]^+$.

3-nitro-4-(((2S,3R,4S,5R,6R)-3,4,5-trihydroxy-6-(hydroxymethyl) tetrahydro-2H-pyran-2-yl)oxy)benzyl (2-aminoethyl)(7-nitrobenzo[c][1,2,5]selenadiazol-4-yl)carbamate (27)

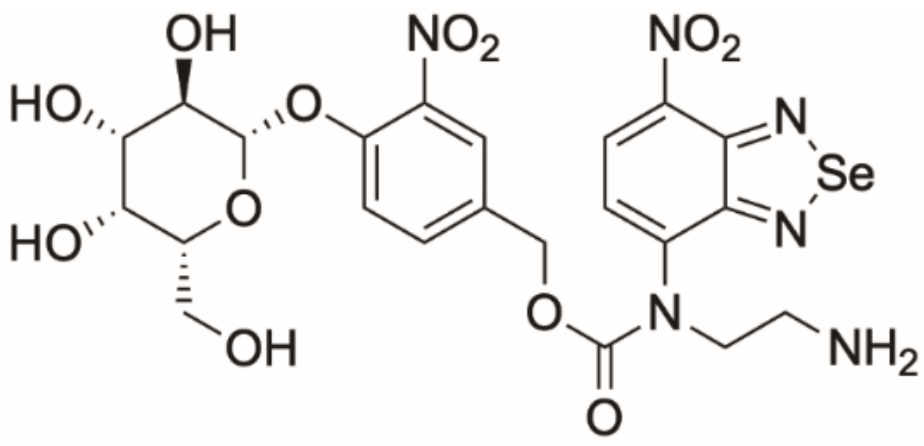

4N HCl in 1,4-dioxane was prepared by adding hydrochloric acid (12 M, 40 mL) to dioxane (80 mL). The acid solution was then added to dissolve compound **26** (364 mg, 0.399 mmol, 1.0 eq) and the reaction mixture was stirred at room temperature overnight. The solution was diluted with 1:1 H_2O:MeCN mixture before being purified by preparative HPLC to yield compound **27** as a yellow powder (51 mg, 20%).

^{1}H NMR (500 MHz, DMSO-d_6) δ 8.57 (d, *J* = 7.9 Hz, 1H), 7.99 (t, *J* = 5.9 Hz, 2H), 7.89 (d, *J* = 7.9 Hz, 1H), 7.65 (d, *J* = 2.2 Hz, 1H), 7.47 (dd, *J* = 8.9, 2.2 Hz, 1H), 7.35 (d, *J* = 8.8 Hz, 1H), 5.10 (s, 2H), 5.01 (d, *J* = 7.6 Hz, 1H), 4.01 (t, *J* = 6.6 Hz, 2H), 3.70 (dd, *J* = 3.3, 1.0 Hz, 1H), 3.62 (td, *J* = 6.1, 1.0 Hz, 1H), 3.58 – 3.52 (m, 2H), 3.47 (dd, *J* = 10.9, 6.5 Hz, 1H), 3.36 (s, 4H), 3.40 (dd, *J* = 9.5, 3.3 Hz, 1H), 3.01 (q, *J* = 6.2 Hz, 2H).

^{13}C NMR (126 MHz, DMSO-d_6) δ 157.0, 154.9, 151.4, 149.8, 140.4, 140.1, 139.4, 133.9, 130.1, 127.9, 126.4, 124.4, 117.4, 101.4, 76.2, 73.8, 70.4, 68.4, 66.2, 60.7, 47.6, 37.8.

HRMS (ESI+) m/z calculated for $C_{22}H_{25}N_6O_{12}Se^+$: 645.0690, found: 645.0683 $[M+H]^+$.

3-nitro-4-(((2S,3R,4S,5R,6R)-3,4,5-trihydroxy-6-(hydroxymethyl) tetrahydro-2H-pyran-2-yl)oxy)benzyl (7-nitrobenzo[c][1,2,5]selenadiazol-4-yl)(2-oleamidoethyl)carbamate (Gal-SeNBD-oleamide)

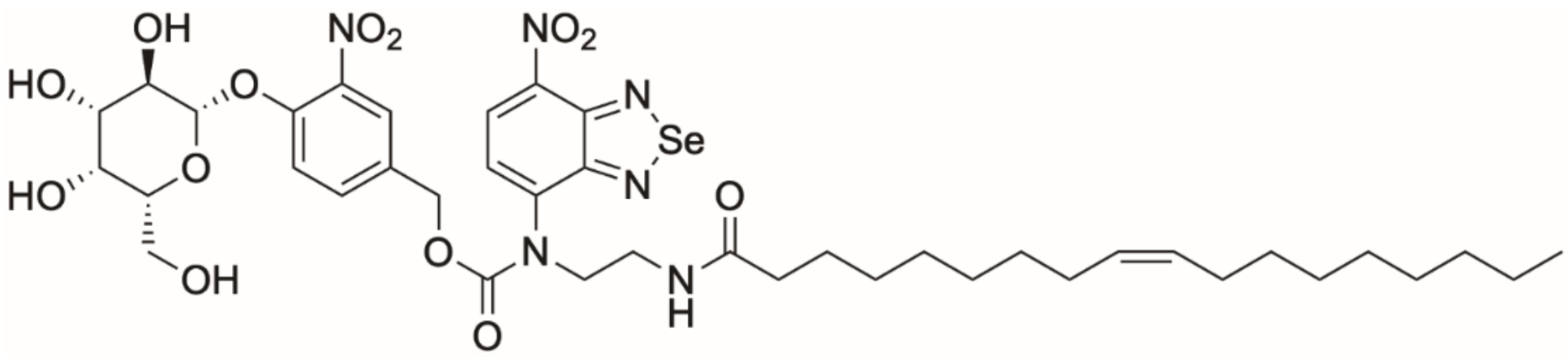

To a solution of oleic acid (17.6 mg, 62.2 µmol) in DMF (300 µL), DIPEA (11.7 µL, 62.2 µmol) was added and stirred for 5 min. HATU (28.4 mg, 74.6 µmol) was then added to the reaction mixture and stirred for 10 min. In a separate flask, compound **27** (20.0 mg, 31.1 µmol) was dissolved in DMF (700 µL). The two mixtures were combined and stirred for 1 h. The solution was diluted with 1:1 H_2O:MeCN mixture before being purified by semi-preparative HPLC to render **Gal-SeNBD-oleamide** as a yellow powder (2.0 mg, 7%).

^{1}H NMR (500 MHz, DMSO-d$_6$) δ 8.54 (d, *J* = 7.9 Hz, 1H), 7.75 (d, *J* = 8.0 Hz, 1H), 7.61 (d, *J* = 2.1 Hz, 1H), 7.44 (dd, *J* = 8.9, 2.2 Hz, 1H), 7.34 (d, *J* = 8.8 Hz, 1H), 5.35 – 5.27 (m, 2H), 5.12 (d, *J* = 5.2 Hz, 1H), 5.07 (s, 2H), 5.00 (d, *J* = 7.7 Hz, 1H), 4.87 (d, *J* = 5.9 Hz, 1H), 4.64 (t, *J* = 5.5 Hz, 1H), 4.57 (d, *J* = 4.5 Hz, 1H), 3.84 (t, *J* = 6.3 Hz, 2H), 3.70 (td, *J* = 3.7, 2.0 Hz, 1H), 3.62 (t, *J* = 6.2 Hz, 1H), 3.58 – 3.52 (m, 2H), 3.48 (ddd, *J* = 10.8, 6.5, 5.4 Hz, 1H), 3.42 – 3.36 (m, 1H), 3.25 (q, *J* = 6.1 Hz, 2H), 1.96 (p, *J* = 6.4 Hz, 4H), 1.87 (t, *J* = 7.5 Hz, 2H), 1.36 – 1.02 (m, 22H), 0.83 (t, 3H).

^{13}C NMR (126 MHz, DMSO-d$_6$) δ 172.0, 156.4, 154.1,150.6, 149.1, 139.8, 139.4, 139.3, 133.1, 129.6, 129.4, 127.3, 124.8, 123.6, 116.8, 100.8, 75.6, 73.1, 69.7, 67.7, 65.3, 60.0, 49.1, 37.2, 35.0, 31.0, 28.9, 28.8, 28.6, 28.4, 28.4, 28.4, 28.3, 28.3, 26.3, 26.3, 24.8, 21.8, 13.7.

HRMS (ESI+): m/z calculated for $C_{40}H_{56}N_6O_{13}SeNa^+$: 931.2963, found: 931.2974 $[M+Na]^+$.

***N*-(2-((7-nitrobenzo[*c*][1,2,5]oxadiazol-4-yl)amino)ethyl)oleamide (28)**

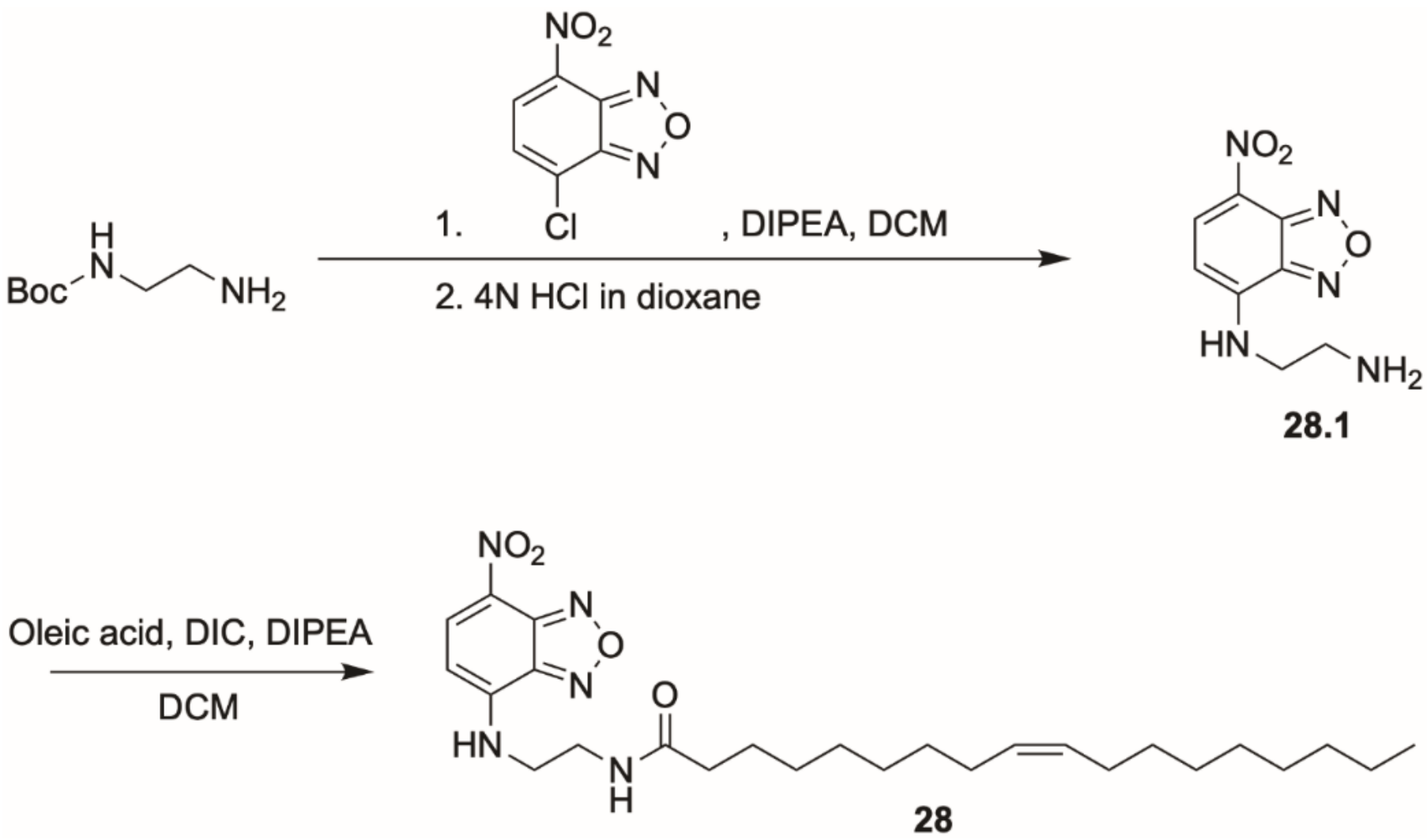

Compound 28.1. 4-Chloro-7-nitrobenzofurazan (100 mg, 0.5 mmol) was added to a solution of N-Boc-1,2-diaminoethane (131 mg, 0.75 mmol) in CH_2Cl_2 (3 mL). DIPEA (174 µL, 1.0 mmol) was added and the reaction mixture was stirred at r.t. for 24 h. The reaction mixture was concentrated *in vacuo* and TFA (80% in CH_2Cl_2, 3 mL) was added and the mixture was stirred for 15 min. The mixture was purified by preparative HPLC which yielded compound **28** as a yellow solid (67 mg, 26%).

^{1}H NMR (500 MHz, DMSO) δ 9.44 – 9.20 (m, 1H), 8.58 (d, *J* = 8.9 Hz, 1H), 7.96 (s, 2H), 6.49 (d, *J* = 8.9 Hz, 1H), 3.78 – 3.74 (m, 2H), 3.16 (t, *J* = 6.3 Hz, 2H).

^{13}C NMR (126 MHz, DMSO) δ 158.1, 157.9, 144.4, 137.6, 121.5, 99.4, 40.7, 37.1.

HRMS (ESI+): m/z calculated for $C_8H_{10}N_5O_3$: 224.0778, found: 224.0769 $[M+H]^+$.

Compound 28. To a solution of oleic acid (51 mg, 0.18 mmol), DIC (28 μL, 0.18 mmol) and DIPEA (32 μL, 0.18 mmol) in CH_2Cl_2 (3 mL), compound **28.1** (20 mg, 0.090 mmol) was added and the solution was stirred overnight at r.t. The reaction mixture was concentrated *in vacuo* and purified by preparative HPLC, which yielded compound **28** as a yellow solid (17 mg, 39%).

^{1}H NMR (500 MHz, DMSO-d_6) δ 9.39 (s, 1H), 8.52 (d, *J* = 8.9 Hz, 1H), 7.99 (t, *J* = 5.8 Hz, 1H), 6.44 (d, *J* = 9.0 Hz, 1H), 5.41 – 5.21 (m, 2H), 3.54 (t, *J* = 6.1 Hz, 2H), 3.38 (q, *J* = 6.1 Hz, 2H), 2.03 (t, *J* = 7.4 Hz, 2H), 1.95 (ddd, *J* = 13.1, 7.2, 5.8 Hz, 4H), 1.43 (t, *J* = 7.2 Hz, 2H), 1.32 – 1.09 (m, 20H), 0.88 – 0.81 (m, 3H).

^{13}C NMR (126 MHz, DMSO-d_6) δ 172.7, 145.3, 144.3, 143.9, 137.7, 129.5, 129.4, 120.6, 99.0, 43.1, 37.1, 35.2, 31.1, 28.9, 28.7, 28.5, 28.4, 28.4, 28.3, 26.4, 26.4, 24.9, 21.9, 13.8.

HRMS (ESI+): m/z calculated for $C_{26}H_{42}N_5O_4$: 488.3231, found: 488.3233 $[M+H]^+$.

N-(2-((7-nitrobenzo[c][1,2,5]oxadiazol-4-yl)amino)ethyl)dodecanamide (29)

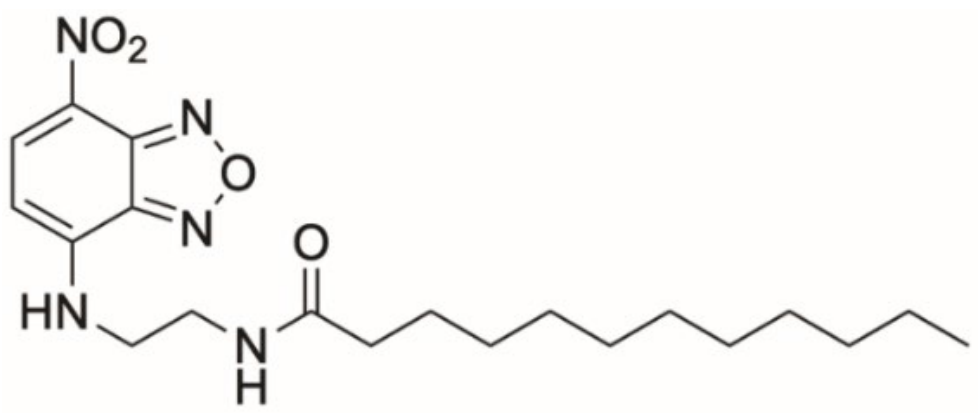

To a solution of lauric acid (36 mg, 0.18 mmol), DIC (28 μL, 0.18 mmol) and DIPEA (32 μL, 0.18 mmol) in CH_2Cl_2 (3 mL), compound **28.1** (20 mg, 0.090 mmol) was added and the solution was stirred overnight at r.t. The reaction mixture was concentrated *in vacuo* and purification by preparative HPLC yielded compound **29** as a yellow solid (13 mg, 36%).

^{1}H NMR (500 MHz, DMSO-d_6) δ 9.38 (t, *J* = 6.0 Hz, 1H), 8.54 (d, *J* = 8.9 Hz, 1H), 7.99 (t, *J* = 5.8 Hz, 1H), 6.45 (d, *J* = 9.0 Hz, 1H), 3.54 (d, *J* = 6.3 Hz, 2H), 3.38 (q, *J* = 6.1 Hz, 2H), 2.29 – 1.93 (m, 2H), 1.52 – 1.36 (m, 2H), 1.36 – 1.06 (m, 16H), 0.86 (td, *J* = 6.9, 3.0 Hz, 3H).

^{13}C NMR (126 MHz, DMSO-d_6) δ 174.3, 172.7, 145.2, 144.3, 143.9, 137.7, 120.7, 99.0, 43.1, 37.1, 35.2, 33.5, 31.1, 28.8, 28.8, 28.7, 28.7, 28.6, 28.6, 28.5, 28.4, 28.4, 24.9, 24.3, 21.9, 13.8.

HRMS (ESI+): m/z calculated for $C_{20}H_{32}N_5O_4$: 406.2449, found: 406.2444 $[M+2H]^{+2}$.

_N_-(2-((7-nitrobenzo[_c_][1,2,5]oxadiazol-4-yl)amino)ethyl)butyramide (30)

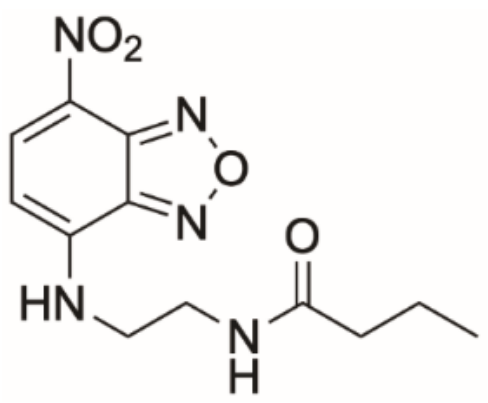

To a solution of butyric acid (16 mg, 0.18 mmol), DIC (28 μL, 0.18 mmol) and DIPEA (32 μL, 0.18 mmol) in CH_2Cl_2 (3 mL), compound **28.1** (20 mg, 0.09 mmol). was added and the solution was stirred overnight at r.t. The reaction mixture was concentrated *in vacuo* and the crude was purified by HPLC which yielded compound **30** as a yellow solid (10 mg, 38%).

^{1}H NMR (500 MHz, DMSO-d_6) δ 9.42 (t, *J* = 6.0 Hz, 1H), 8.54 (d, *J* = 8.9 Hz, 1H), 8.01 (t, *J* = 5.9 Hz, 1H), 6.45 (d, *J* = 8.9 Hz, 1H), 3.54 (d, *J* = 6.0 Hz, 2H), 3.42 – 3.36 (m, 2H), 2.03 (t, *J* = 7.3 Hz, 2H), 1.49 (h, *J* = 7.4 Hz, 2H), 0.82 (t, *J* = 7.4 Hz, 3H).

^{13}C NMR (126 MHz, DMSO-d_6) δ 172.5, 145.2, 144.3, 143.9, 137.8, 120.7, 99.0, 43.0, 37.1, 37.0, 18.3, 13.4.

HRMS (ESI+): m/z calculated for $C_{12}H_{16}N_5O_4$: 294.1197, found: 294.1199 $[M+H]^+$.

3. Supplementary Figures and Tables

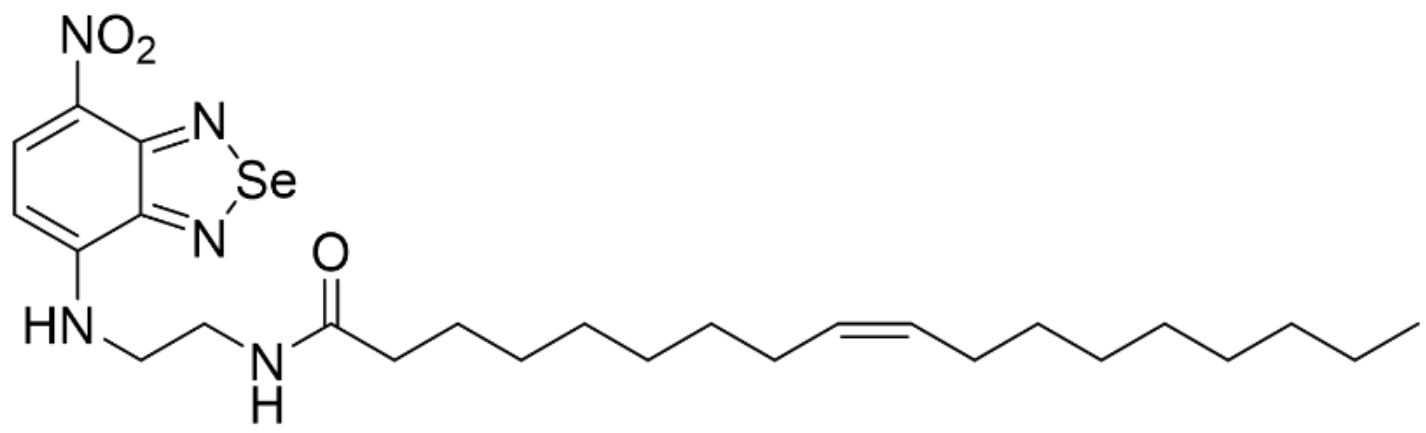

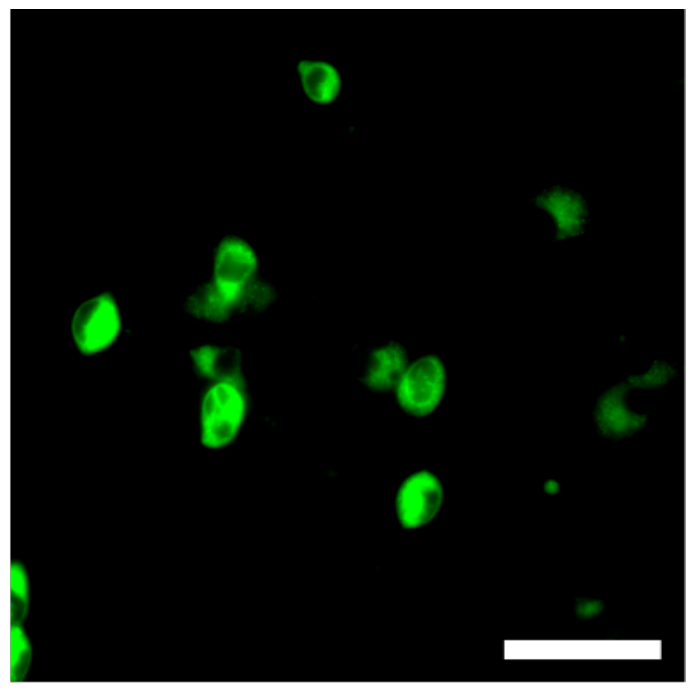

ONBD-oleamide

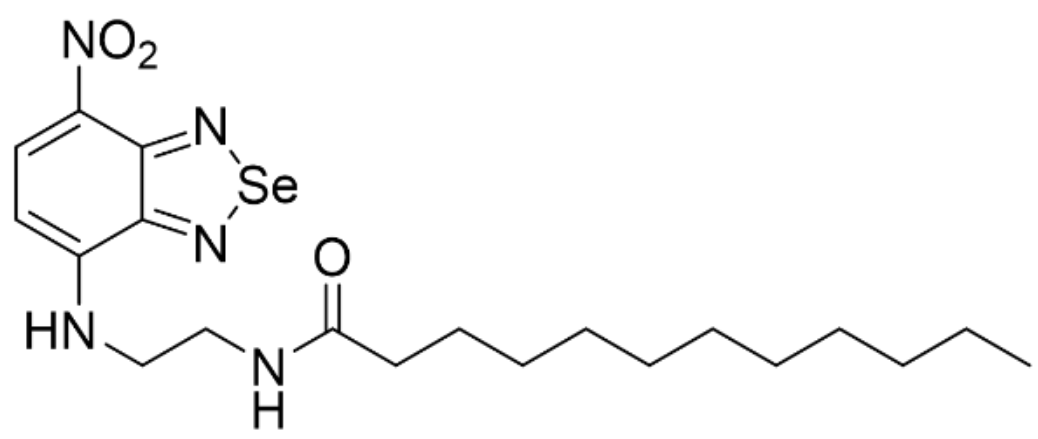

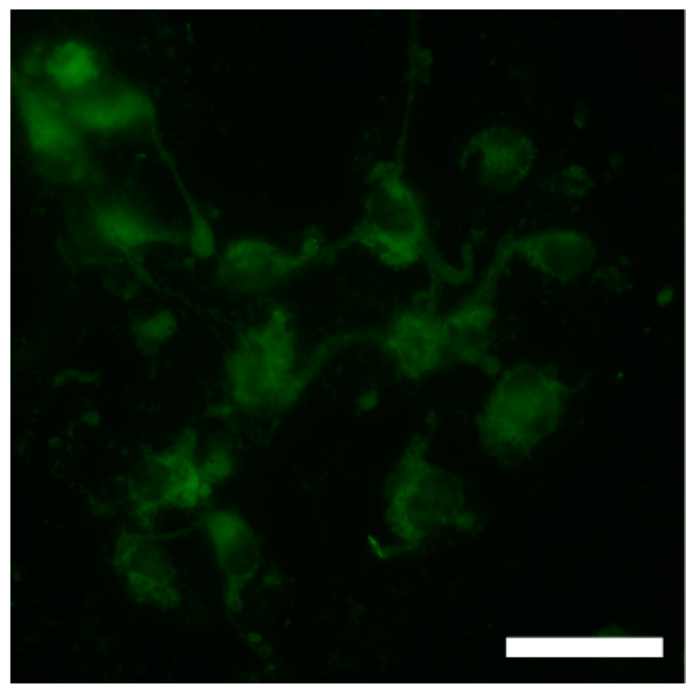

29

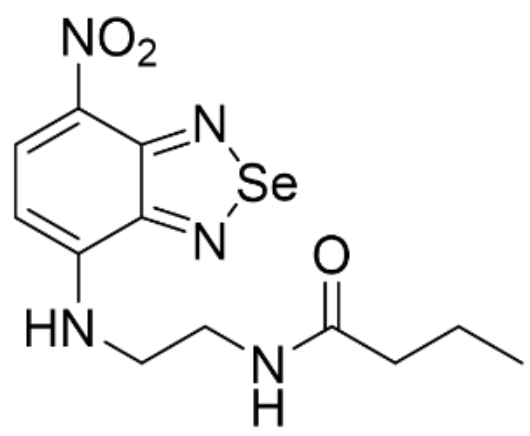

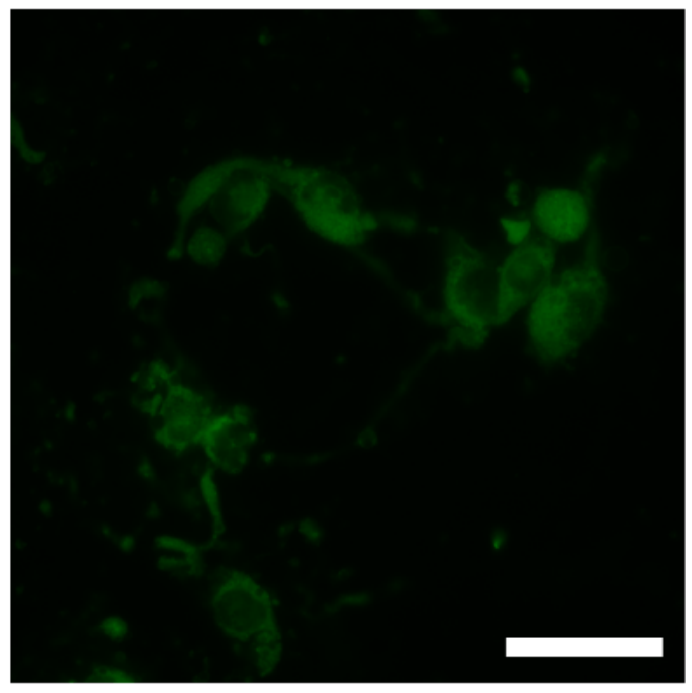

30

Figure S1. Fluorescence microscopy images of E17 cells after incubation with benzoxadiazole analogues of fatty acid conjugates. Representative fluorescence microscopy images of E17 cells after incubation with **ONBD-oleamide** (oleic acid derivative), compound **29** (lauric acid derivative) and compound **30** (butyric acid derivative), all at 10 μM for 30 min at 37°C. Exc. 470 nm; em. 525±25 nm. Scale bars: 50 μm.

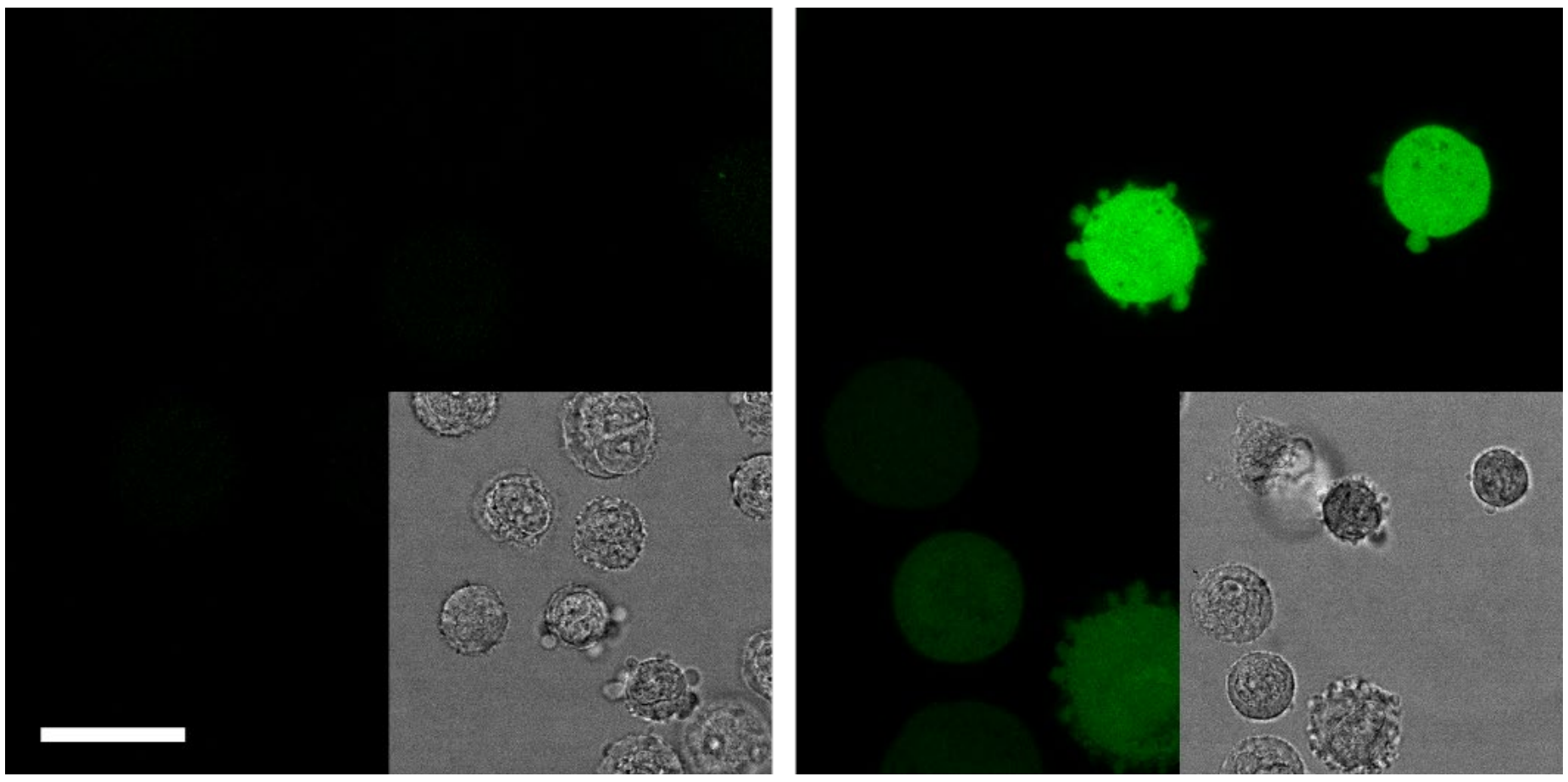

Figure S2. Fluorescence microscopy images of E17 cells after incubation with SeNBD-oleamide and DCFH-DA. Representative brightfield and fluorescence microscopy images of E17 cells after incubation with only **SeNBD-oleamide** (10 μM) (left) or **SeNBD-oleamide** (10 μM) plus DCFH-DA (10 μM, 30 min at 37 °C) (right), followed by illumination (15 mW cm^{-2}, 520 nm, 60 min). Exc. 490 nm; em. 525±25 nm. Scale bar: 10 μm.

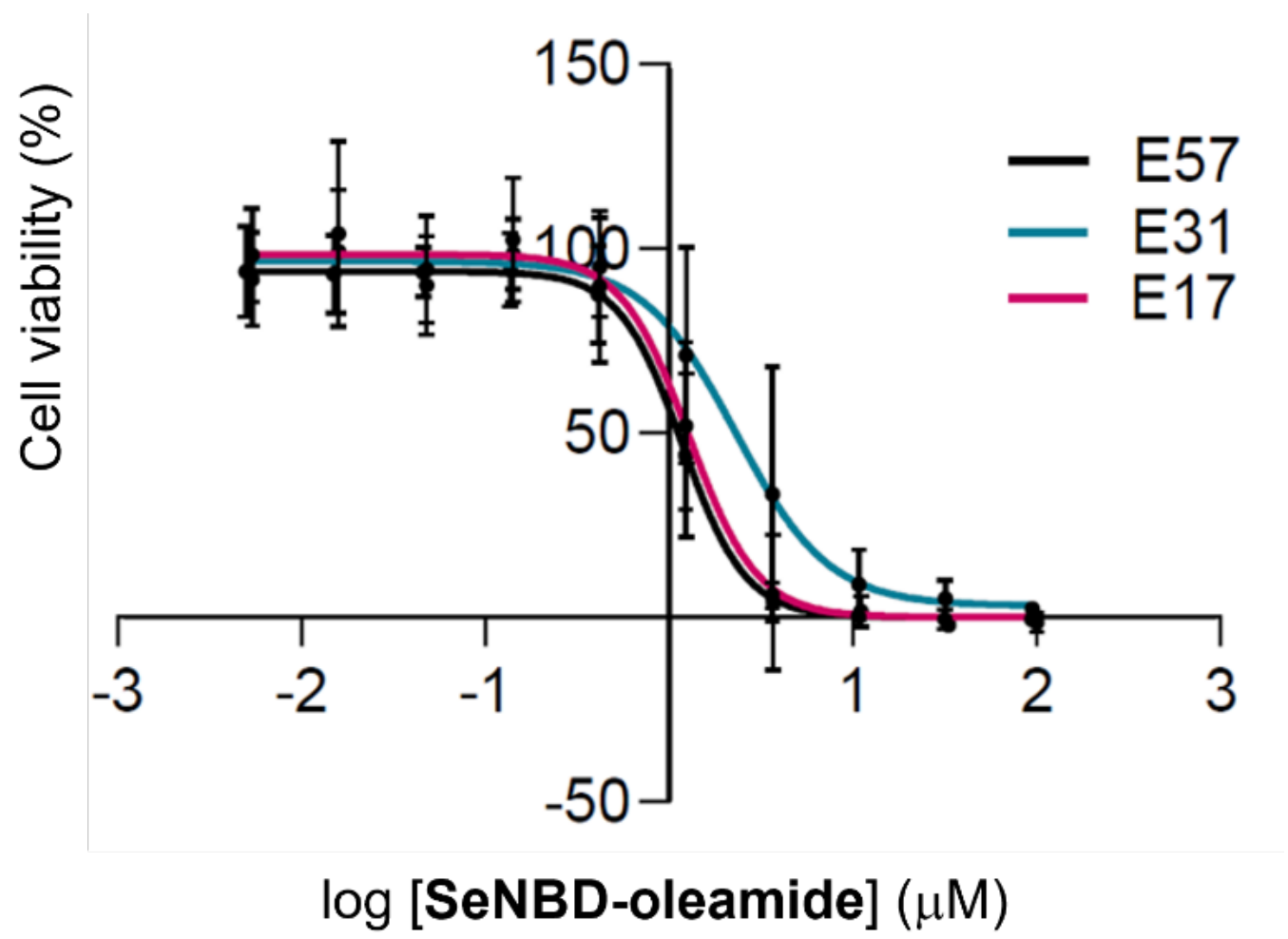

Figure S3. Half-maximal inhibitory concentrations of compound 4 in different patient-derived cell lines. Phototoxicity analysis of compound **4** (**SeNBD-oleamide**) in patient-derived glioma cell lines: classical (E17, red), proneuronal (E31, blue), mesenchymal (E57, black). Values presented as means±SD (n=4). IC_{50} values were determined as 0.6±0.3 μM for E17 cells, 3.1±1.0 μM for E31 cells, and 1.3±0.1 μM for E57 cells.

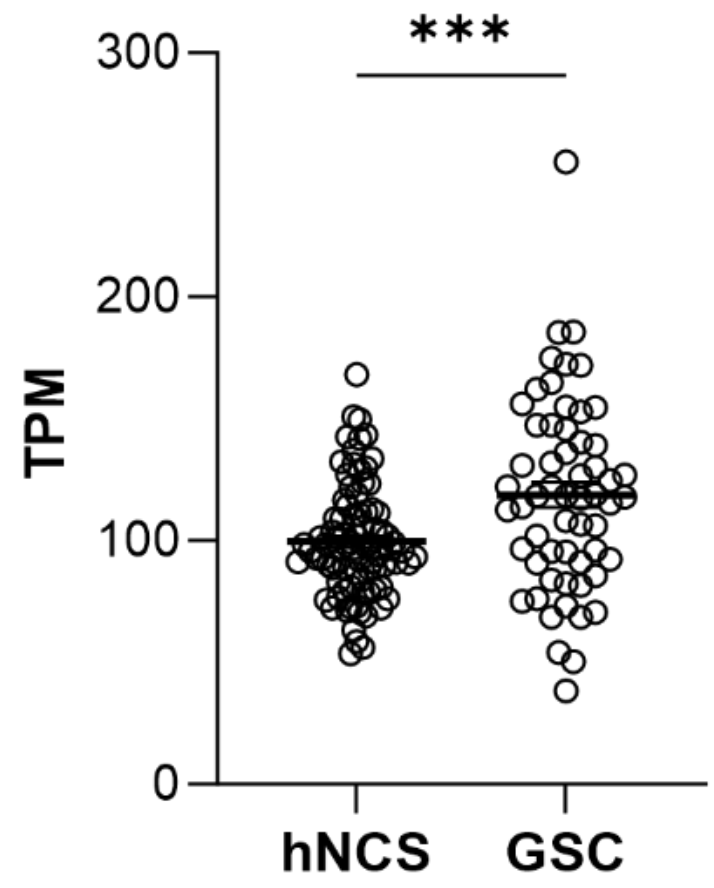

Figure S4. Transcriptomic analysis of human β-galactosidase gene (*GLB1*) in human neural stem cells (hNSCs) and human glioma stem cells (GSC). Gene expression data (as transcripts per million) presented as means±SEM (n=58). P values were determined two-tailed unpaired t test (*** for $p<0.001$).

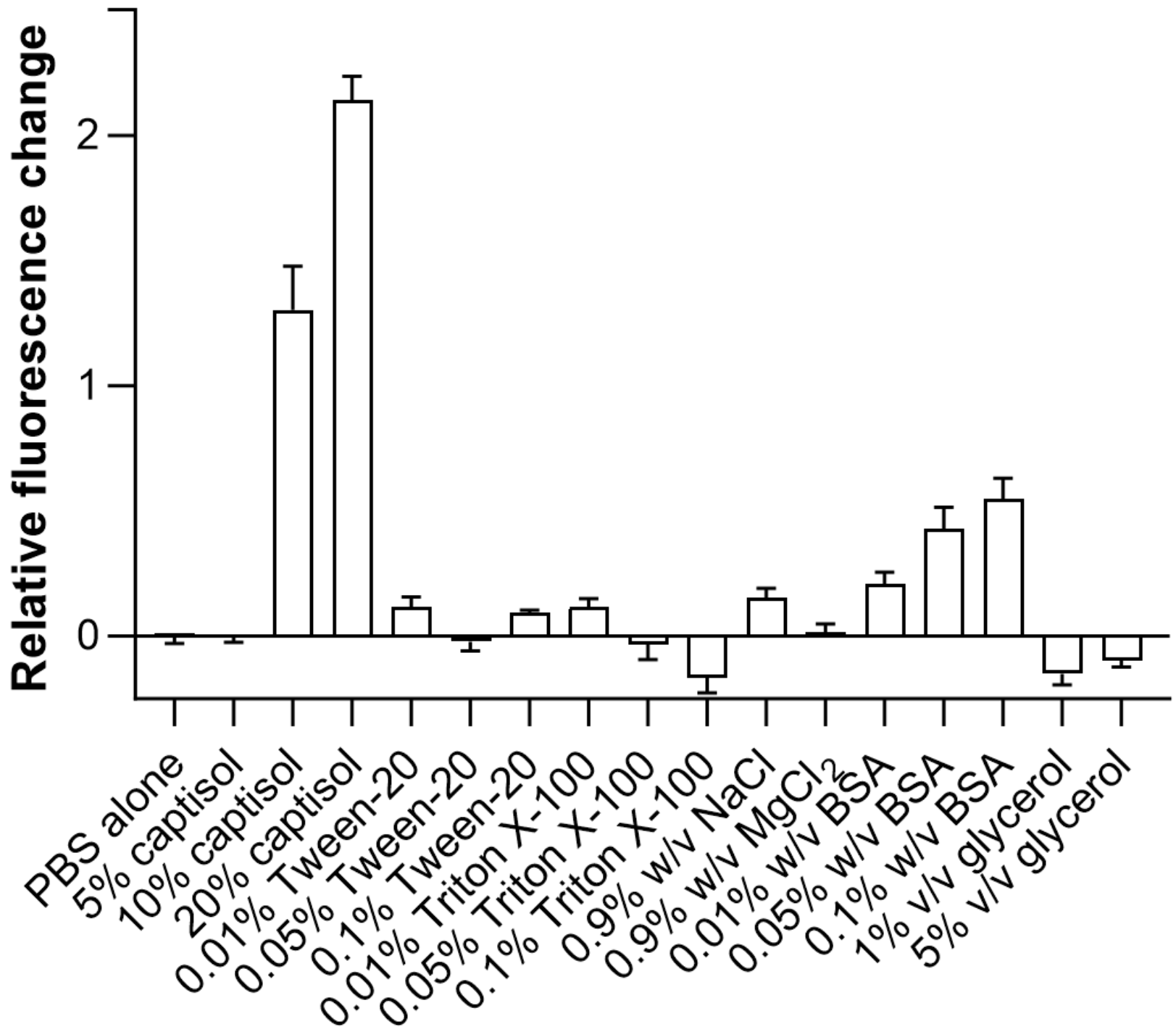

Figure S5. Optimization of Gal-SeNBD-oleamide formulation in the reaction with β-galactosidase. Excipients were included in the solutions of **Gal-SeNBD-oleamide** (10 μM) in supplemented PBS buffers, followed by addition of β-galactosidase (20 nM) and incubation at 37°C. Relative fluorescence fold changes (exc. 520 nm, em. 590 nm) before and after addition of β-galactosidase are presented as means±SEM (n=3).

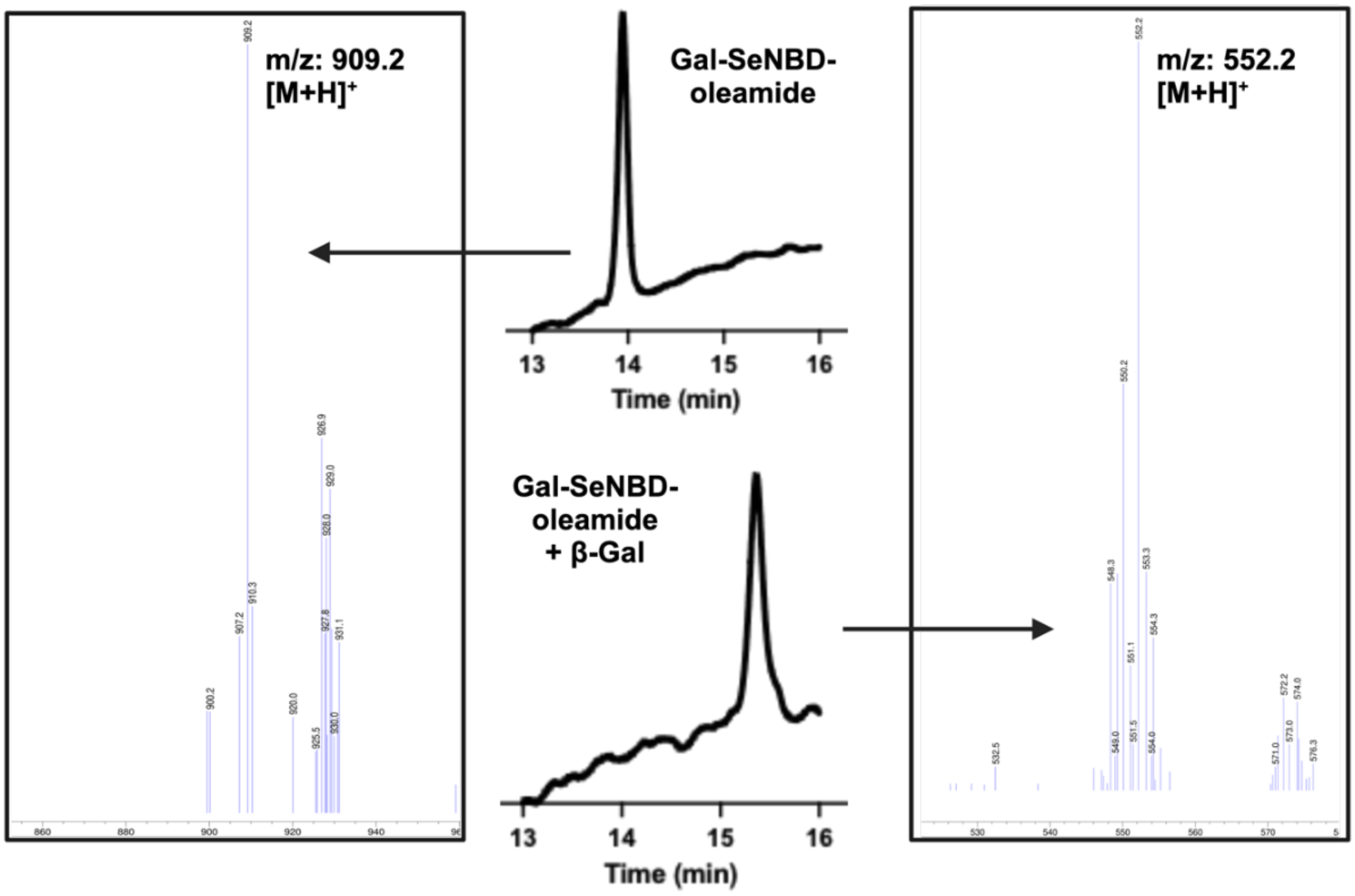

Figure S6. HPLC-MS analysis of Gal-SeNBD-oleamide before and after reaction with β-galactosidase. HPLC traces (UV detection: 254 nm) and MS spectra of **Gal-SeNBD-oleamide** (10 µM) in the absence (top panel) and presence (bottom panel) of β-galactosidase (100 nM) for 2 h at 37°C. Calculated and experimental $[M+H]^+$ m/z values (**Gal-SeNBD-oleamide**: m/z 909.3; **SeNBD-oleamide**: m/z 552.2).

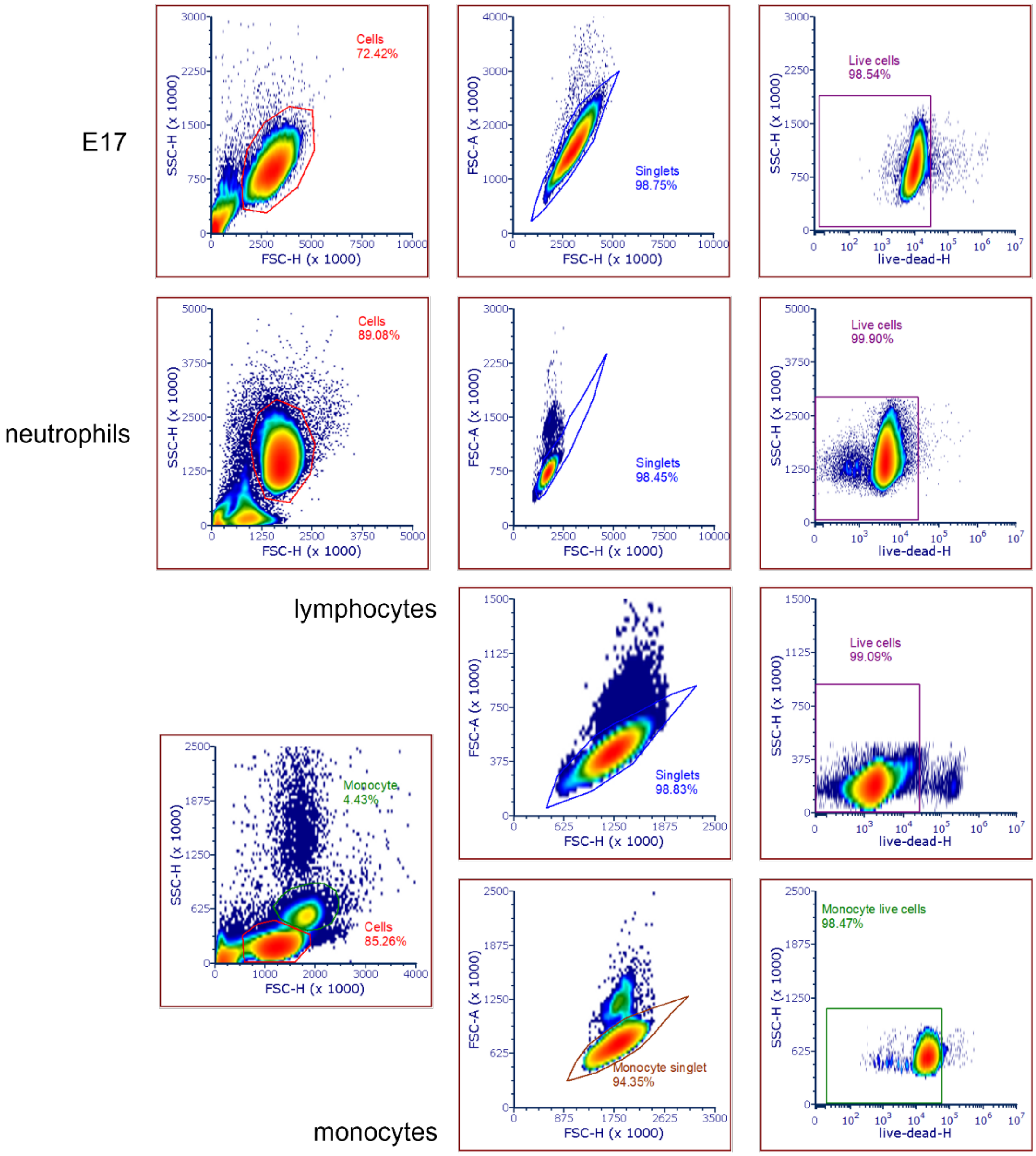

Figure S7. Flow cytometry gating strategy for Gal-SeNBD-oleamide in E17 cells and different human immune cells. Cells were gated first based on the forward and side scattering eliminating all debris and singlets were gated next based on forward scatter area versus height. Live cells were selected from the singlets based on live-dead staining (Zombie Violet, 1:100). The panels were acquired on FCS express 7 (De Novo).

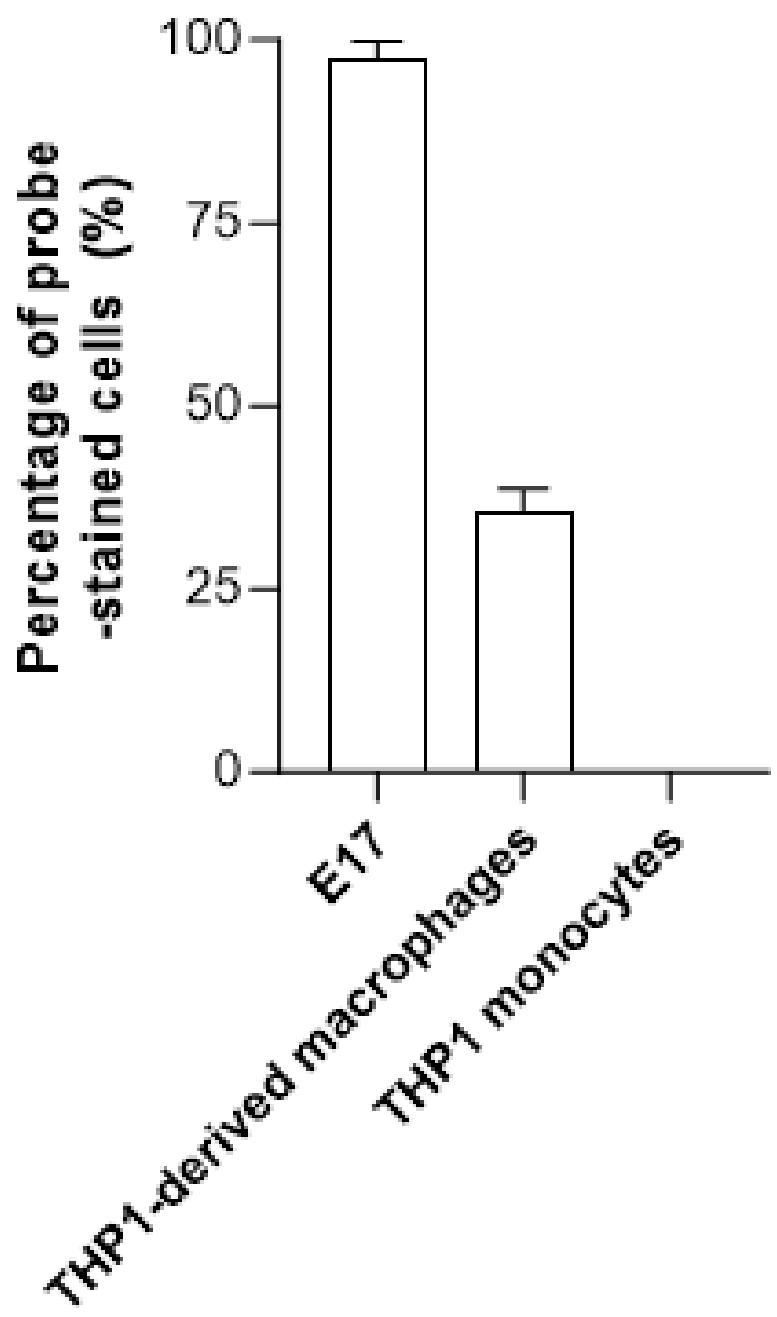

Figure S8. **Flow cytometry of E17 cells, THP1 monocytes and THP1-derived macrophages**. Flow cytometry analysis after incubation with **Gal-SeNBD-oleamide** (10 µM) for 60 min at r.t. Values as means±SD (n=3).

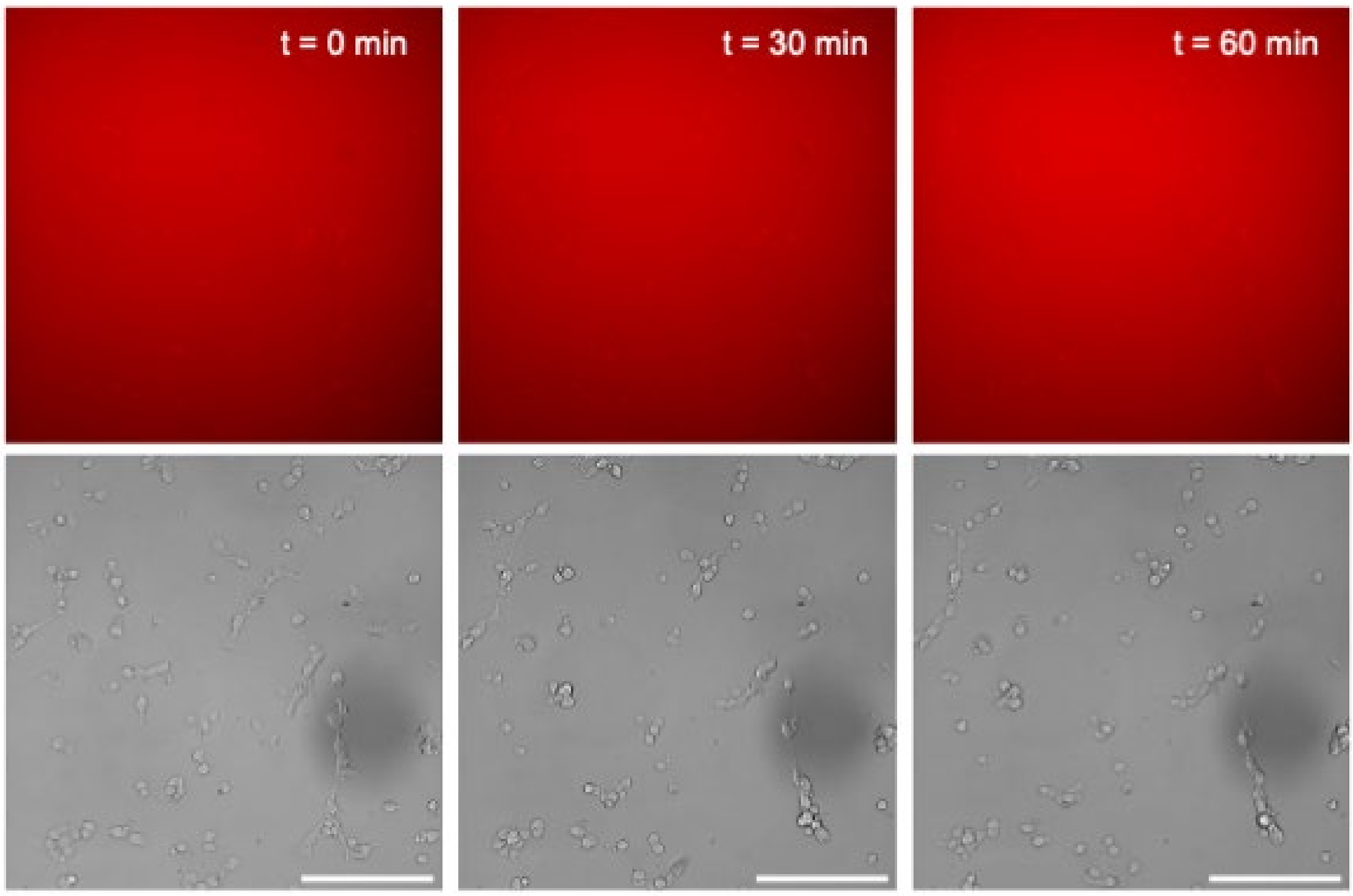

Figure S9. Fluorescence activation of control compound 27 (Gal-SeNBD-NH_2) in E17 cells. Representative wash-free brightfield and fluorescence microscopy images of human glioma cells during the incubation with compound **27** (10 µM) for 60 min at 37°C. Exc. 555 nm; em. 592±25 nm. Scale bars: 200 µm.

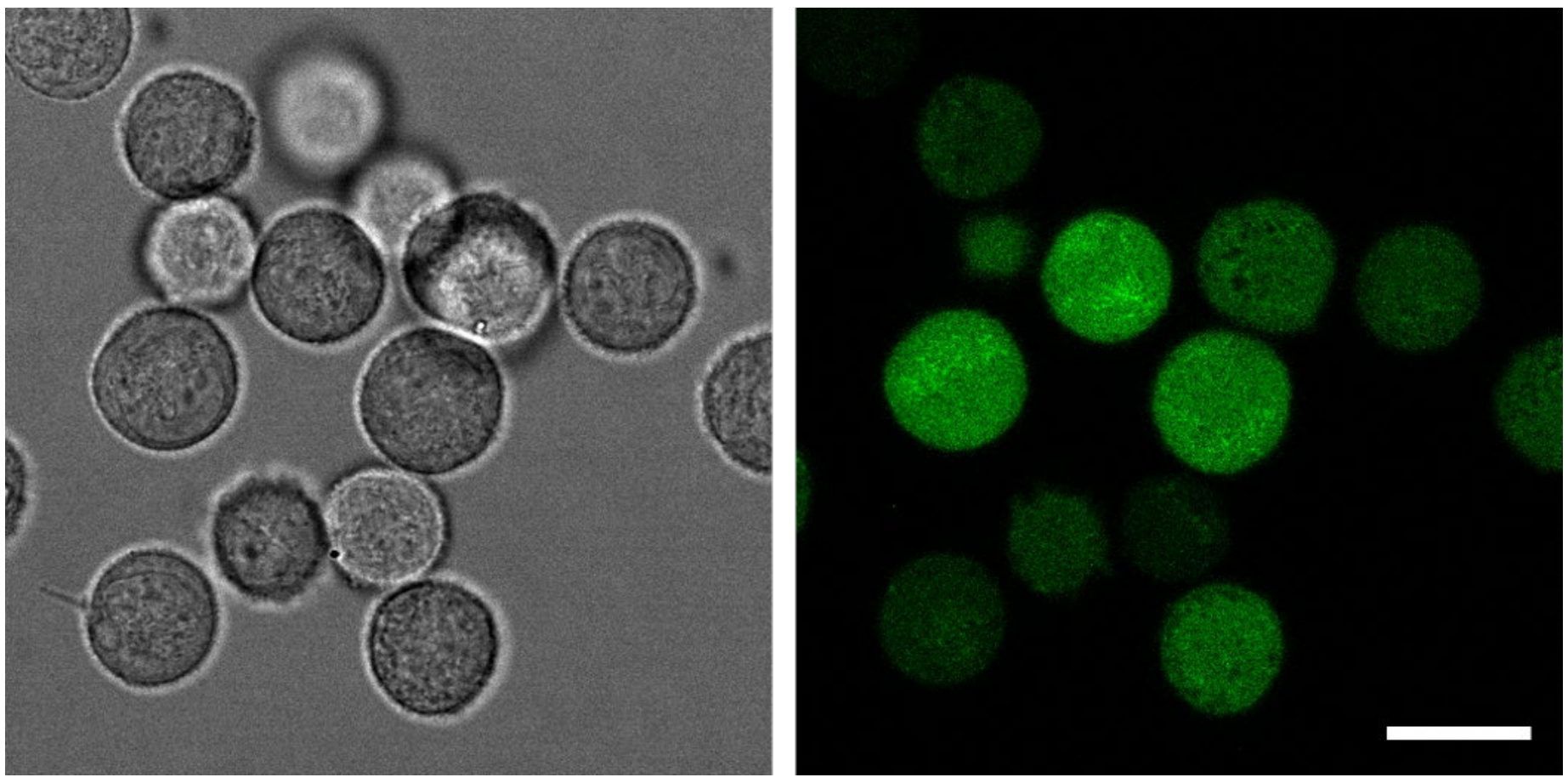

Figure S10. Fluorescence microscopy images of E17 cells after incubation with Gal-SeNBD-oleamide and DCFH-DA. Representative brightfield and fluorescence microscopy images of E17 cells after incubation with **Gal-SeNBD-oleamide** (10 μM) plus DCFH-DA (10 μM, 30 min at 37 °C) (right), followed by illumination (15 mW cm^{-2}, 520 nm, 60 min). Exc. 490 nm; em. 525±25 nm. Scale bar: 10 μm.

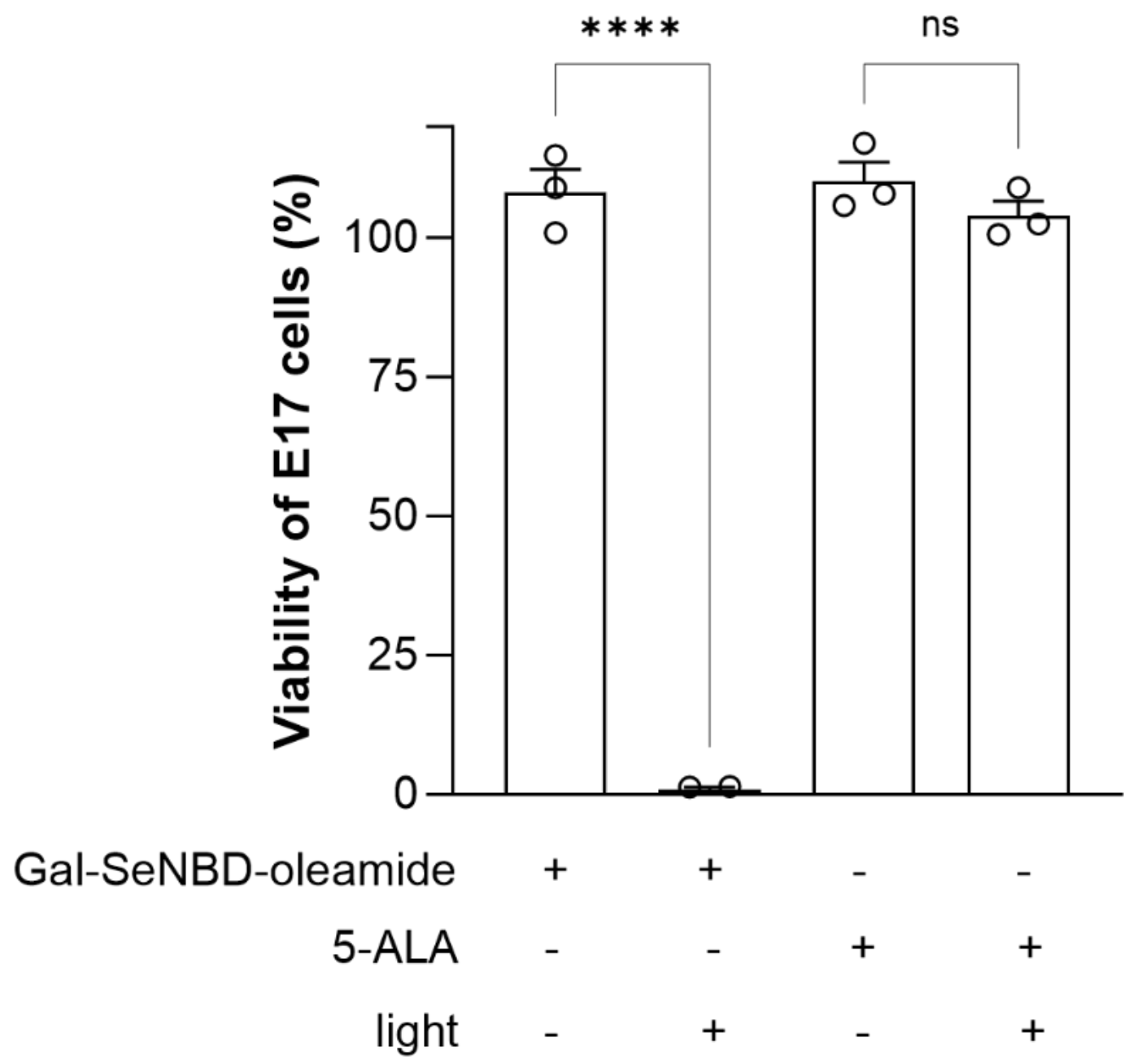

Figure S11. Phototoxicity assays in E17 cells after incubation with **Gal-SeNBD-oleamide** or **5-ALA** (both at 10 μM) for 60 min at 37 °C and absence/presence of light irradiation (15 mW cm^{-2}, 520 nm, 60 min). Values as means±SD (n=3). Statistical analysis using one-way ANOVA. **** for $p < 0.0001$, ns for $p > 0.05$.

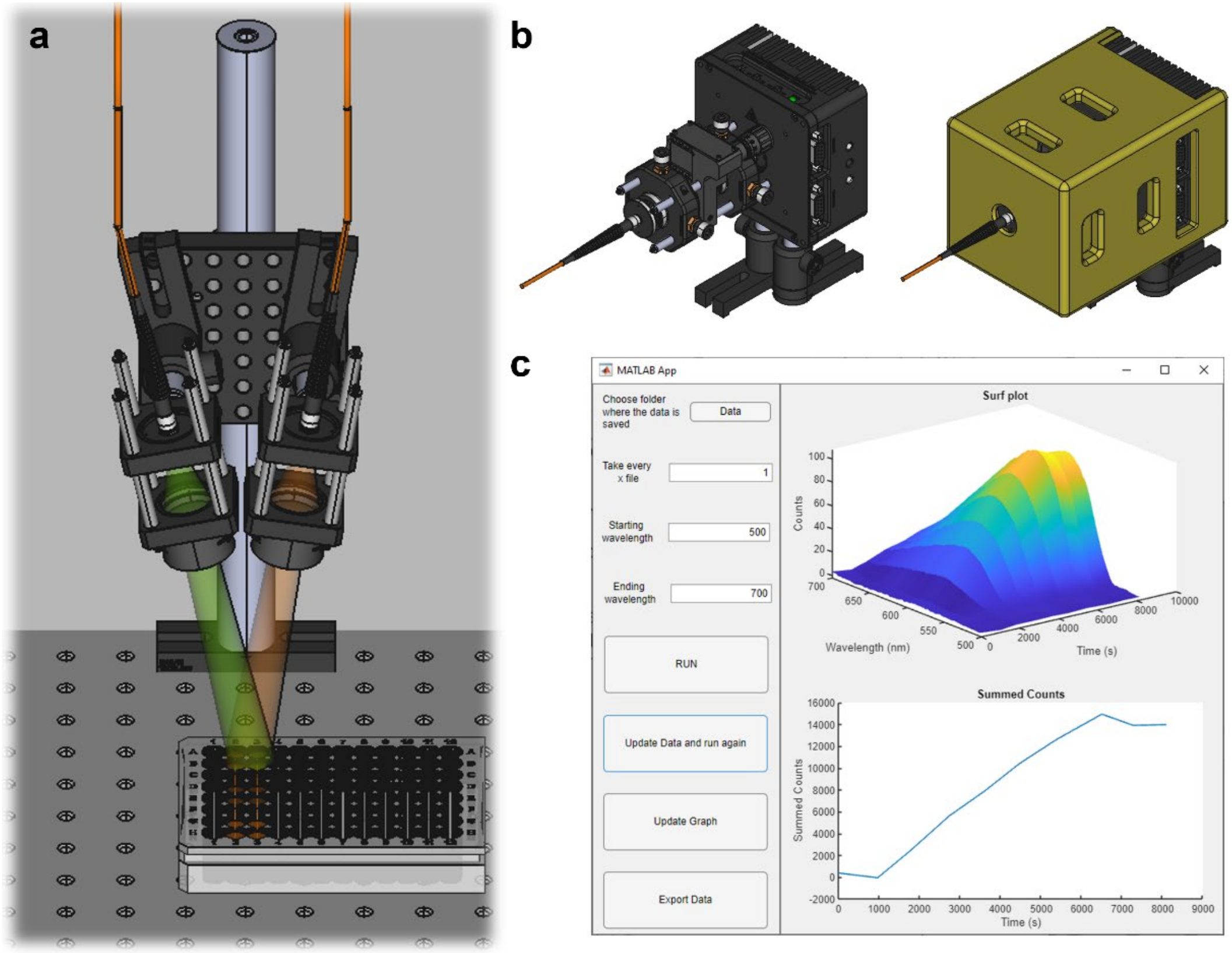

Figure S12. In-house illumination and spectroscopy system. a) Co-aligned illumination and collection utilizing standard optomechanical and construction components. The fibre-coupled illumination head is suitable for a medical enclosure and attachment to an articulated support arm. b) Laser diode coupling mechanics, also shown with cover for robust use in lab. Alignment components allow adjustment for interchangeability of the low-cost laser diode components. c) Automated data acquisition GUI (Matlab). Data collection is co-triggered by a physical button controlling an optical shutter (avoiding unwanted continuous illumination). GUI displays full spectral data and direct monitoring of PS activation.

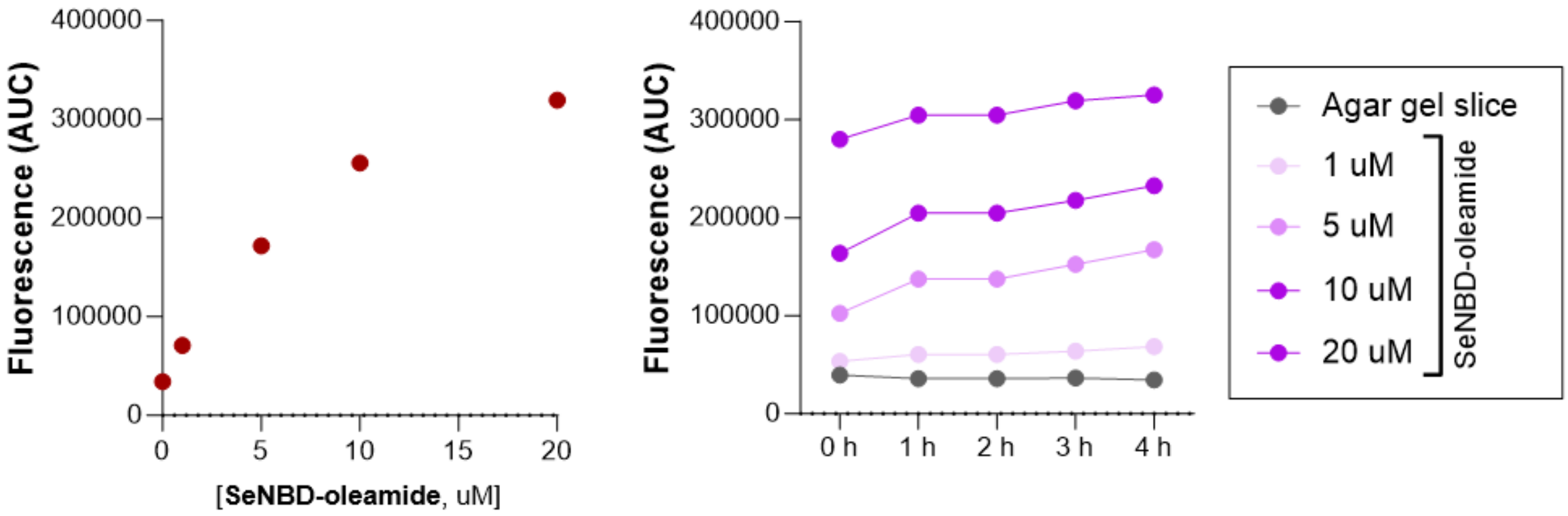

Figure S13. Fluorescence emission spectra of SeNBD-oleamide using in-house illuminator. Fluorescence signals of increasing concentrations of **SeNBD-oleamide** (0.4 μM to 20 μM) in tissue-mimicking phantoms and at different time points. LoD = 1.4 μM. Excitation at 520 nm, power 25 mW cm^{-2} and exposure times of 1 s.

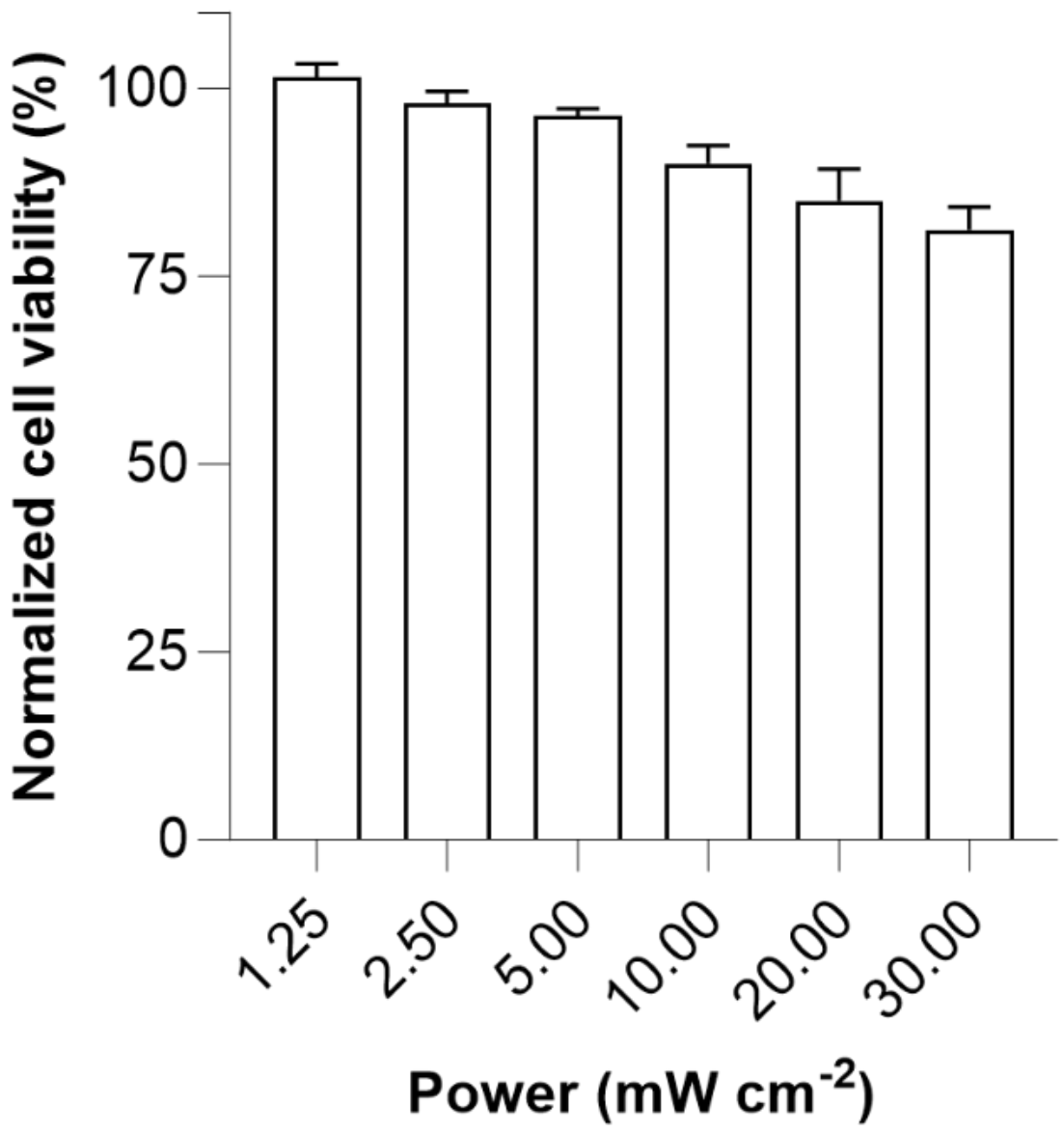

Figure S14. Cytotoxicity analysis for continuous light irradiation in human glioma cells. E17 cells (10,000 cells per well) were illuminated with light (520 nm, indicated power) continuously for 60 min. Cell viability was assessed using resazurin assays and calculated as a percentage of viable cells relative to cells in the dark. Values presented as means±SD (n=3).

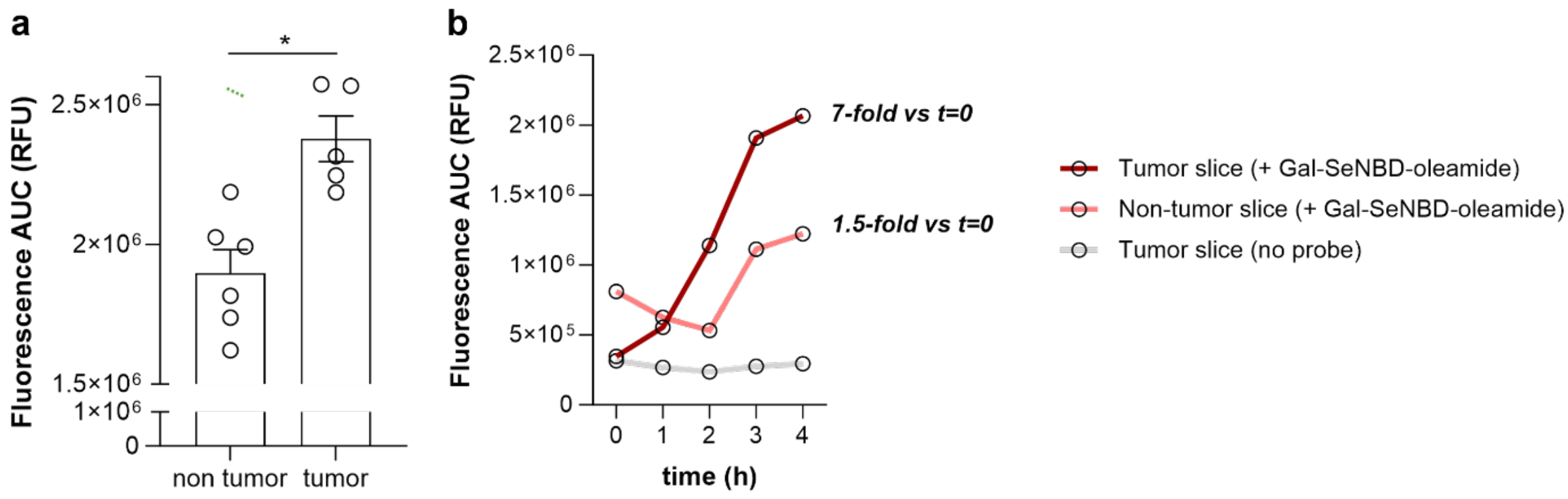

Figure S15. Fluorescence emission of mouse tissue slices using in-house illuminator. a) Mouse brain tissue slices were incubated with **Gal-SeNBD-oleamide** (20 μM) at t=0 and the fluorescence area under the curve (AUC, exc. 520 nm) were determined after 4 hours using the in-house illuminator. Values presented as means±SD (n=5 from 5 mice). P values were determined two-tailed unpaired t test (* for $p<0.05$). b) Representative kinetics of **Gal-SeNBD-oleamide** activation in tumor vs non-tumor slices.

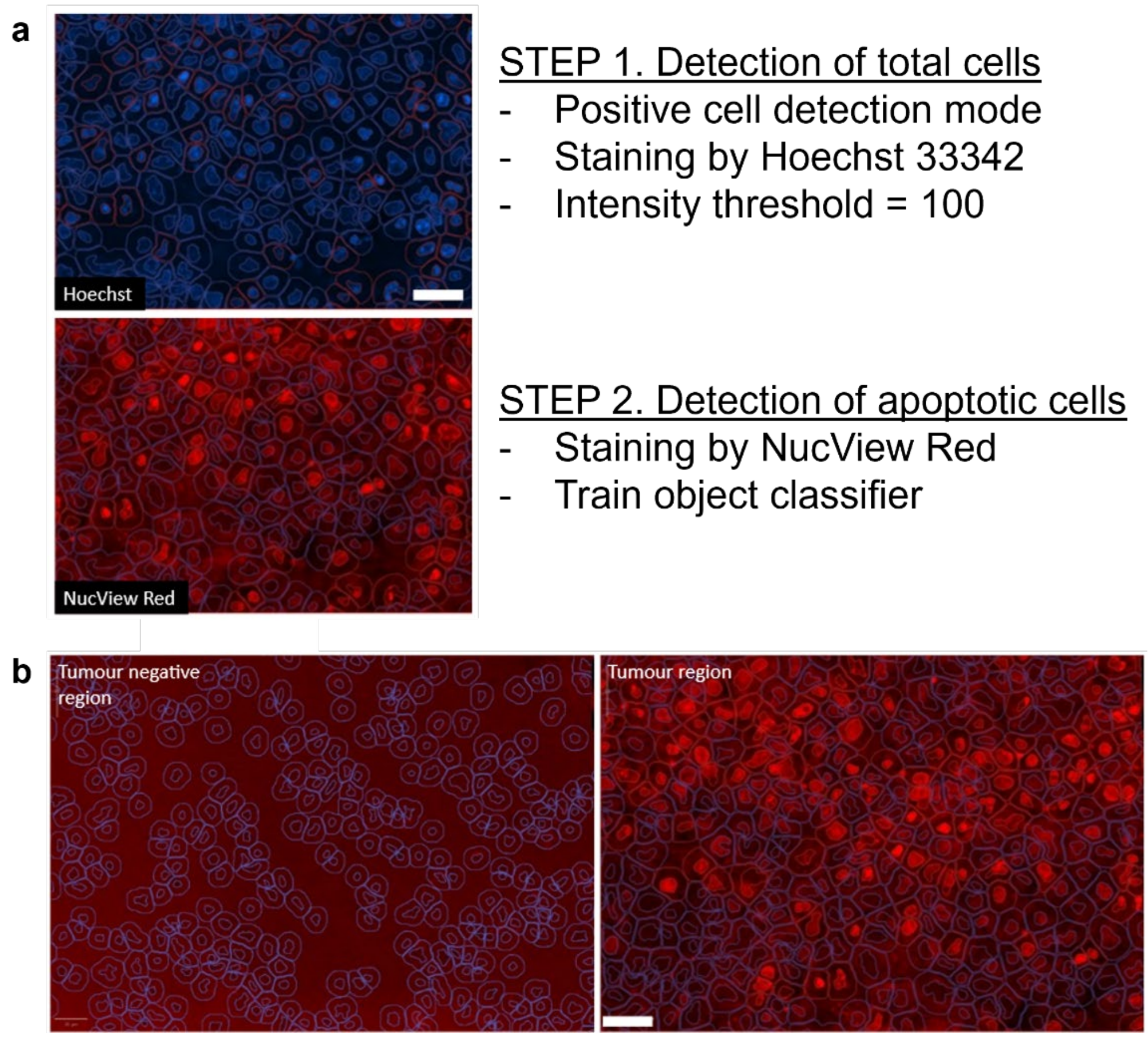

Figure S16. Image data analysis of mouse brain tissue slices. Image analysis strategy using QuPath (version 0.5.1) with approx. 30,000 cells being analyzed per annotation. a) Panels showing positive cell detection followed by object classifier training using a random tree classifier, which was applied to all the images to quantify the number of NucView Red-stained apoptotic cells. b) Representative panels showing NucView Red-unstained cells in non tumor slices and NucView Red-stained cells in tumor slices. Scale bars: 20 µm.

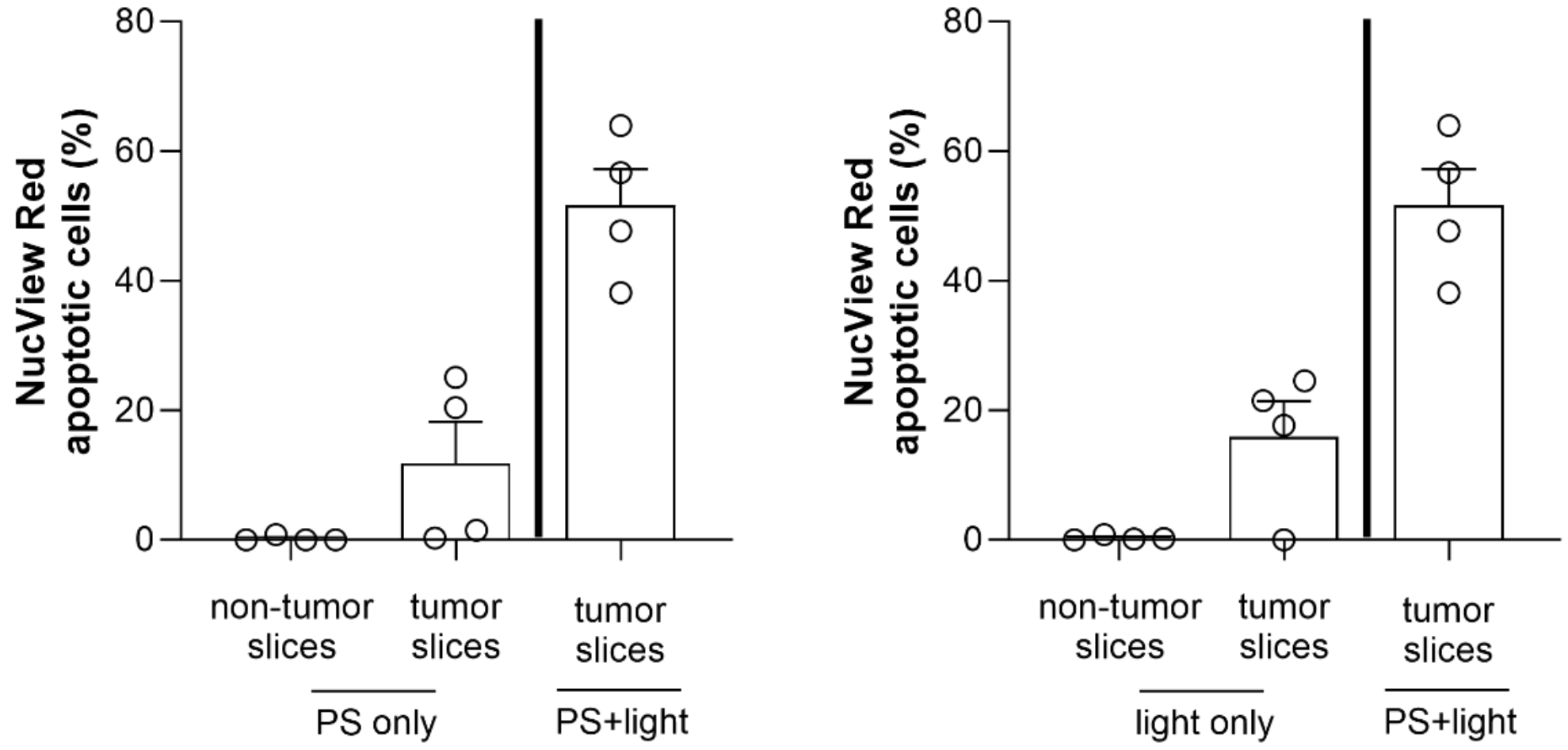

Figure S17. NucView Red quantification in control mouse brain slices. Mouse brain tissue slices were incubated with **Gal-SeNBD-oleamide** (20 μM) and/or only illuminated with or without PS (25 mW cm^{-2}, 520 nm, 60 min). Values presented as means±SEM (n=4).

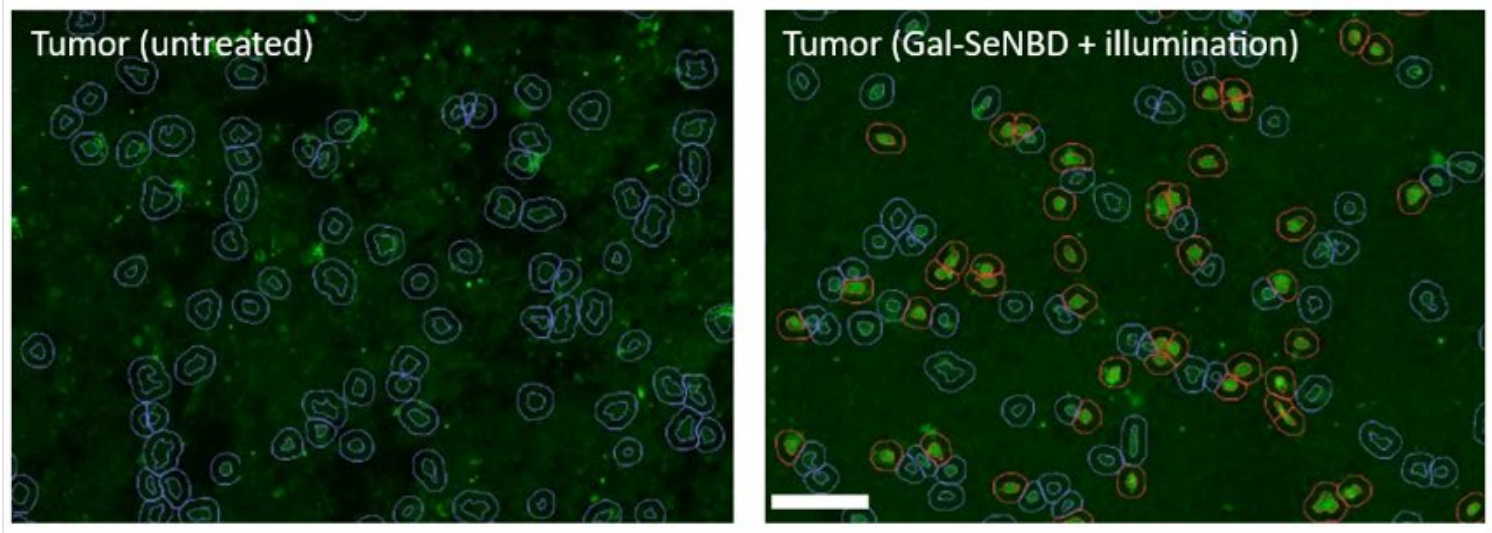

Percentage of NucView Green-stained cells	**1%**	**22%**
Total number of cells analyzed per slice	5,359	5,222

Figure S18. The PDT treatment with Gal-SeNBD-oleamide PDT causes apoptosis in human tumor tissues from brain cancer patients. Tumor slices were incubated with or without **Gal-SeNBD-oleamide** (20 μM) followed by illumination or not (520 nm, 25 mW cm^{-2}, 60 min). NucView Green-stained apoptotic cells were imaged using an Opera Phenix microscope (exc. 488 nm, em. 520 nm, 40×) and quantified using QuPath (version 0.5.1). Scale bar: 50 μm.

Table S1. Photophysical properties of SeNBD PS conjugates. Table summarizing molar extinction coefficients (ε), fluorescent quantum yields (Φf) and calculated partition coefficients (cLogP). Relative Φf values were obtained using fluorescein as the reference.

SeNBD PS conjugates	metabolite/ligand	ε (M^{-1} cm^{-1})	Φf (%)	clogP
**1**	lactate	8,100	2	-1.84
**2**	triphenylphosphine	14,200	3	4.61
**3**	linoleic acid	12,000	5	7.91
**4**	oleic acid	7,600	3	8.49
**5**	lauric acid	12,700	2	6.09
**6**	butyric acid	14,600	1	2.05
**7**	amine (primary)	2,500	1	1.13
**8**	diethylamine	7,500	<1	2.51
**9**	propylamine	13,200	2	2.39
**10**	piperidine	n.d.	n.d.	2.66
**11**	cyclohexyl-N-ethylamine	11,600	<1	4.04
**12**	ododiethylamine	8,500	<1	3.52
**13**	γ-aminobutyric acid	13,700	3	1.30
**14**	choline	16,600	2	-3.07
**15**	acetylcholine	6,400	2	-2.37
**16**	putrescine	10,300	2	0.85
**17**	spermidine	22,500	3	0.56
**18**	spermine	n.d.	n.d.	0.27
**19**	glycine	9,700	3	-0.16
**20**	phenylalanine	1,600	<1	0.47
**21**	alanine	4,500	4	-1.84
**22**	D-alanine	9,900	3	-1.84
**23**	Ahx-phenylalanine	7,600	3	0.70

Table S2. Specifications and sources of the instrument parts used to build on-the-fly illumination and spectroscopy device.

Component	Specification / Function	Source (Manufacturer, Model)
Laser diode (520 nm)	50 mW available power; TO-can packaging (~5.6 mm diameter); matches PS excitation peak	Thorlabs, L520P50
Aspheric lens pair	Coupling diode emission into optical fibre (70% efficiency)	Thorlabs, A220TM-A
Achromatic doublet lenses	30 mm focal length; reimage fibre facets onto sample	Thorlabs, AC254-030-A-ML
Band-pass filter (illumination path)	Removal of unwanted long-wavelength emission from diode	Thorlabs, FBH520-40
Long-pass filter (collection path)	Removal of unwanted illumination laser signal	Thorlabs, FELH0550
Spectrometer	Monitoring and recording PS fluorescence signal	Ocean Optics, QEPro
Electrically actuated shutter	Placed after fibre output, before clean-up filter; control of illumination	Thorlabs, SHBH1T

Table S3. NucView-positive (apoptotic) cells and GFP-positive glioma cells versus across annotated tumor regions. Total number and percentages of apoptosis in annotated tumor regions as well as in GFP-positive cells following PDT treatment with **Gal-SeNBD-oleamide** (data from four independent mouse brain tissue experiments).

Experiment	Total cells in annotated regions	Dead cells in annotated regions	% NucView +ve cells in tumors	GFP+ve cells in annotated regions	Dead cells among GFP+ve cells	% NucView +ve cells (GFP+ve, tumor region)
1	20,197	9,628	47%	19,228	9,124	47%
2	21,731	12,317	57%	20,026	11,231	56%
3	3,649	1,393	38%	3,142	1,240	39%
4	29,792	19,065	64%	24,612	15,670	66%

Table S4. Summary of therapeutic strategies for glioblastoma.

Strategy	Selectivity	Activation	λ_{exc} (nm)	Real-time monitoring	Validation	Limitations
Surgery + radiotherapy+ temozolomide (standard of care) (ref 3 & 5)	Anatomical (surgical) + proliferation	n.a.	n.a.	n.a.	Clinical (standard of care)	Residual infiltrating cells remain
5-ALA / PpIX PDT (ref 11 & 12)	Metabolic (heme pathway) accumulation	Formation of PpIX + light	635 nm (PDT) 405 nm (fluorescence)	No controlled /quantitative release monitoring	Clinical (approved for fluorescence-guided resection)	Heterogeneous PpIX accumulation
Non-activatable organic PS (e.g. porphyrin/chlorin-type) (ref 8)	Passive accumulation (EPR-type)	Light	Red or NIR range	No	Varies (cell → in vivo)	Dark toxicity risk and limited tumor selectivity
Other β-galactosidase-activatable PDT agents (ref 42)	Enzymatic (β-galactosidase)	β-galactosidase + light	580 nm	No	Glioblastoma cells (in vitro)	Validation reported at cellular leve
Enzyme-activatable hemicyanine PS (ref 20)	Enzymatic	Cathepsin-B +light	720 nm	No	In vivo PDT in zebrafish model	Not proven in glioma brain tissue
**Gal-SeNBD-oleamide**	Oleamide-driven glioma uptake + enzymatic (β-galactosidase, GLB1↑)	β-galactosidase-triggered self-immolative uncaging + light	520 nm	Yes. On-the-fly spectroscopy + PDT dosimetry	Patient-derived cells, orthotopic mouse tissue, and resected human glioblastoma tissue (ex vivo)	Green excitation (limited depth)

4. Supplementary Movie Legends

Movie S1. Time-lapse fluorescence confocal microscopy images of E17 cells after incubation with **Gal-SeNBD-oleamide** (10 μM) (red). **Gal-SeNBD-oleamide** was added to the cells, and fluorescence images were immediately recorded. Exc. 555 nm; em. 592±25 nm. Scale bars: 200 μm. The movie was compressed (jpeg) at 10 fps.

5. NMR Spectra

(2) ^{1}H NMR (500 MHz, D_2O)

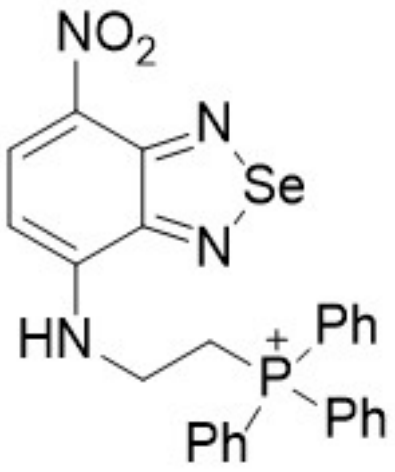

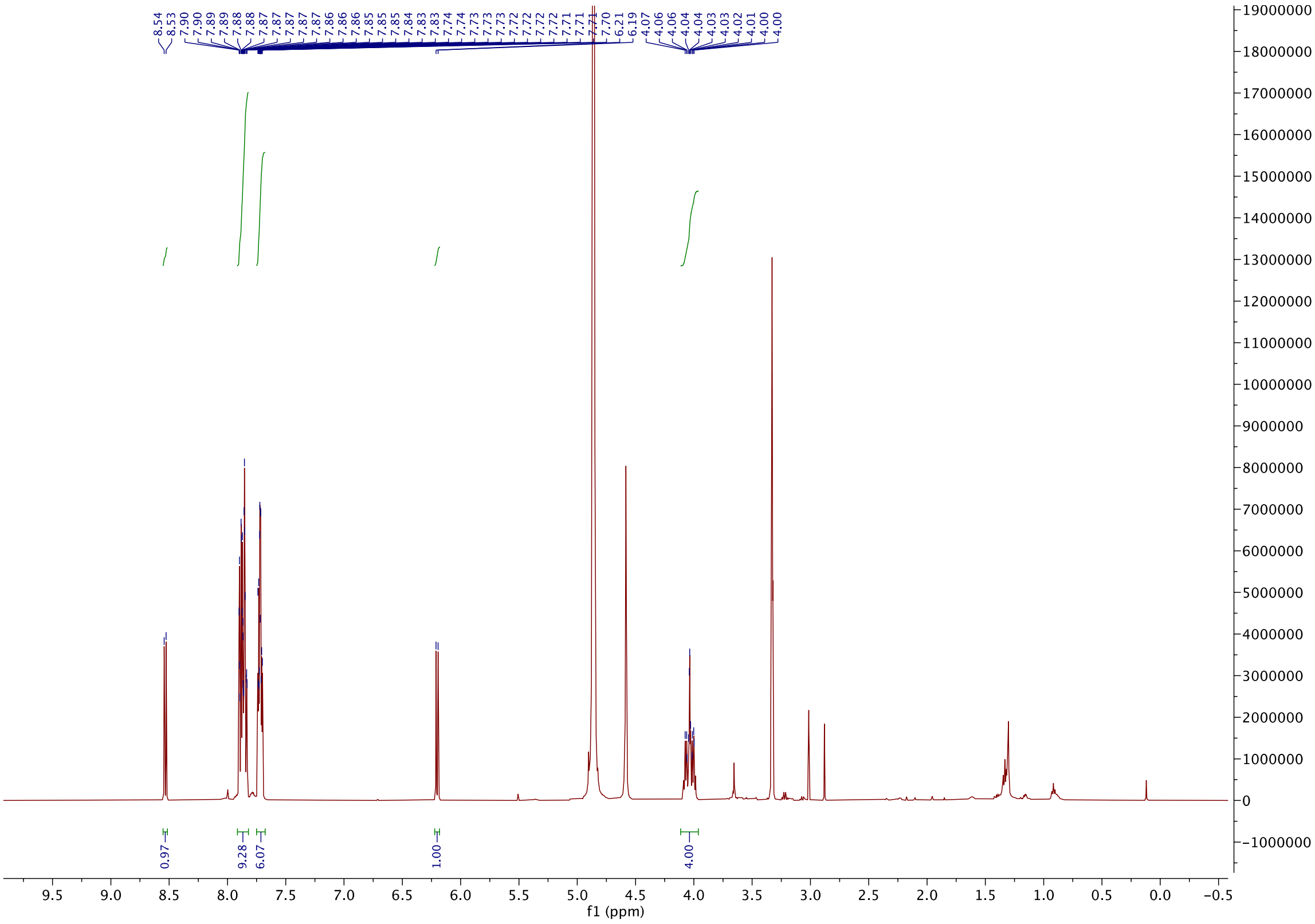

(2) ^{13}C NMR (126 MHz, CD_3OD)

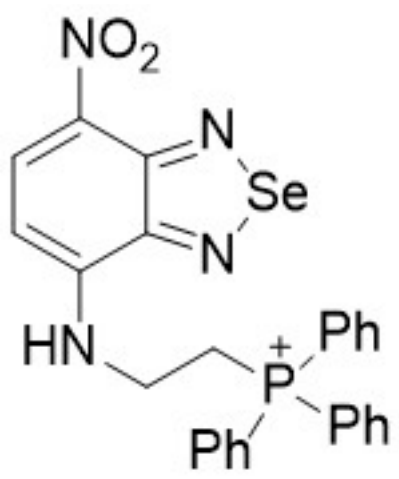

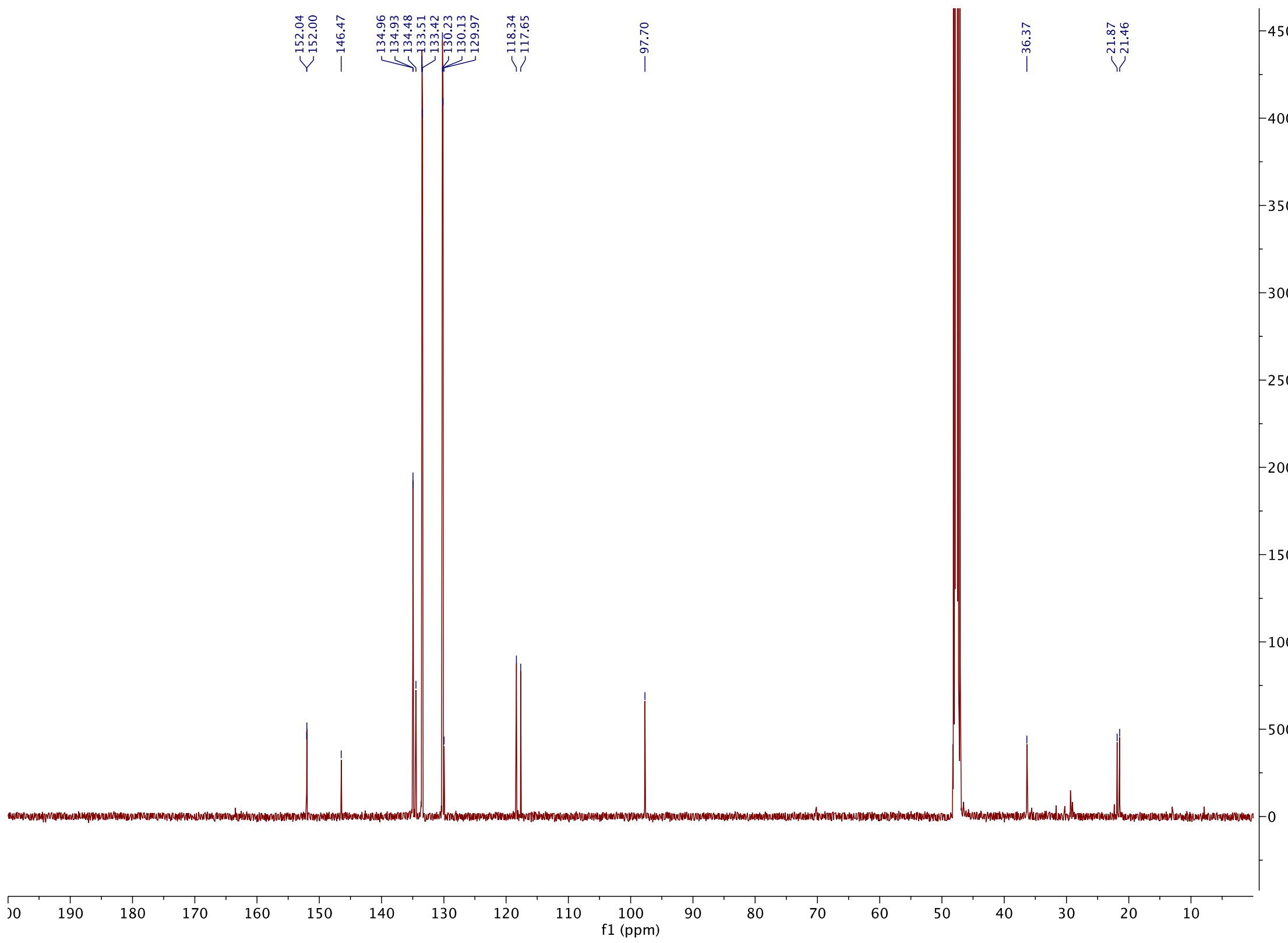

(3.1) ^1H NMR (500 MHz, DMSO-d_6)

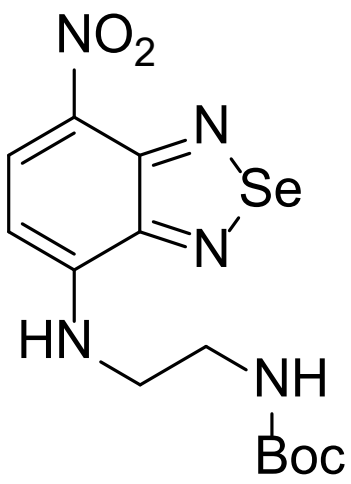

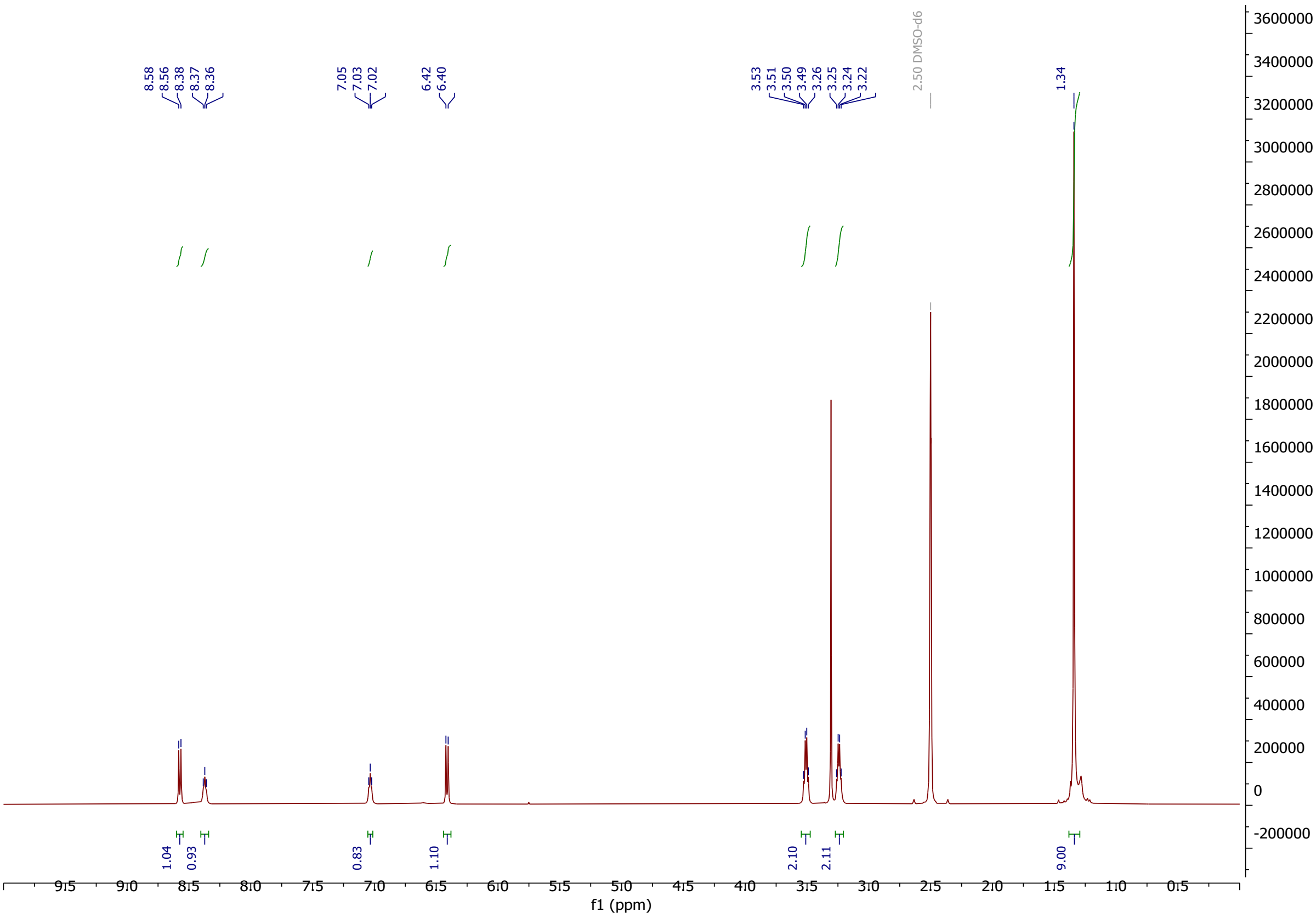

(3.1) ^{13}C NMR (126 MHz, DMSO-d$_6$)

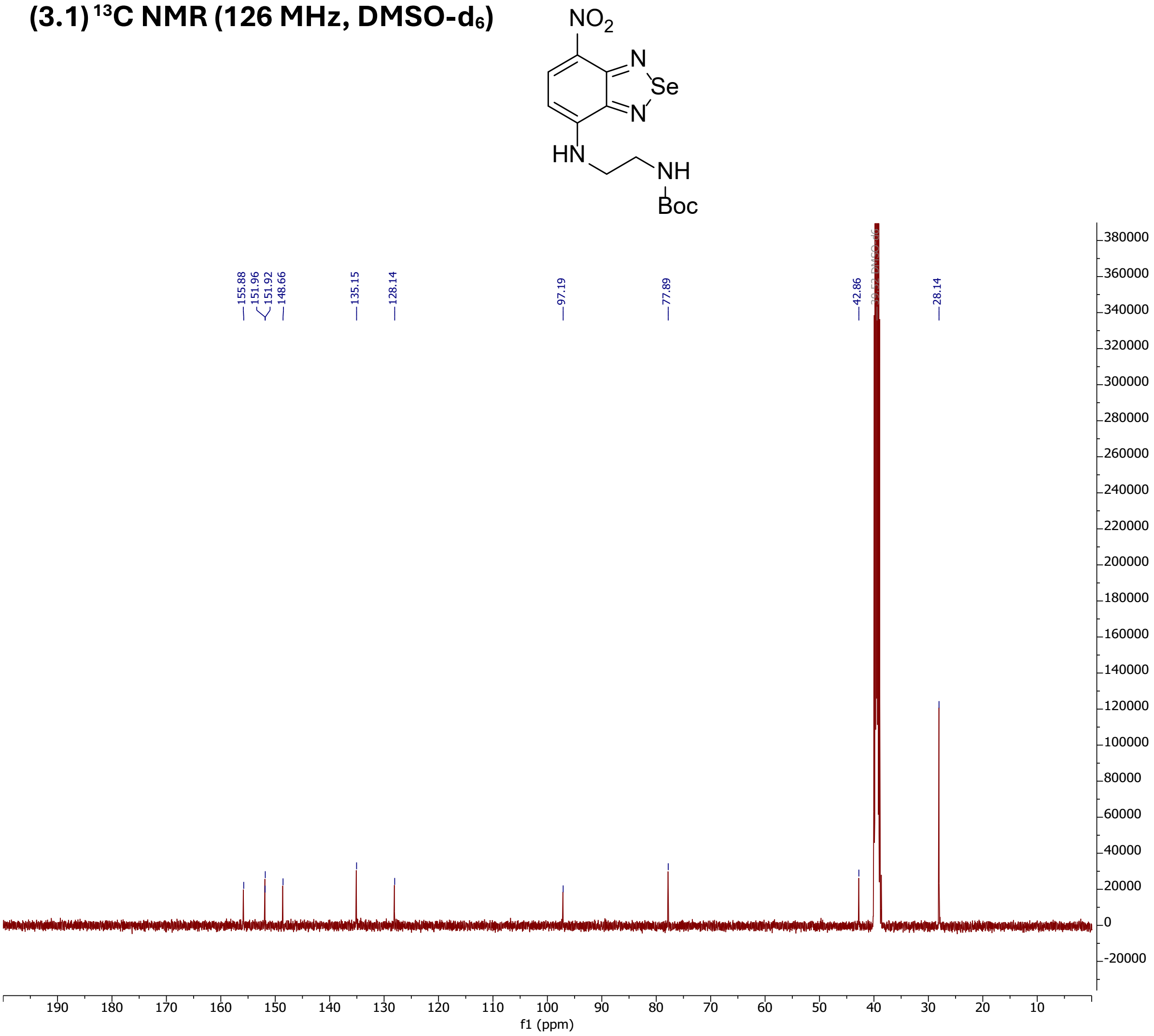

(3) ^{1}H NMR (500 MHz, $CDCl_3$)

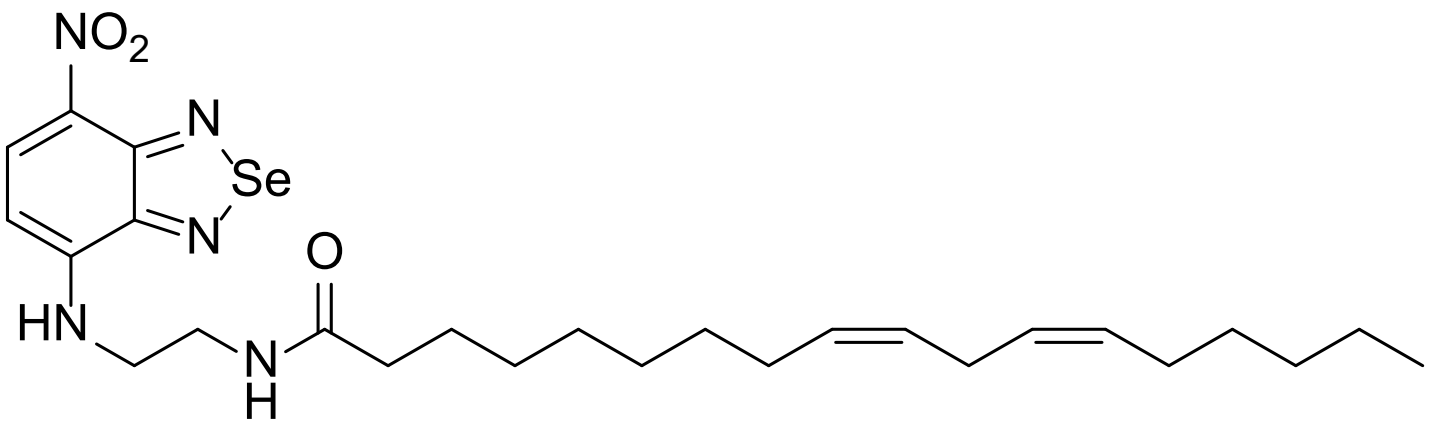

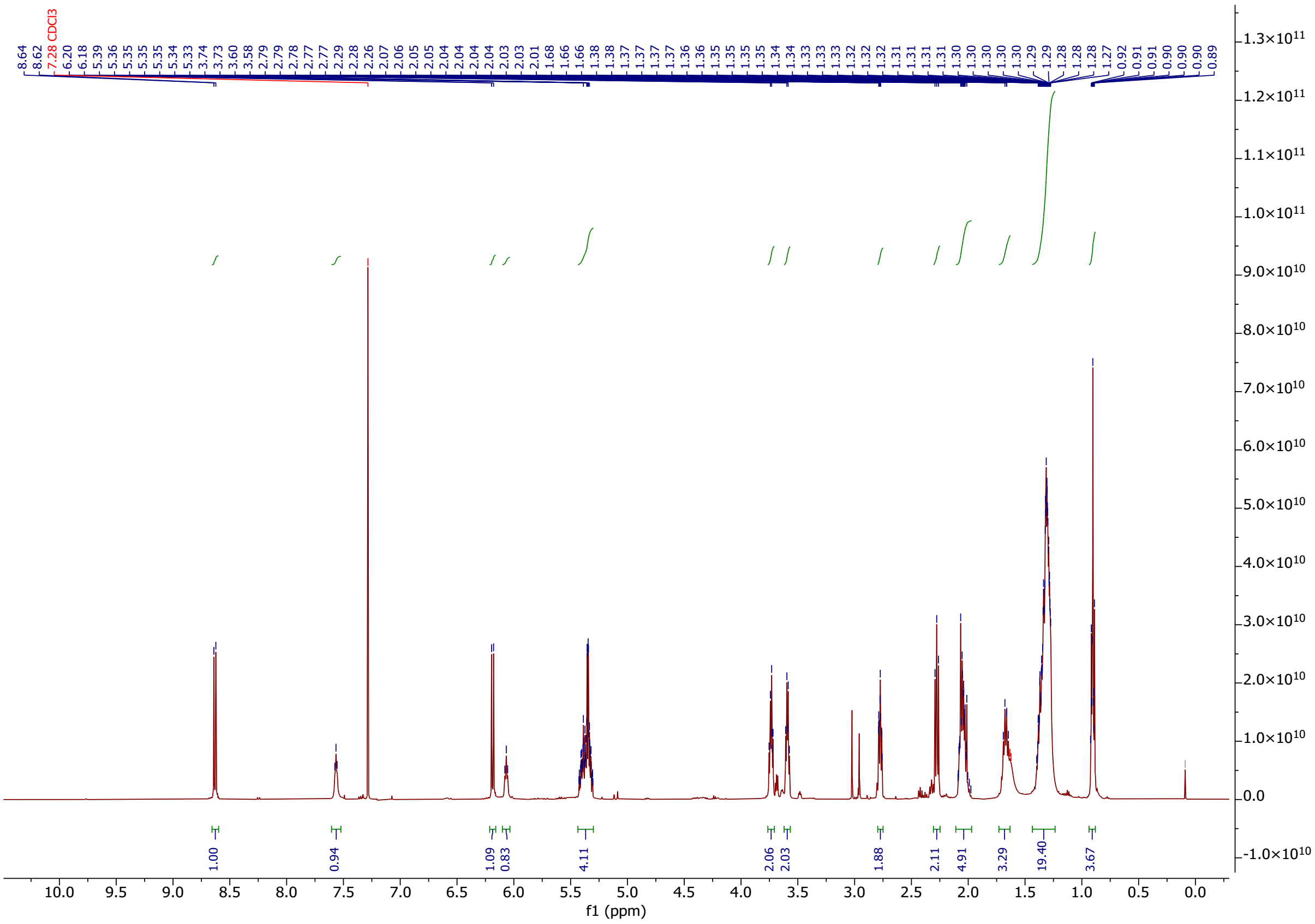

(3) ^{13}C NMR (126 MHz, $CDCl_3$)

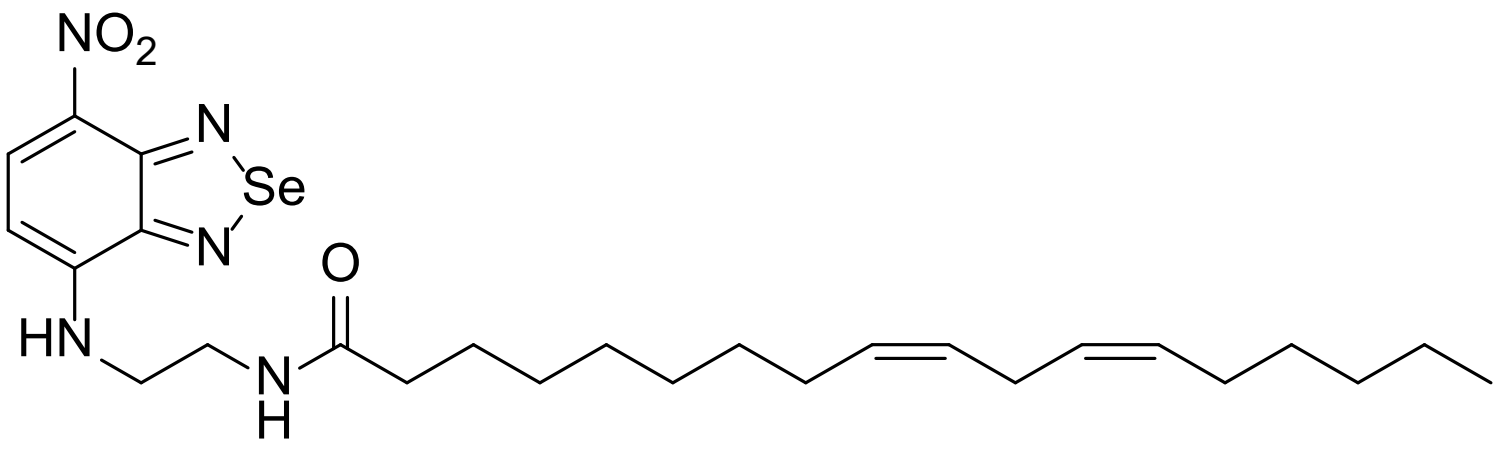

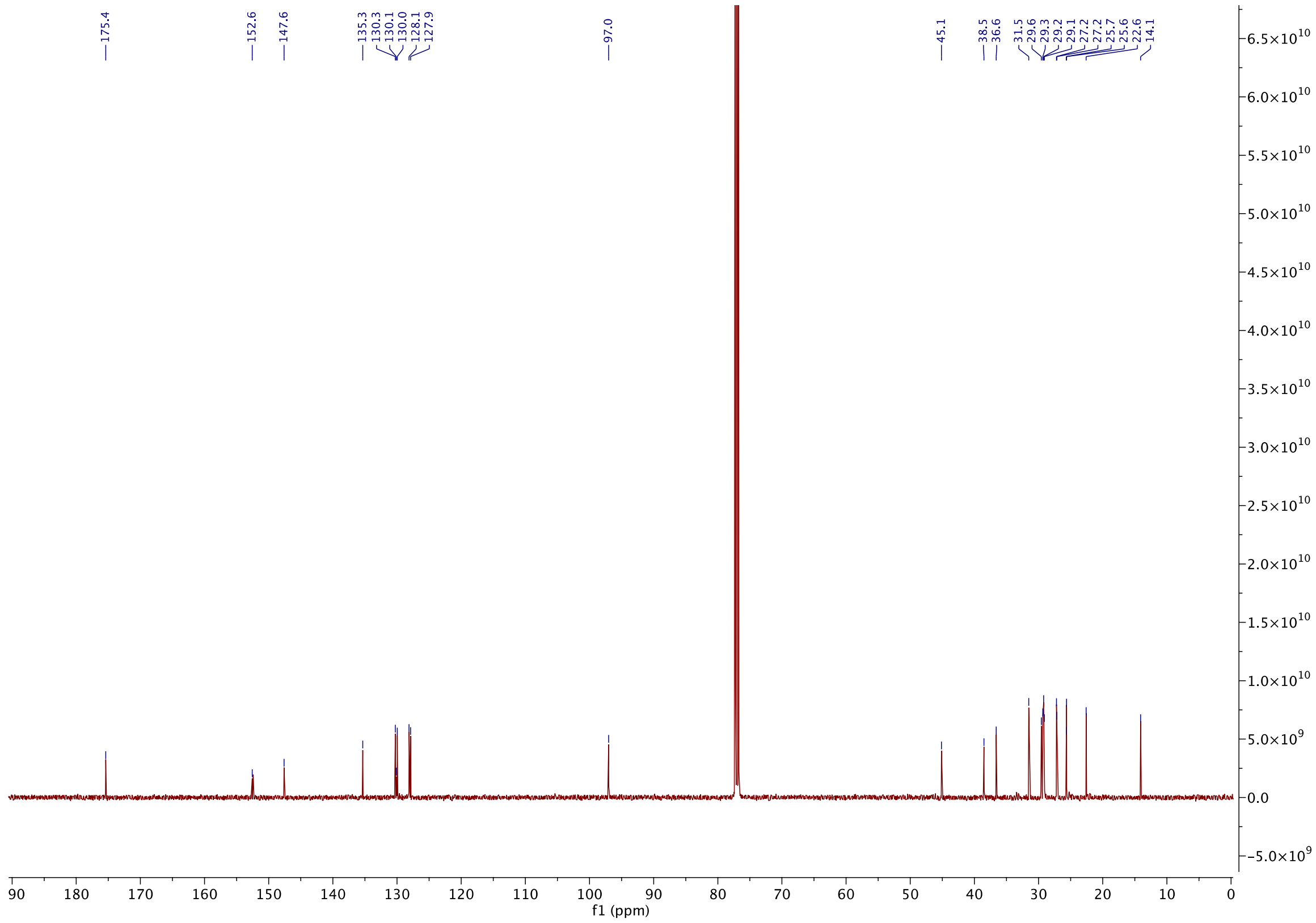

(4) ^{1}H NMR (500 MHz, DMSO-d_6)

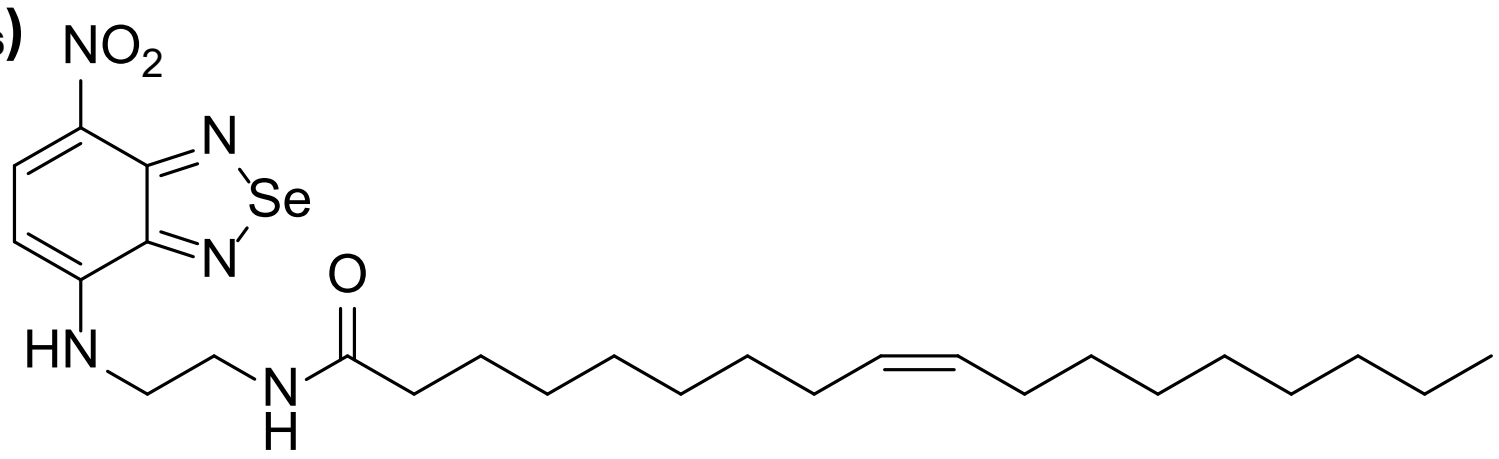

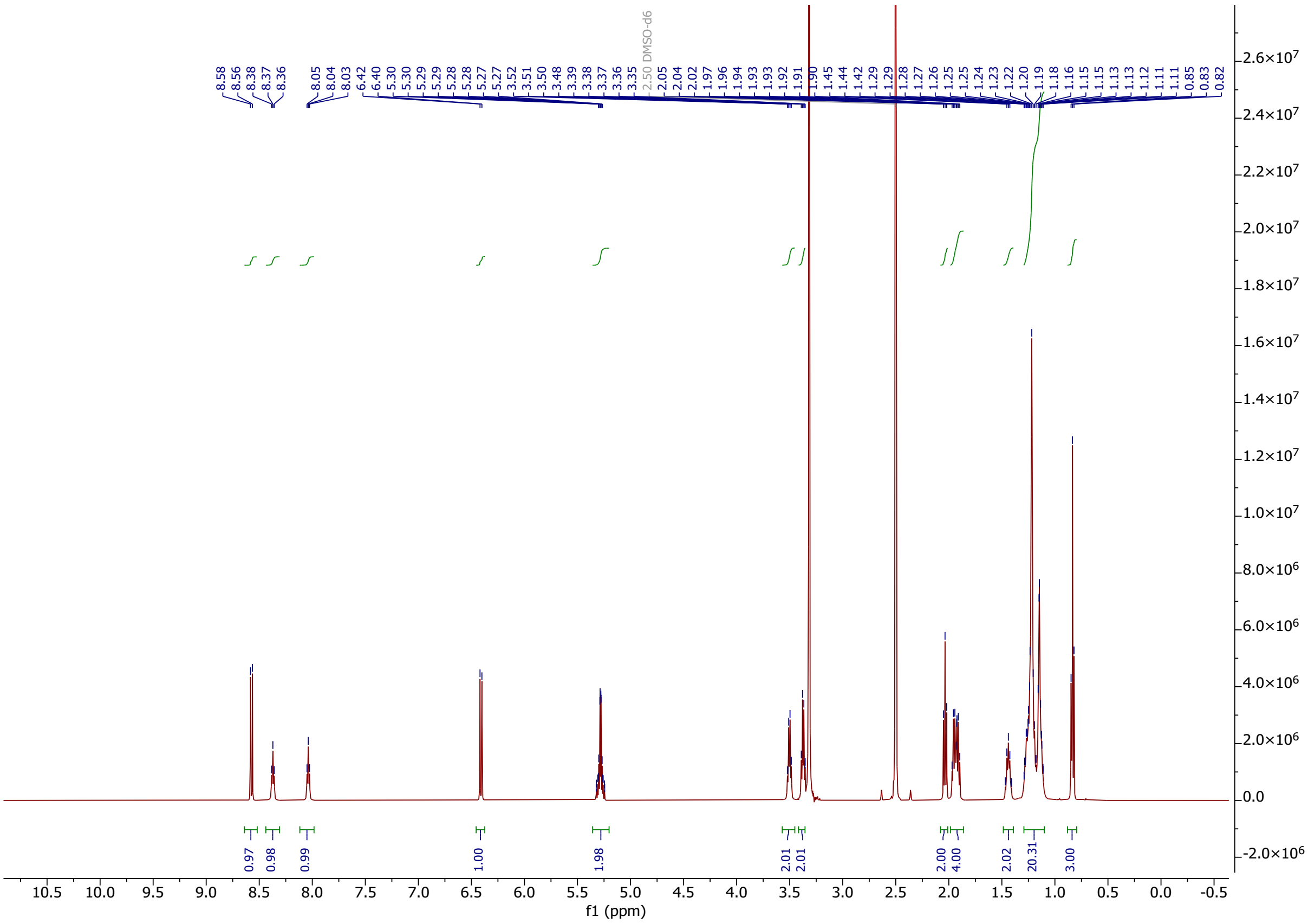

(4) ^{13}C NMR (126 MHz, DMSO-d$_6$)

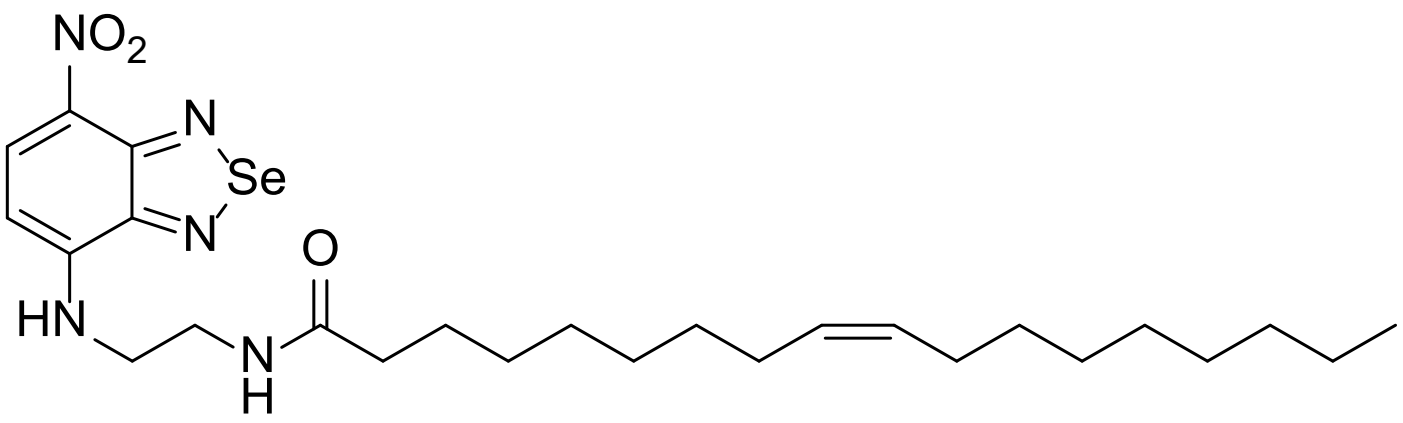

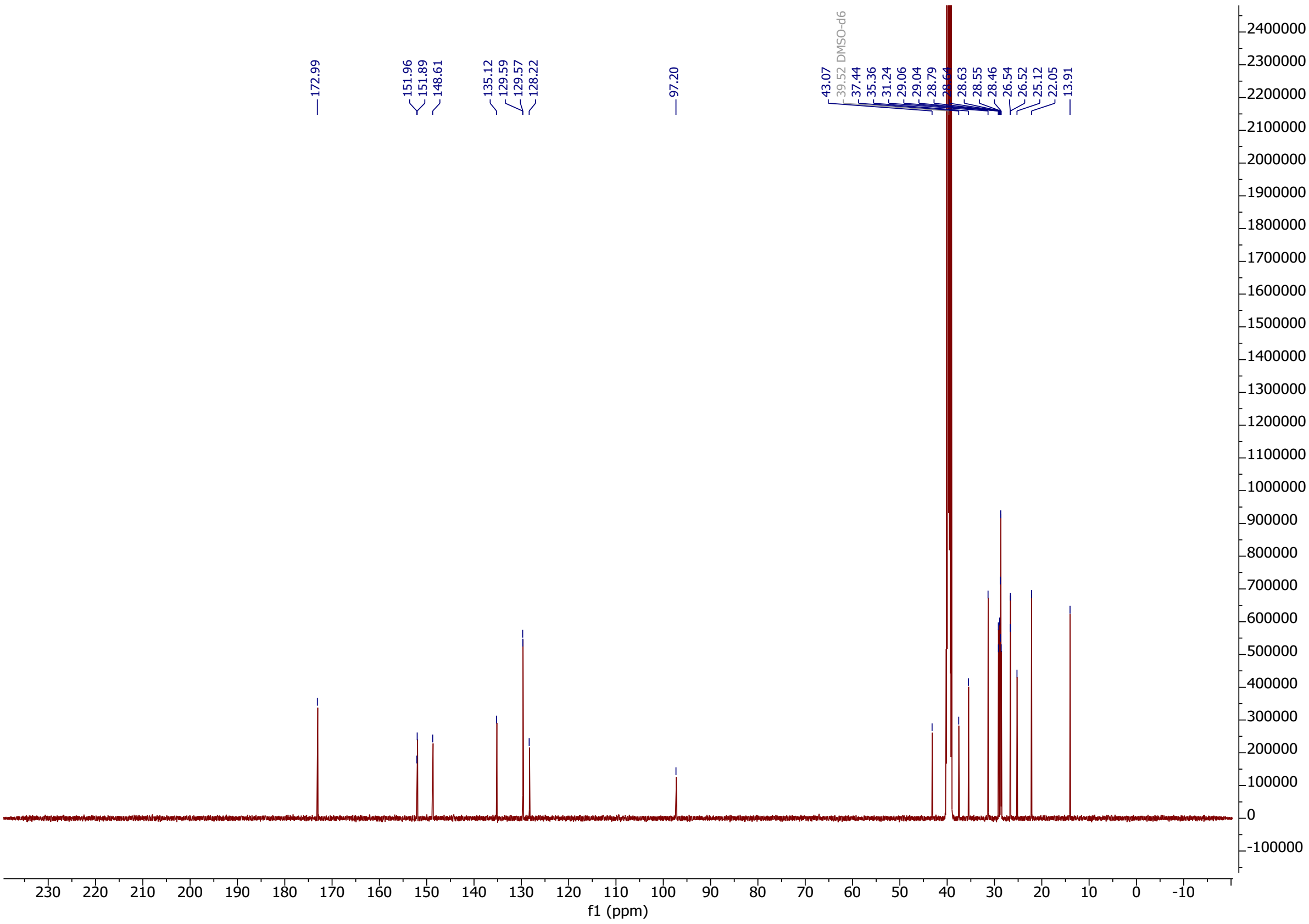

(5) ^{1}H NMR (500 MHz, DMSO-d_6)

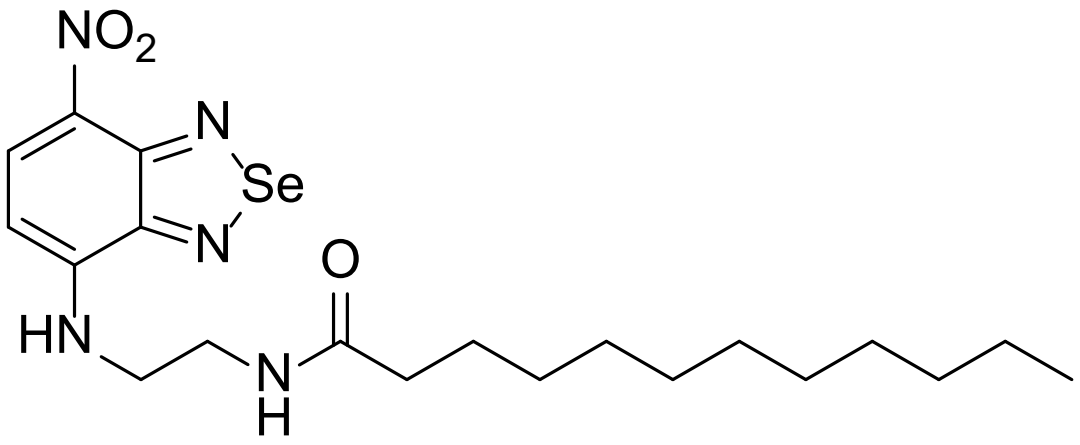

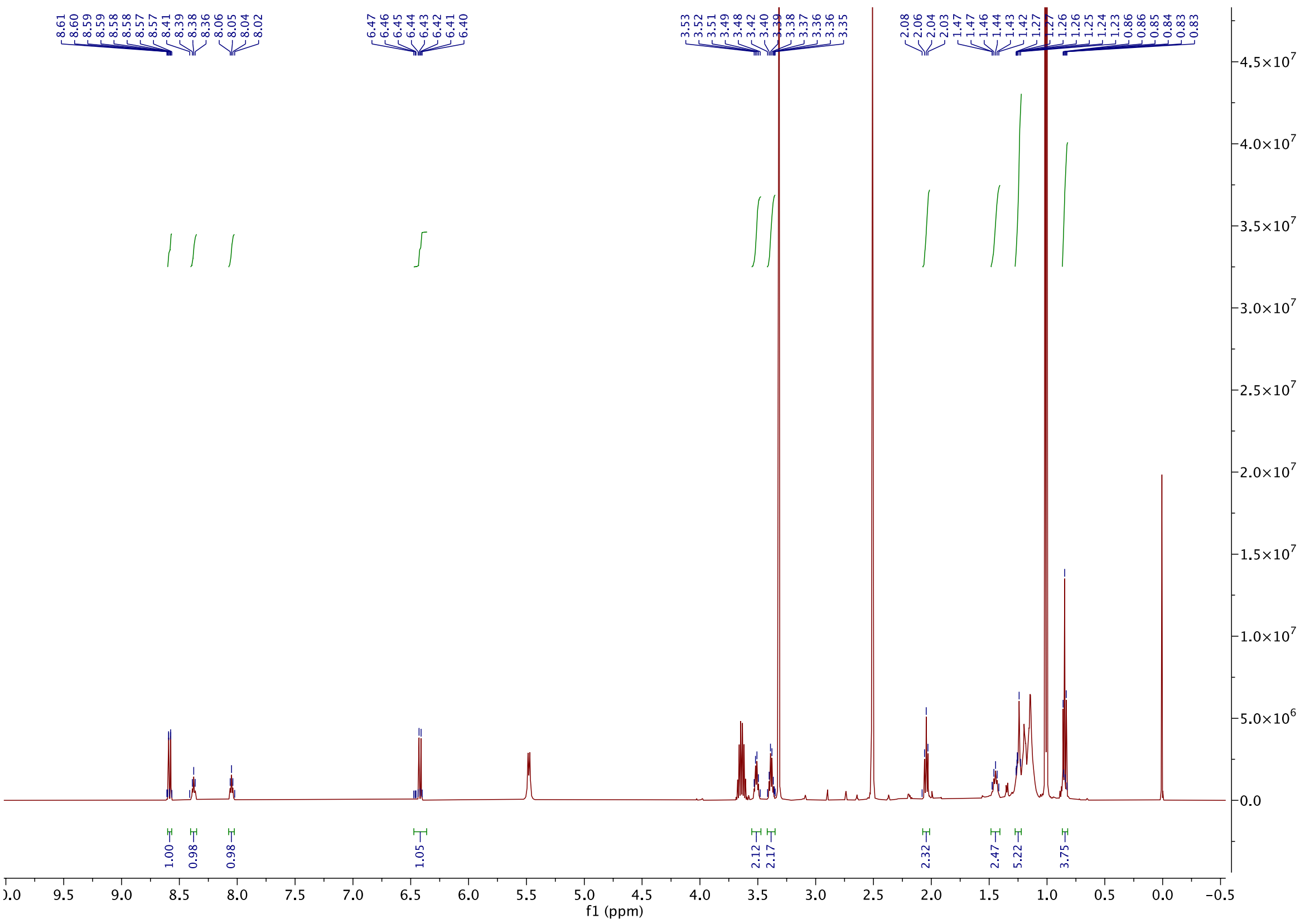

(5) ^{13}C NMR (126 MHz, DMSO-d$_6$)

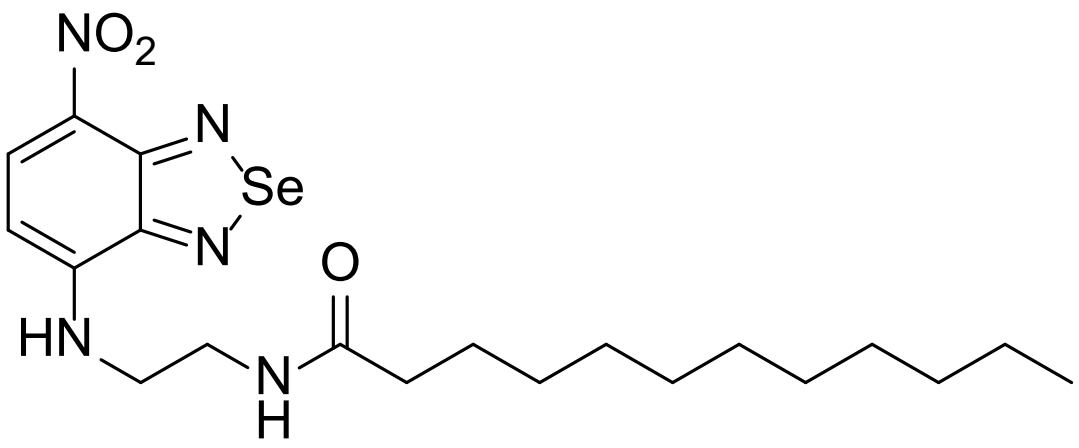

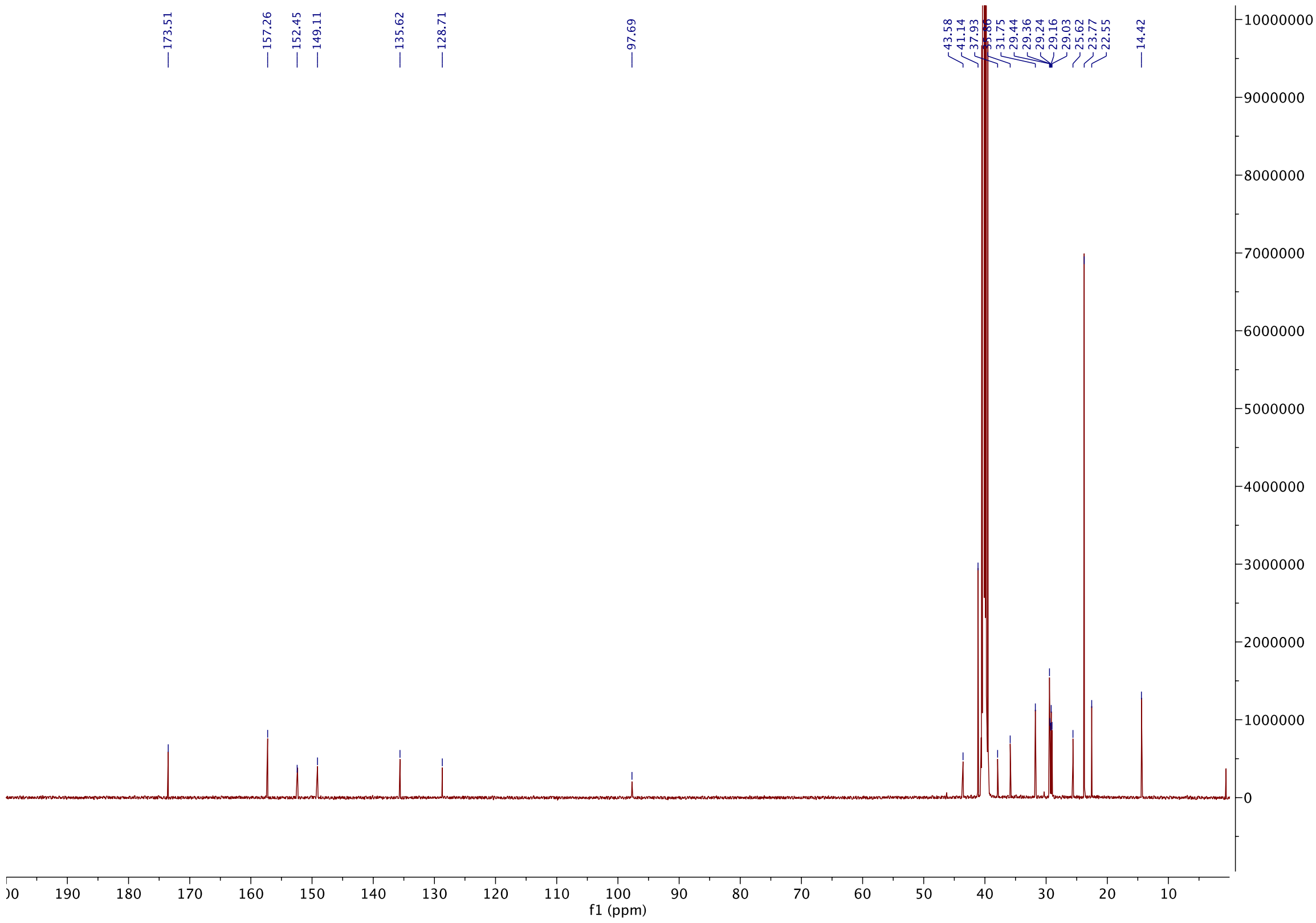

(6) ^{1}H NMR (500 MHz, DMSO-d$_6$)

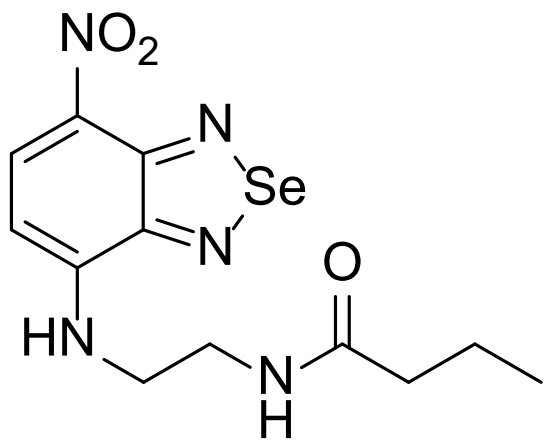

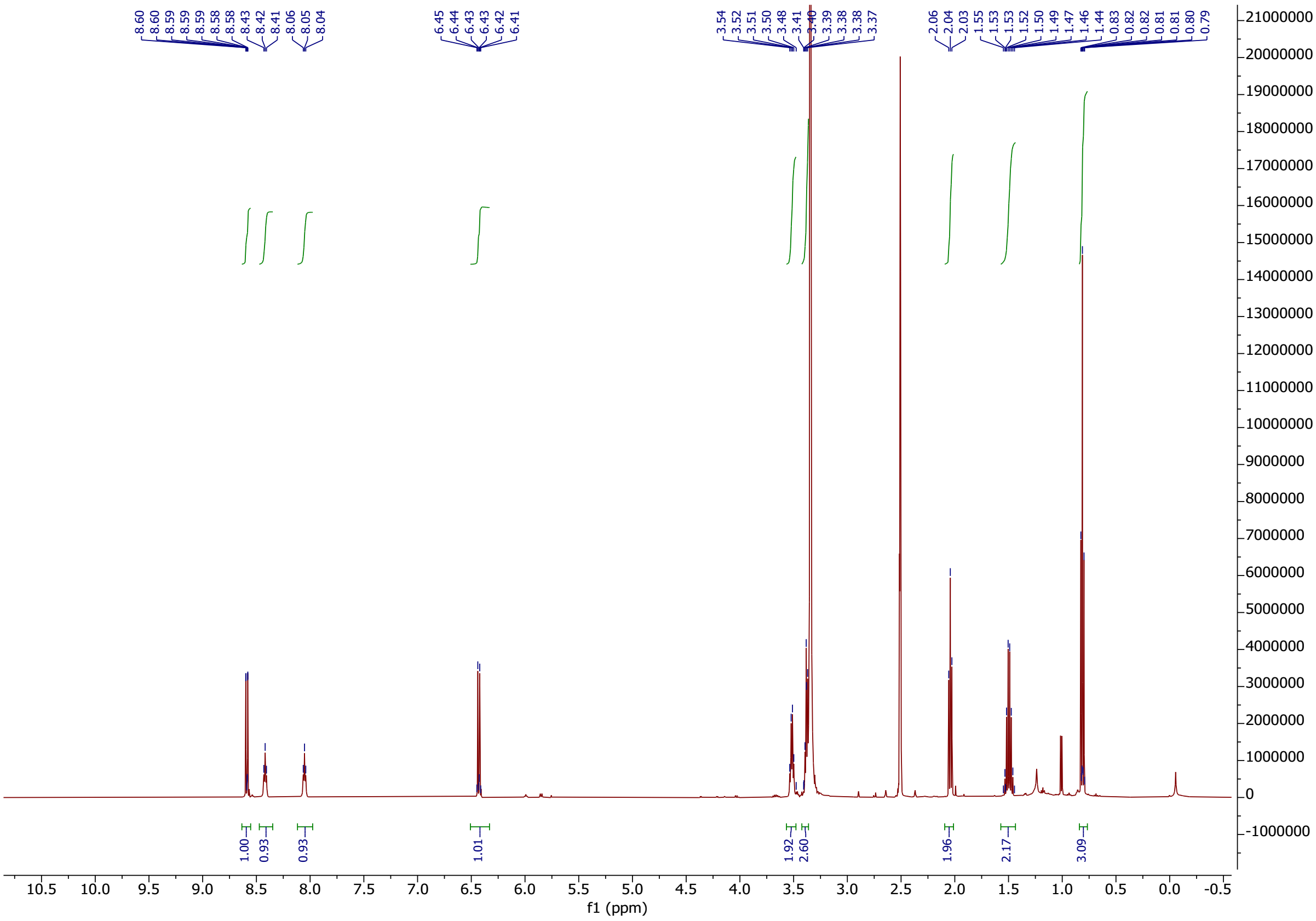

(6) ^{13}C NMR (126 MHz, DMSO-d$_6$)

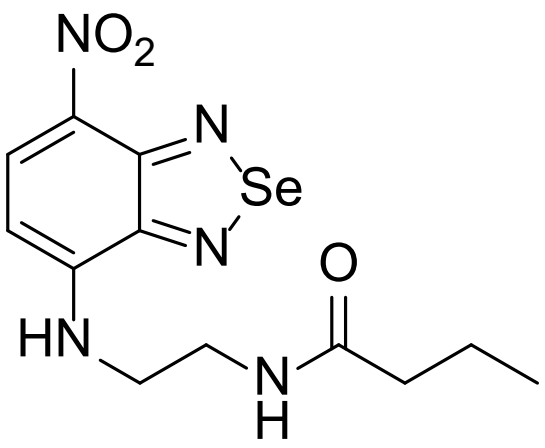

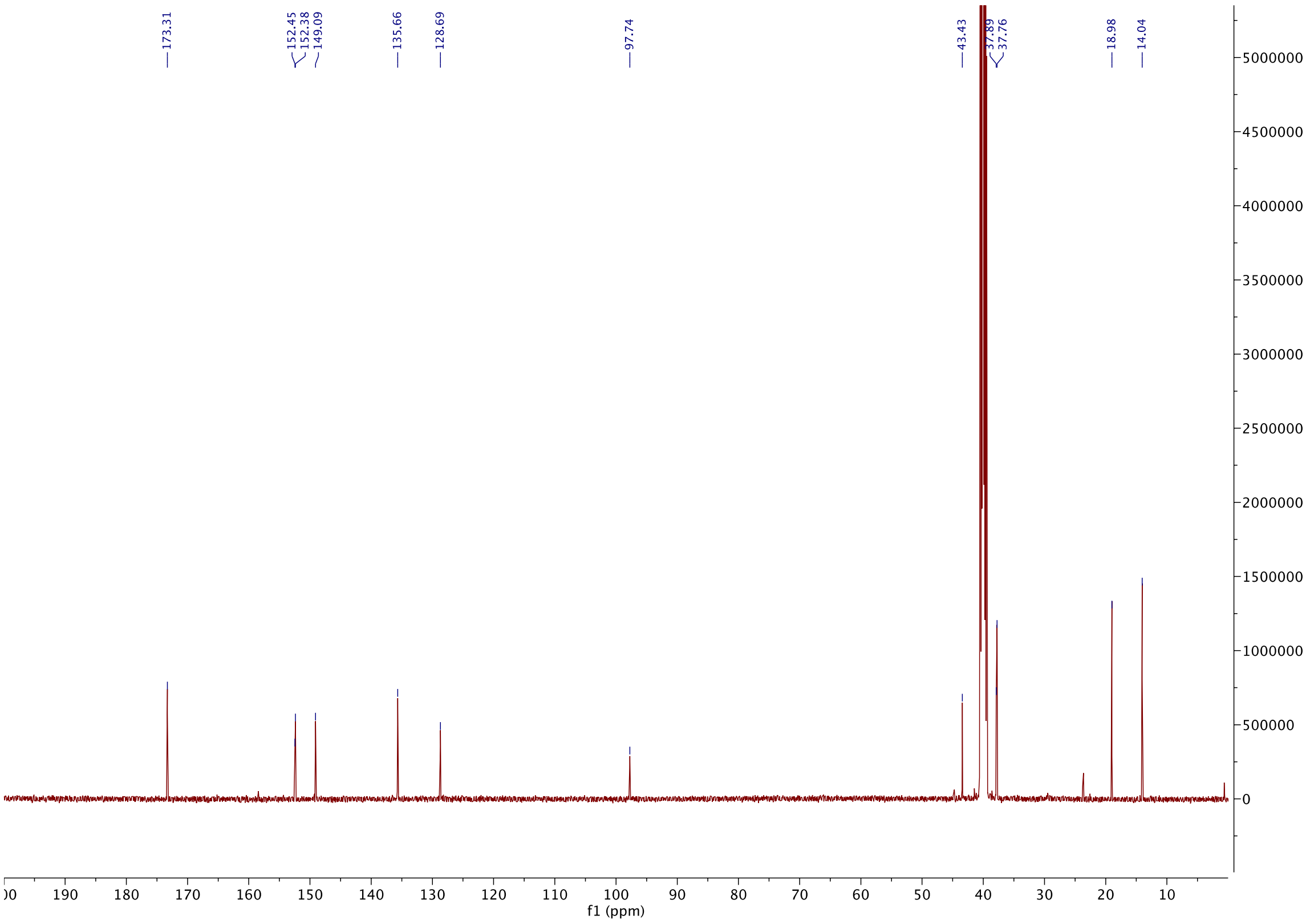

(7) ¹H NMR (500 MHz, DMSO-d₆)

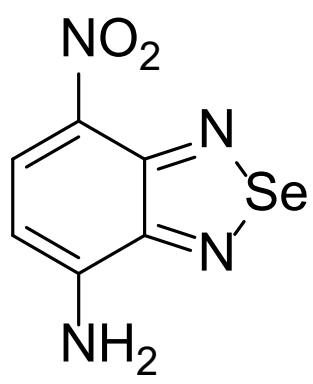

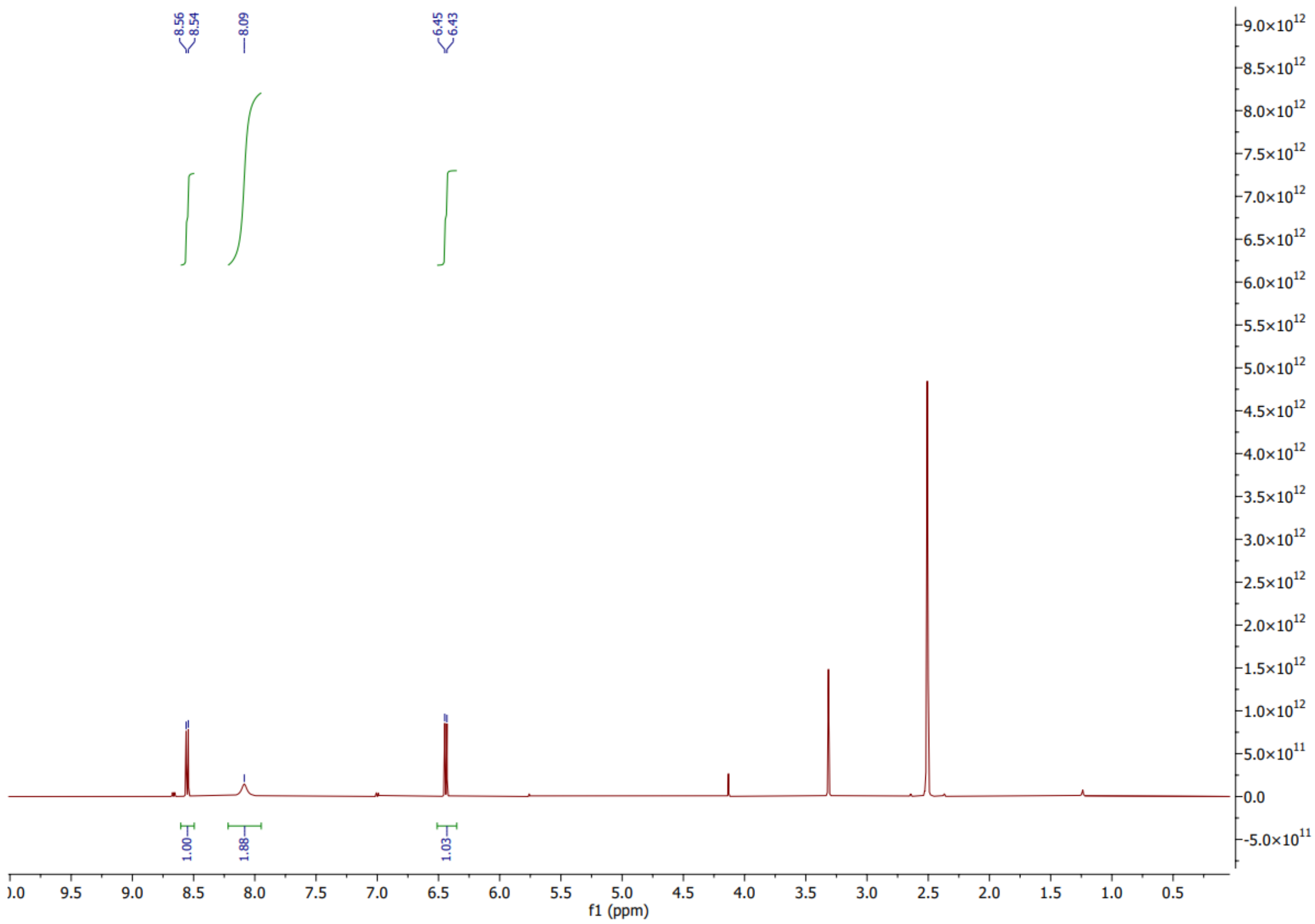

(7) ^{13}C NMR (126 MHz, DMSO-d_6)

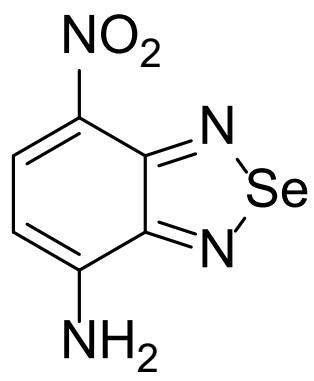

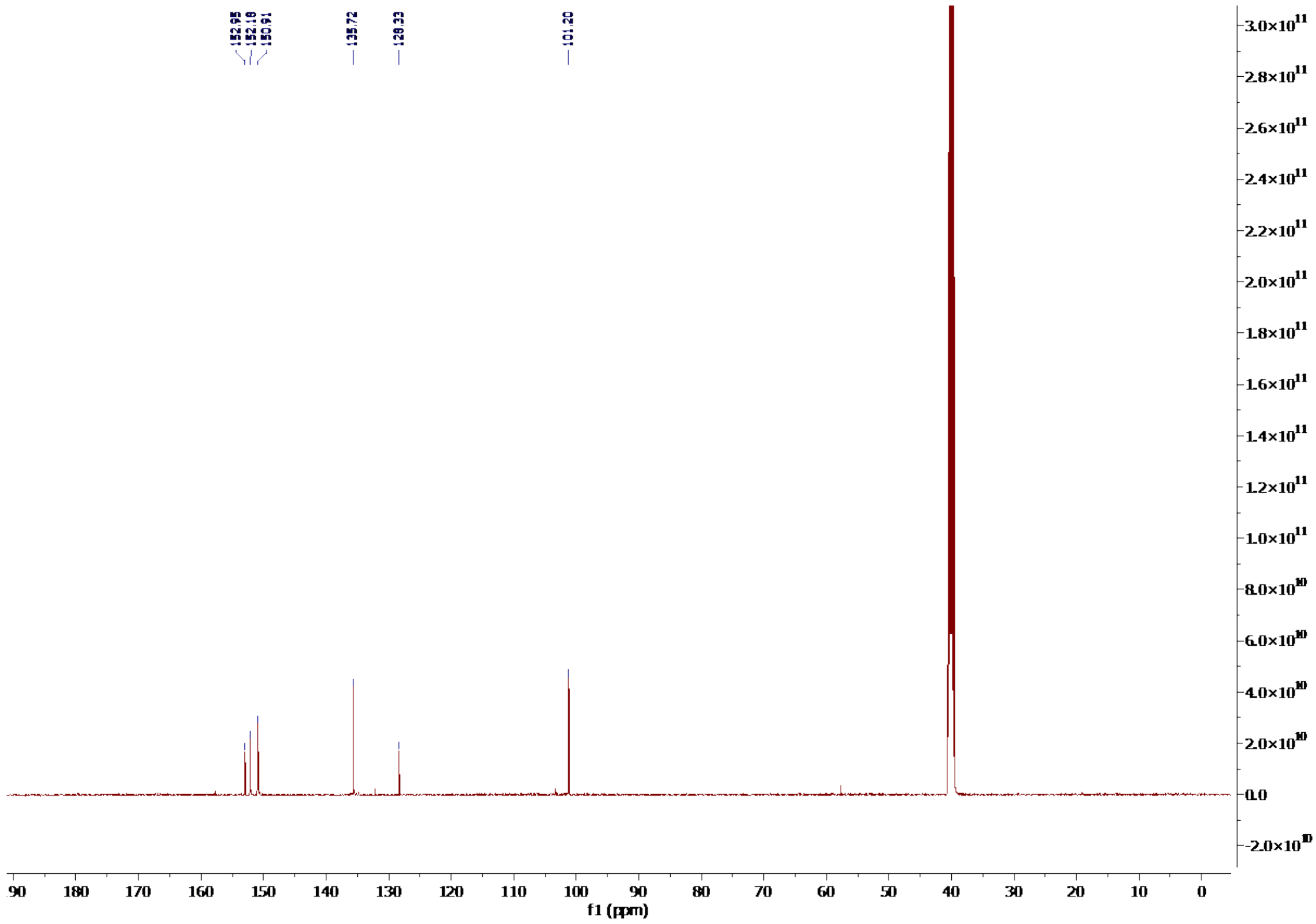

(11) ^{1}H NMR (500 MHz, $CDCl_3$)

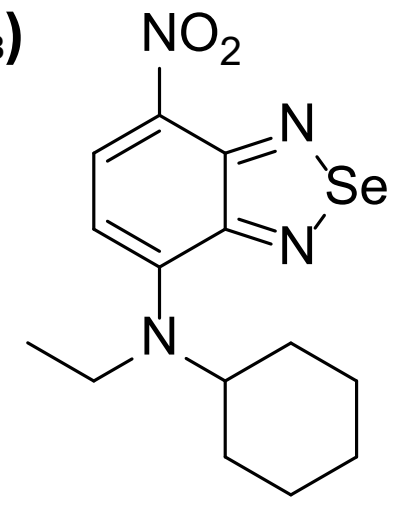

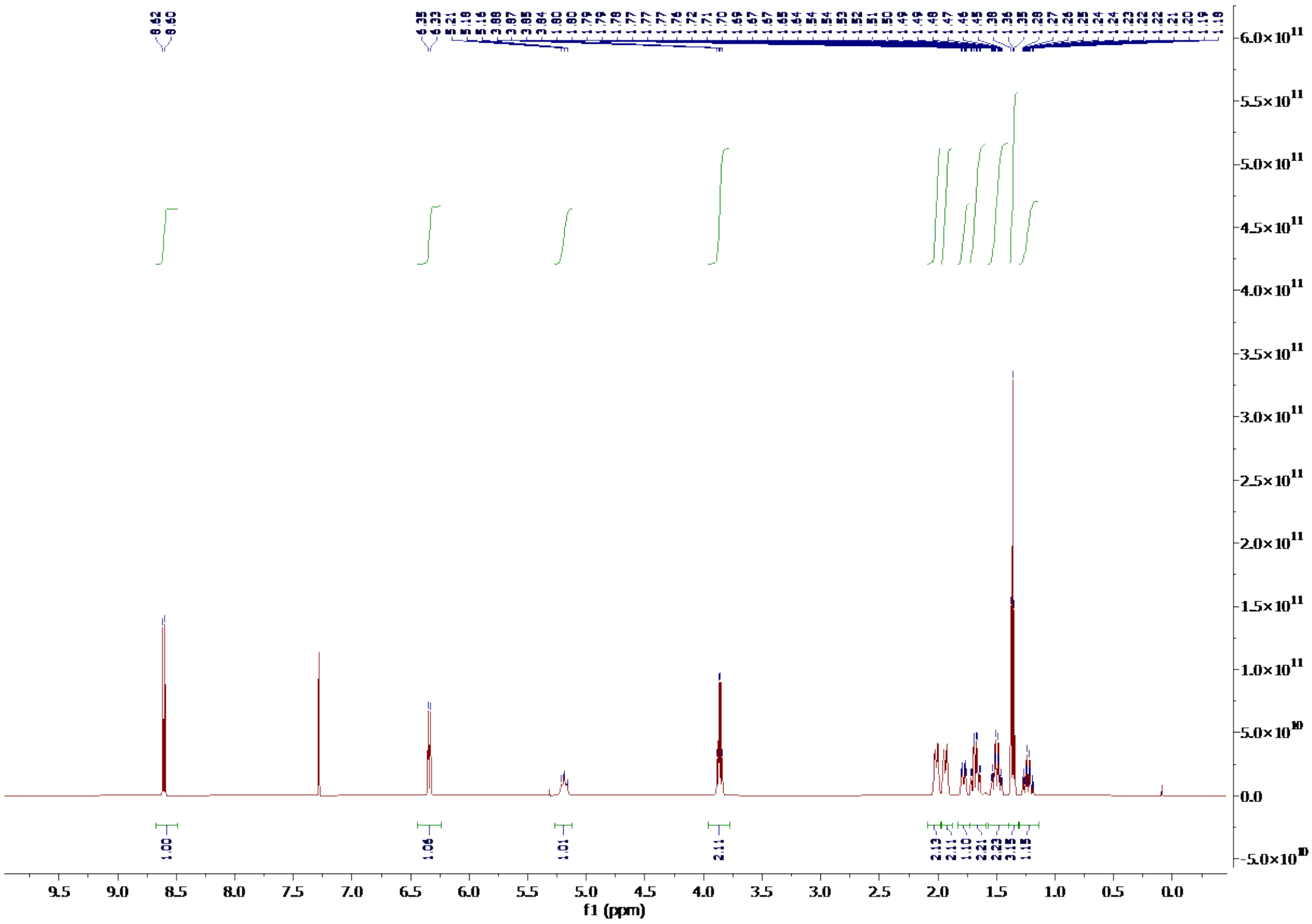

(11) ^{13}C NMR (126 MHz, $CDCl_3$)

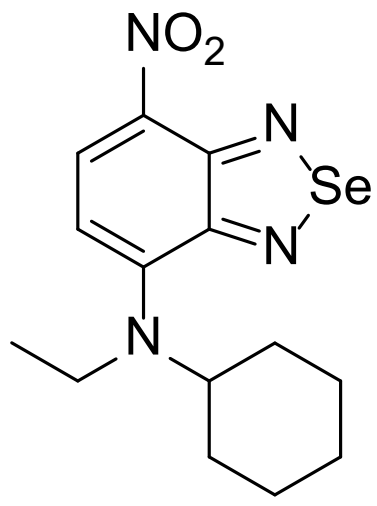

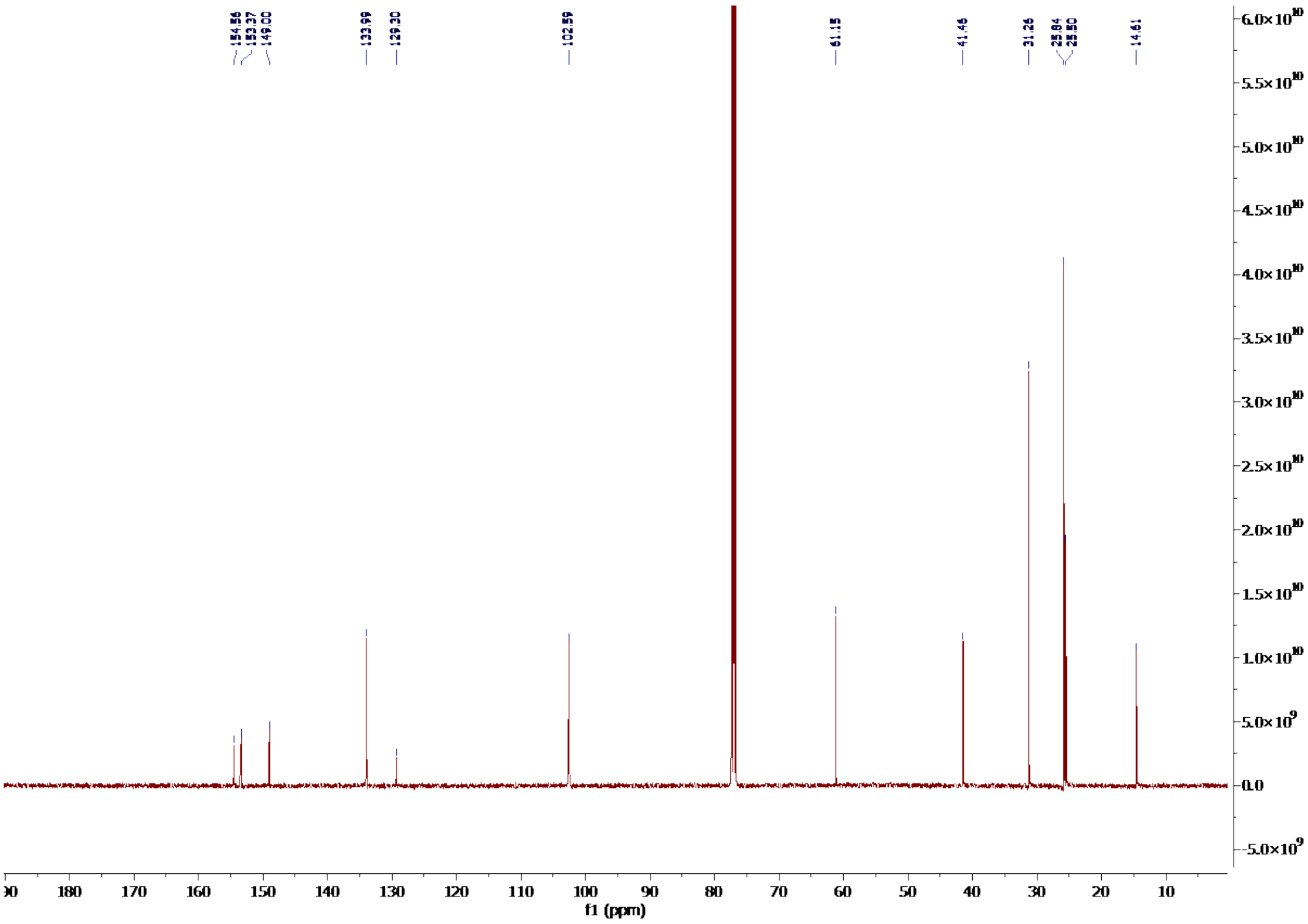

(13) ^{1}H NMR (500 MHz, DMSO-d_6)

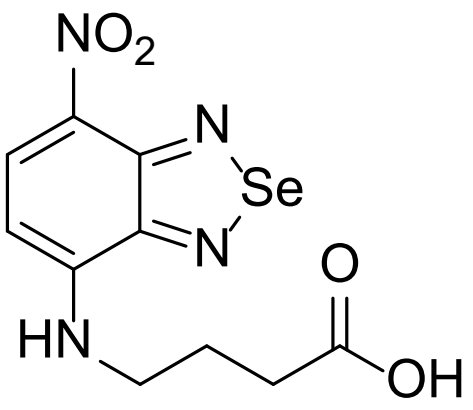

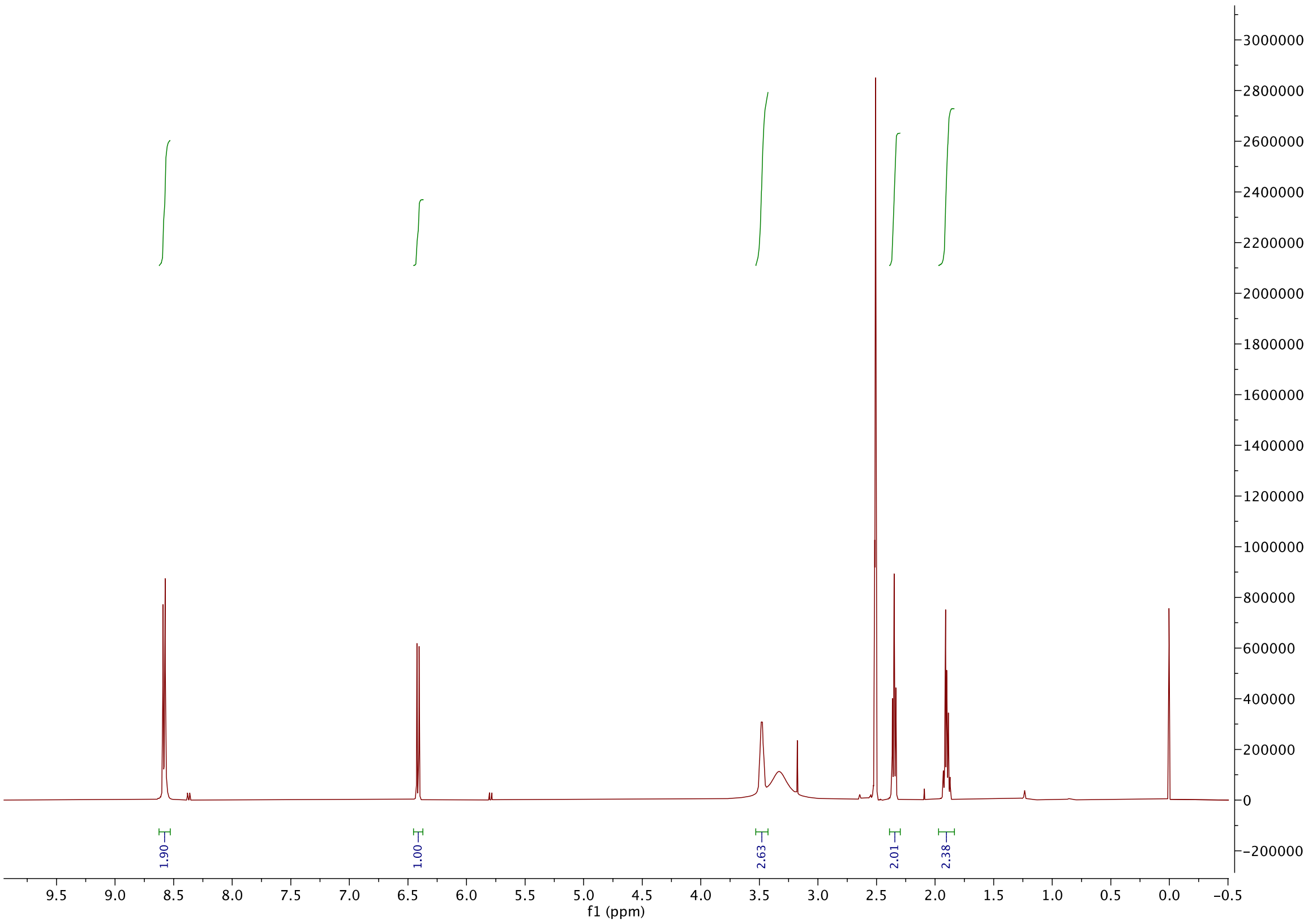

(13) ^{13}C NMR (126 MHz, DMSO-d_6)

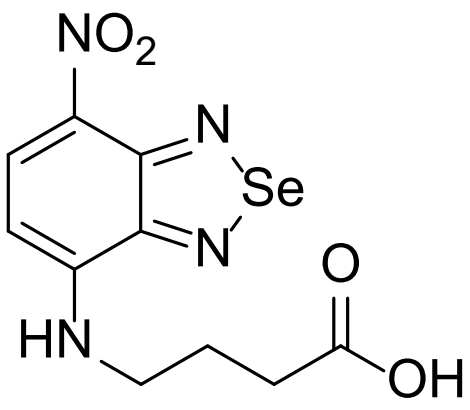

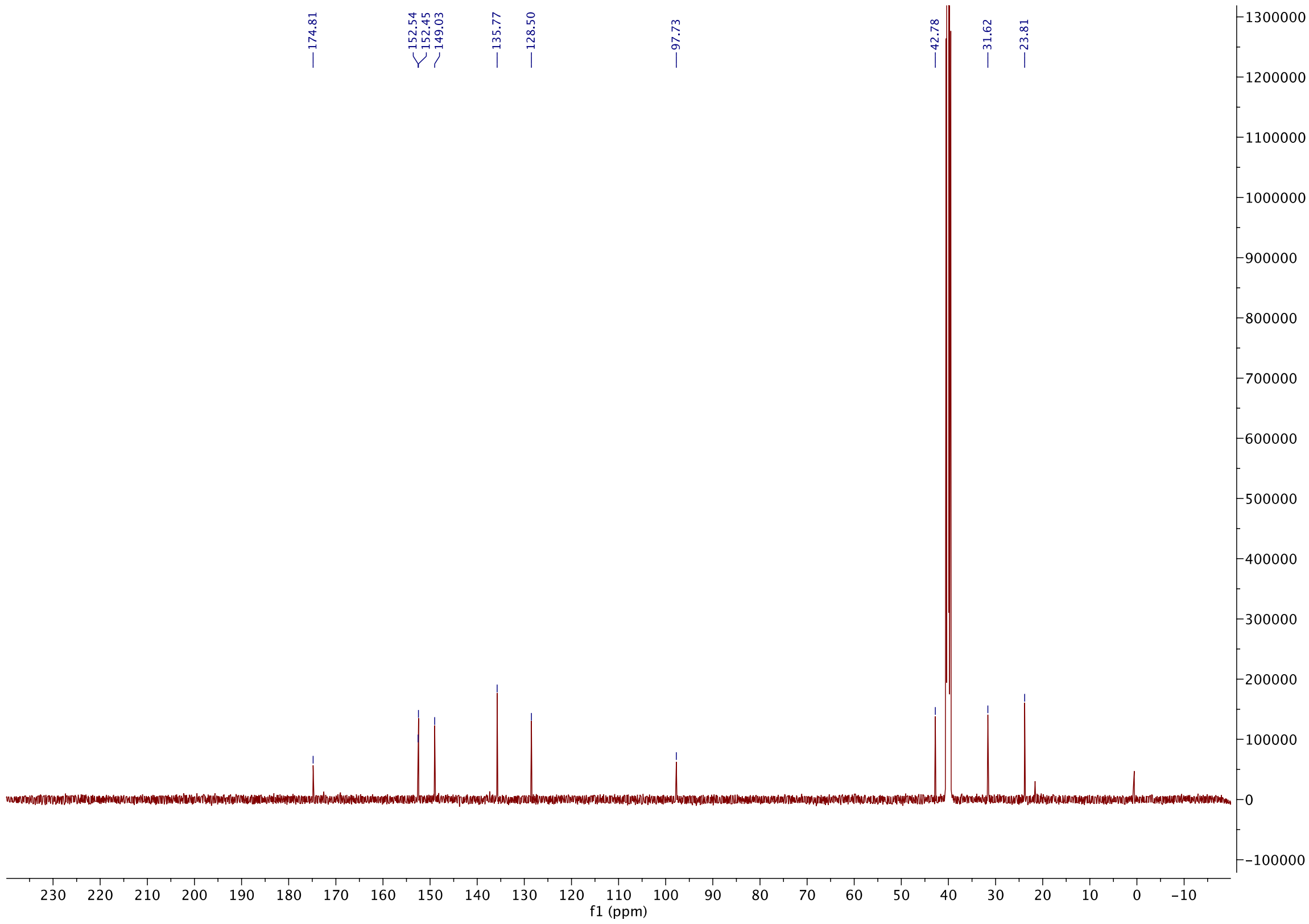

(14.1) ^{1}H NMR (500 MHz, D_2O)

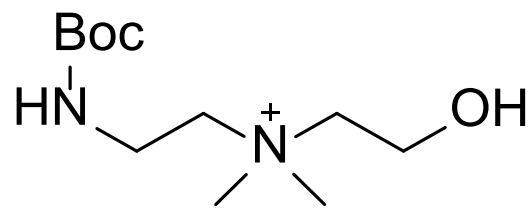

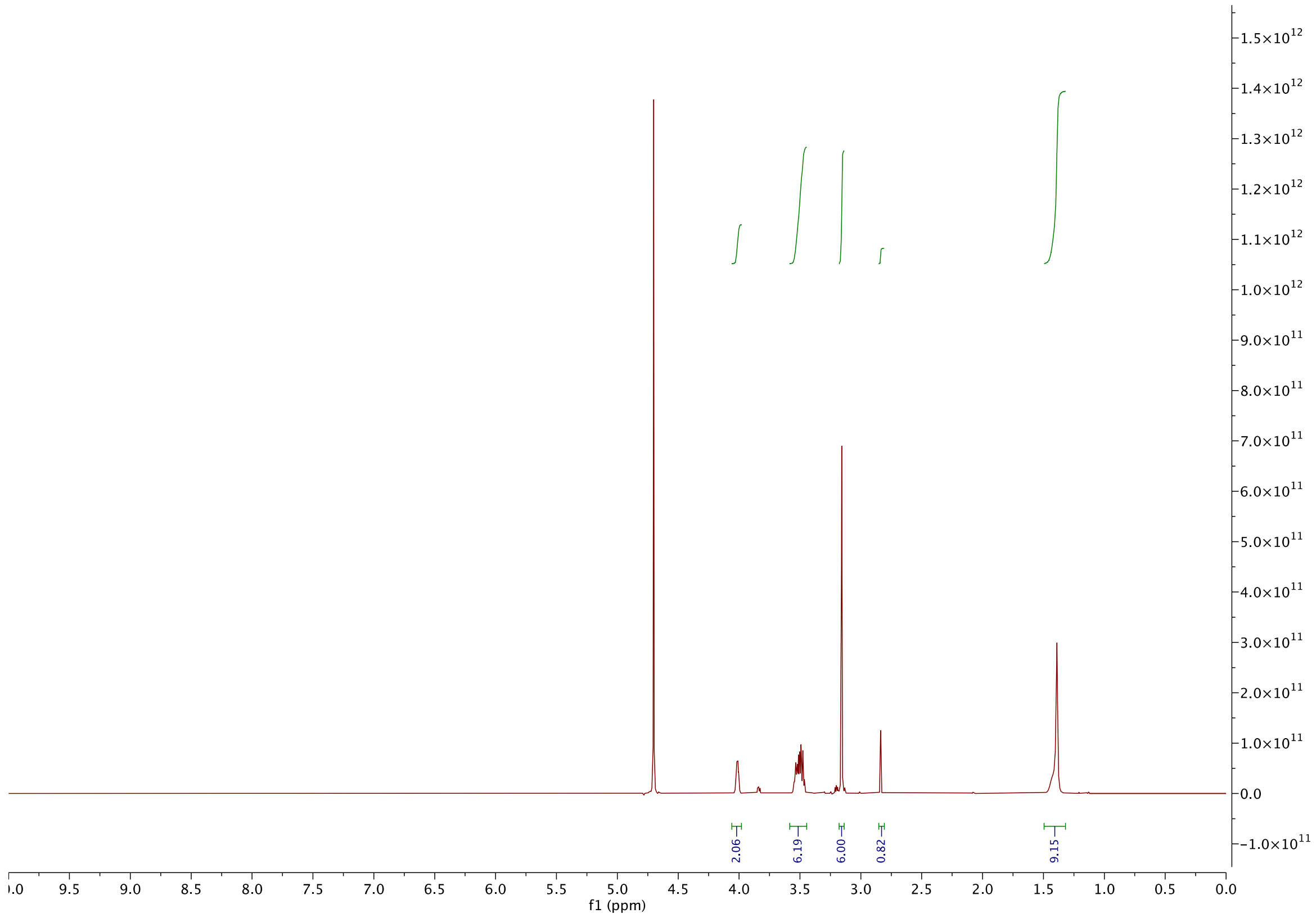

(14.1) ^{13}C NMR (126 MHz, D_2O)

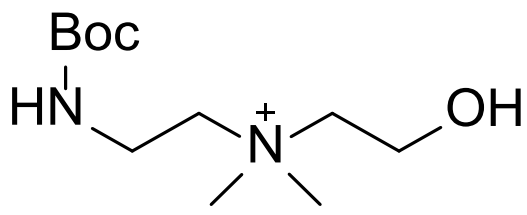

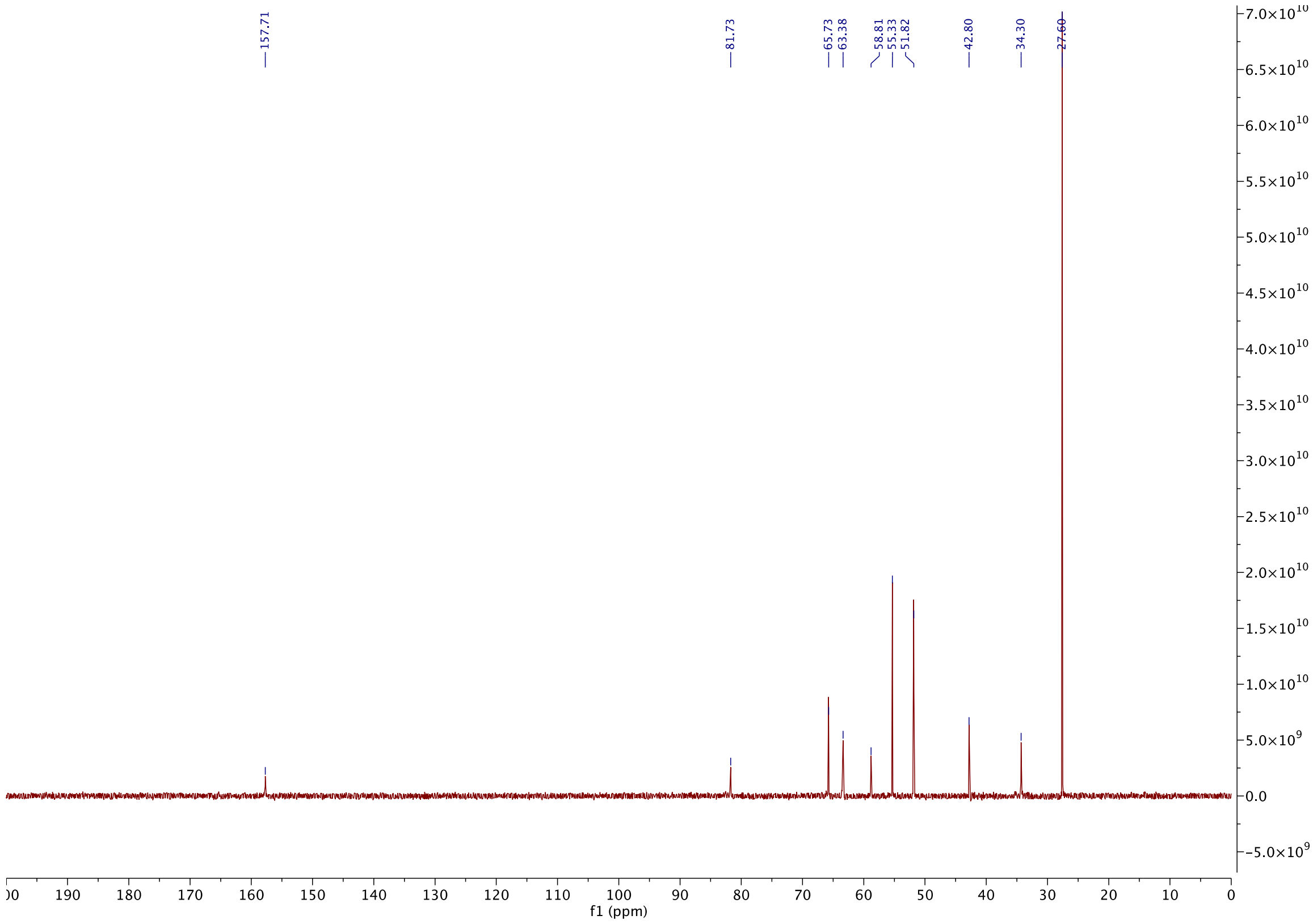

(14) ^1H NMR (500 MHz, CD_3OD)

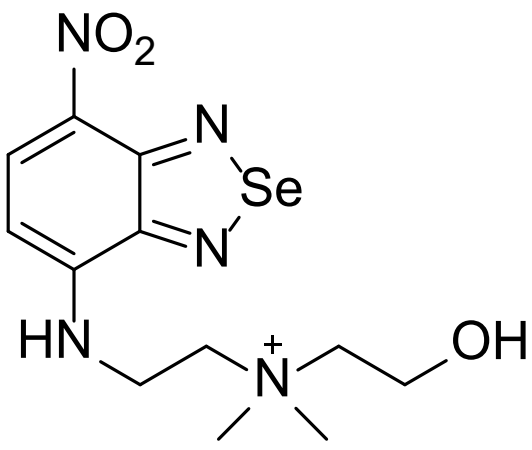

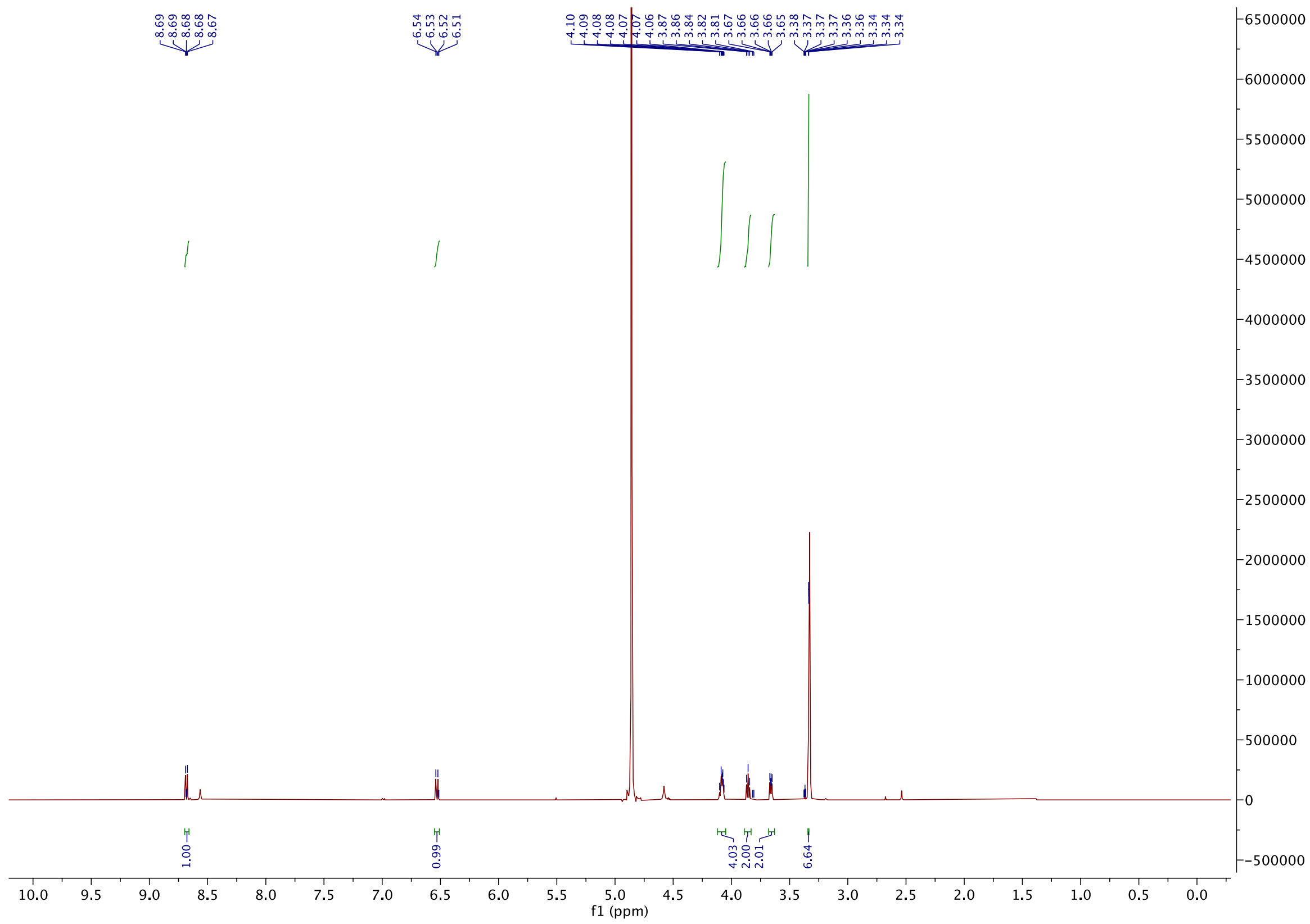

(14) ^{13}C NMR (126 MHz, CD_3OD)

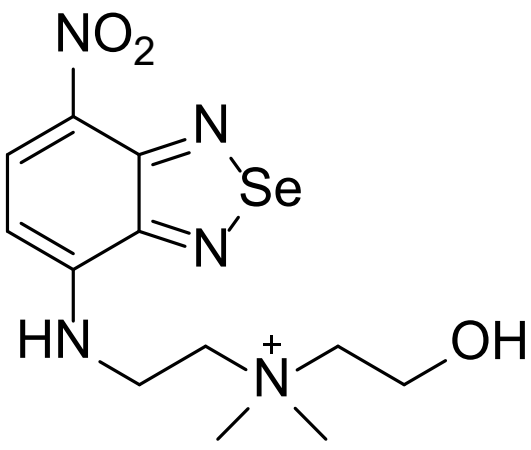

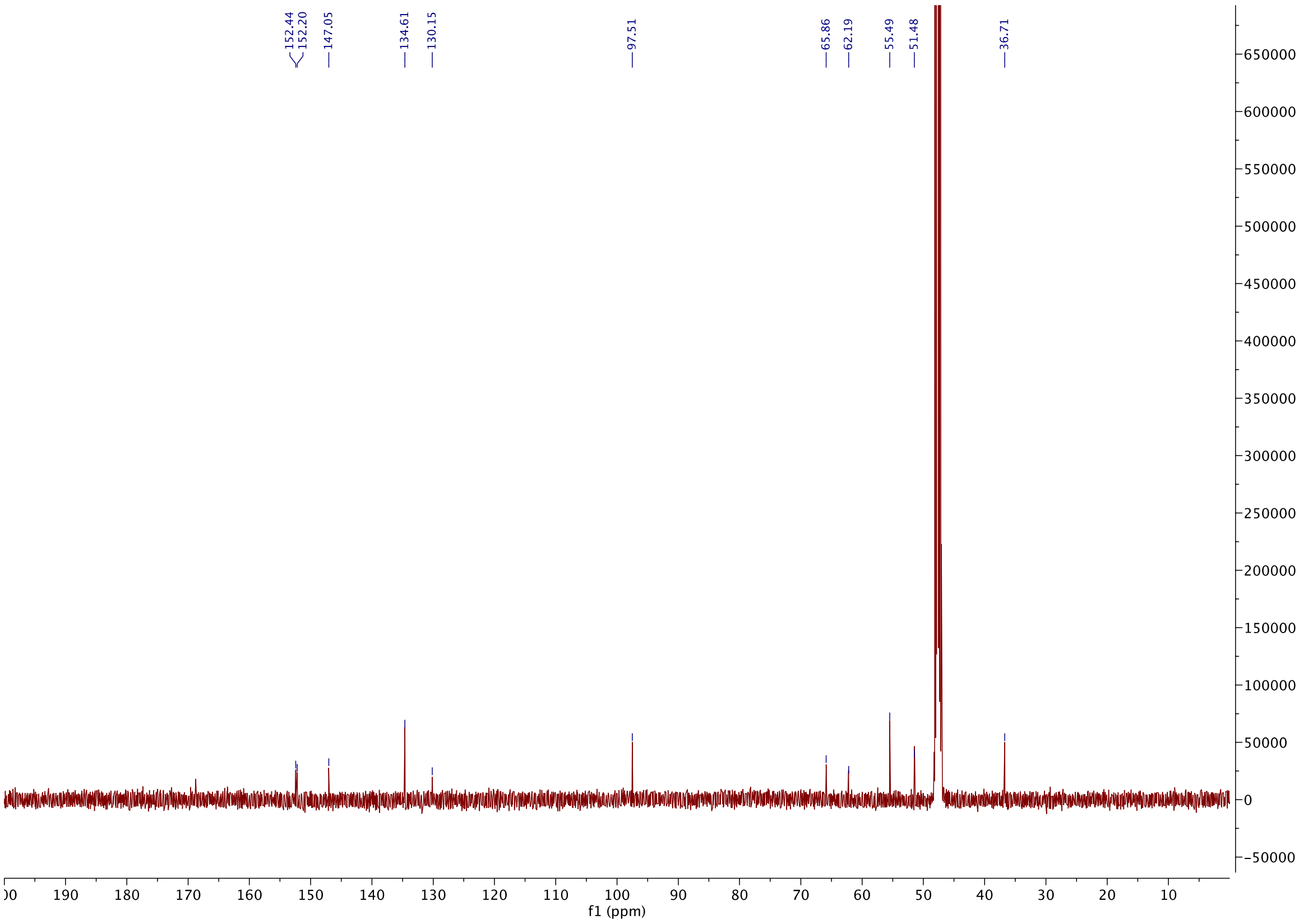

(15.1) ^{1}H NMR (500 MHz, CD_3OD)

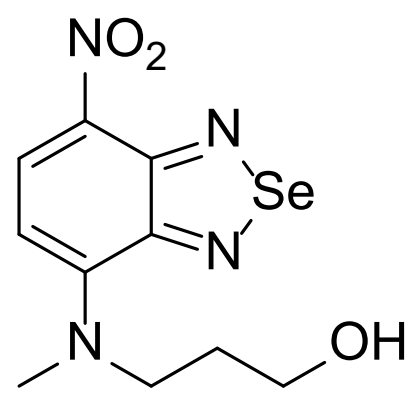

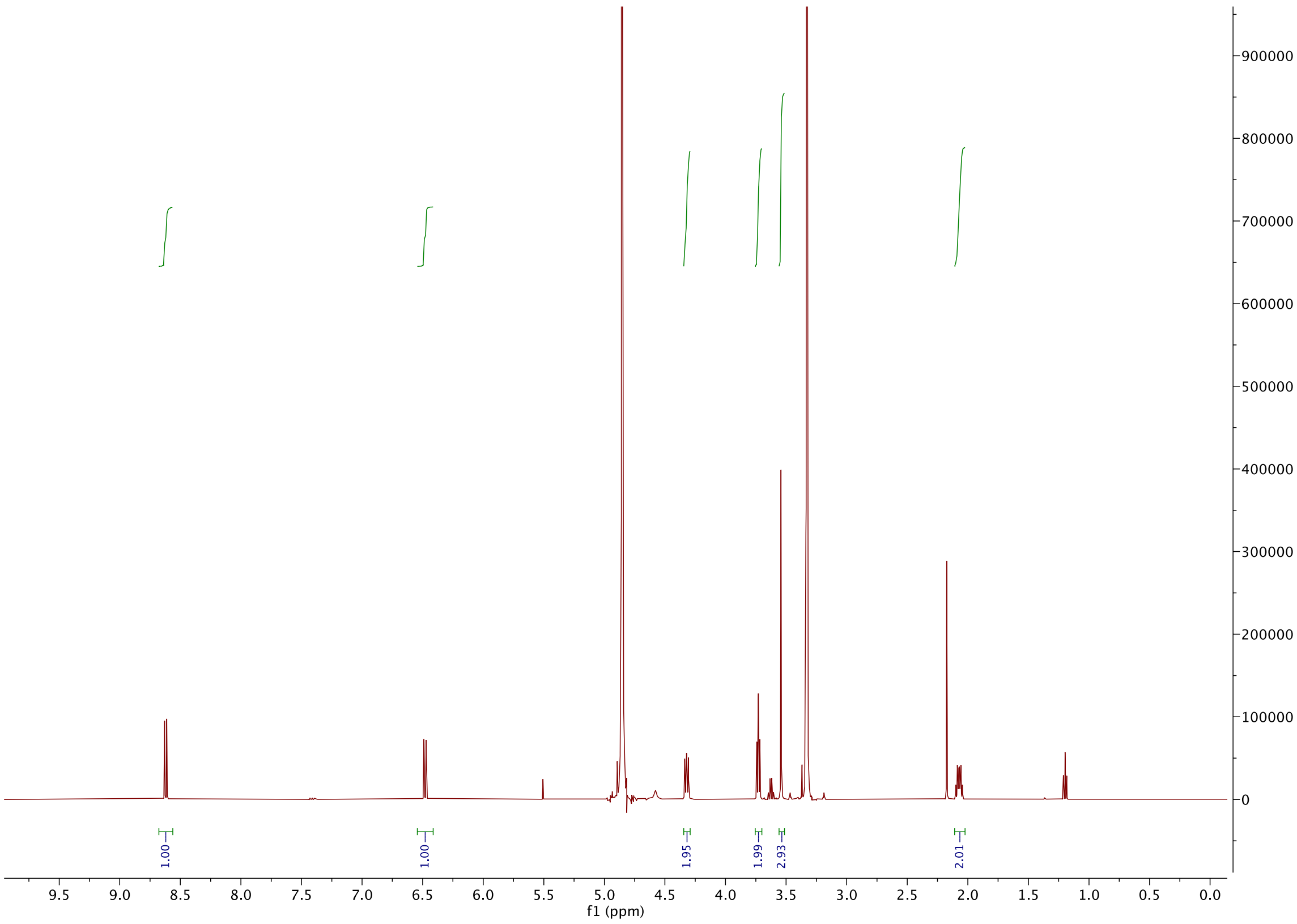

(15.1) ^{13}C NMR (126 MHz, CD_3OD)

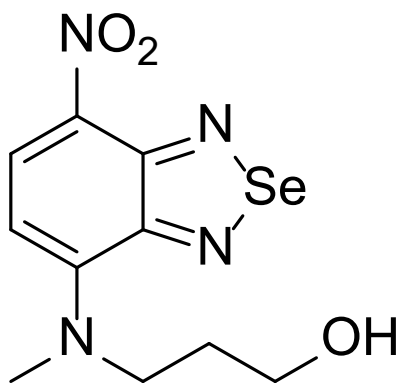

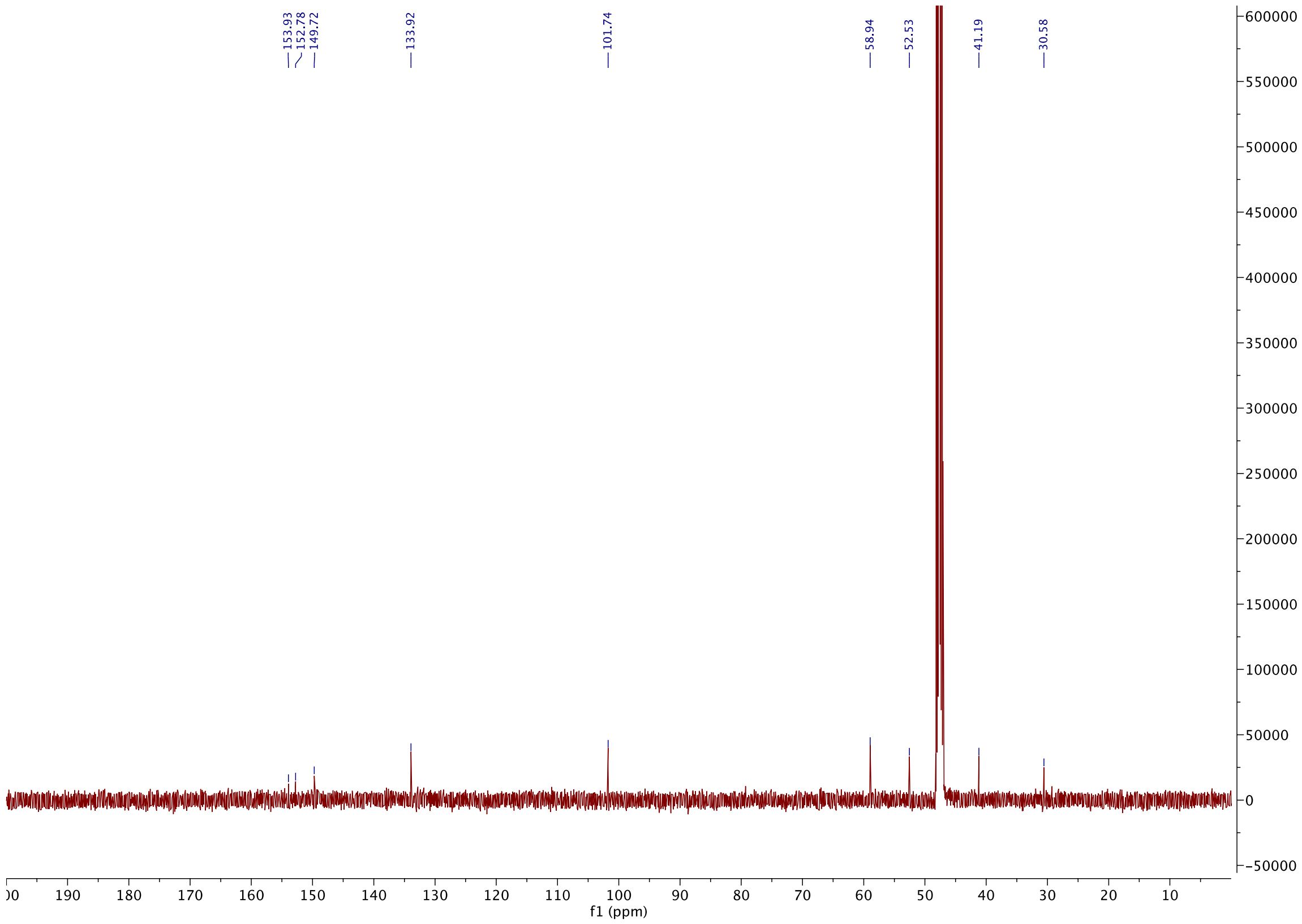

(15.2) ^{1}H NMR (500 MHz, DMSO-d_6)

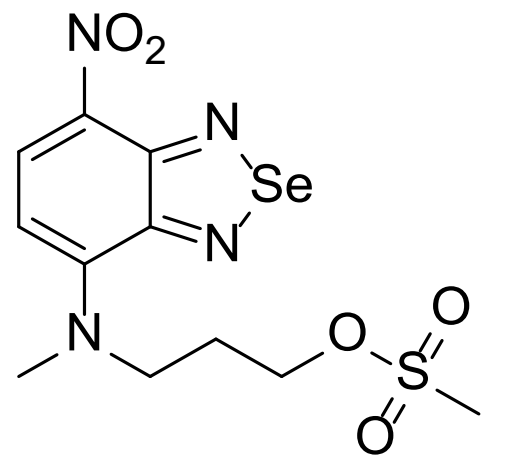

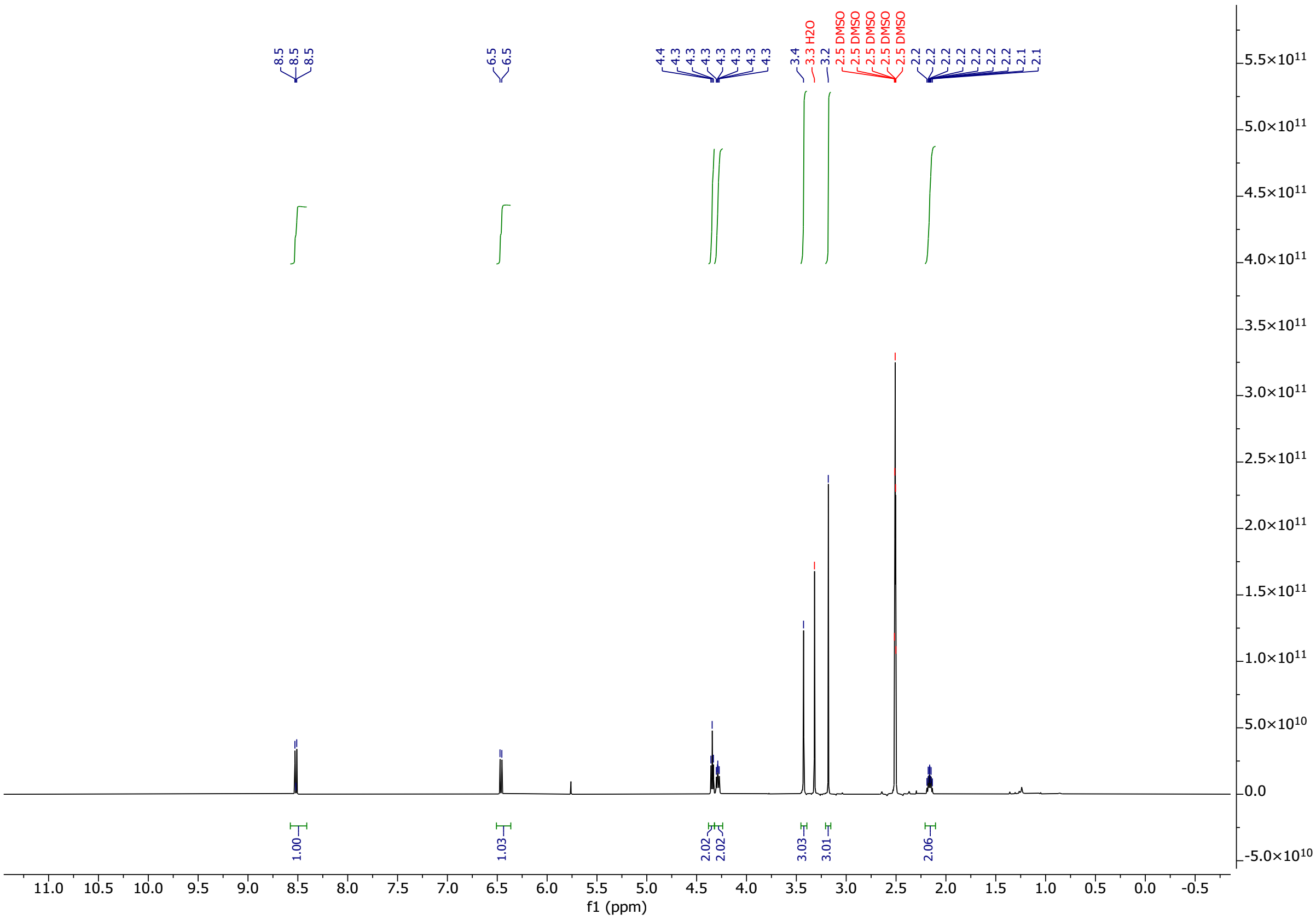

(15.2) ^{13}C NMR (126 MHz, DMSO-d_6)

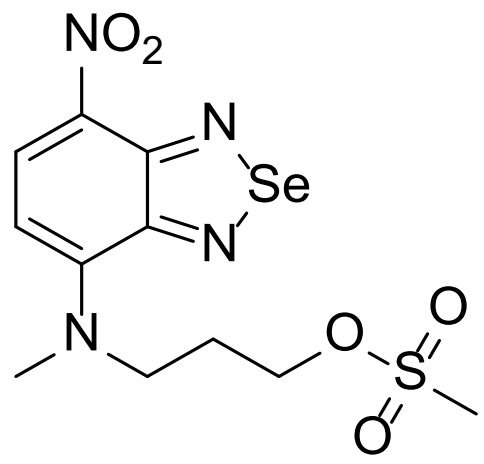

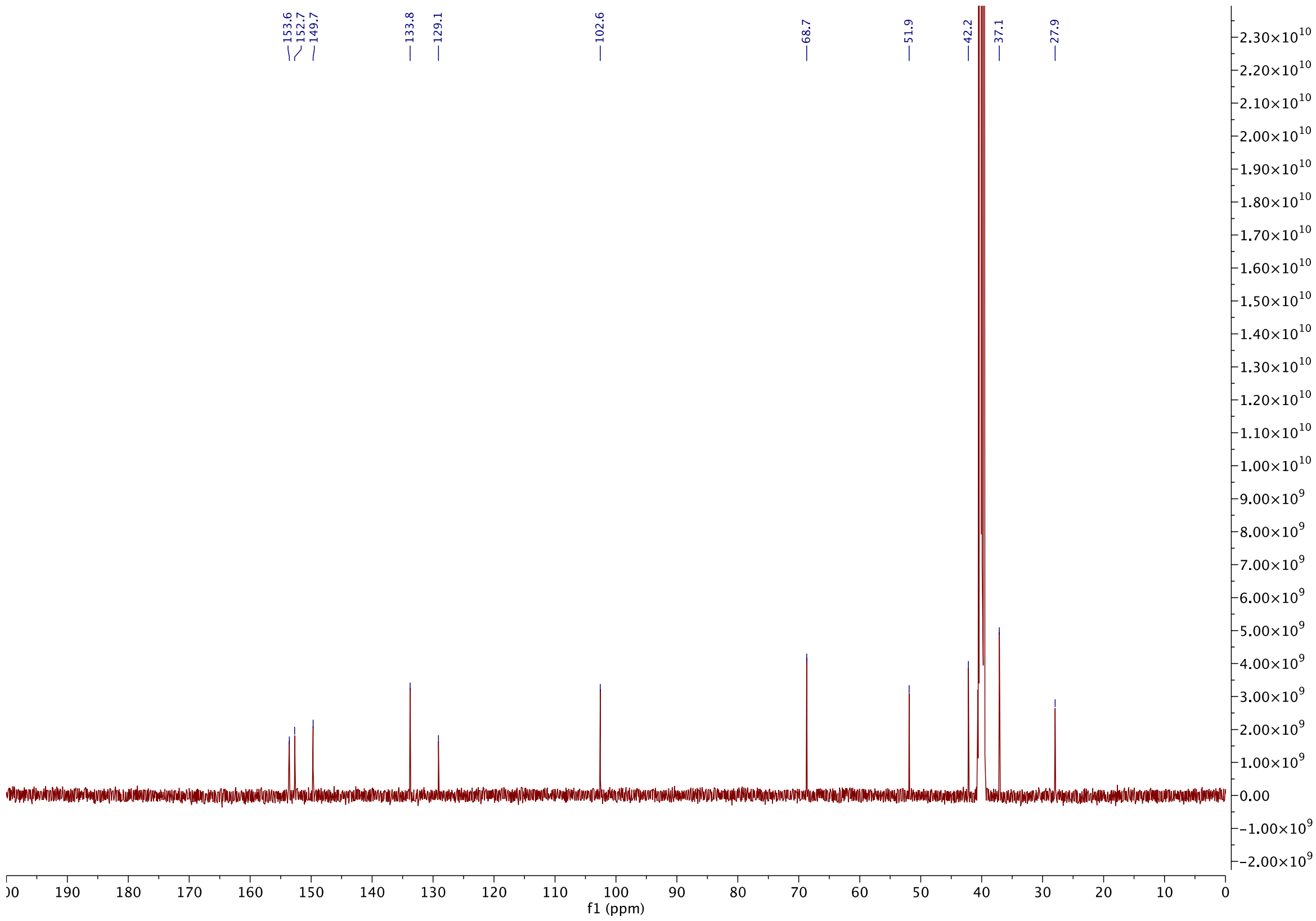

(15) ¹H NMR (500 MHz, DMSO-d₆)

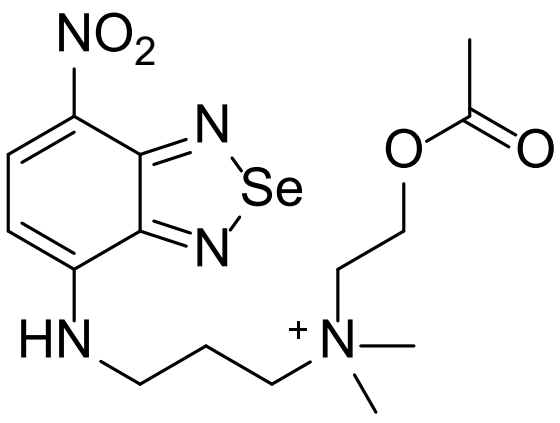

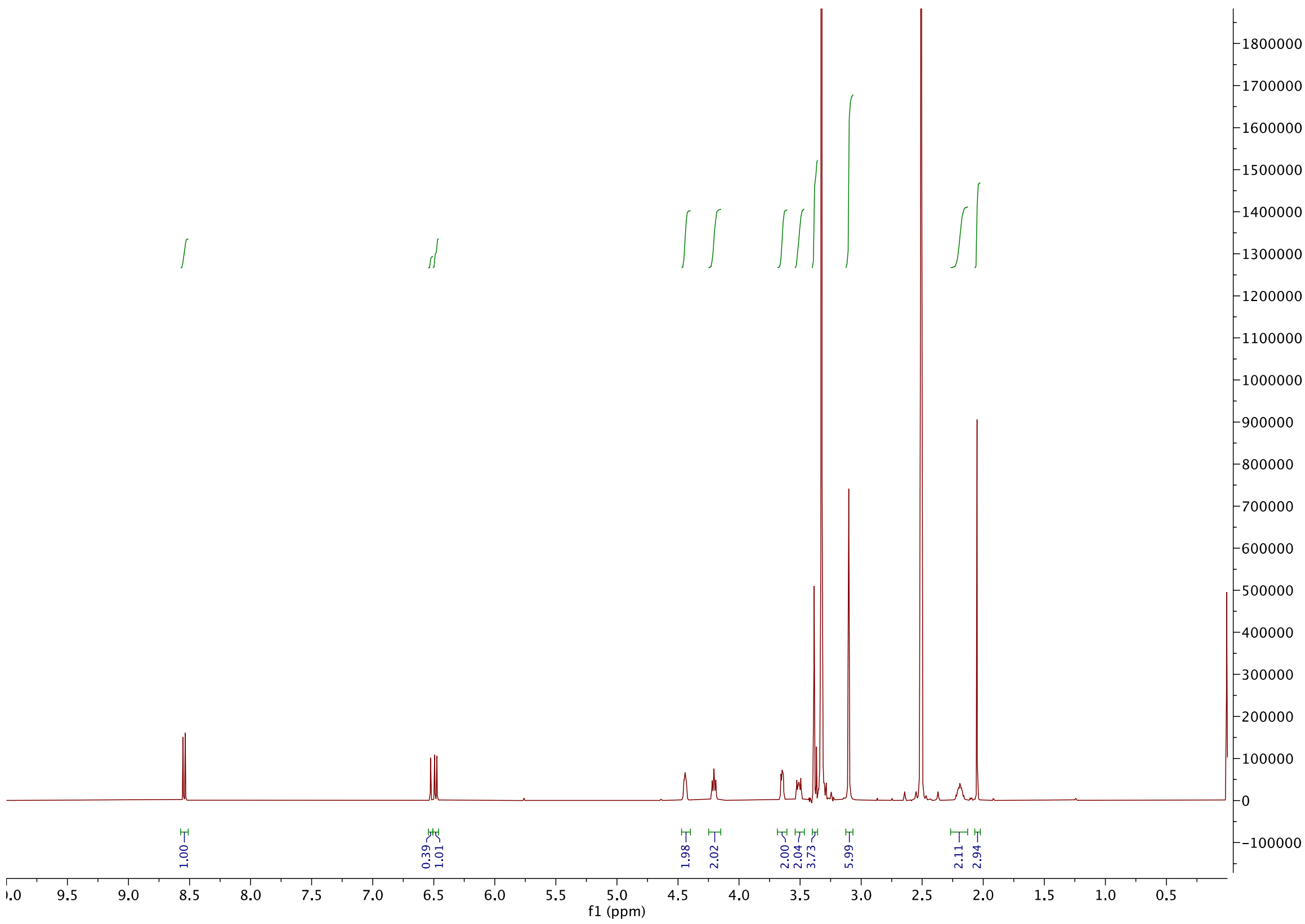

(15) ^{13}C NMR (126 MHz, DMSO-d_6)

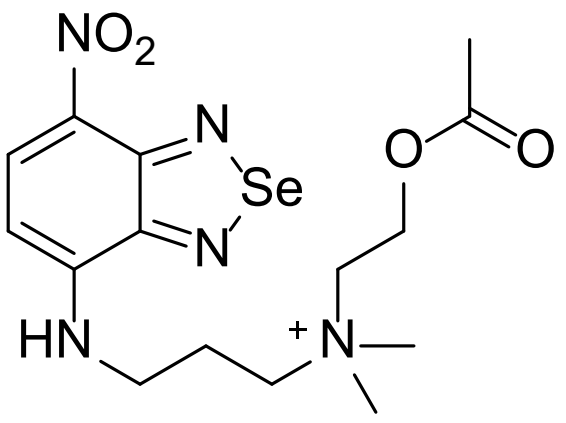

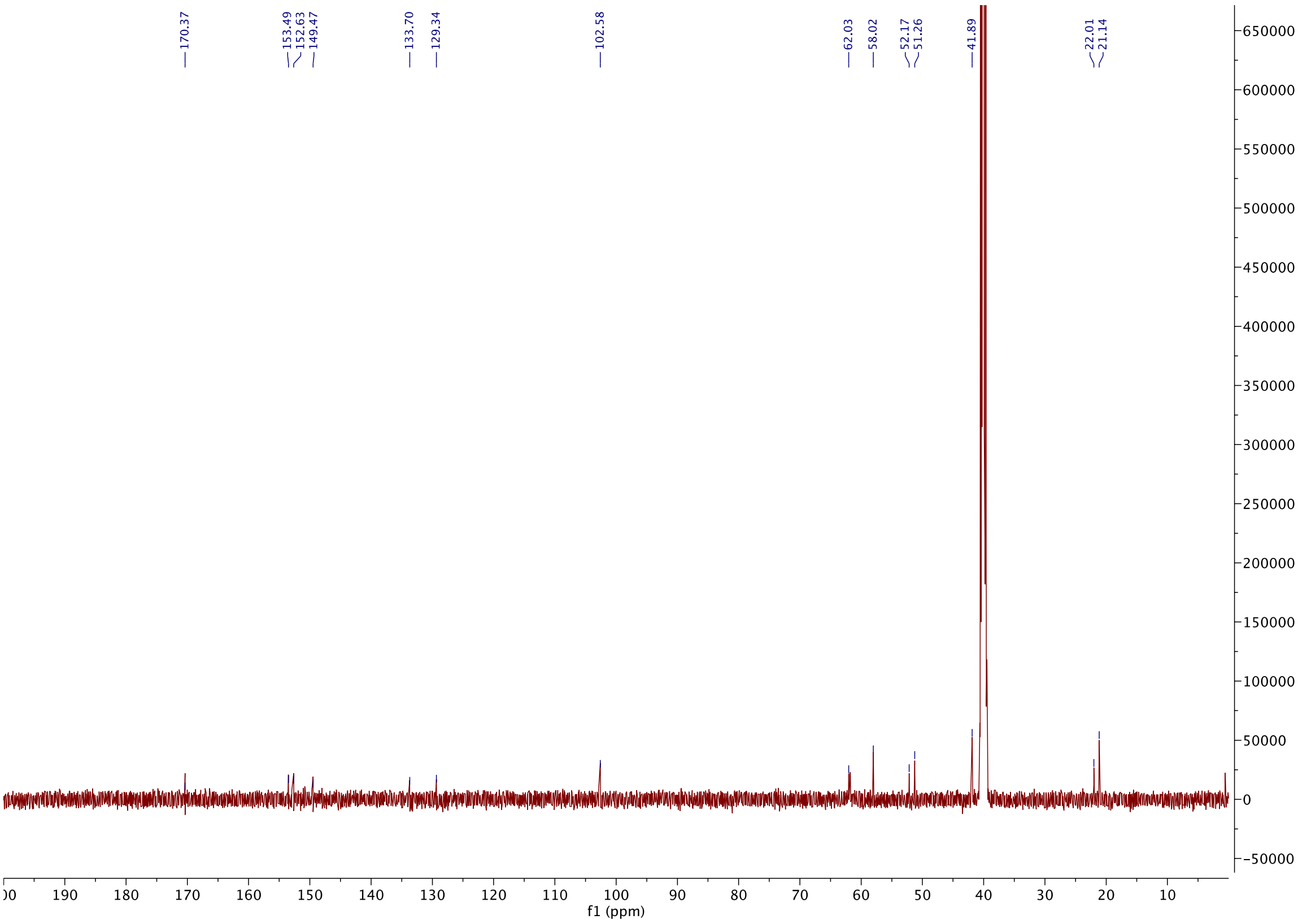

(16.1) ^{1}H NMR (500 MHz, $CDCl_3$)

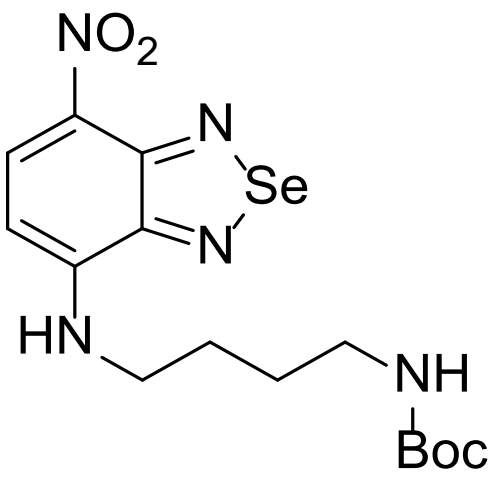

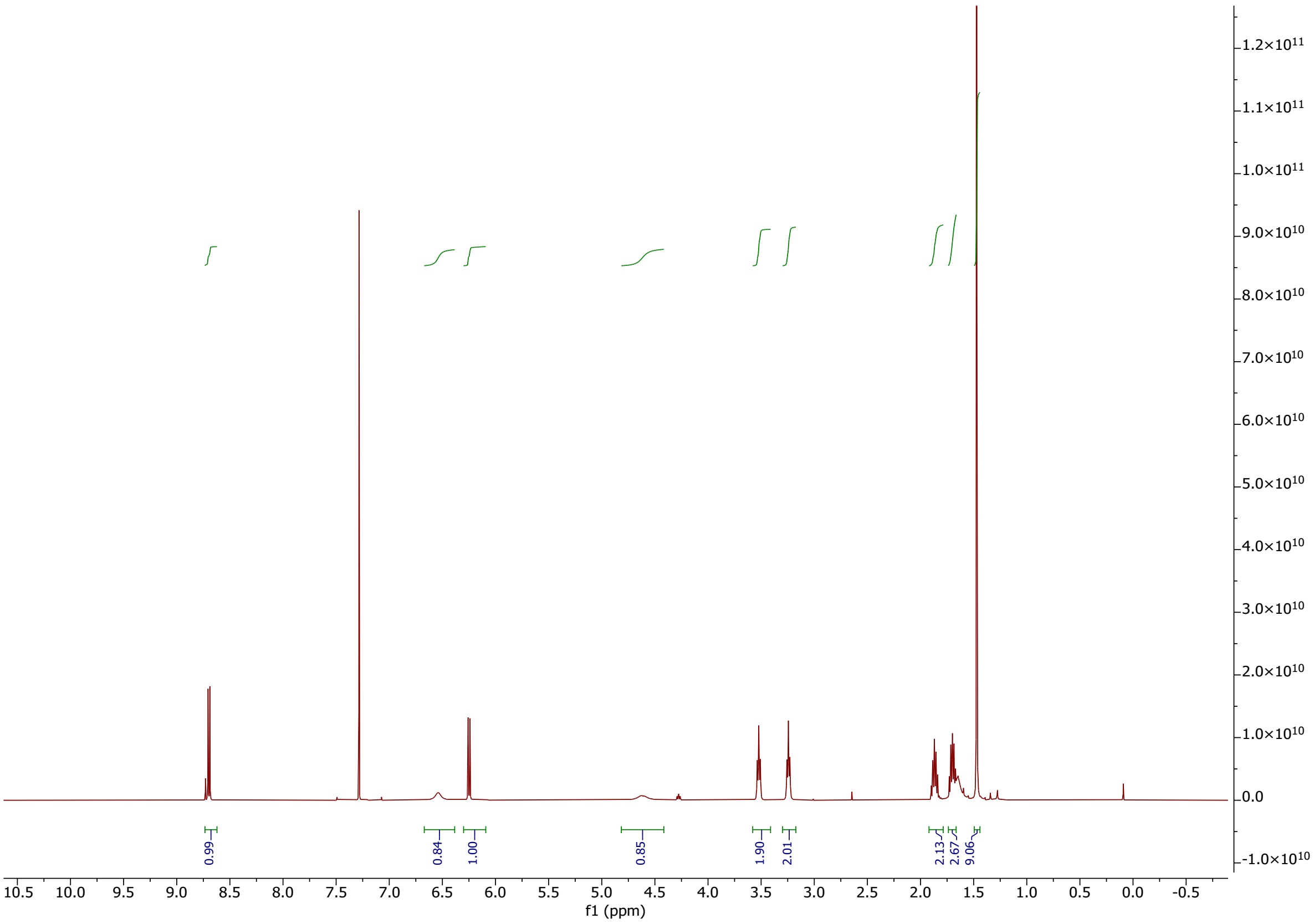

(16.1) ^{13}C NMR (126 MHz, $CDCl_3$)

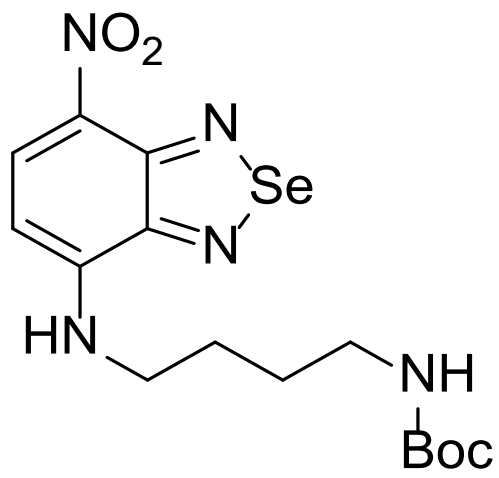

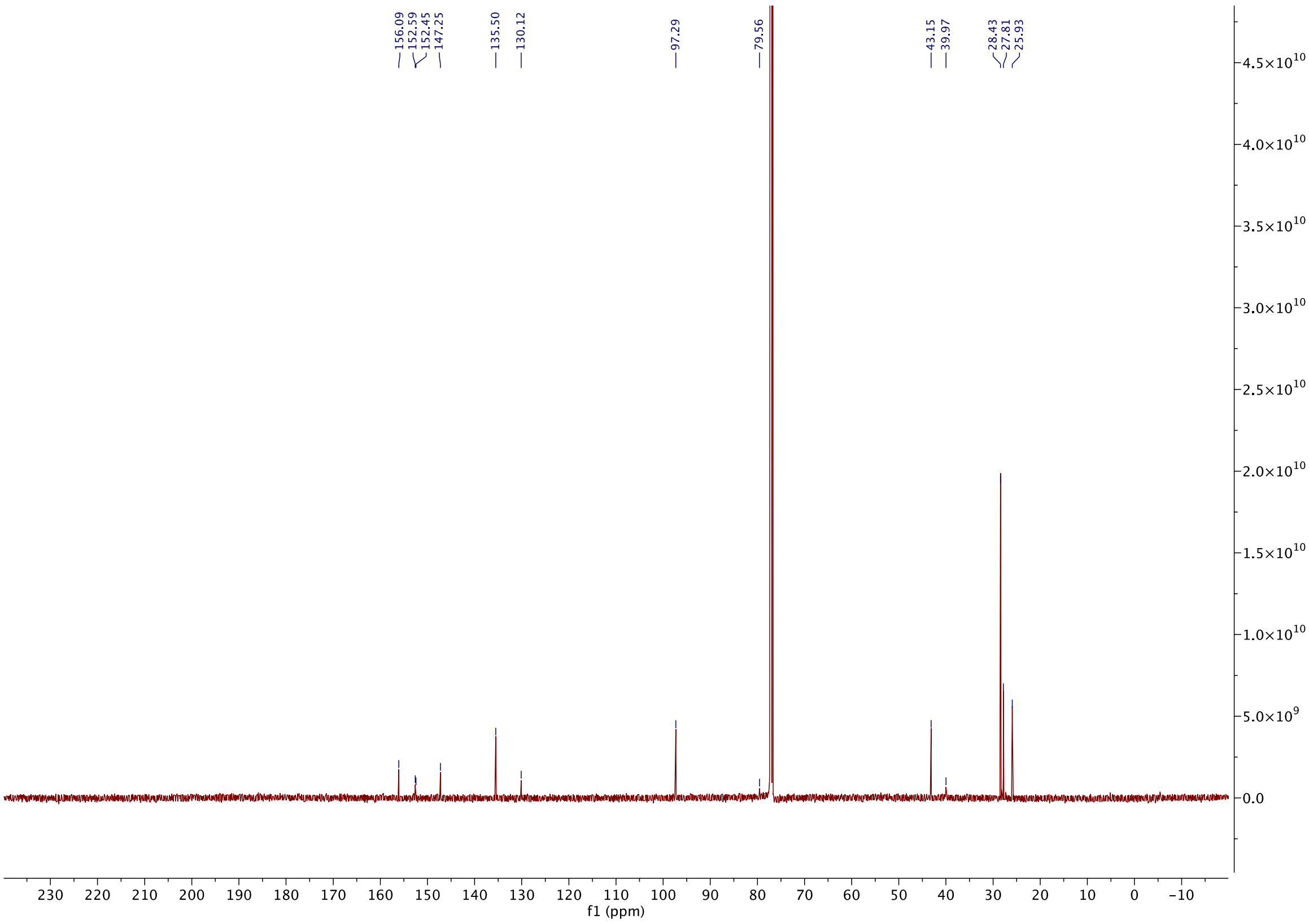

(16) ^{1}H NMR (500 MHz, CD_3OD)

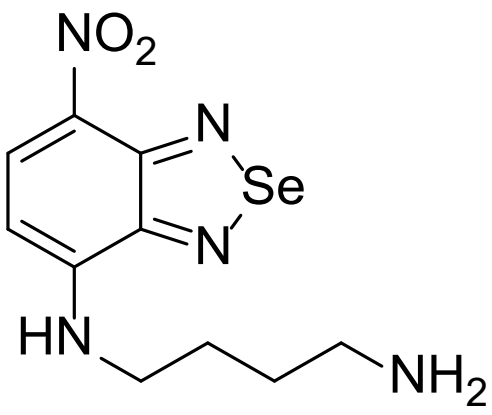

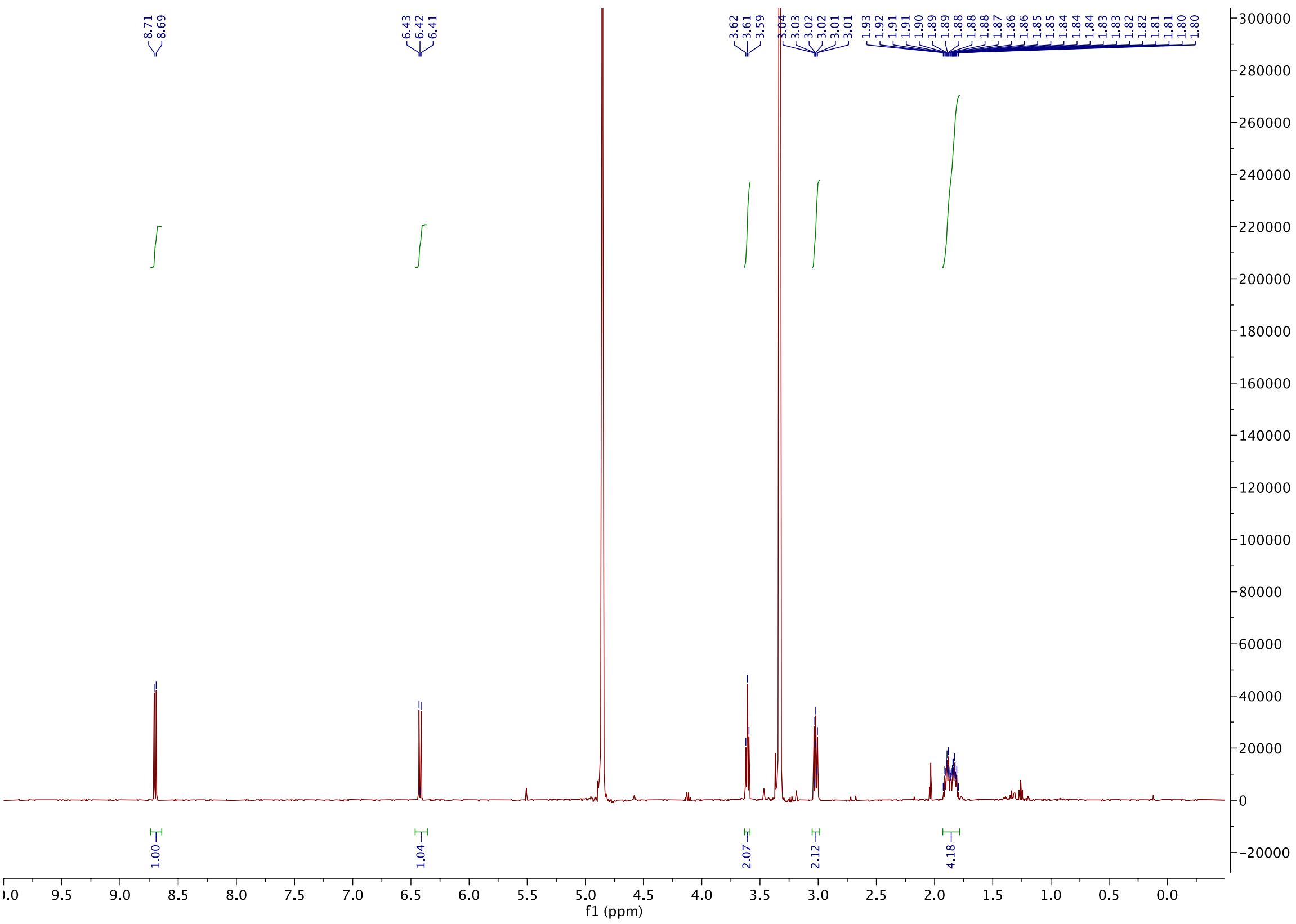

(16) ^{13}C NMR (126 MHz, CD_3OD)

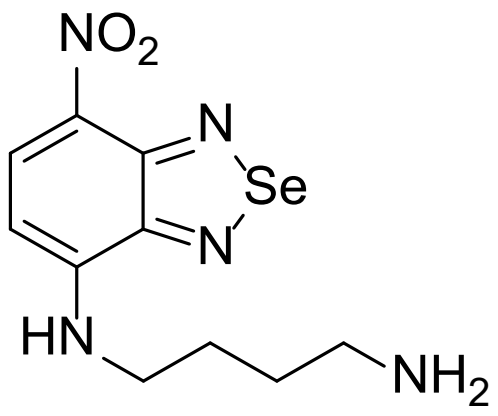

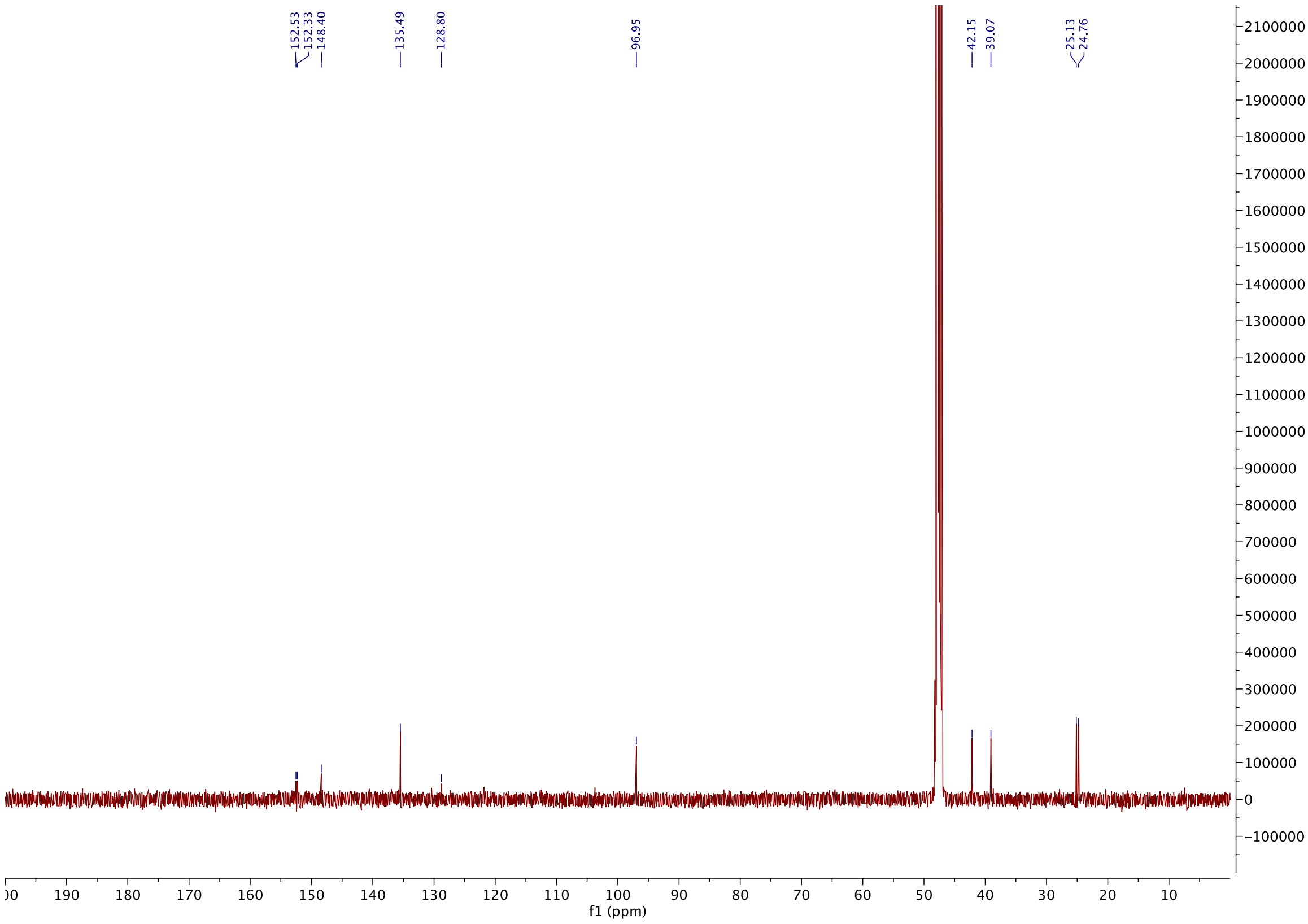

(17.1) ^{1}H NMR (500 MHz, $CDCl_3$)

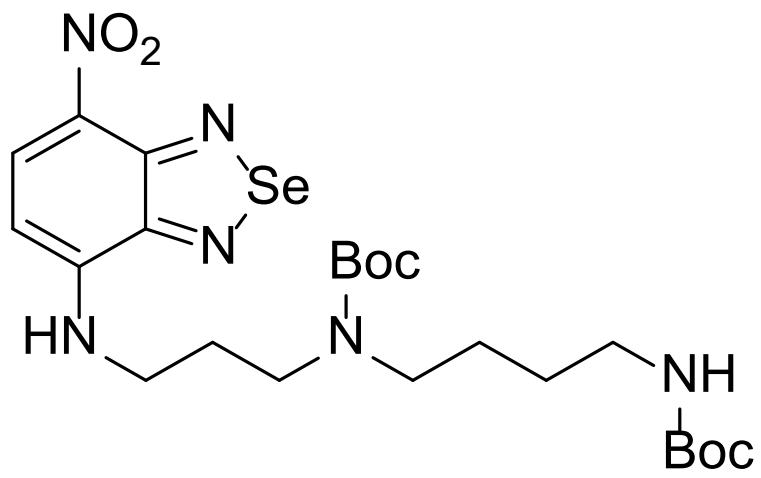

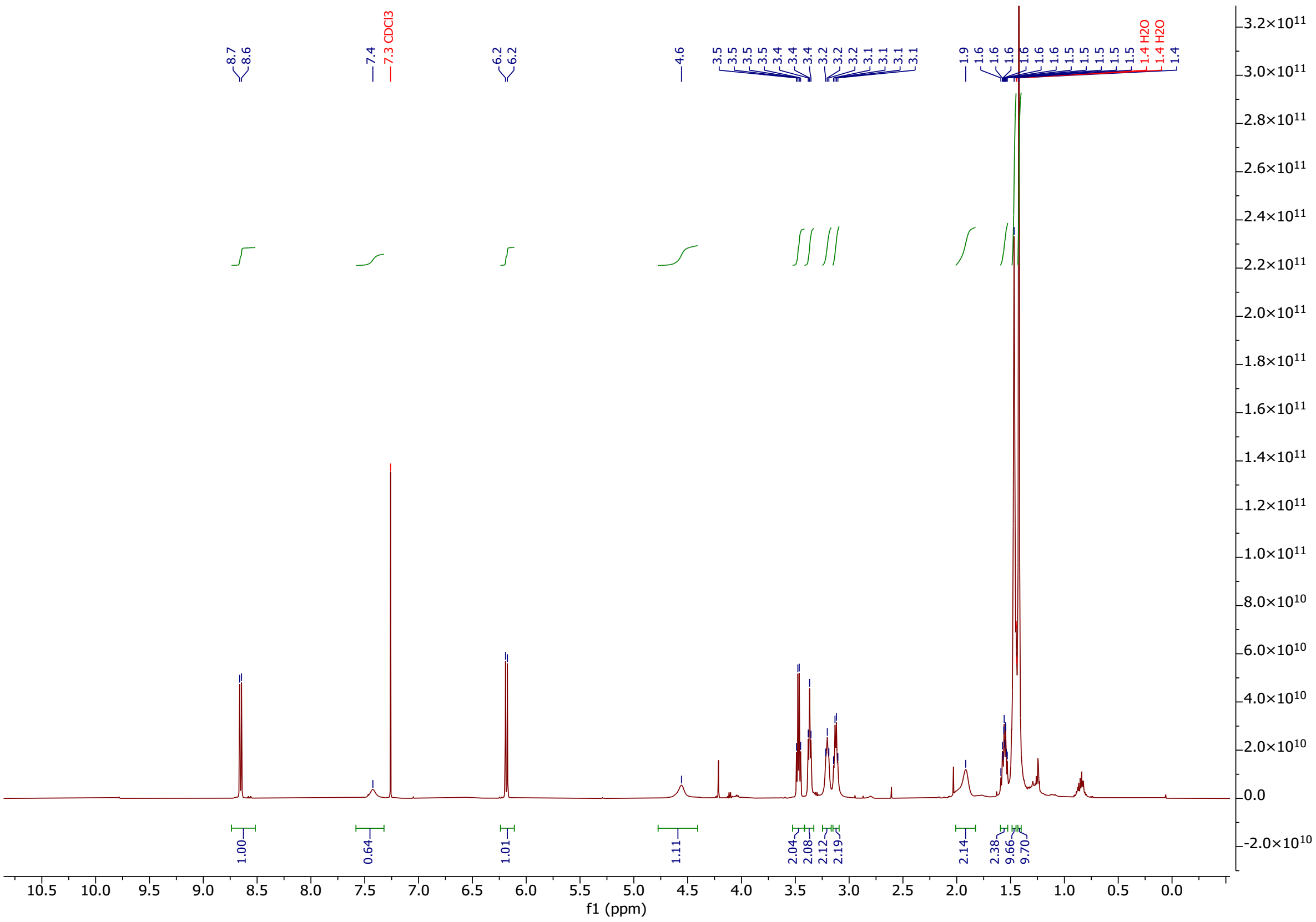

(17.1) ^{13}C NMR (126 MHz, $CDCl_3$)

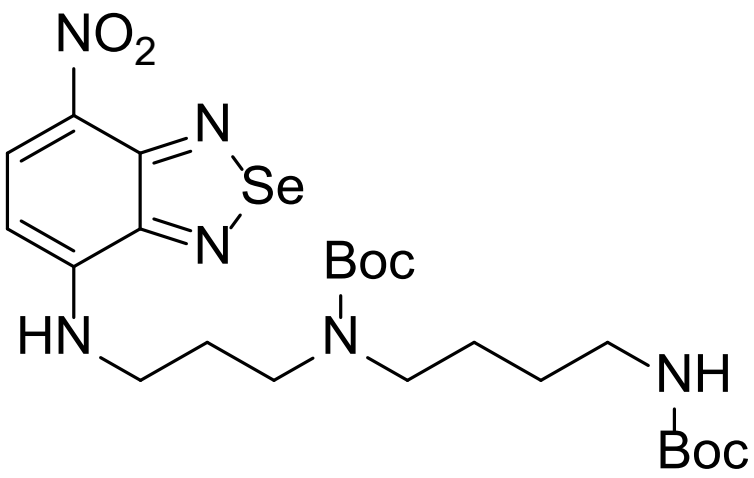

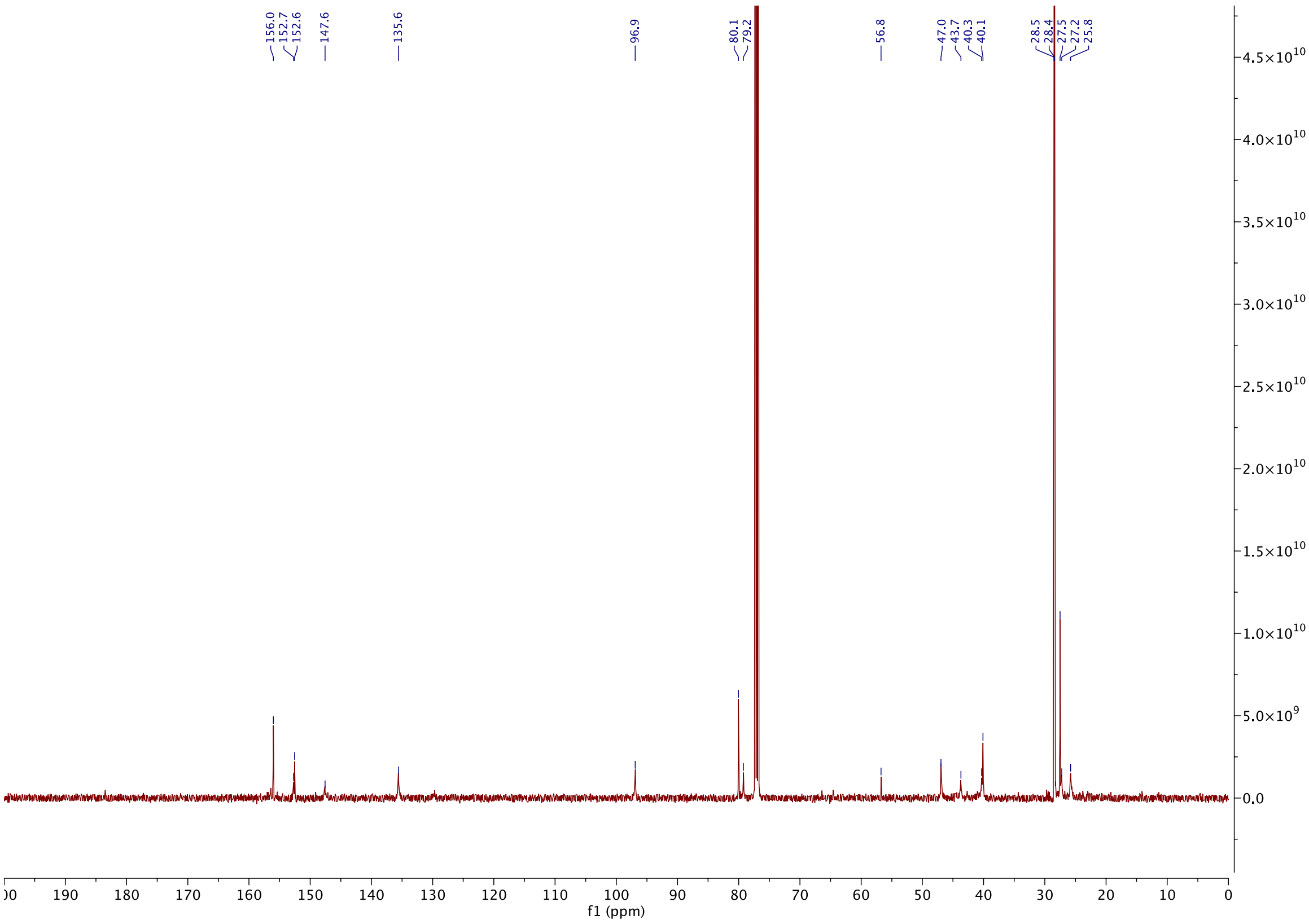

(17) ^{1}H NMR (500 MHz, CD_3OD)

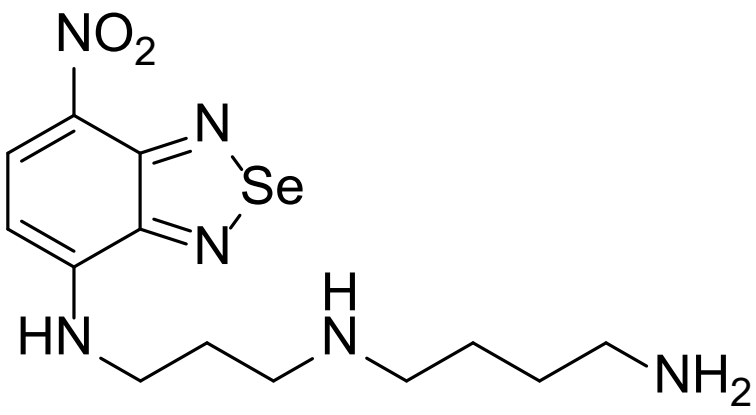

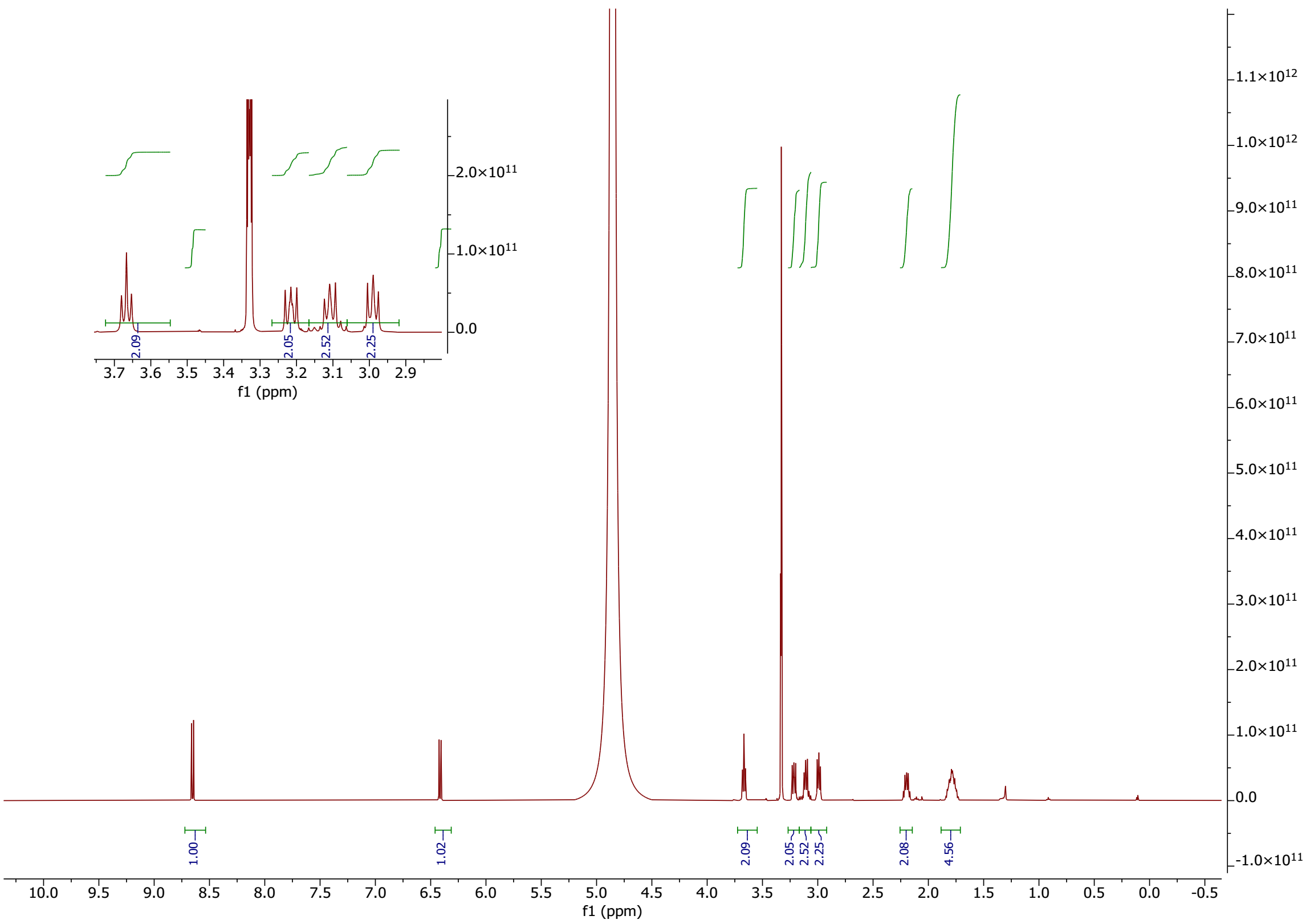

(17) ^{13}C NMR (126 MHz, CD_3OD)

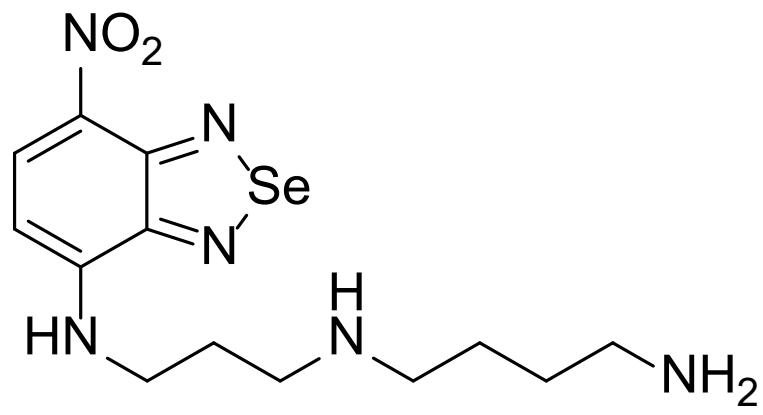

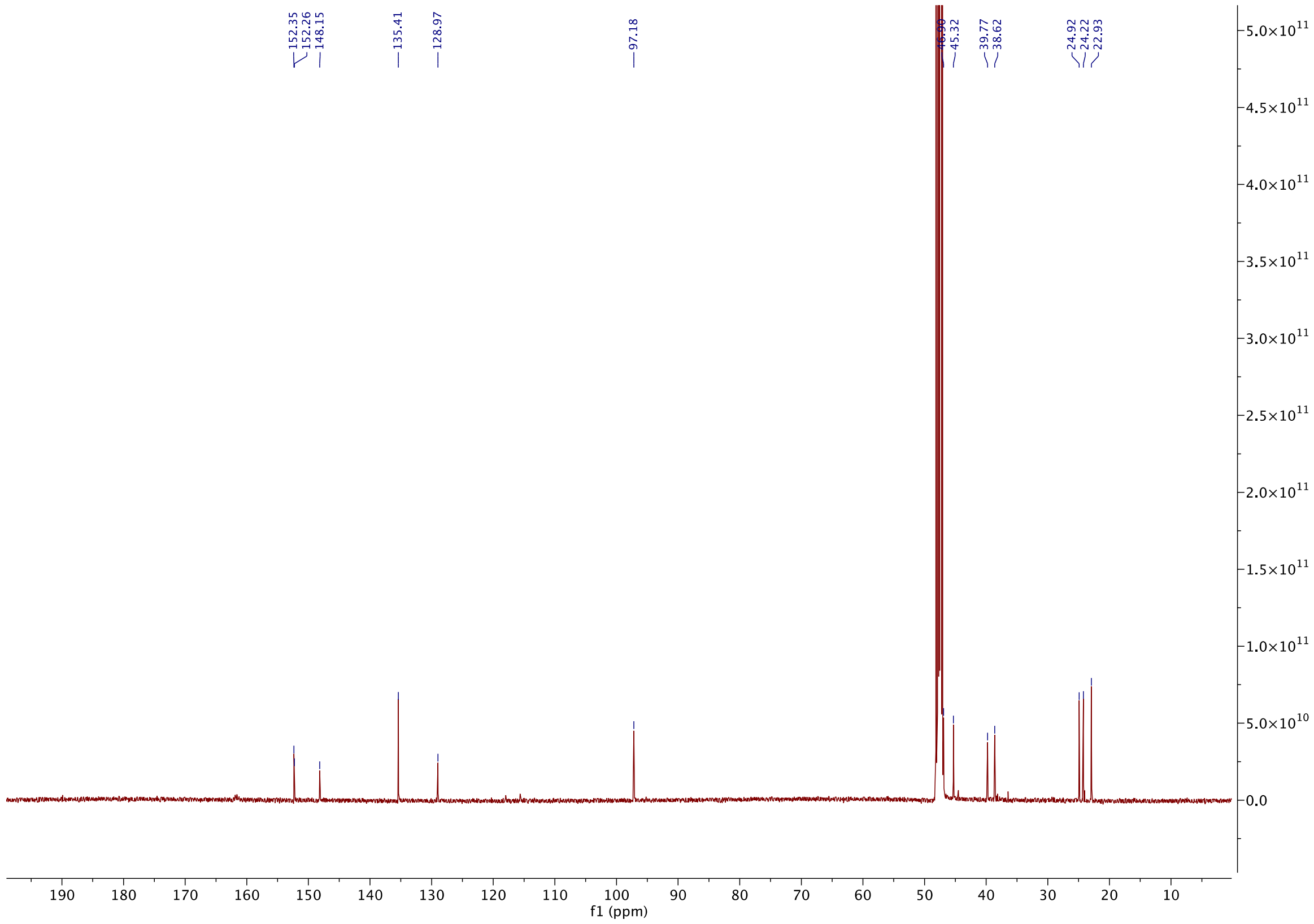

(18.3) ^{1}H NMR (500 MHz, CD_3OD)

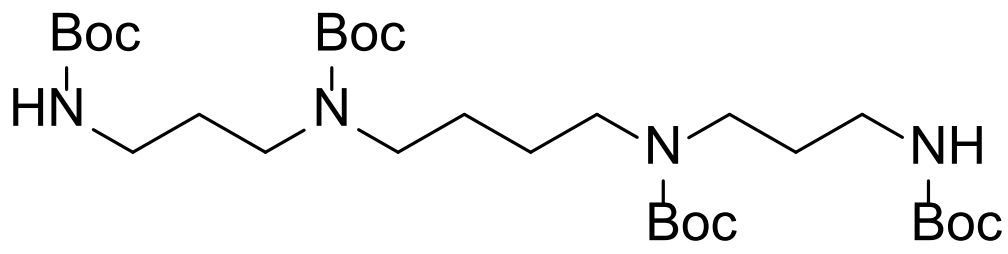

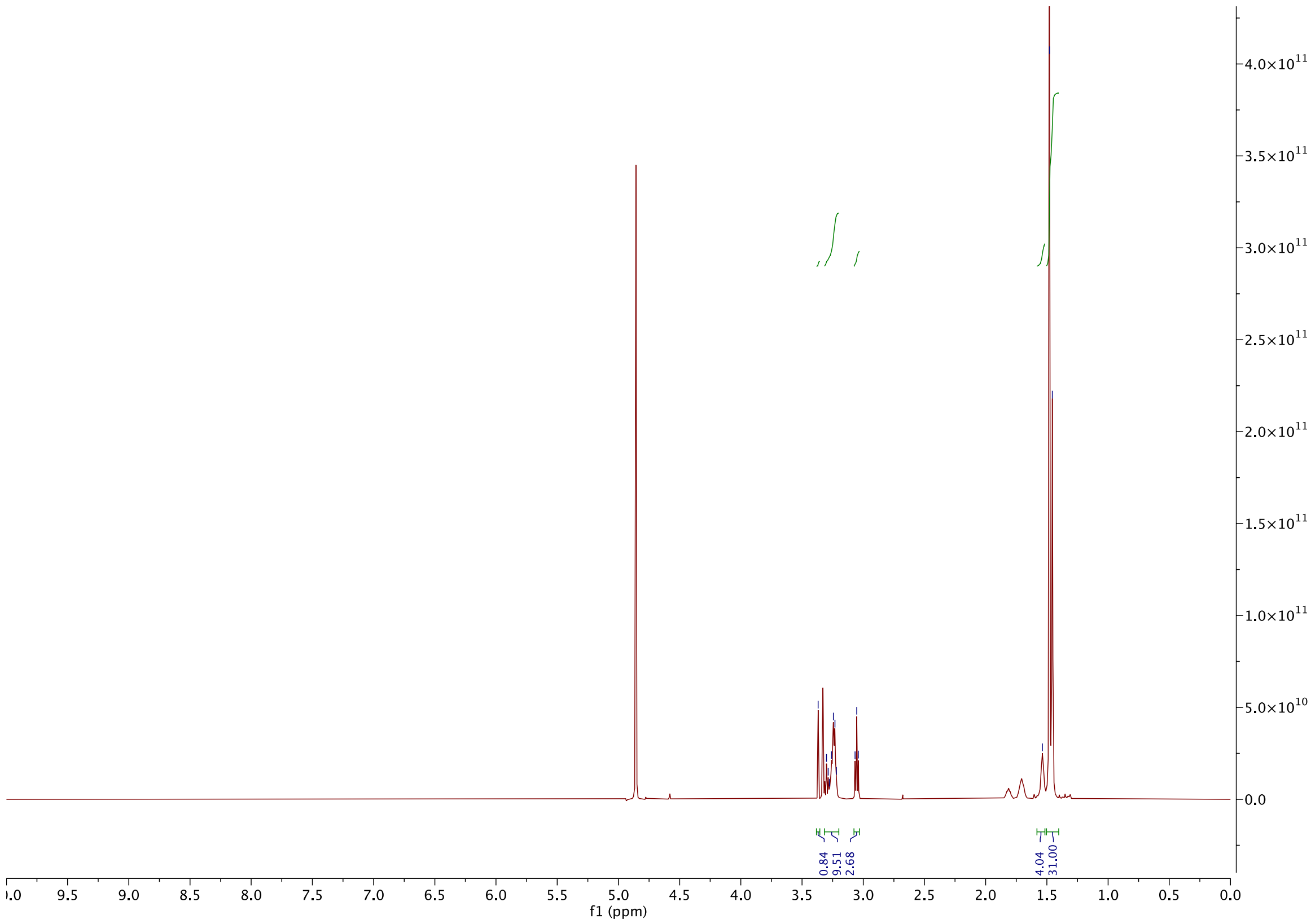

(18.3) ^{13}C NMR (126 MHz, CD_3OD)

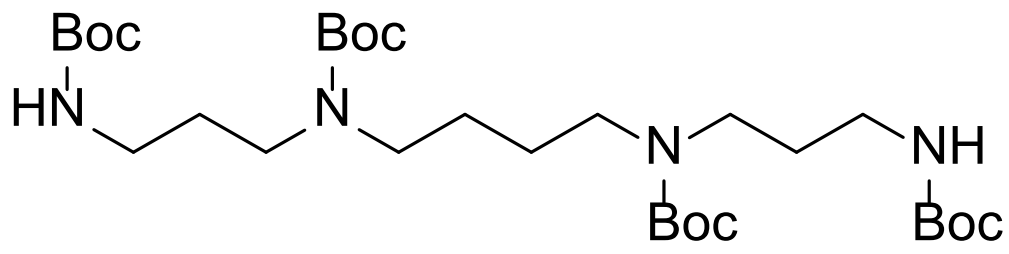

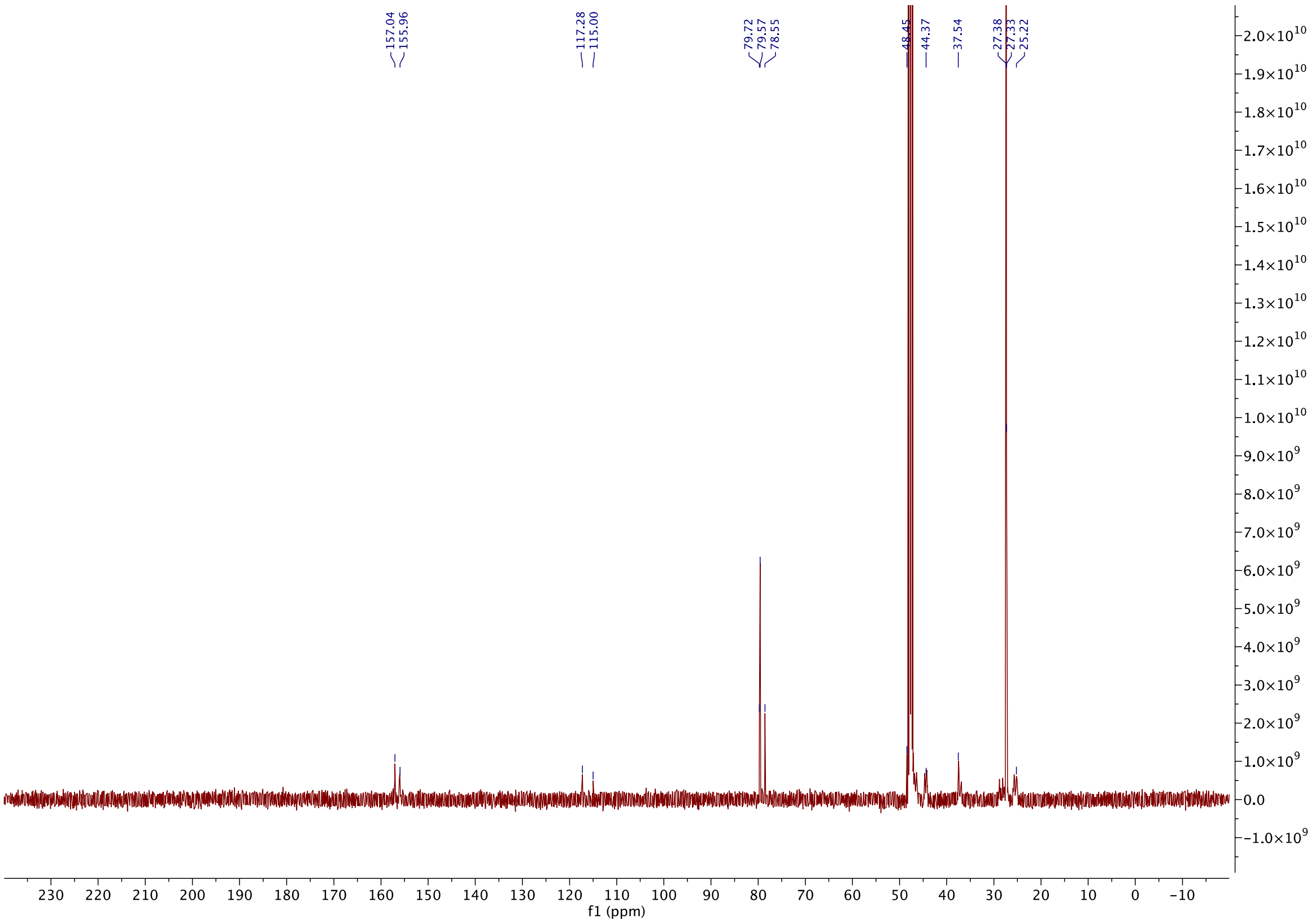

(18.4) ^{1}H NMR (500 MHz, CD_3OD)

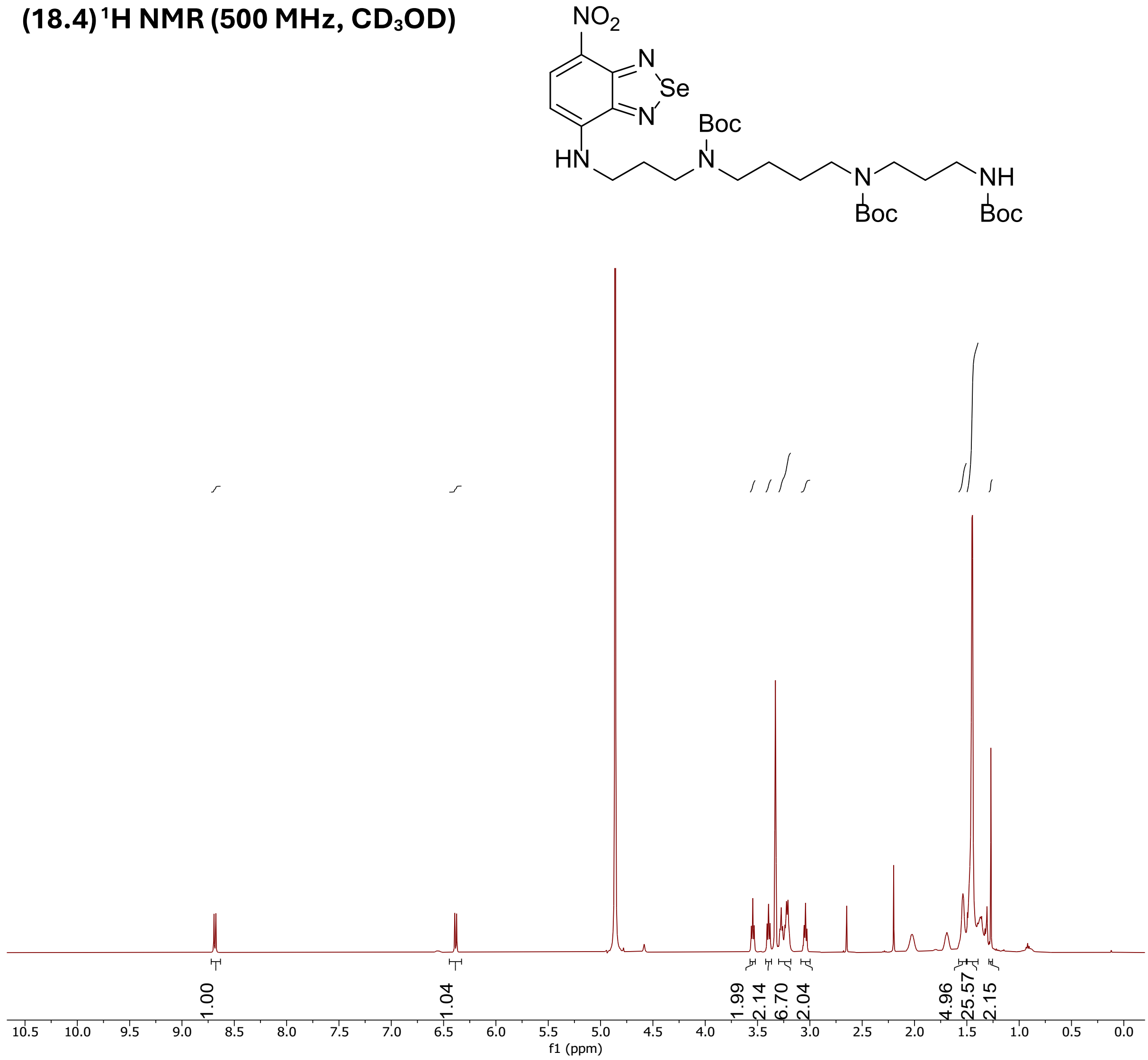

(18.4) ^{13}C NMR (126 MHz, CD_3OD)

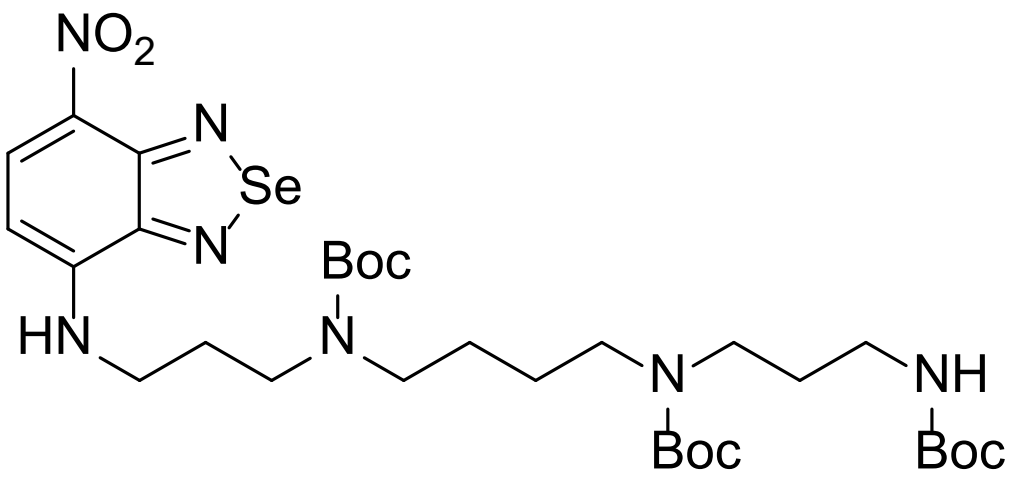

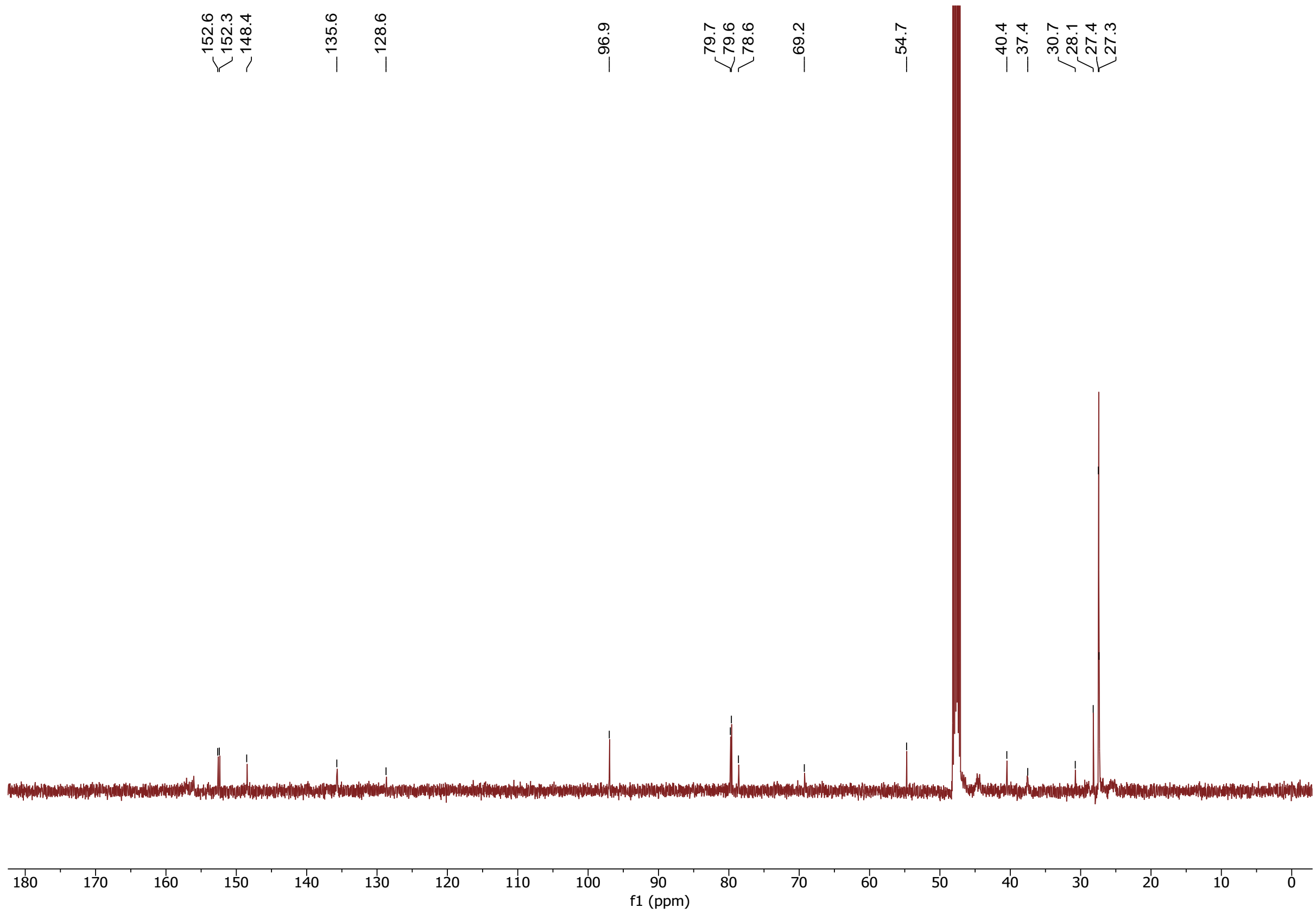

(18) ^{1}H NMR (500 MHz, DMSO-d$_6$)

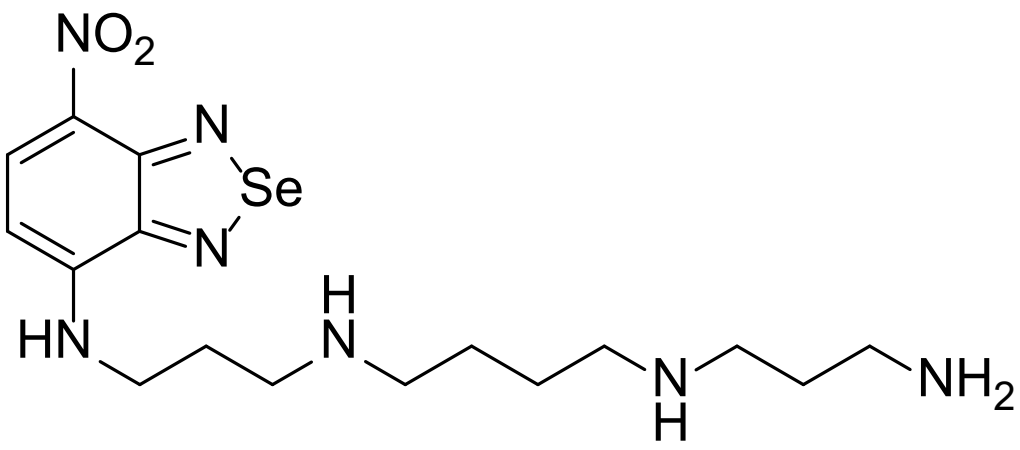

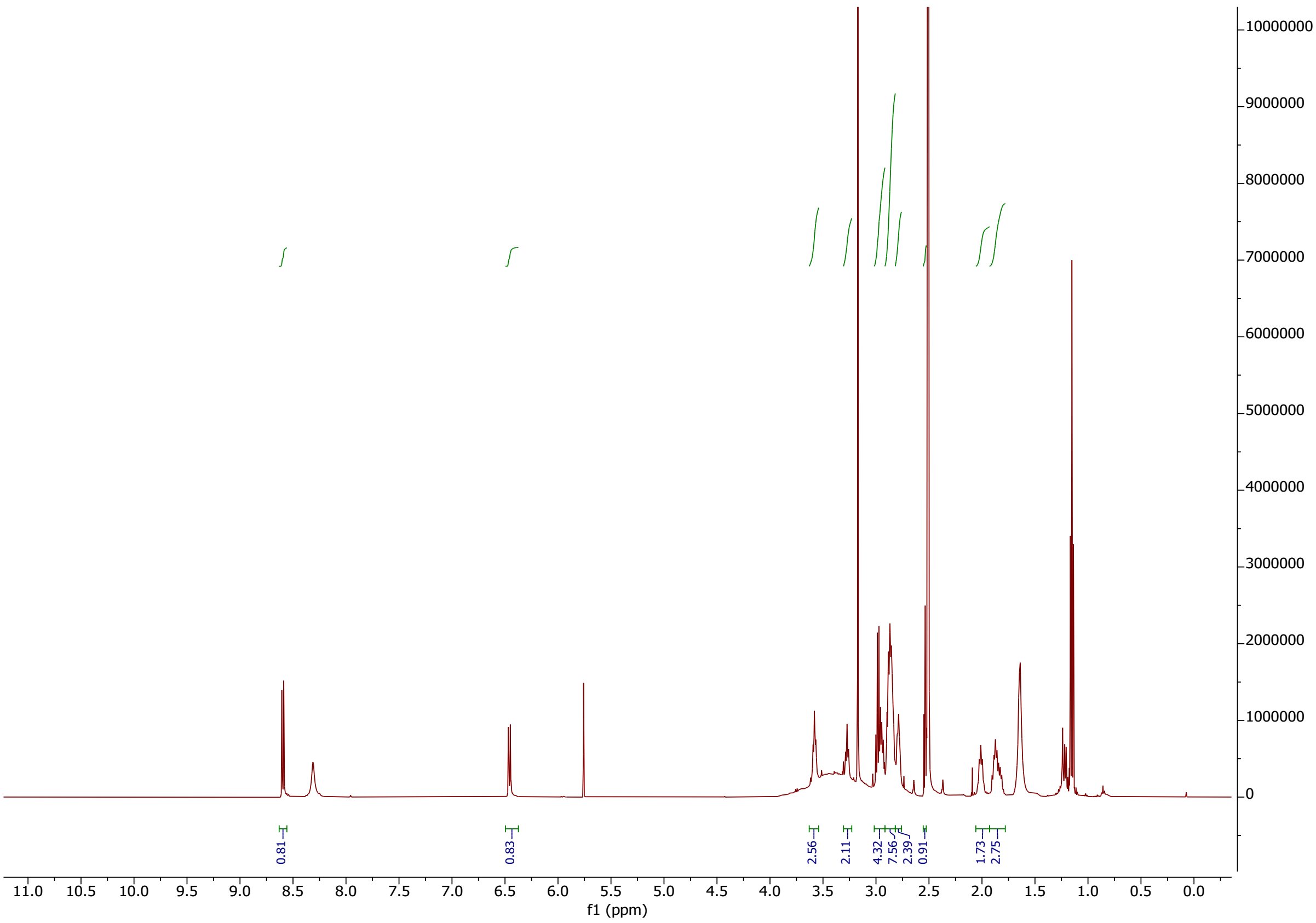

(19) ^1H NMR (500 MHz, D_2O)

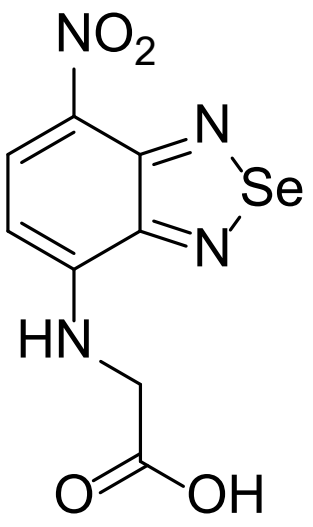

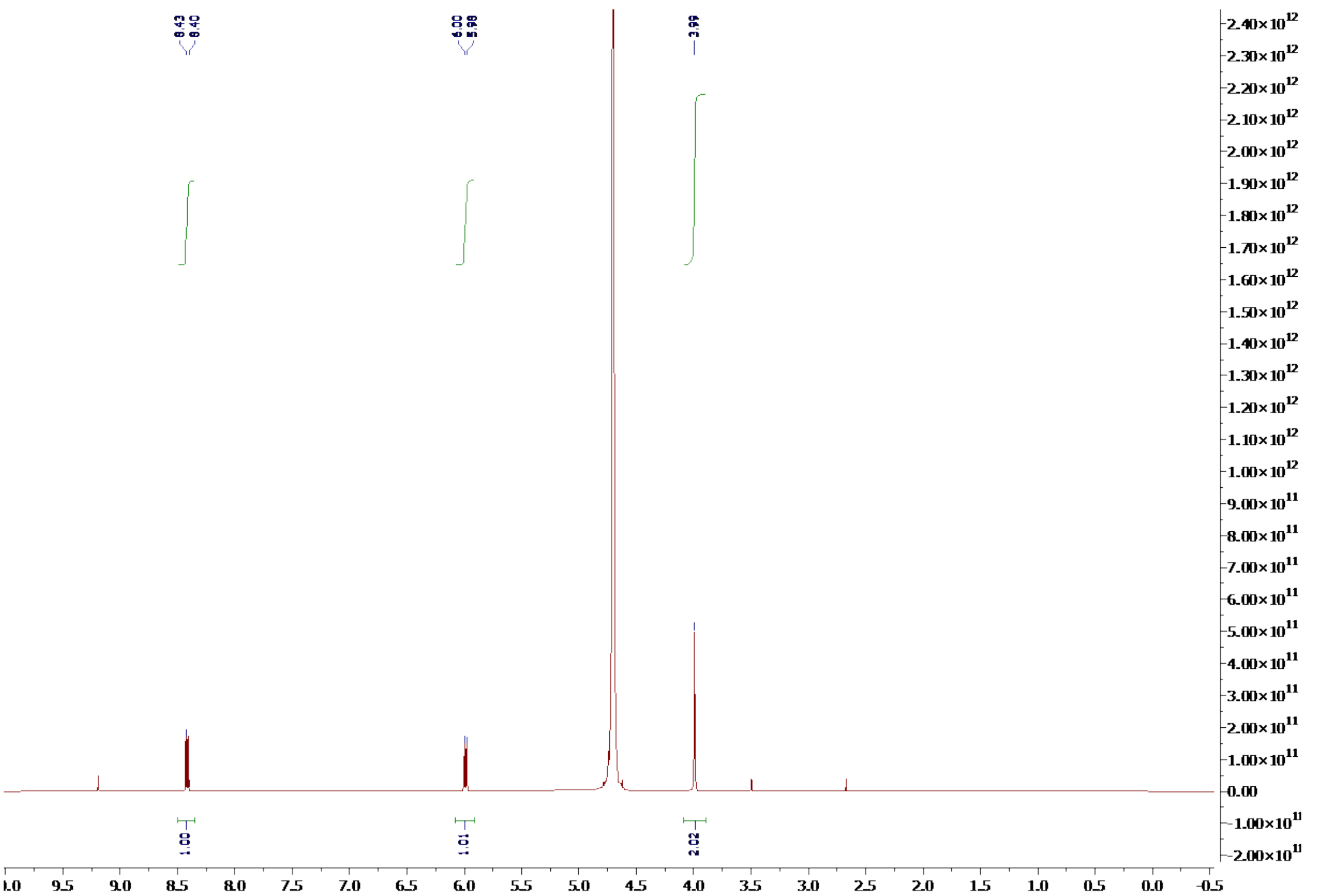

(19) ^{13}C NMR (126 MHz, D_2O)

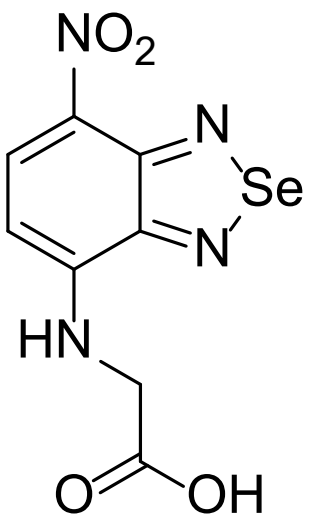

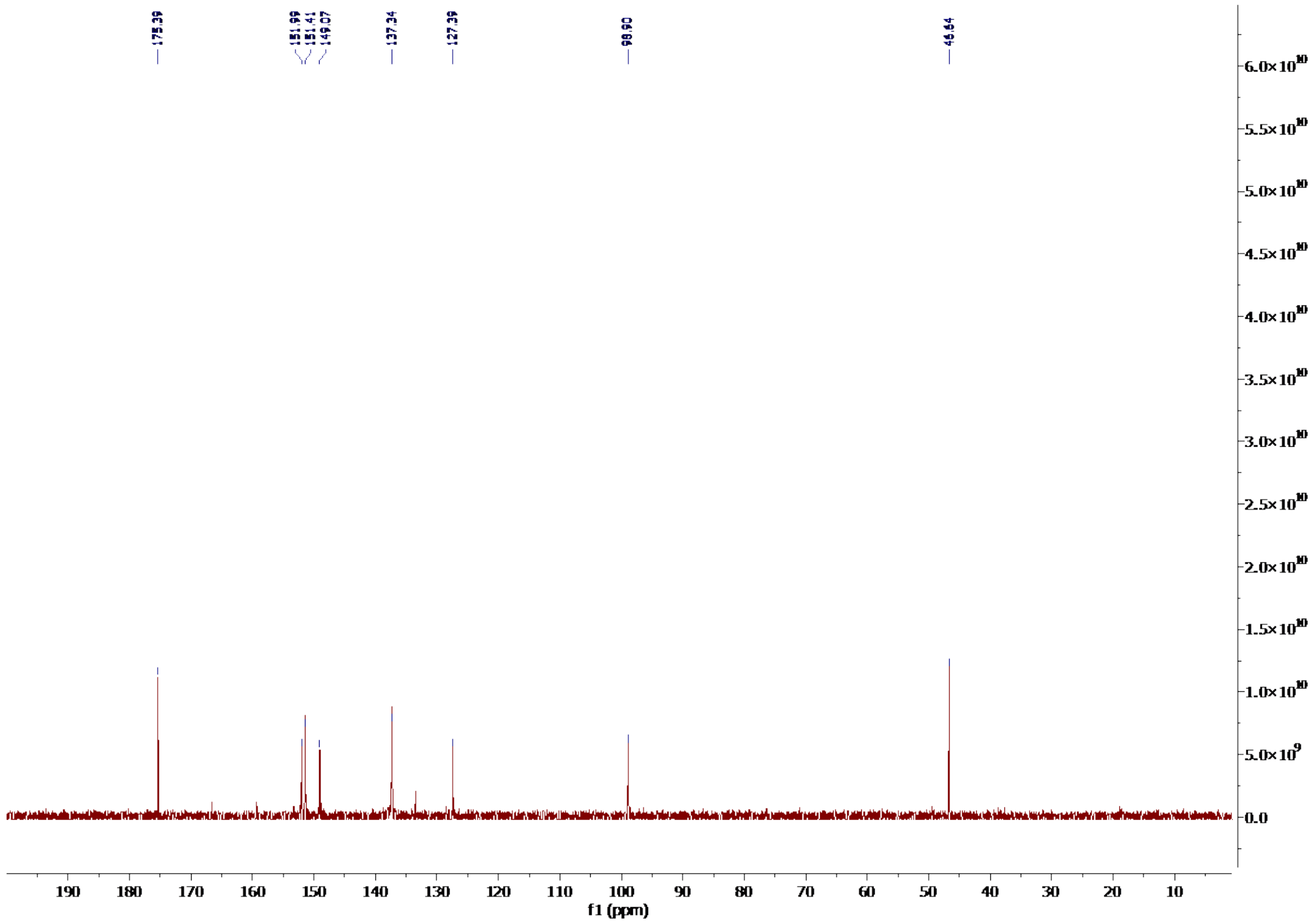

(20) ^{1}H NMR (500 MHz, D_2O)

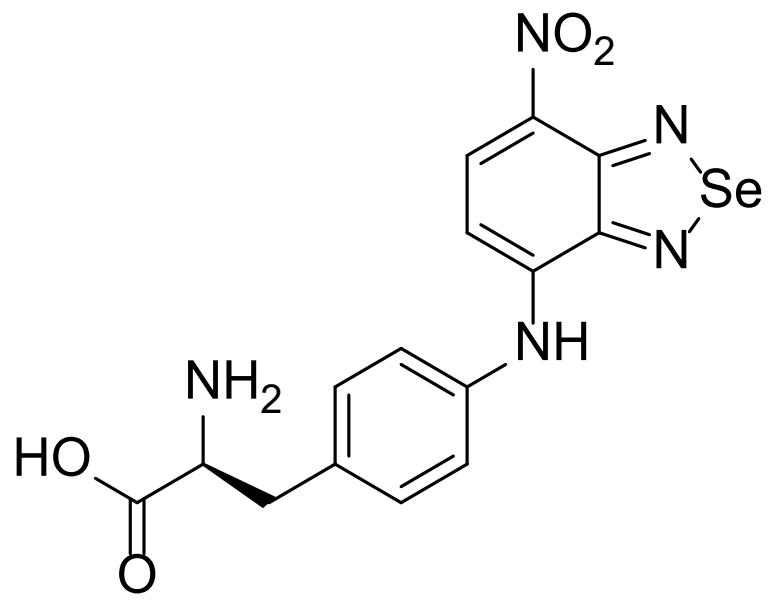

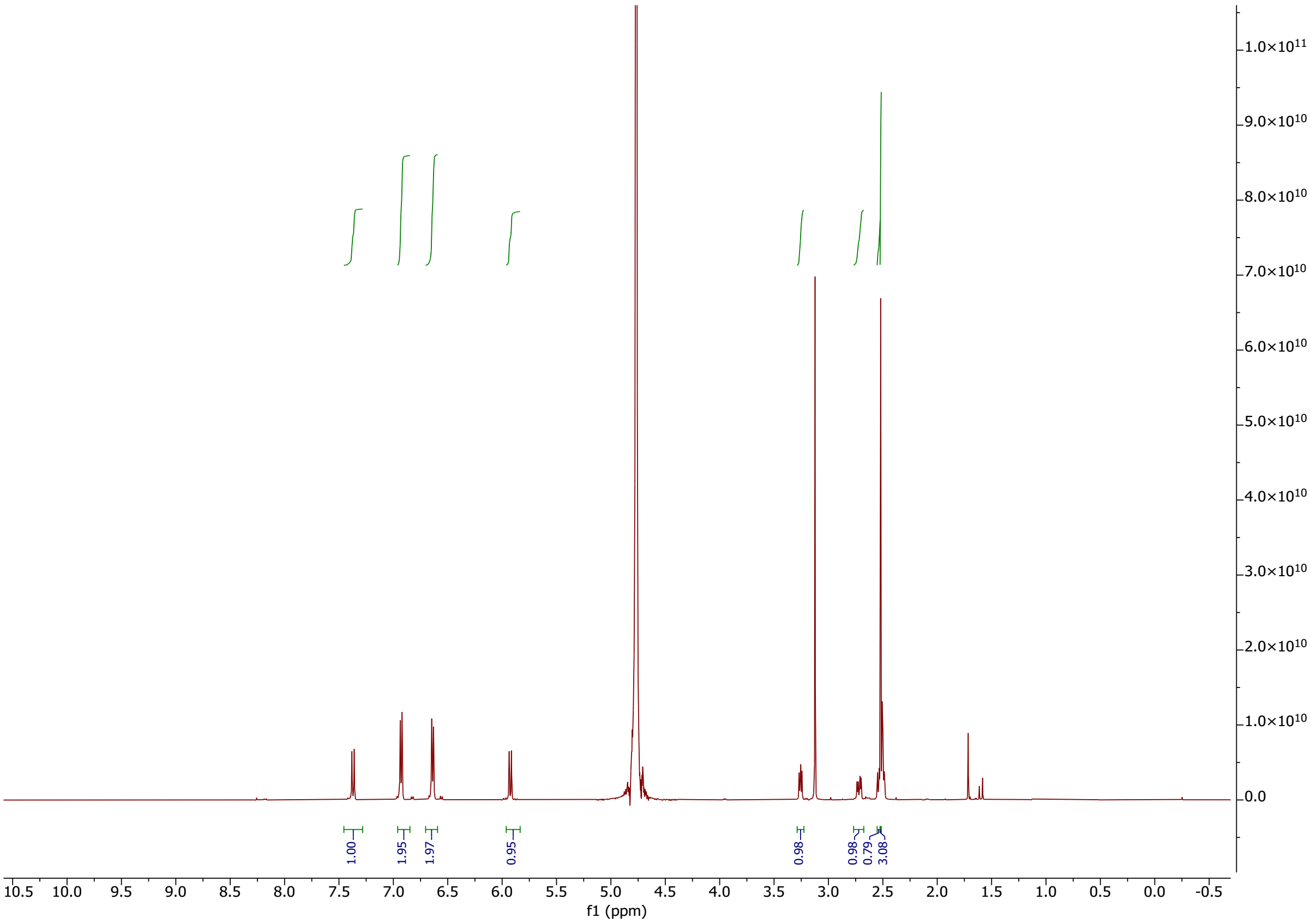

(20) ^{13}C NMR (126 MHz, D_2O)

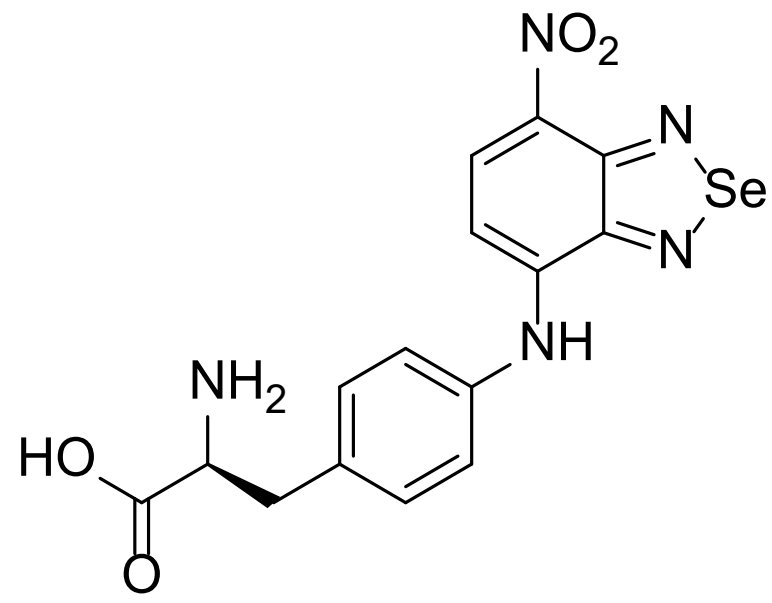

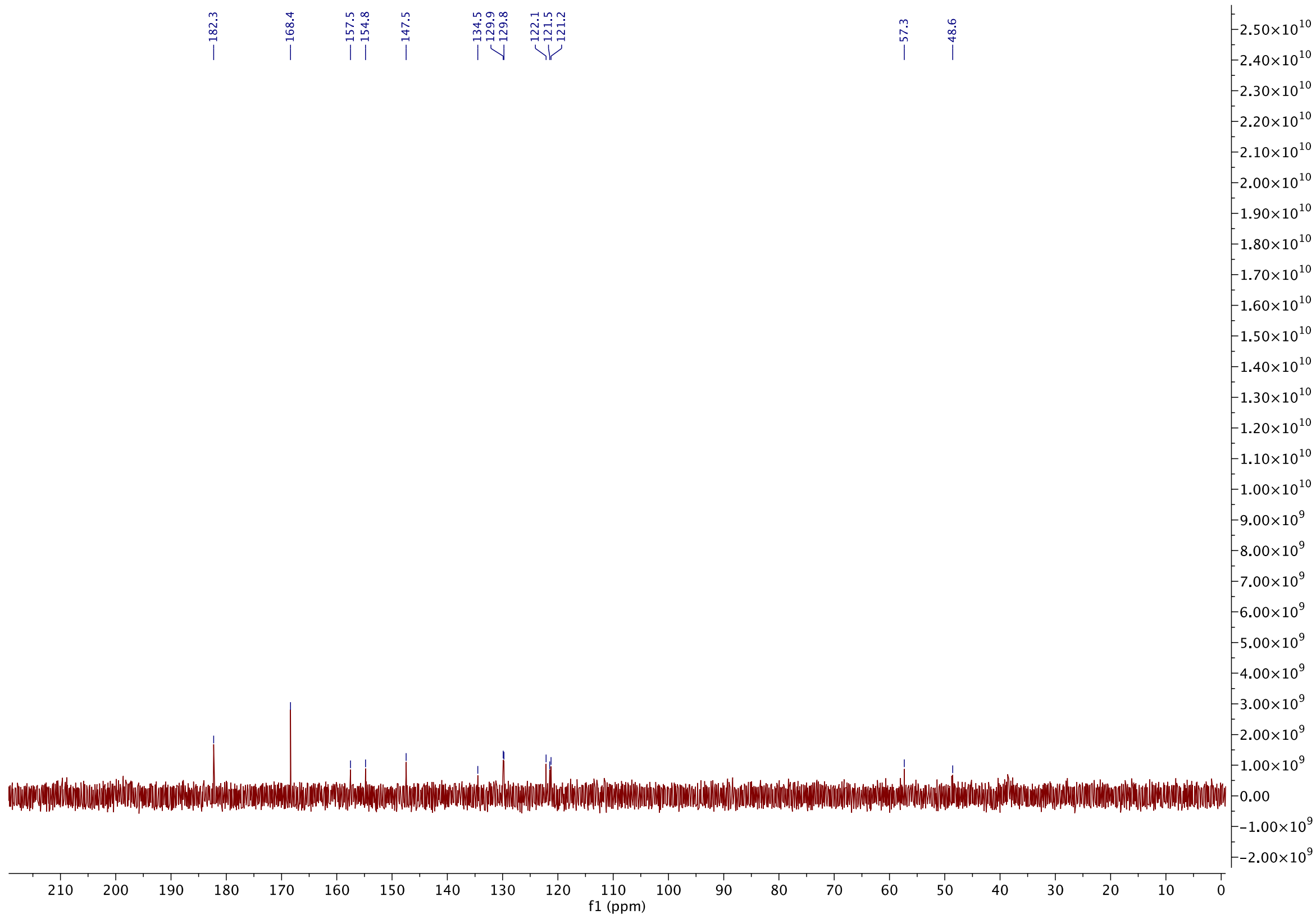

(23.1) ¹H NMR (500 MHz, $CDCl_3$)

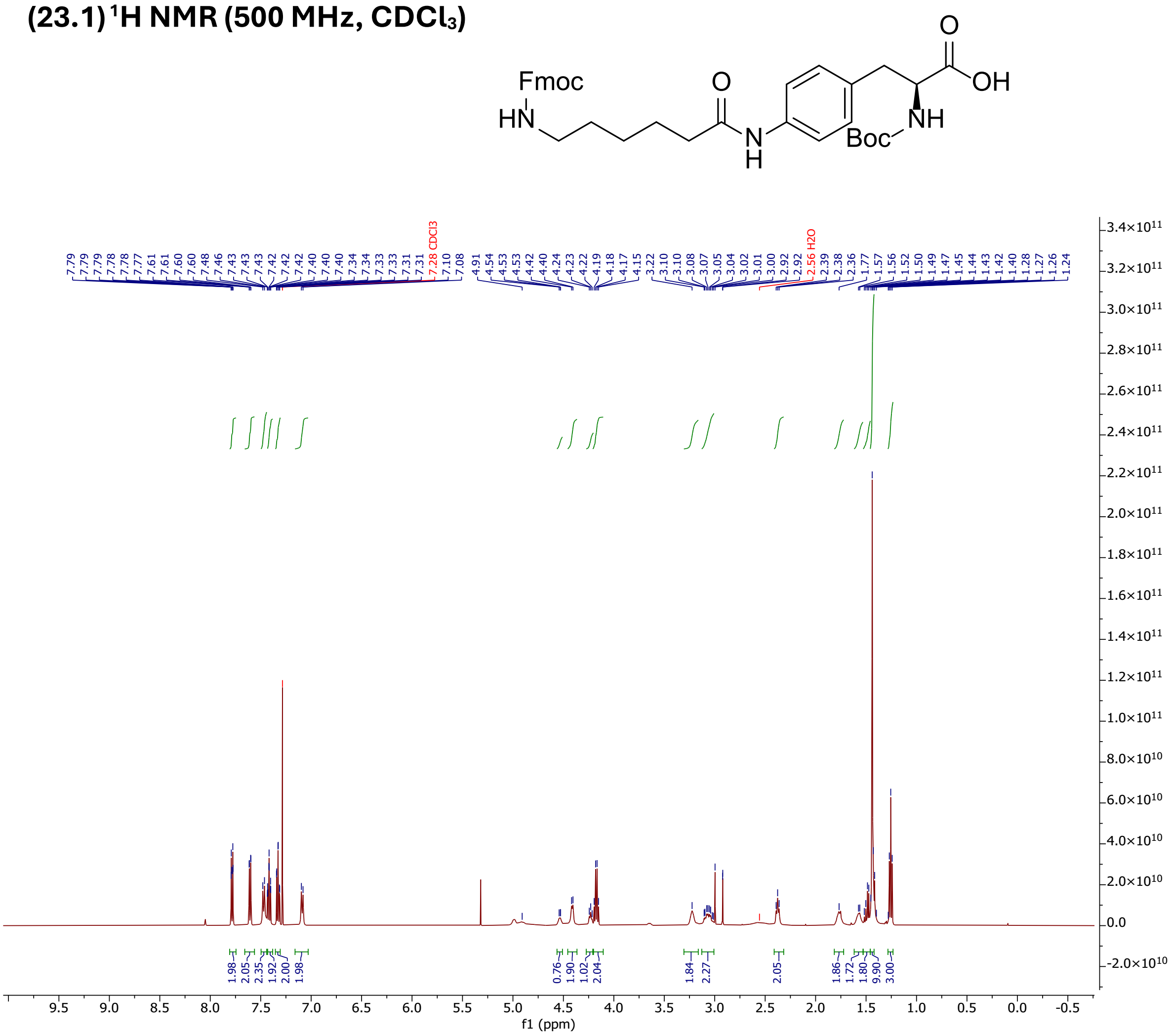

(23.1) ^{13}C NMR (126 MHz, $CDCl_3$)

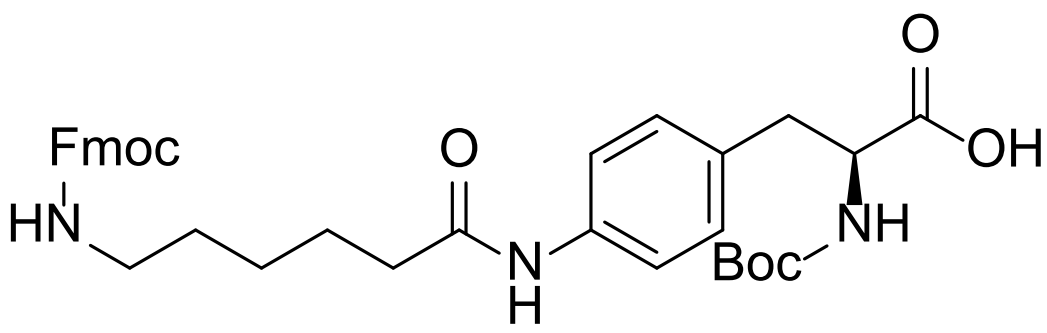

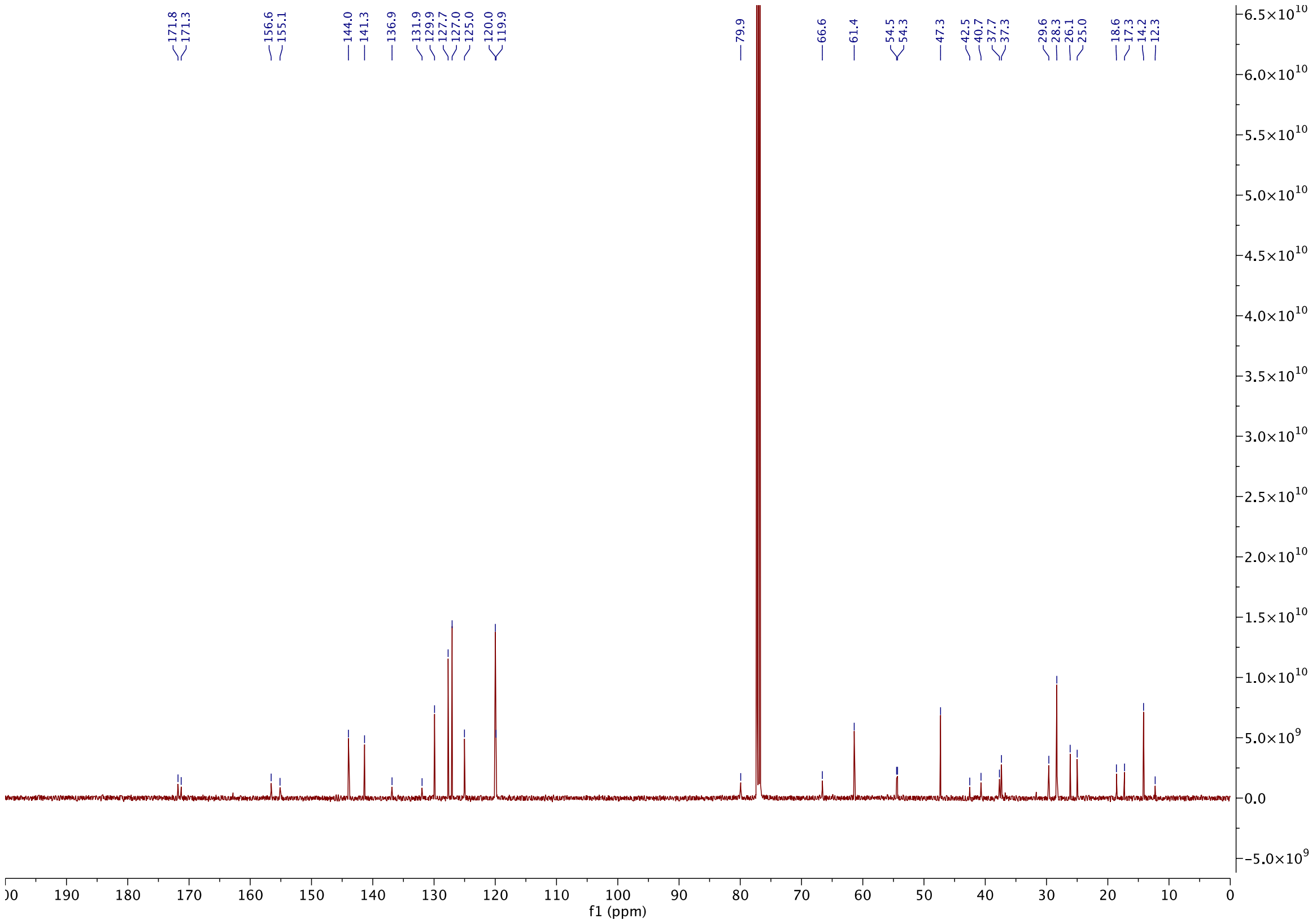

(23.2) ^{1}H NMR (500 MHz, $CDCl_3$)

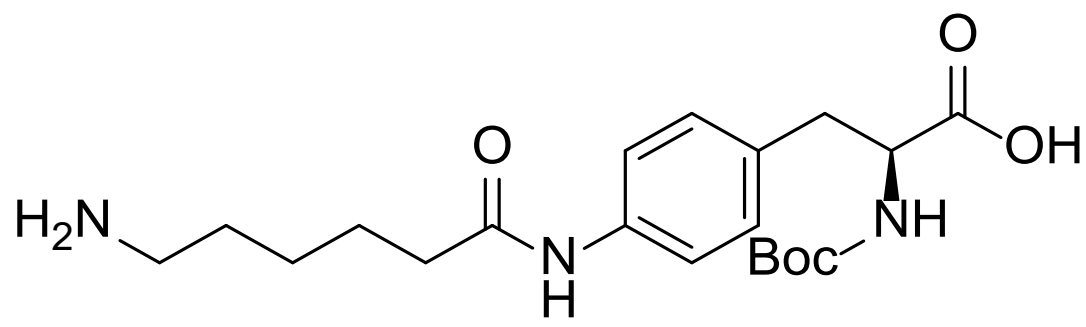

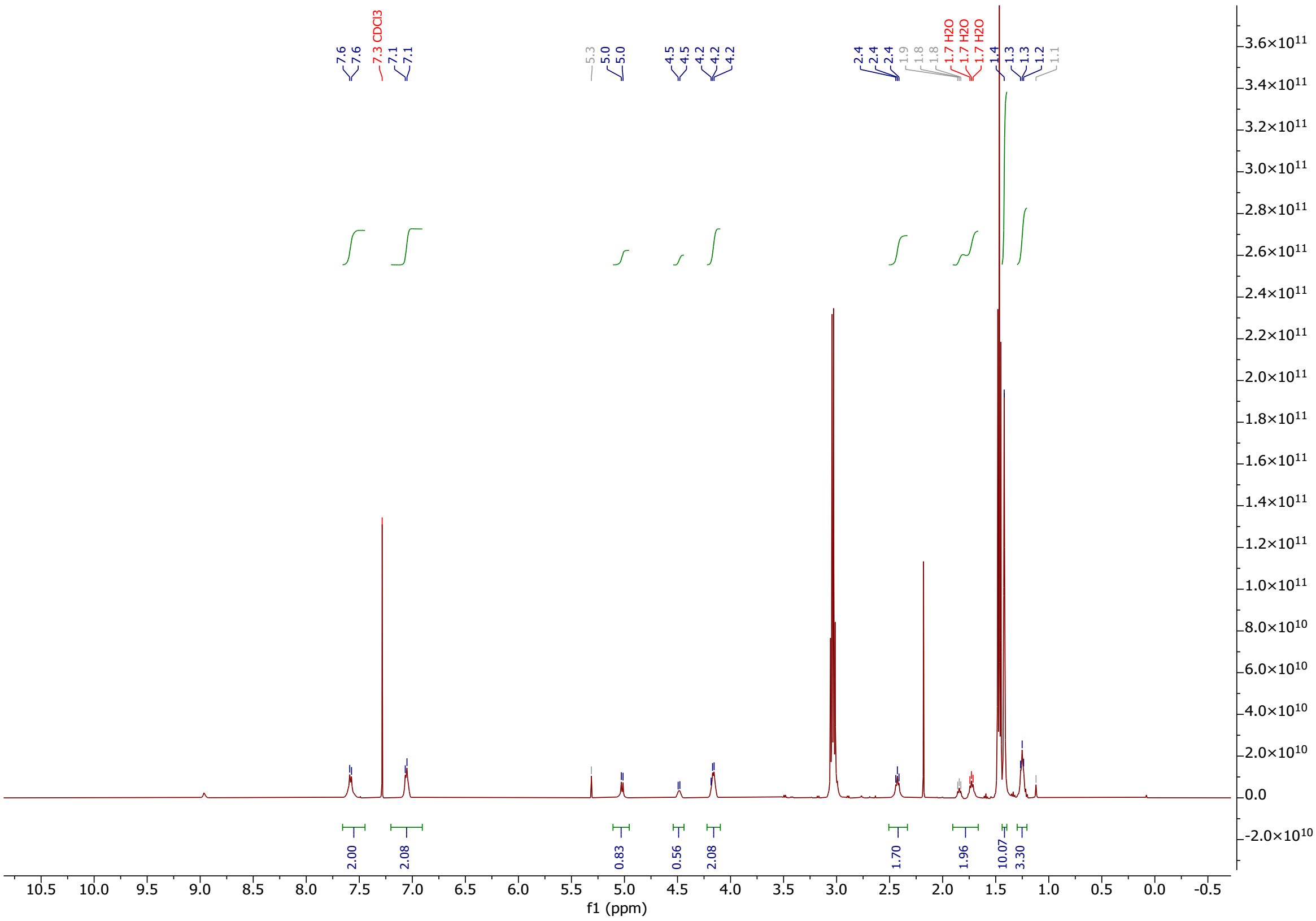

(23.2) ^{13}C NMR (126 MHz, $CDCl_3$)

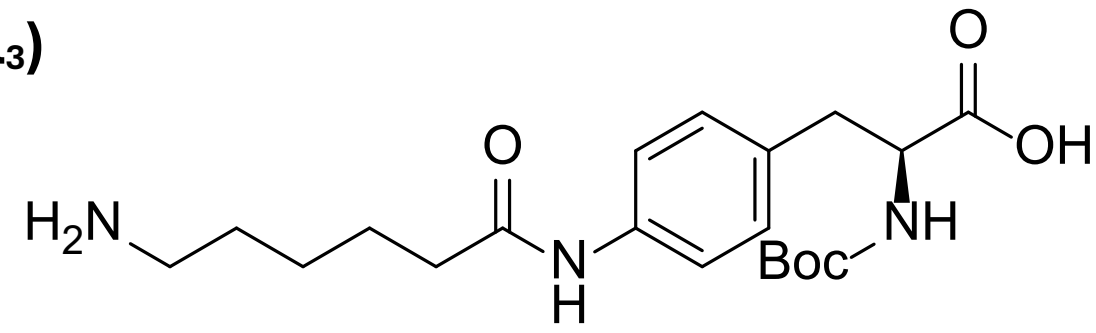

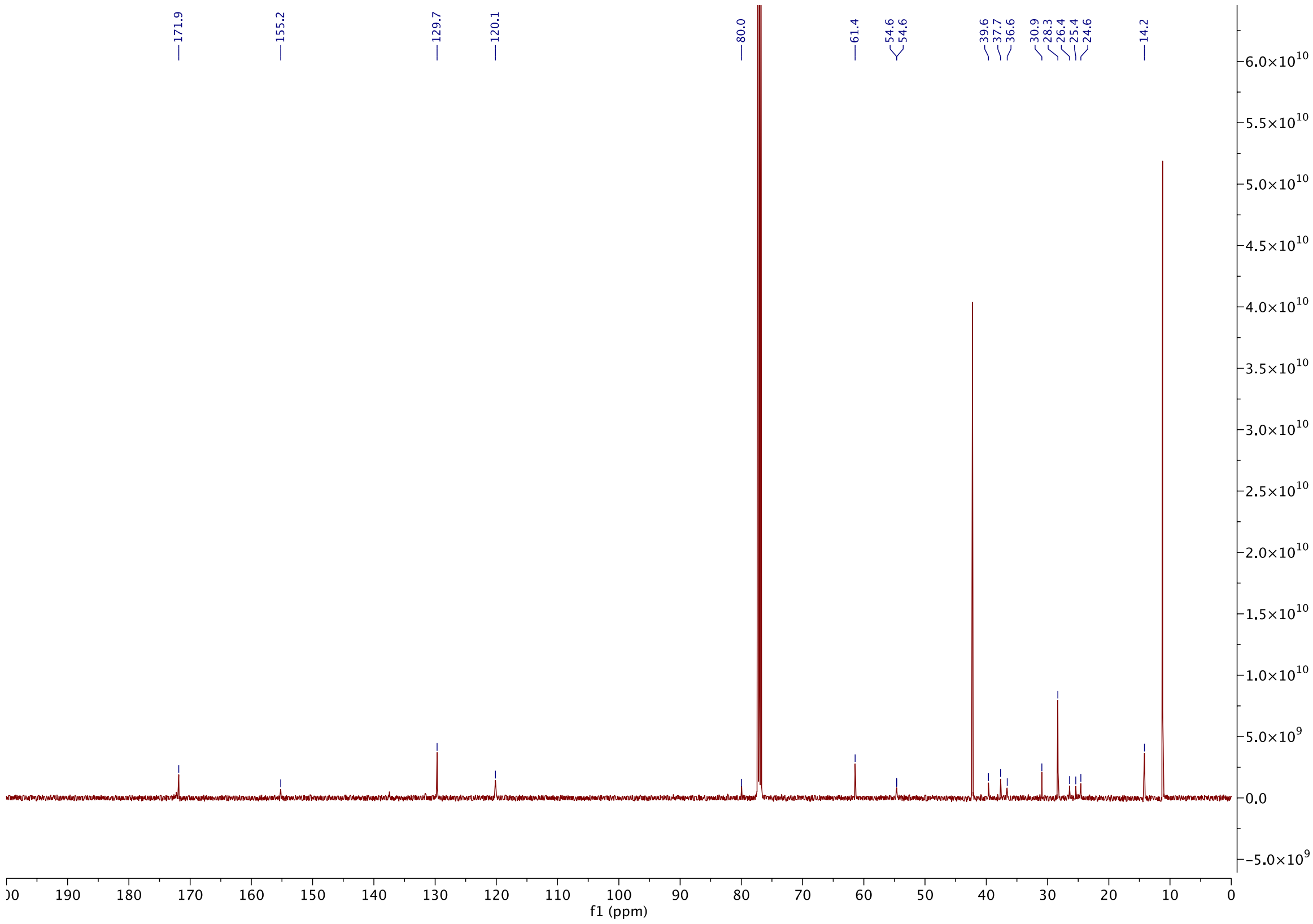

(23.3) ¹H NMR (500 MHz, $CDCl_3$)

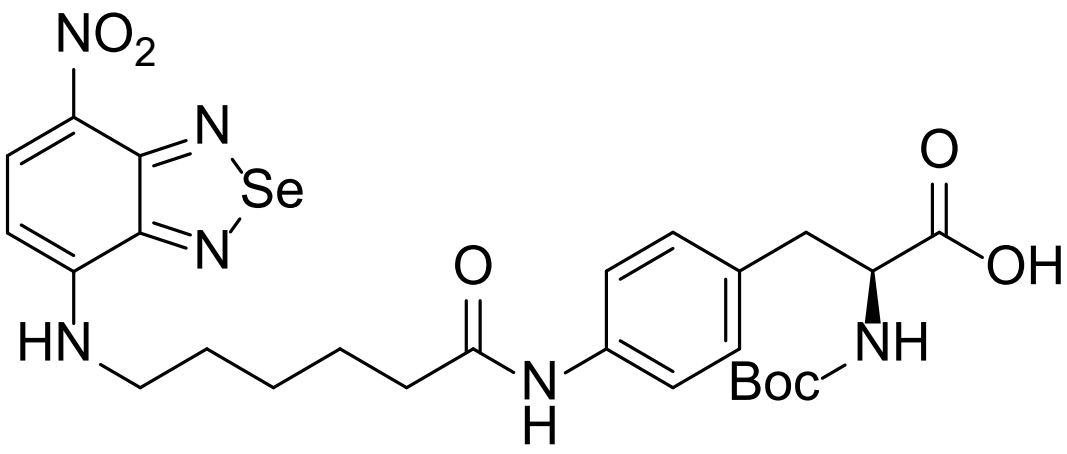

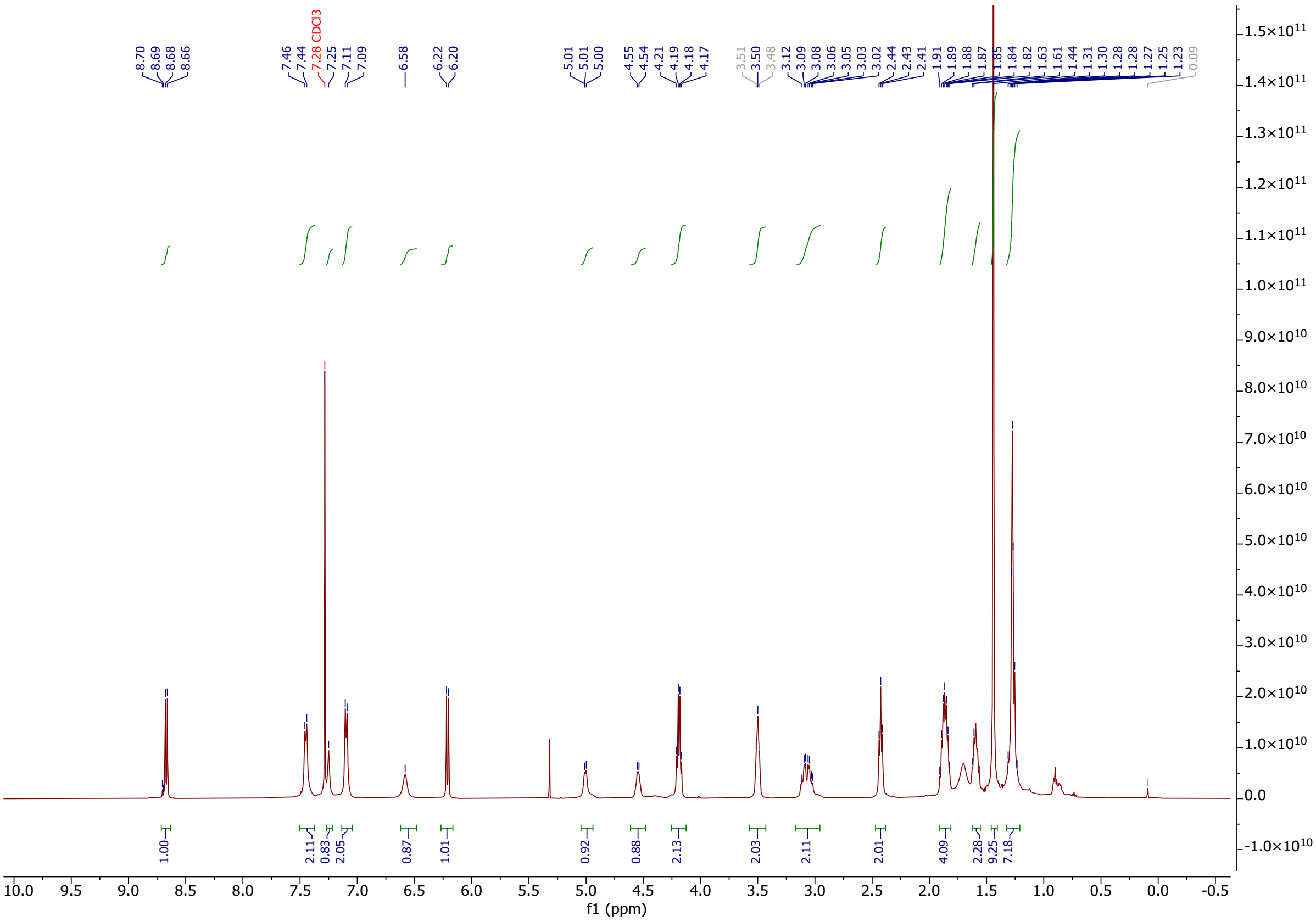

(23.3) ^{13}C NMR (126 MHz, $CDCl_3$)

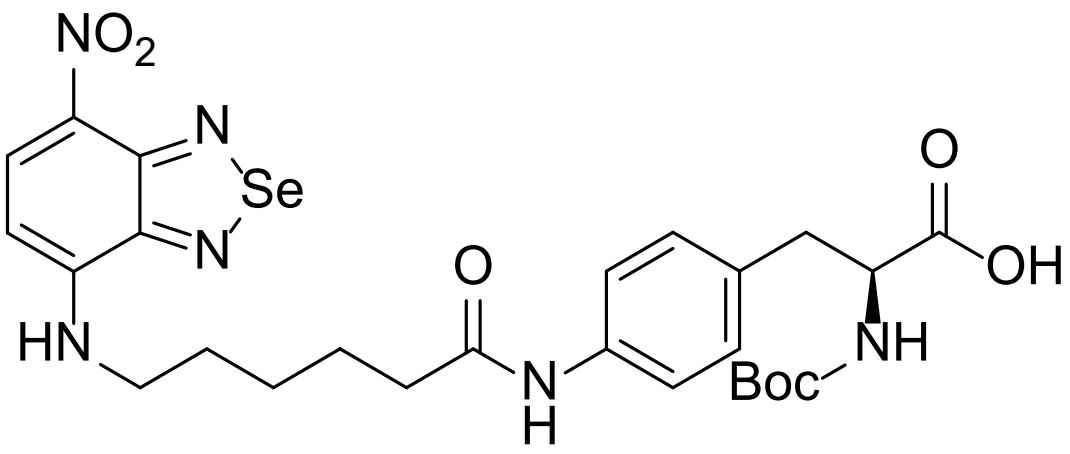

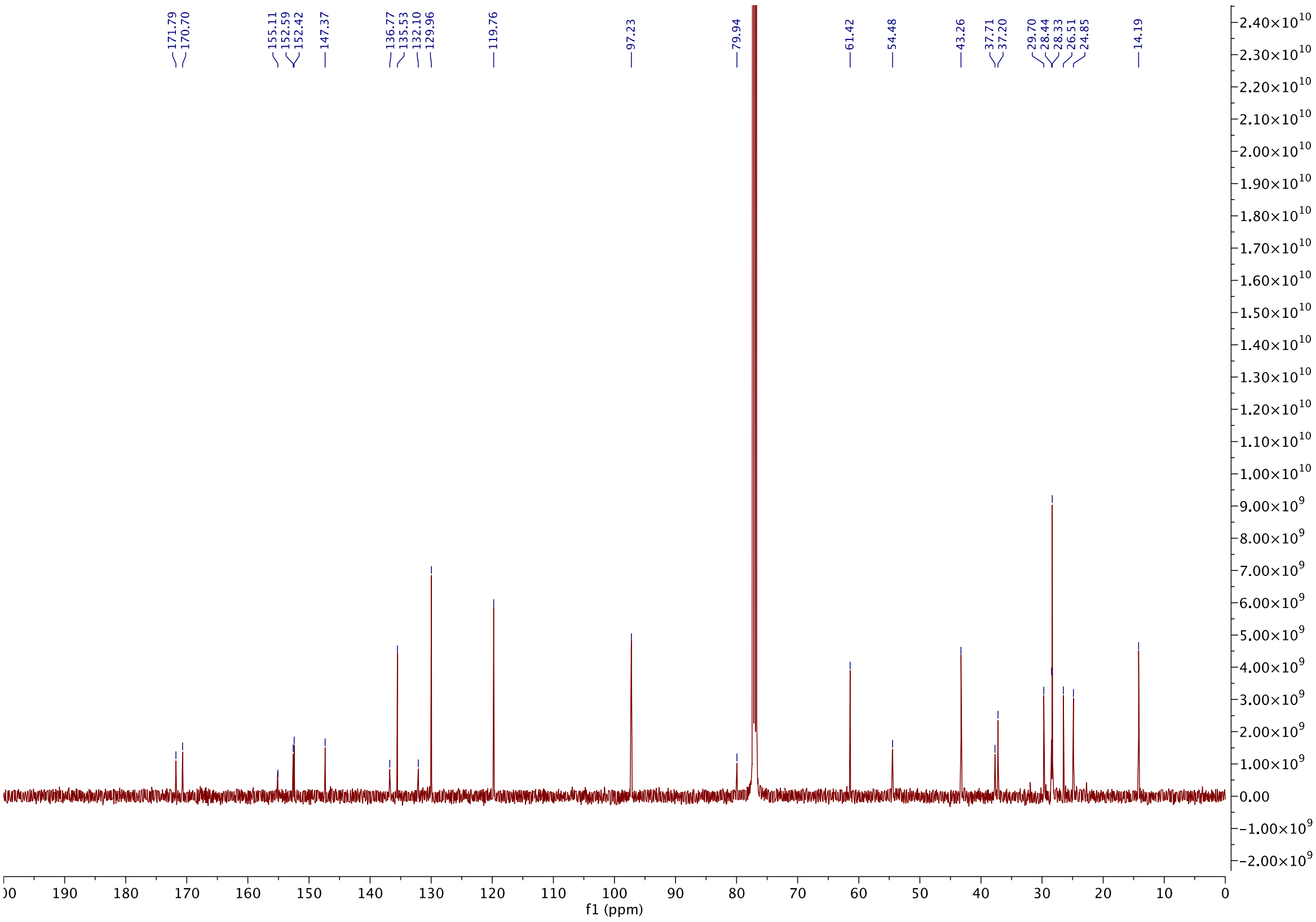

(23) ^{1}H NMR (500 MHz, DMSO-d_6)

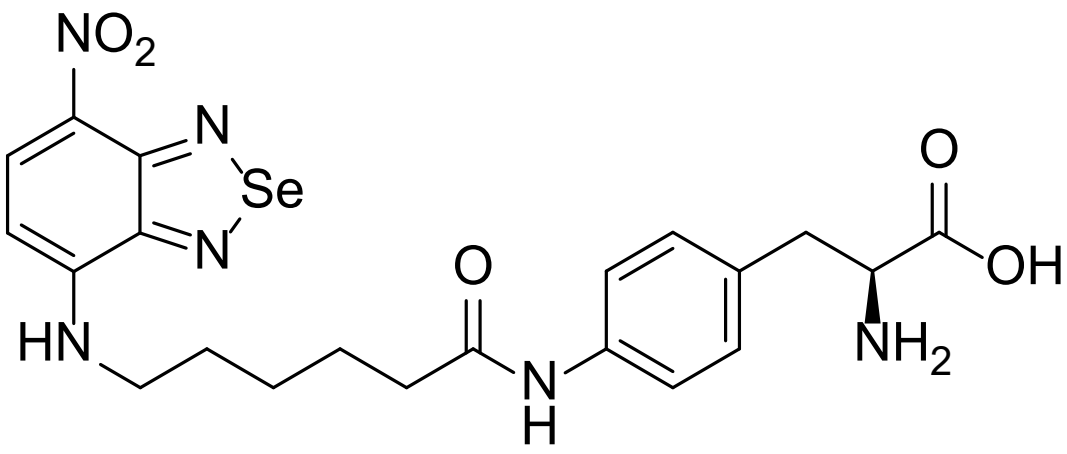

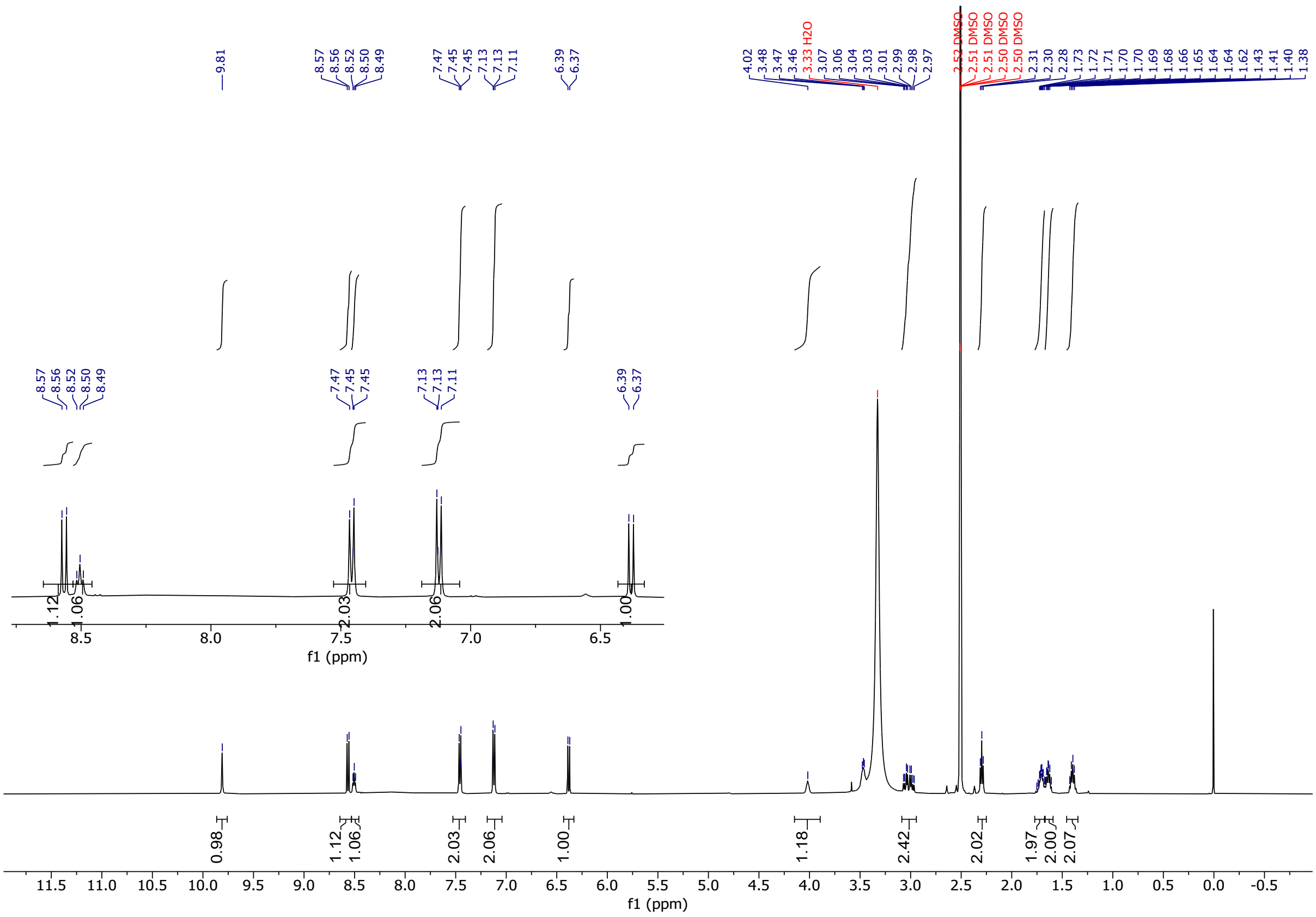

(23) ^{13}C NMR (126 MHz, DMSO-d$_6$)

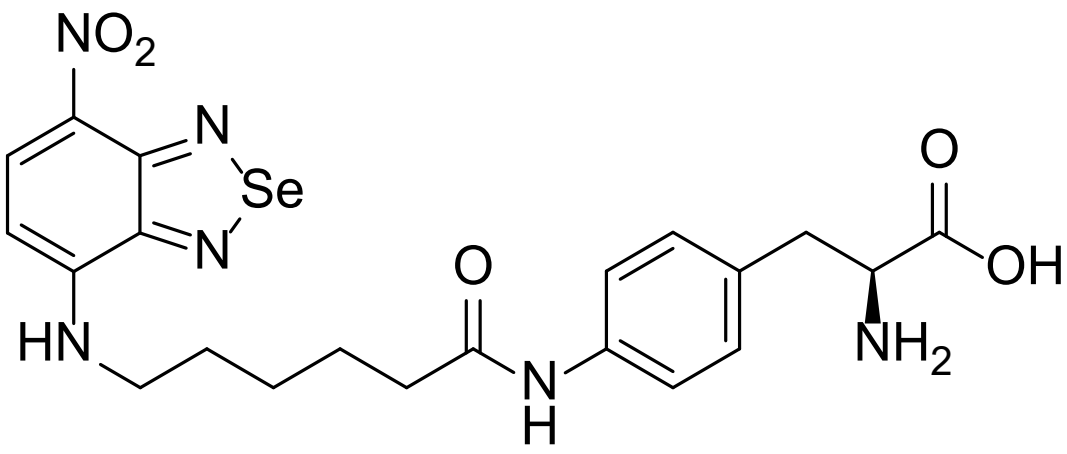

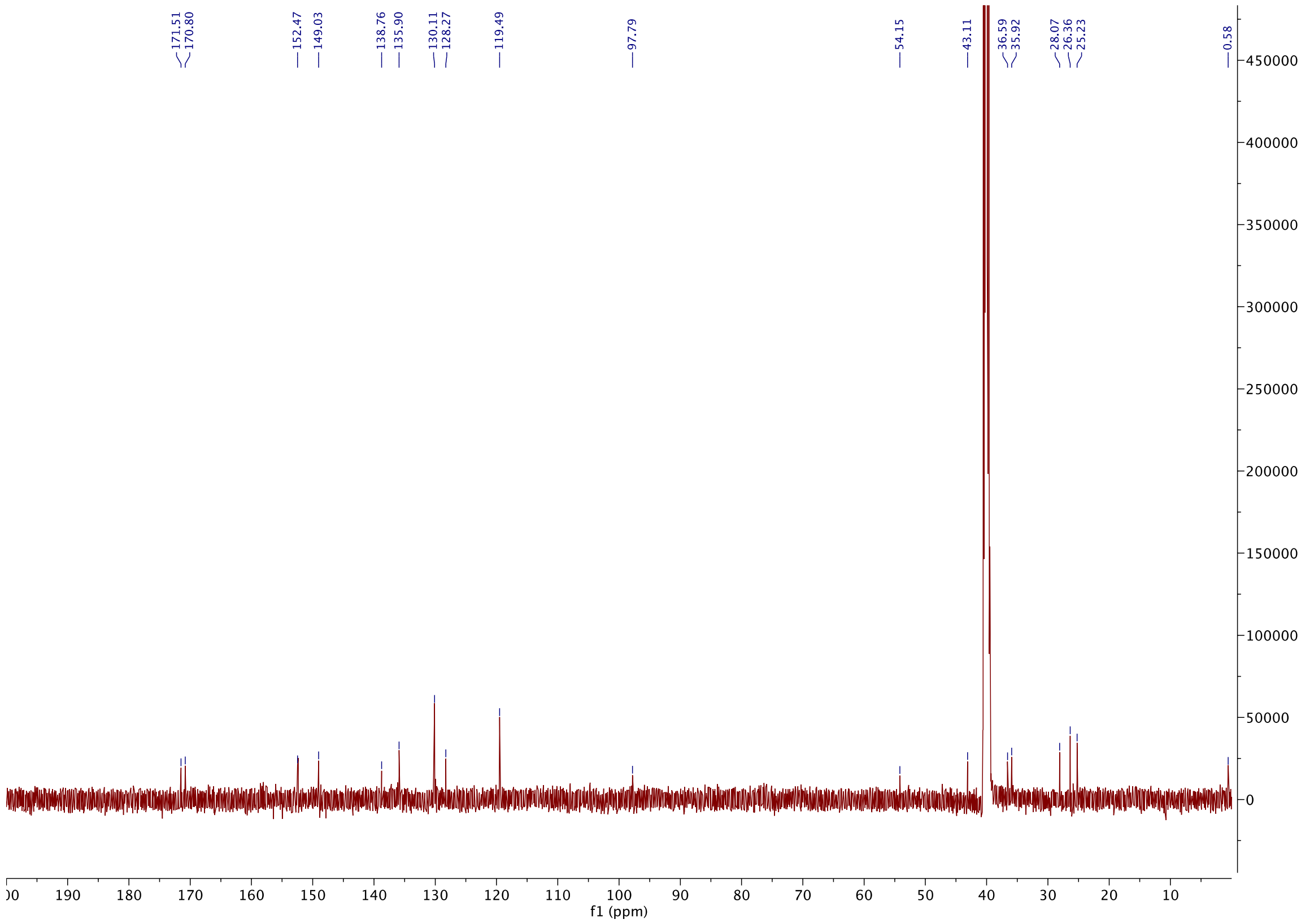

(25) ¹H NMR (500 MHz, DMSO-d₆)

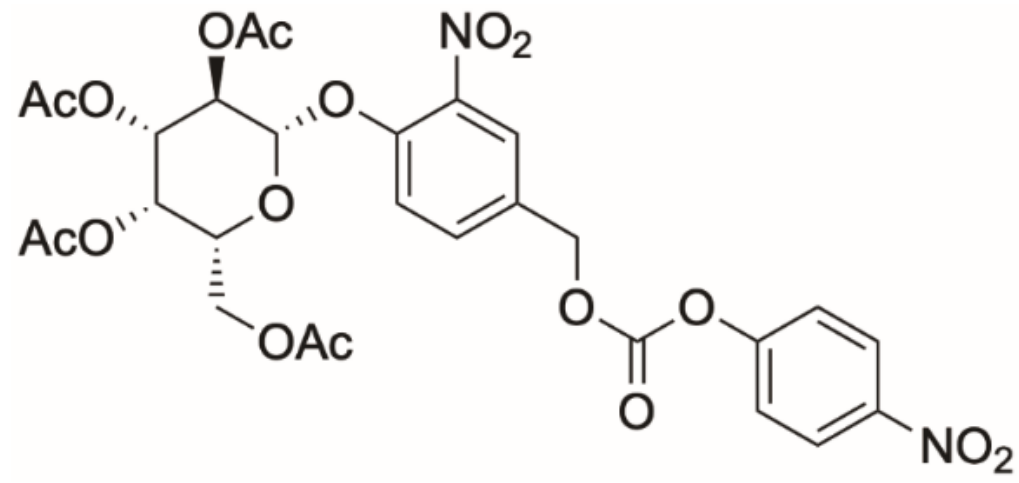

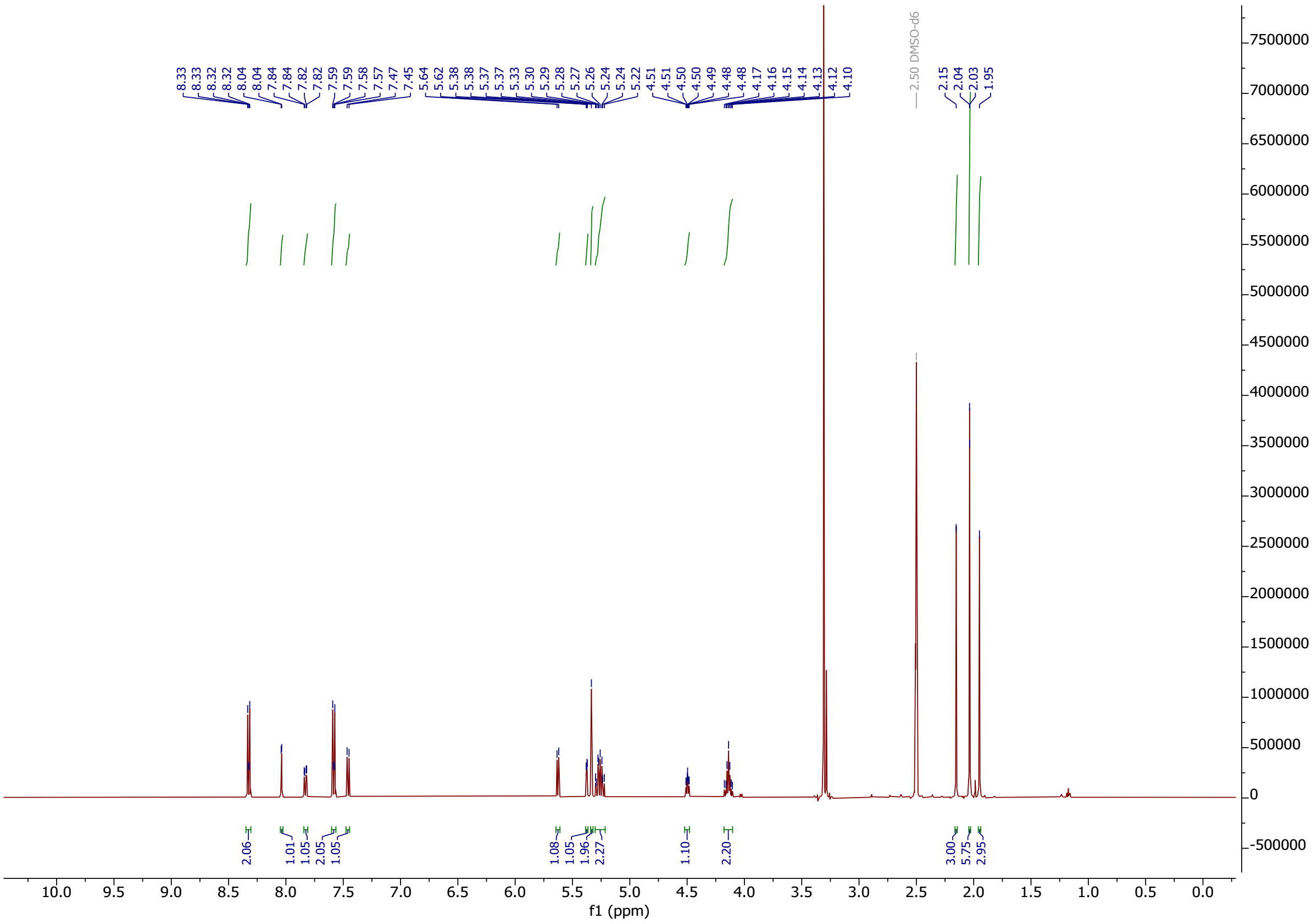

(25) ^{13}C NMR (126 MHz, DMSO-d$_6$)

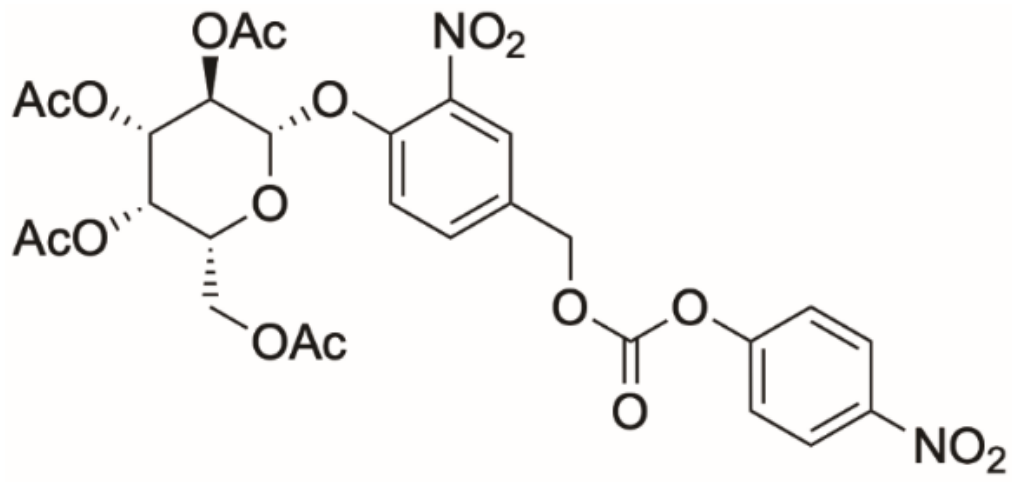

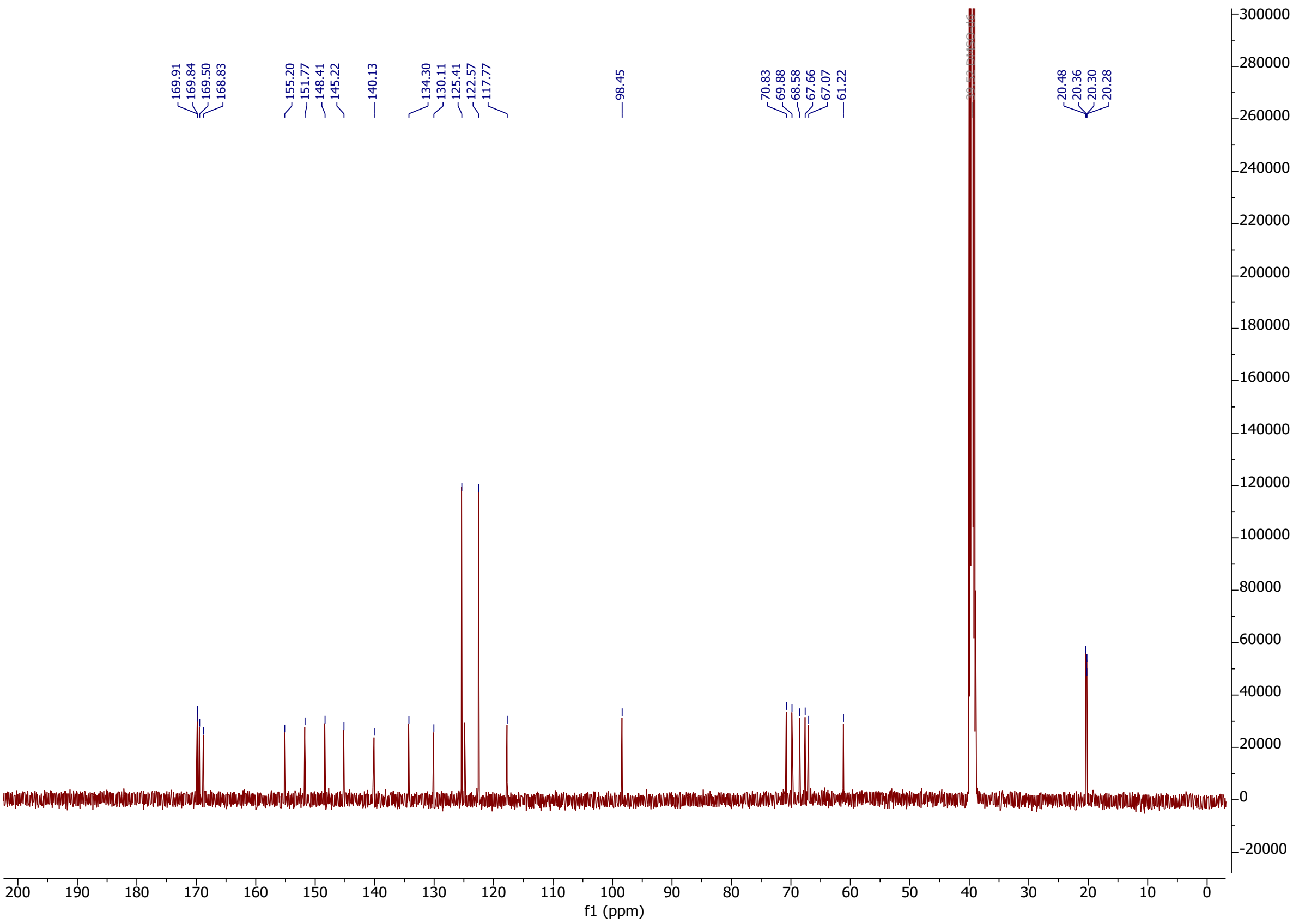

(26) ^{1}H NMR (500 MHz, DMSO-d$_6$)

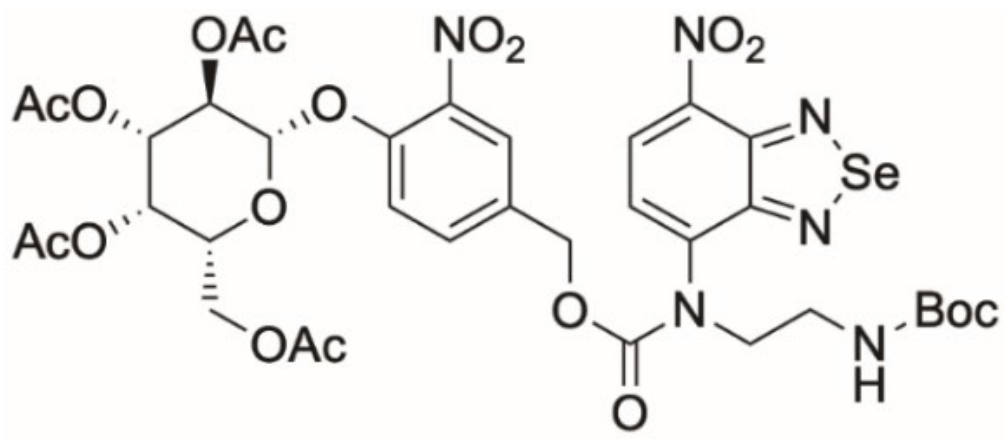

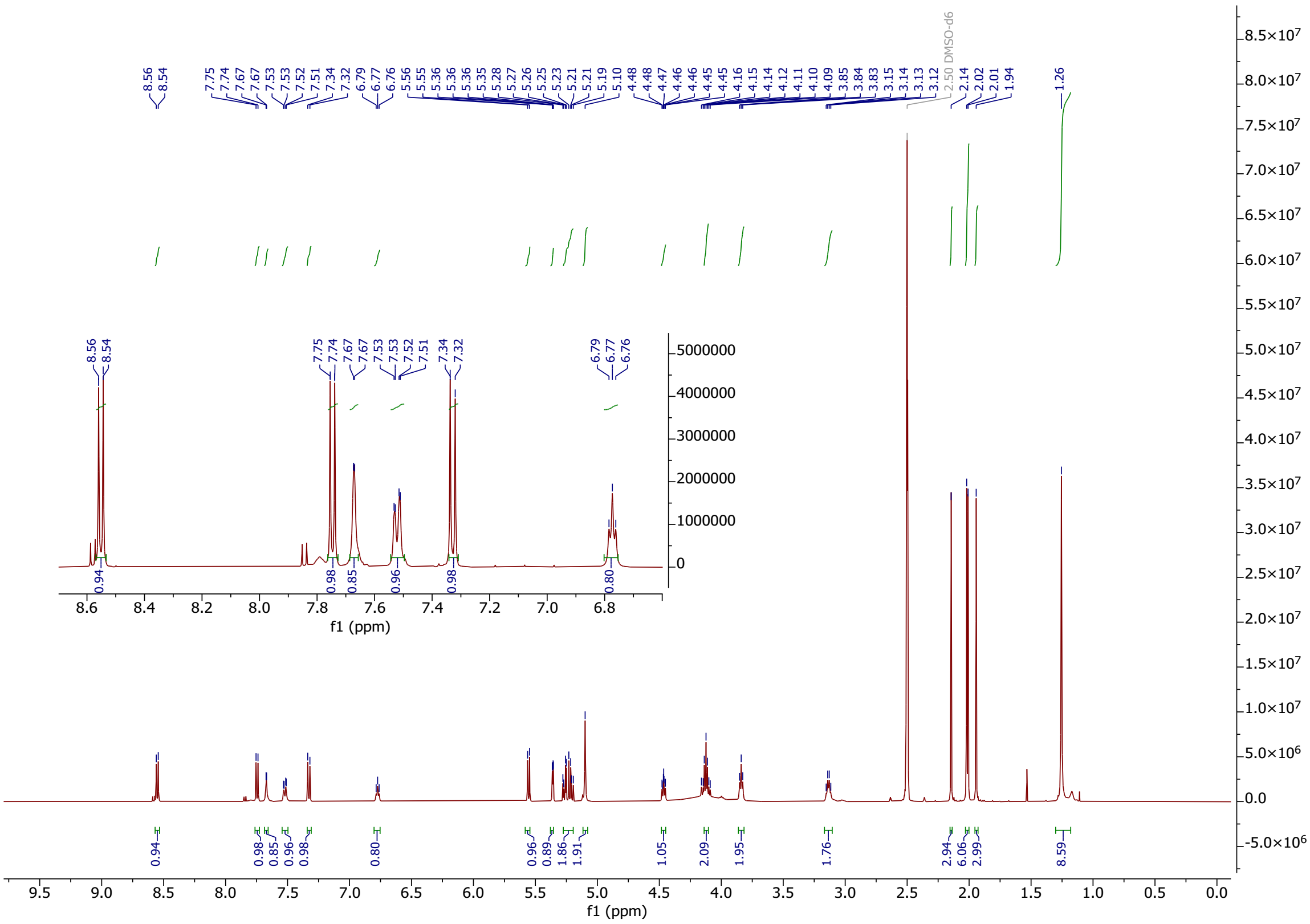

(26) ^{13}C NMR (126 MHz, DMSO-d$_6$)

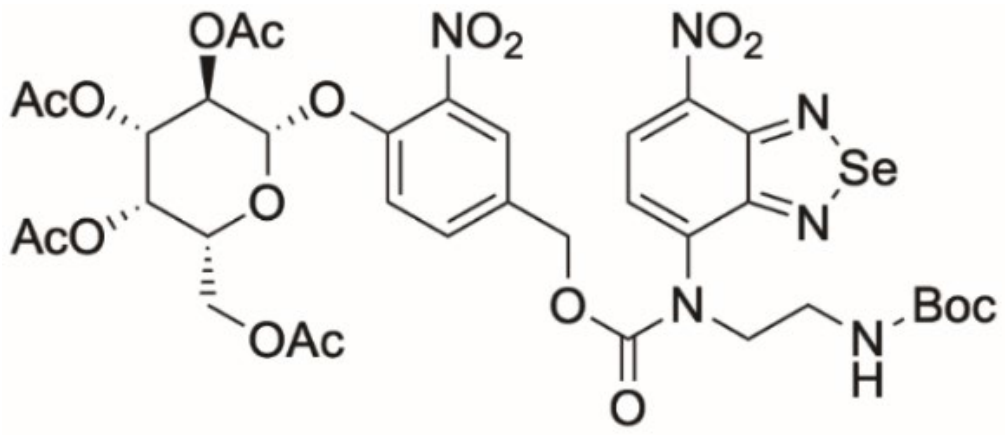

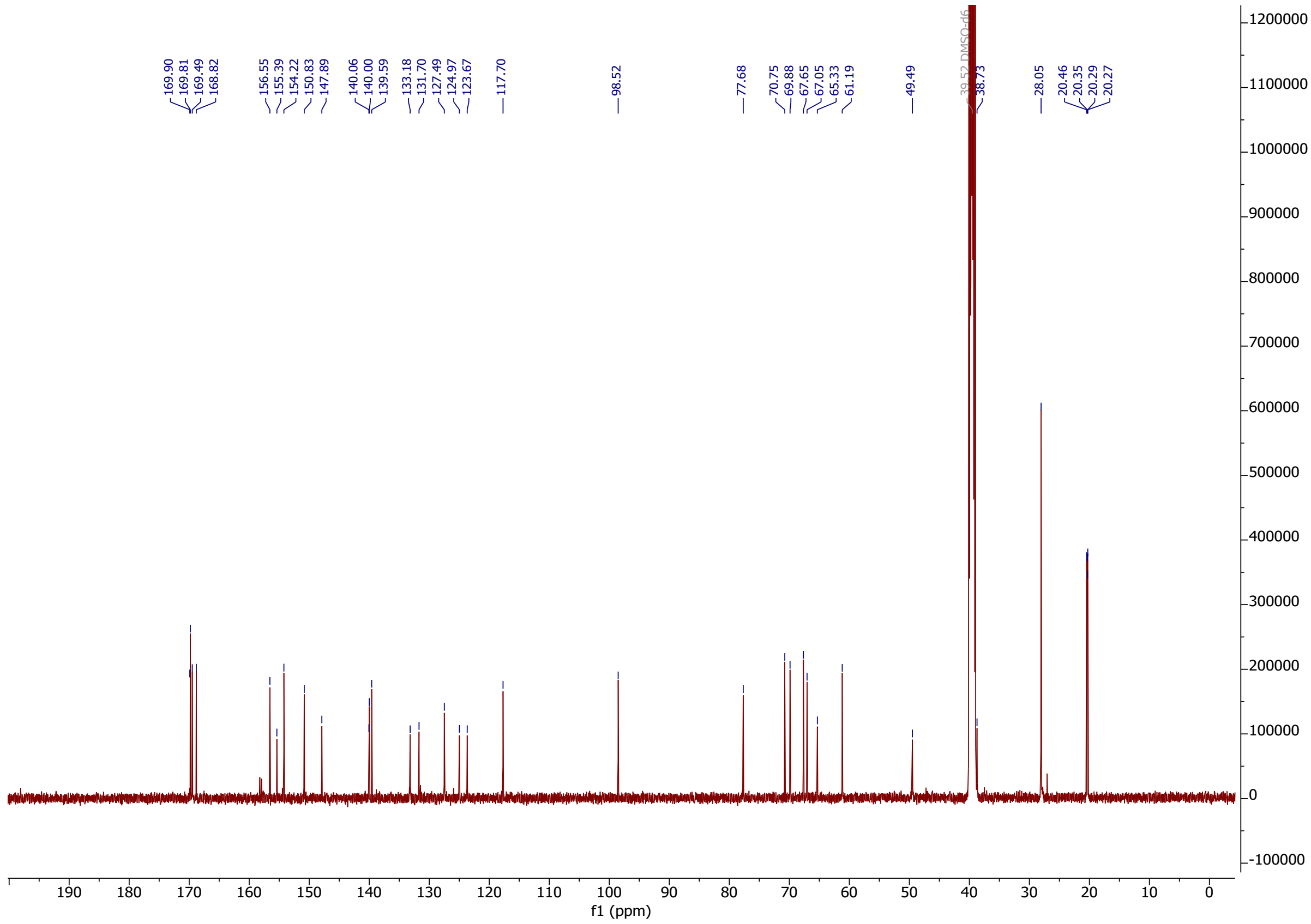

(27) ^{1}H NMR (500 MHz, DMSO-d_6)

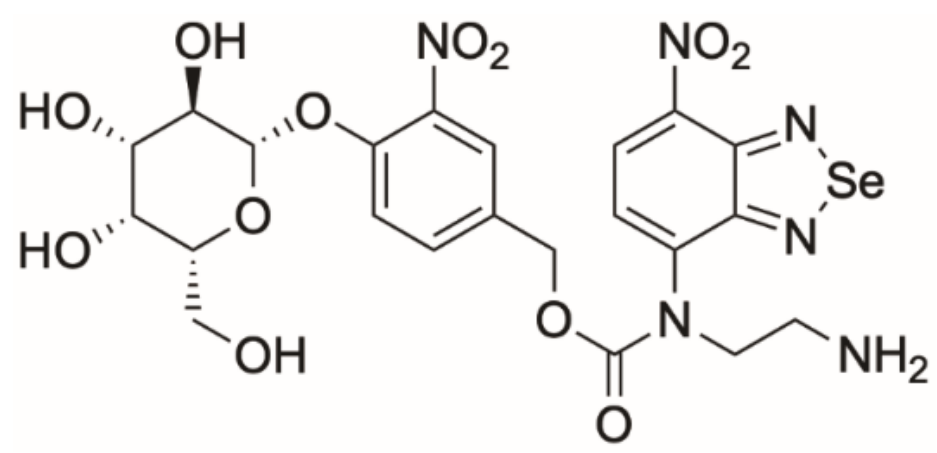

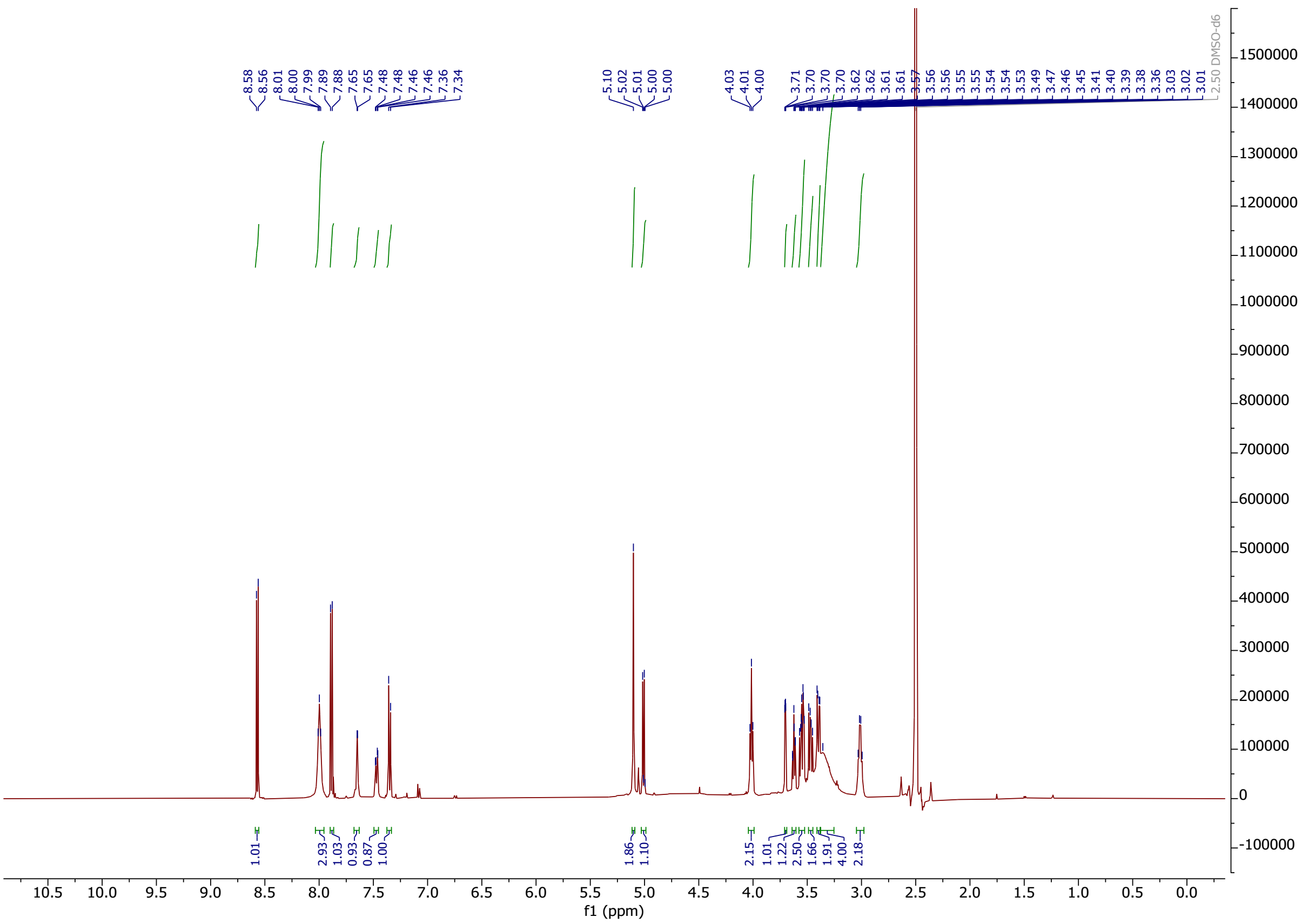

(27) ^{13}C NMR (126 MHz, DMSO-d_6)

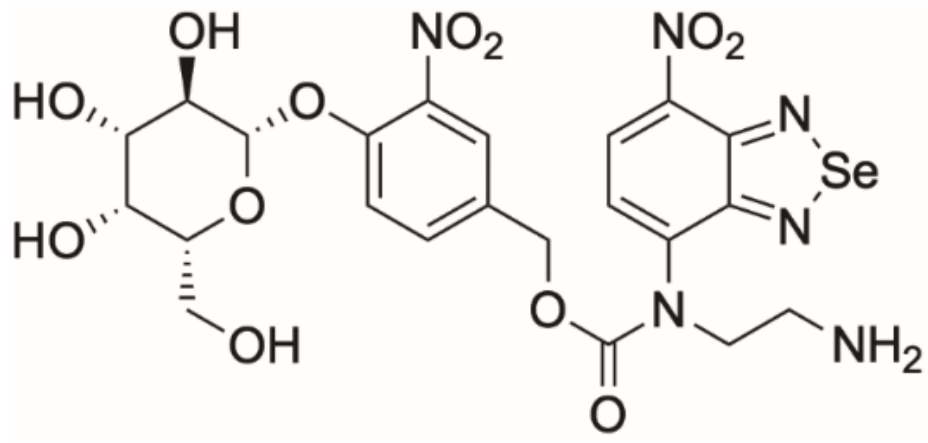

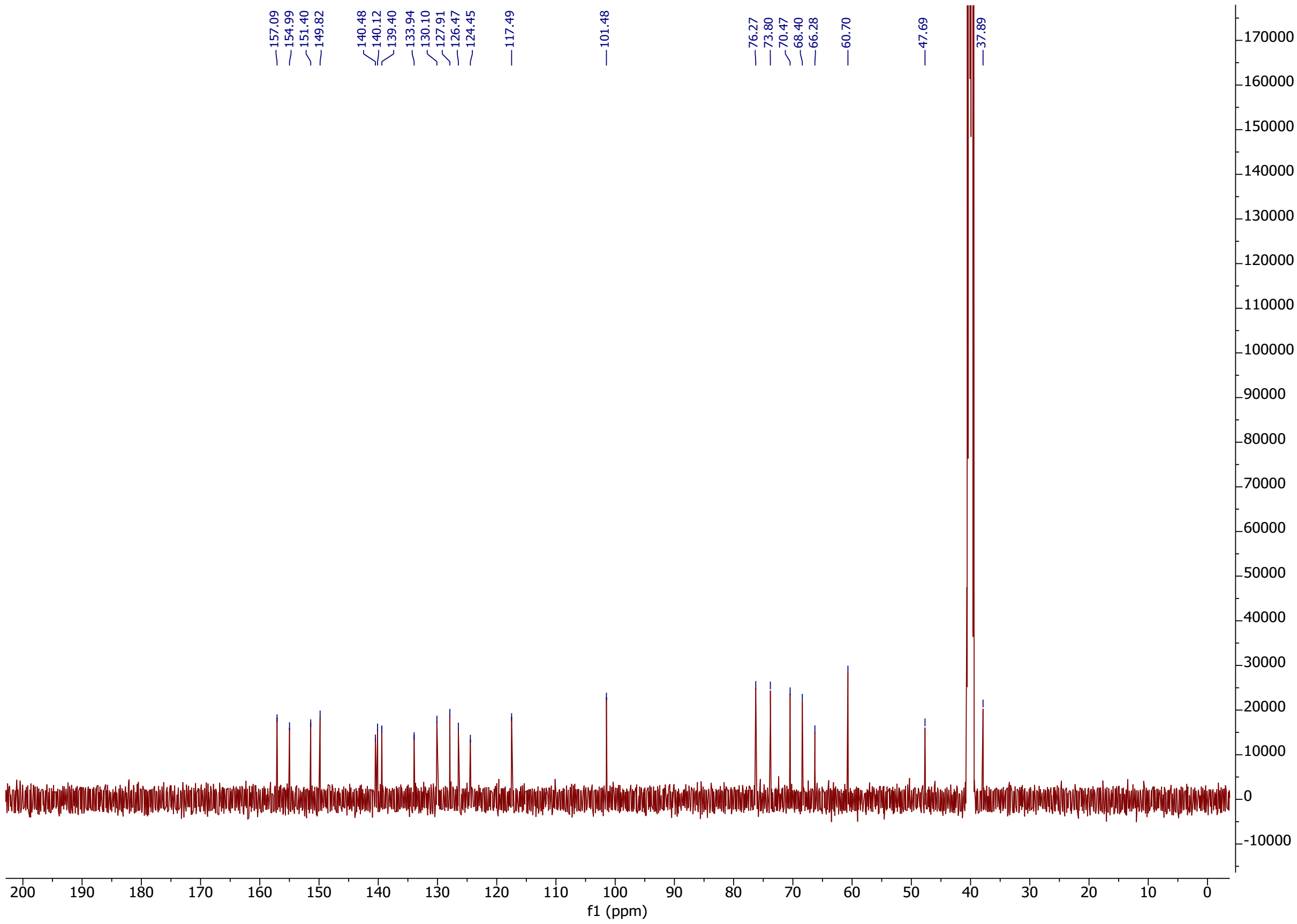

(Gal-SeNBD-oleamide) ^{1}H NMR (500 MHz, DMSO-d_6)

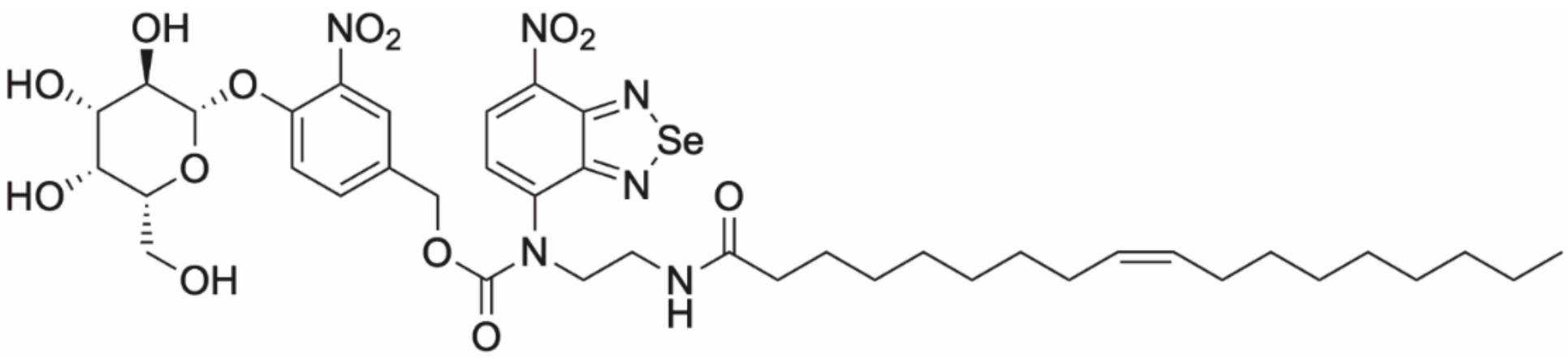

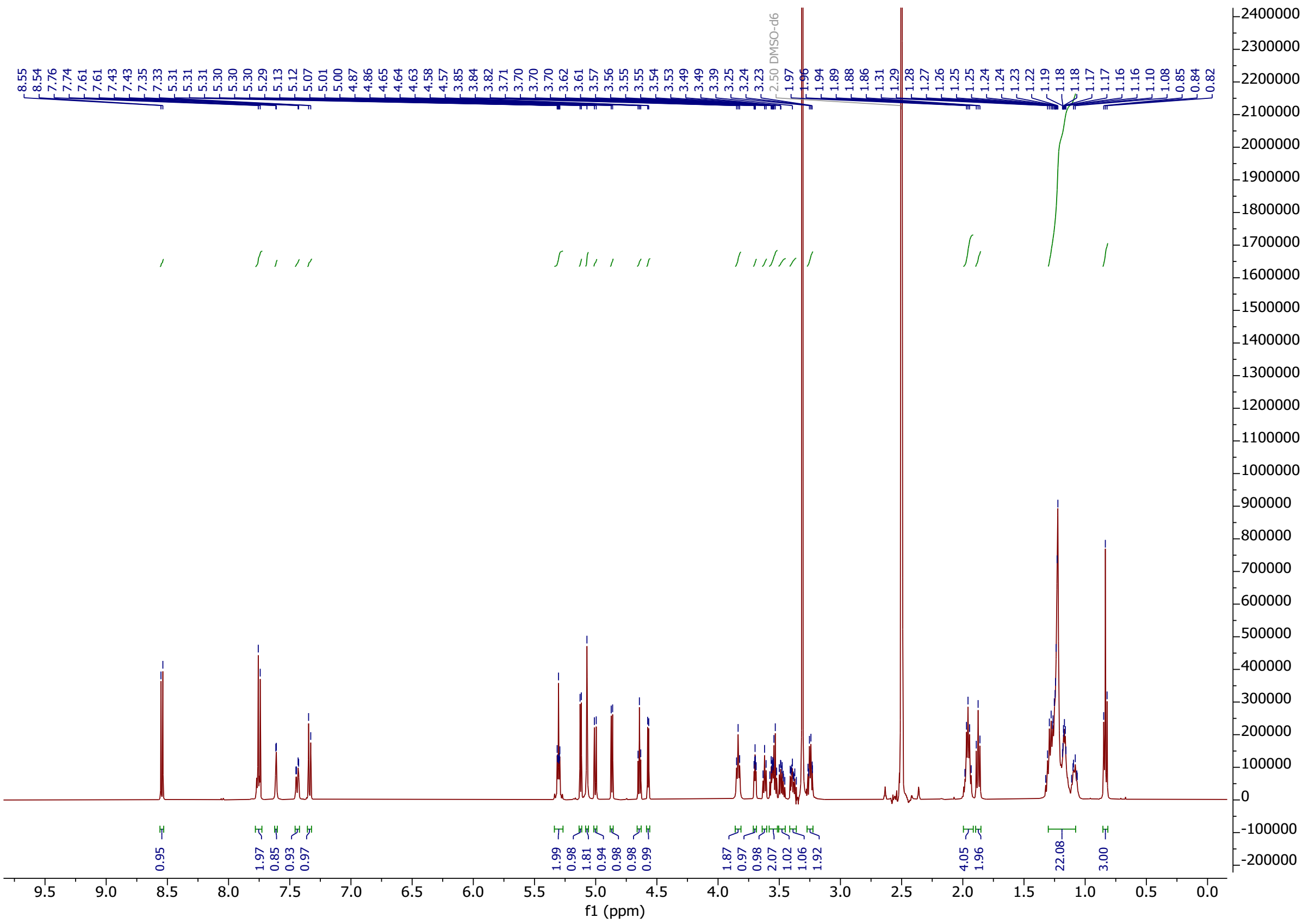

(Gal-SeNBD-oleamide) ^{13}C NMR (126 MHz, DMSO-d_6)

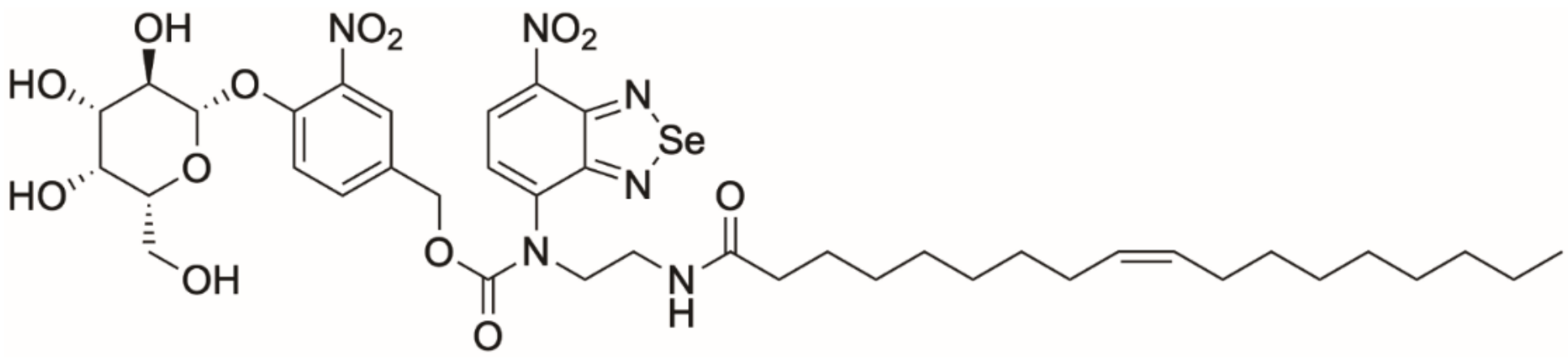

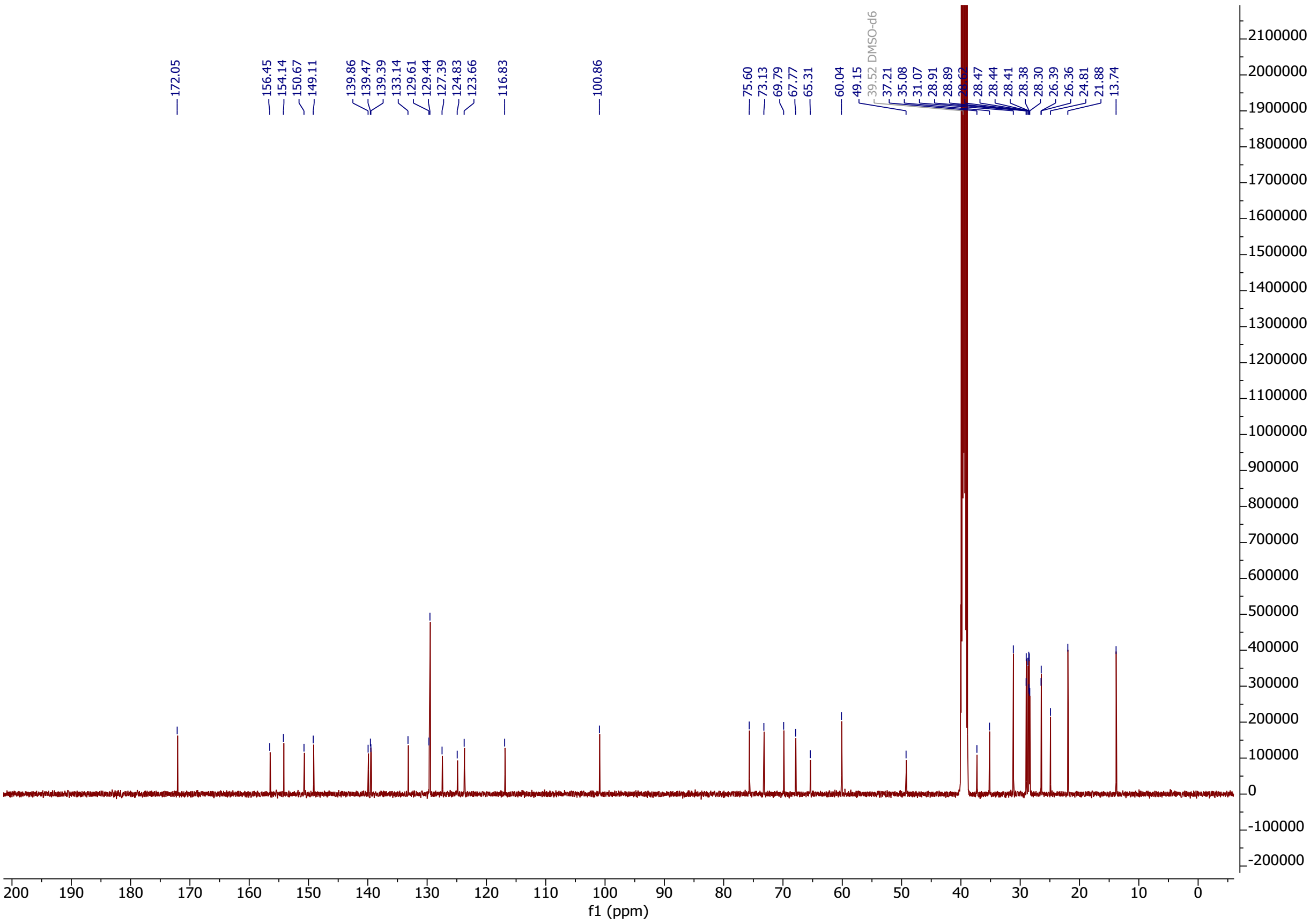

(28.1) ^1H NMR (500 MHz, DMSO-d_6)

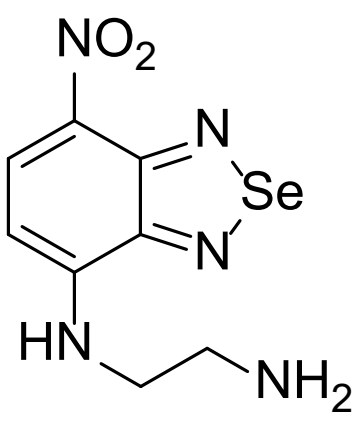

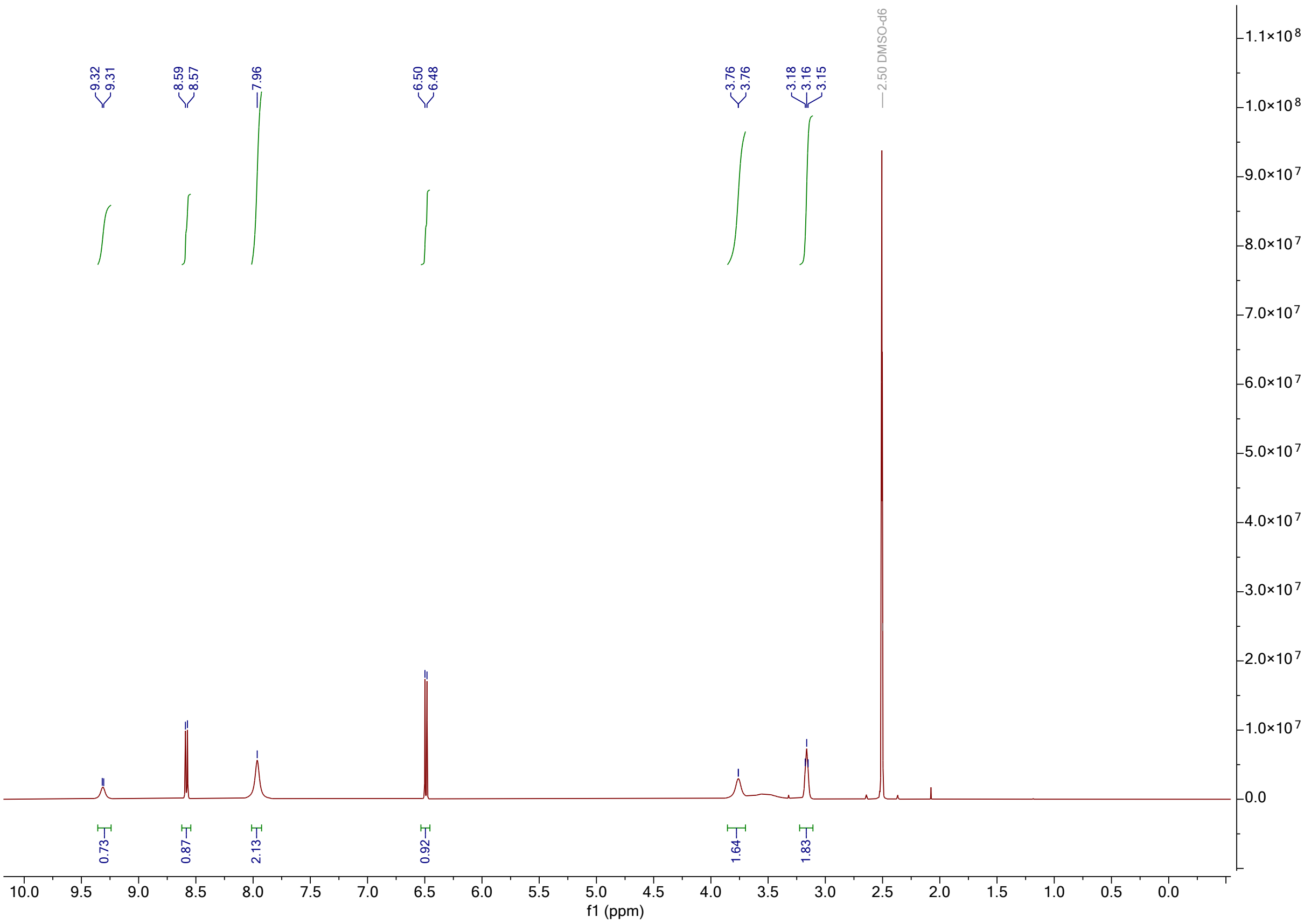

(28.1) ^{13}C NMR (126 MHz, DMSO-d_6)

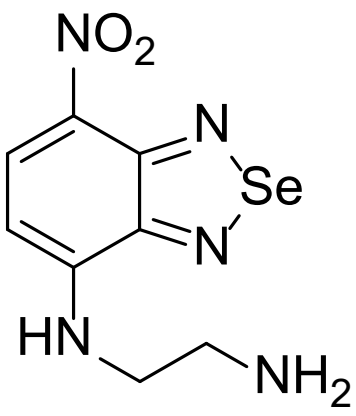

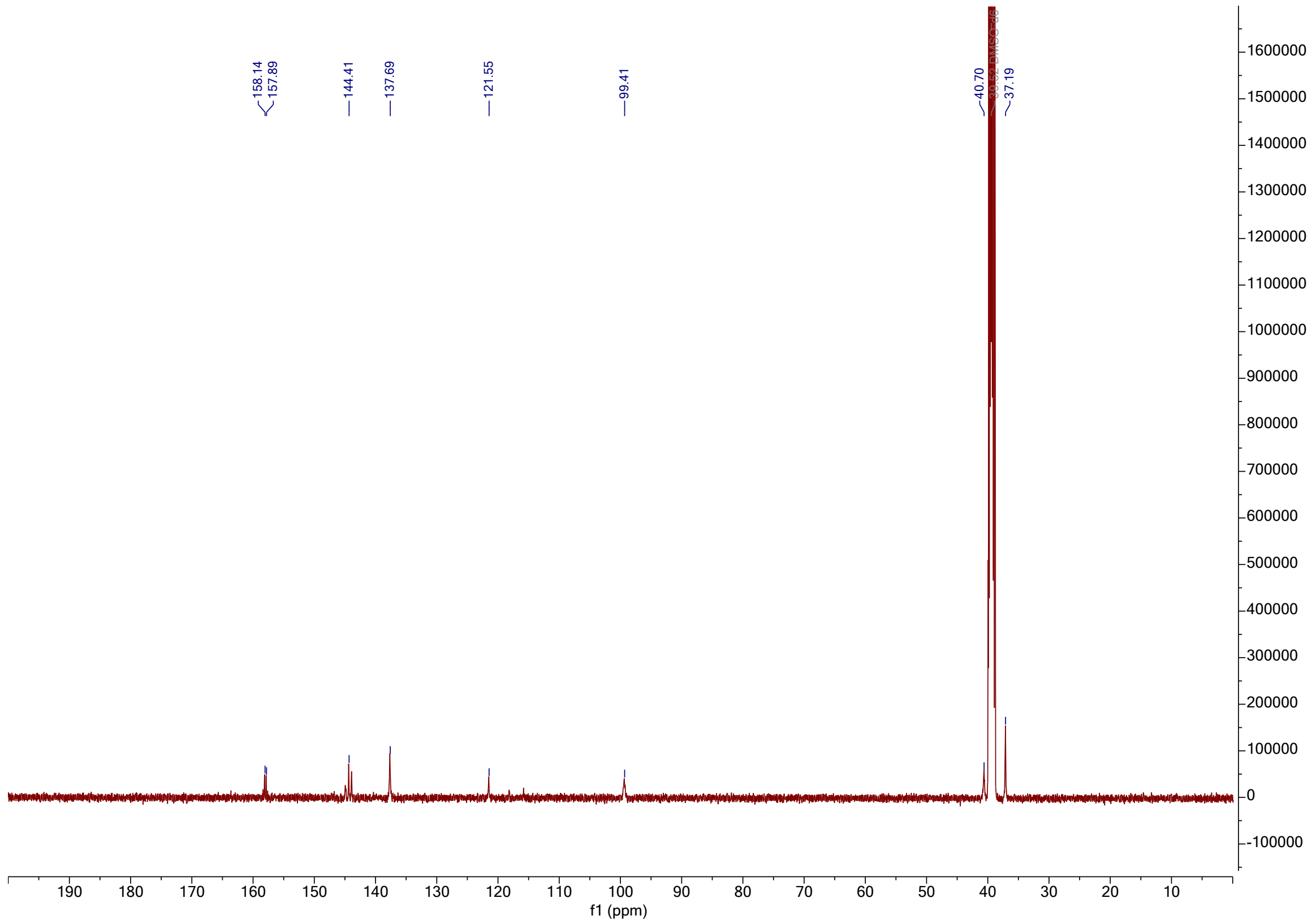

(28) ^{1}H NMR (500 MHz, DMSO-d$_6$)

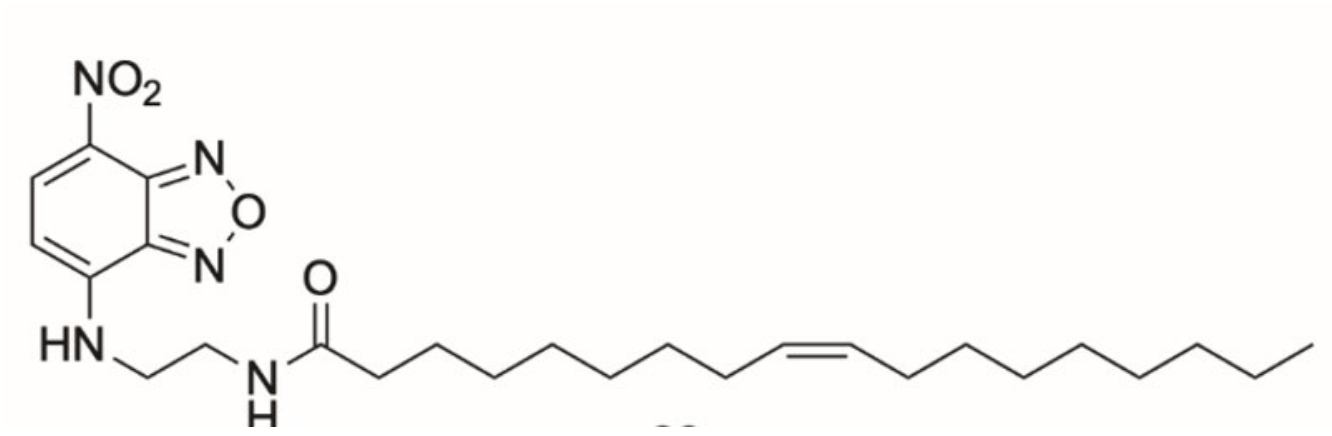

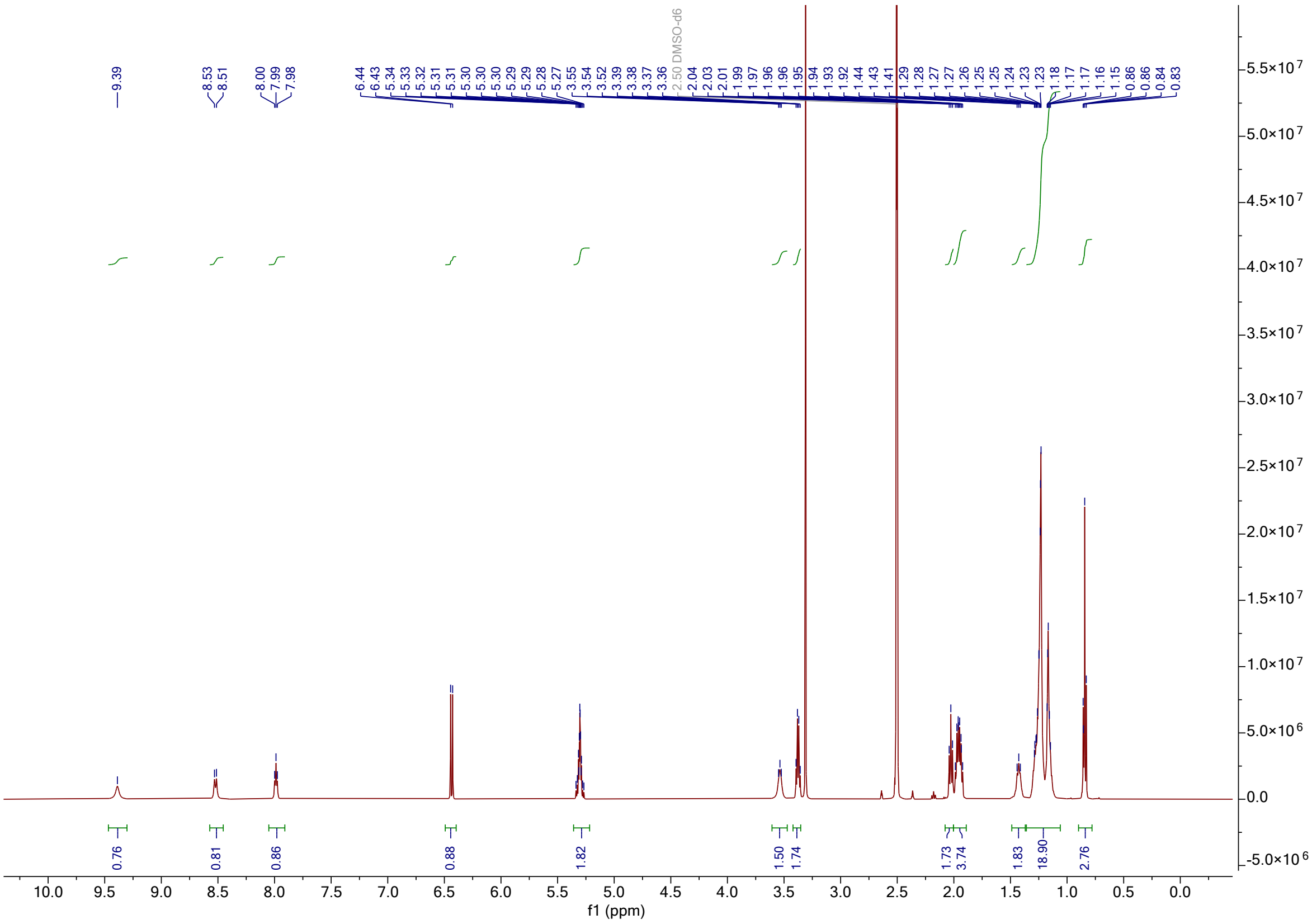

(28) ^{13}C NMR (126 MHz, DMSO-d_6)

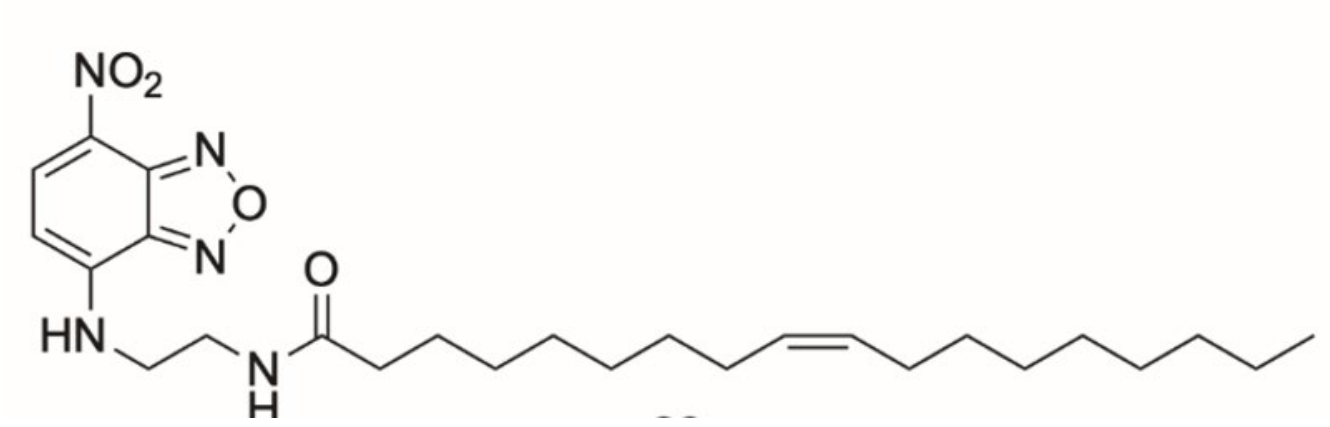

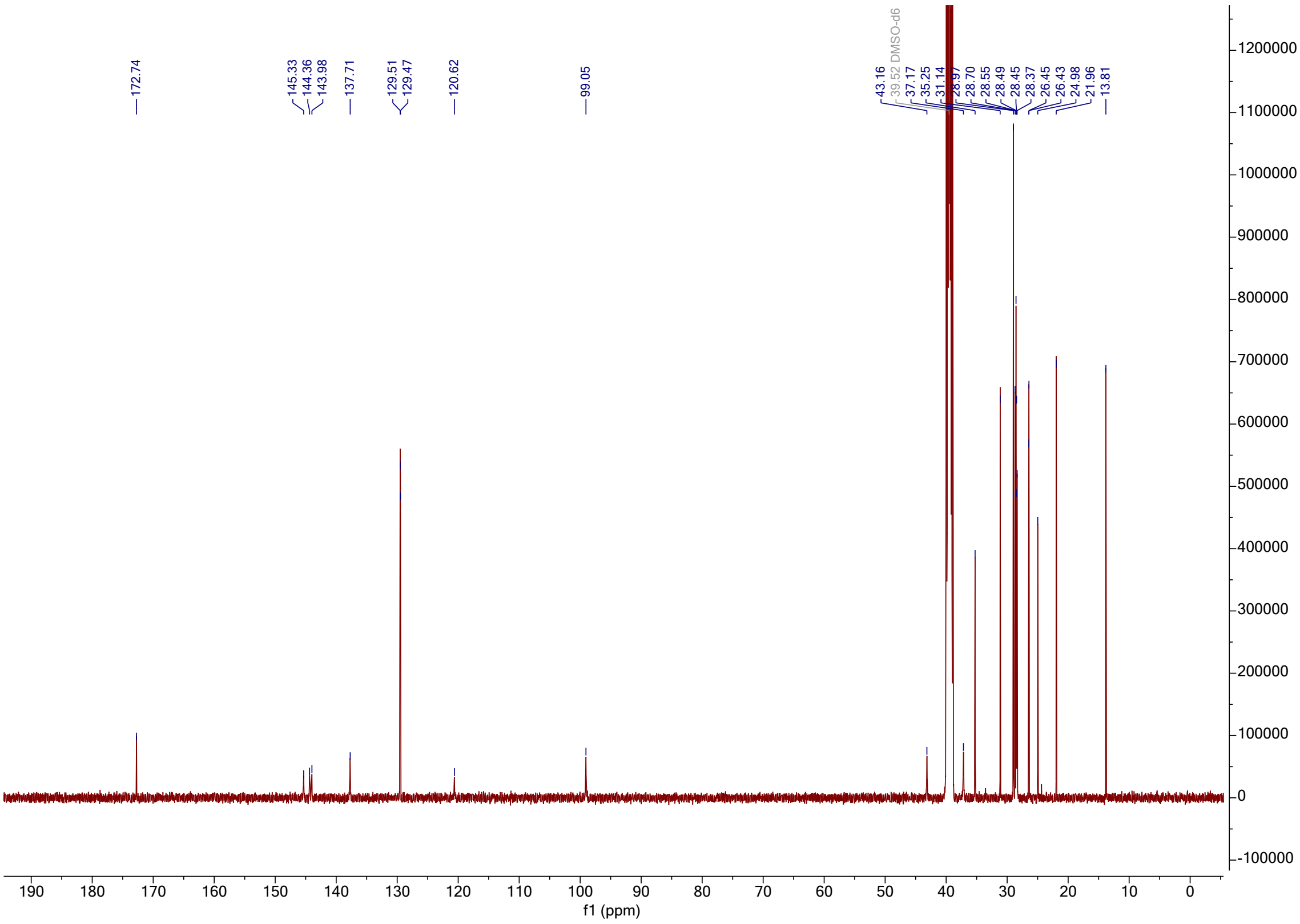

(29) ¹H NMR (500 MHz, DMSO-d_6)

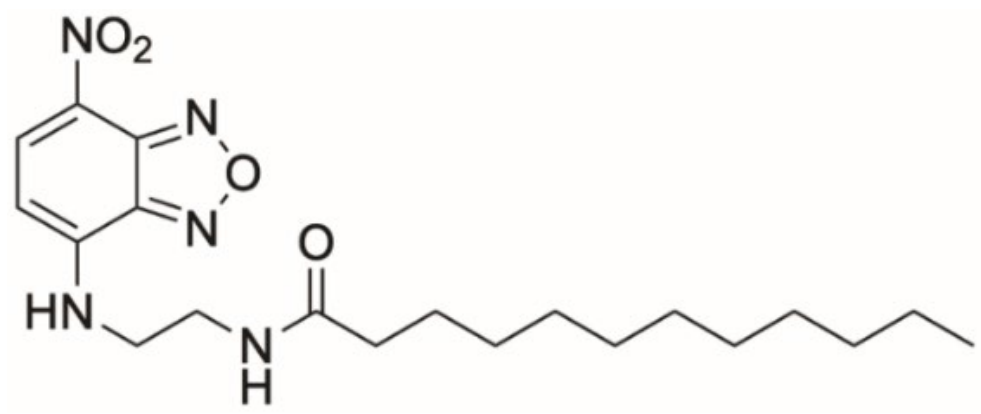

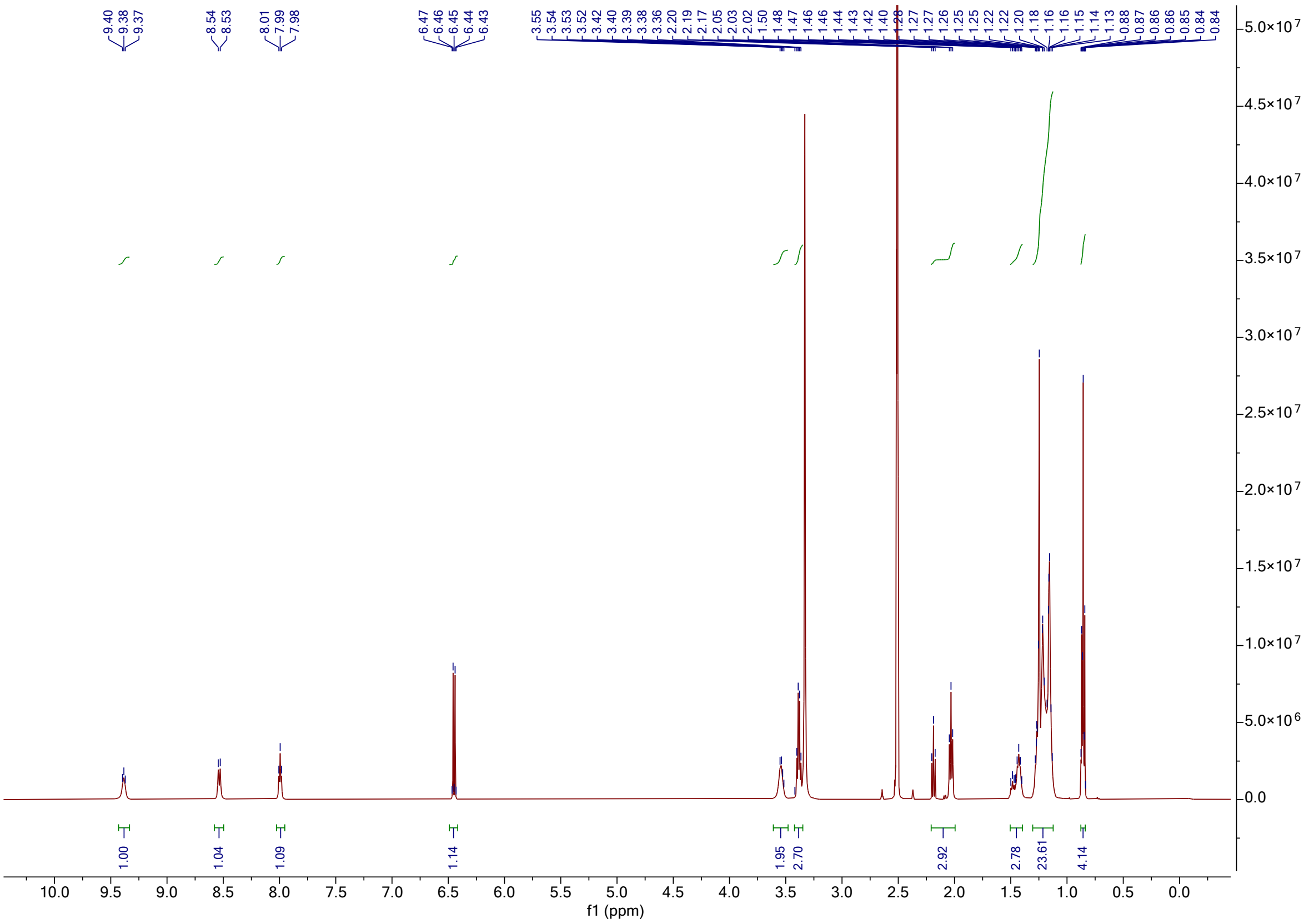

(29) ^{13}C NMR (126 MHz, DMSO-d_6)

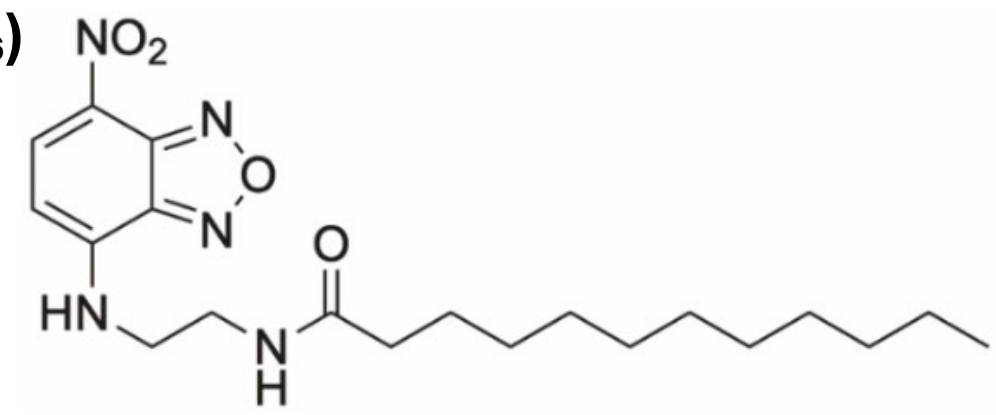

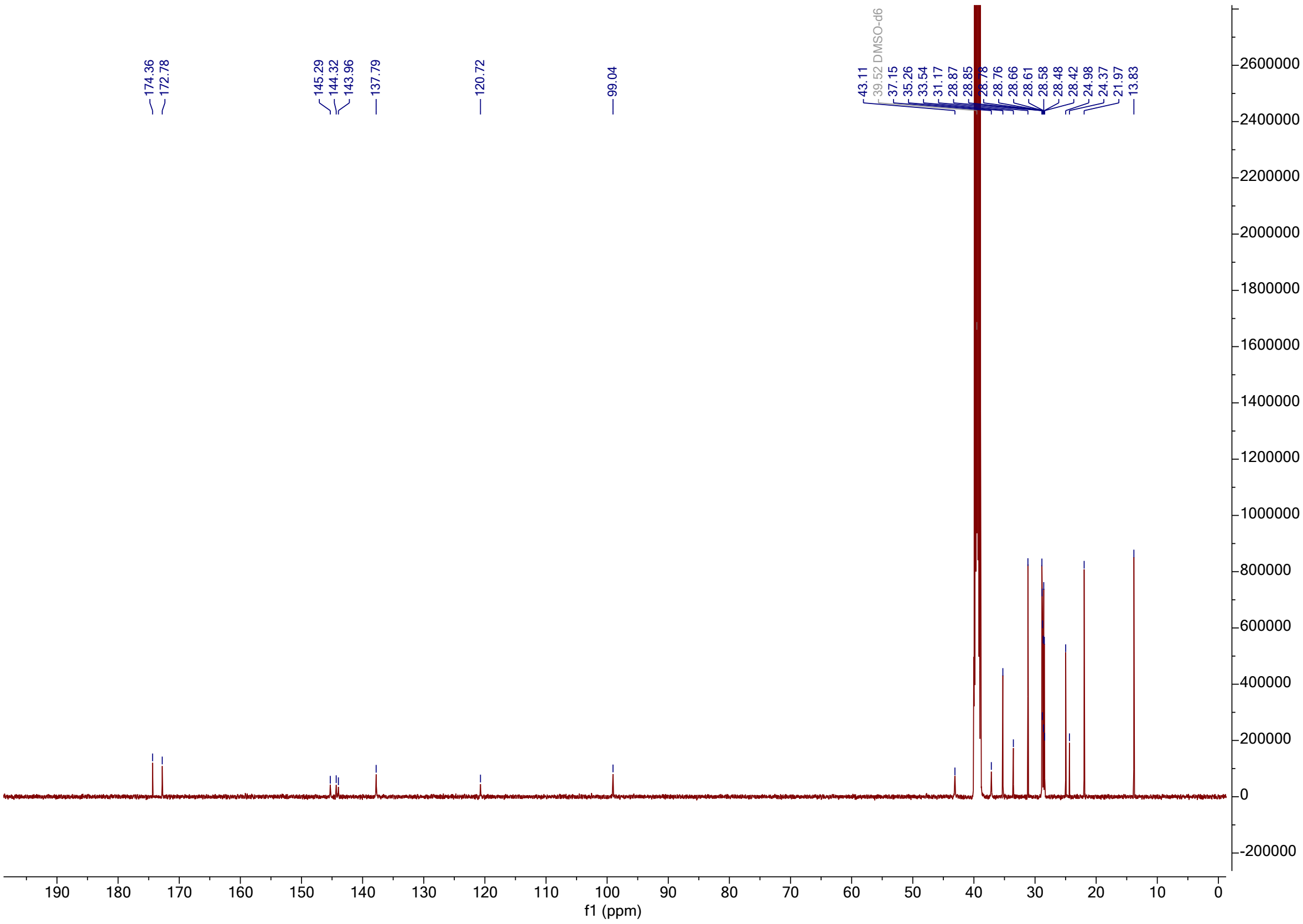

(30) ^{1}H NMR (500 MHz, DMSO-d_6)

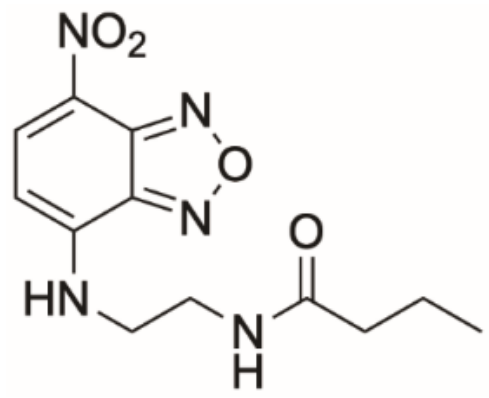

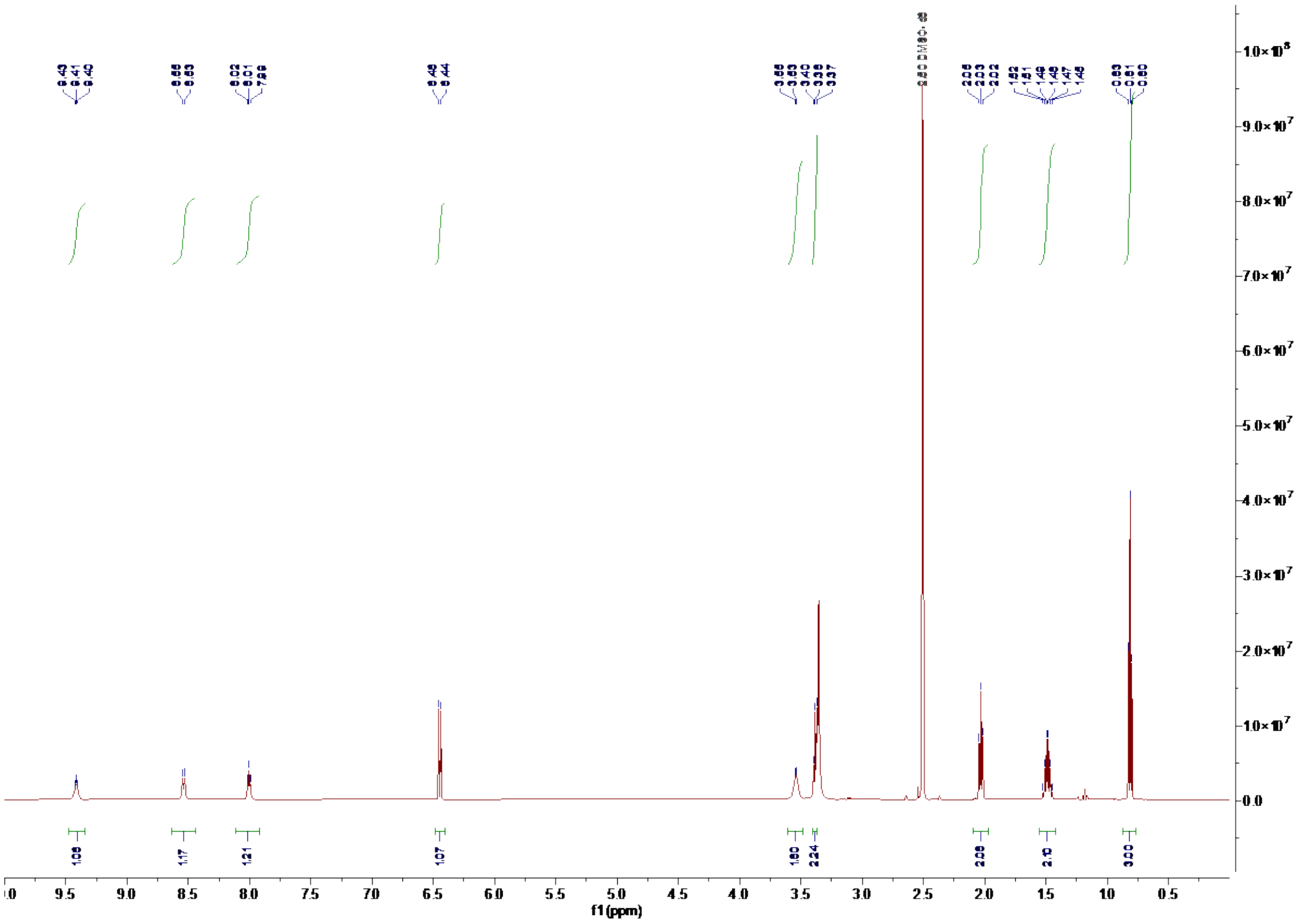

(30) ^{13}C NMR (126 MHz, DMSO-d_6)

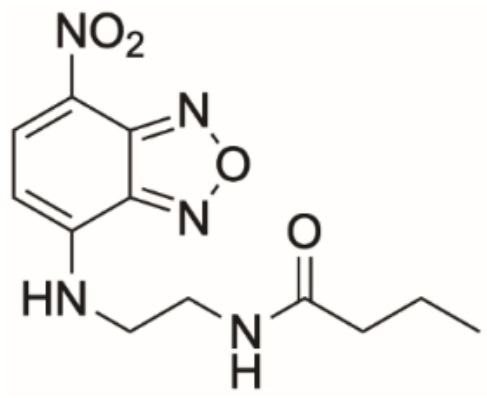

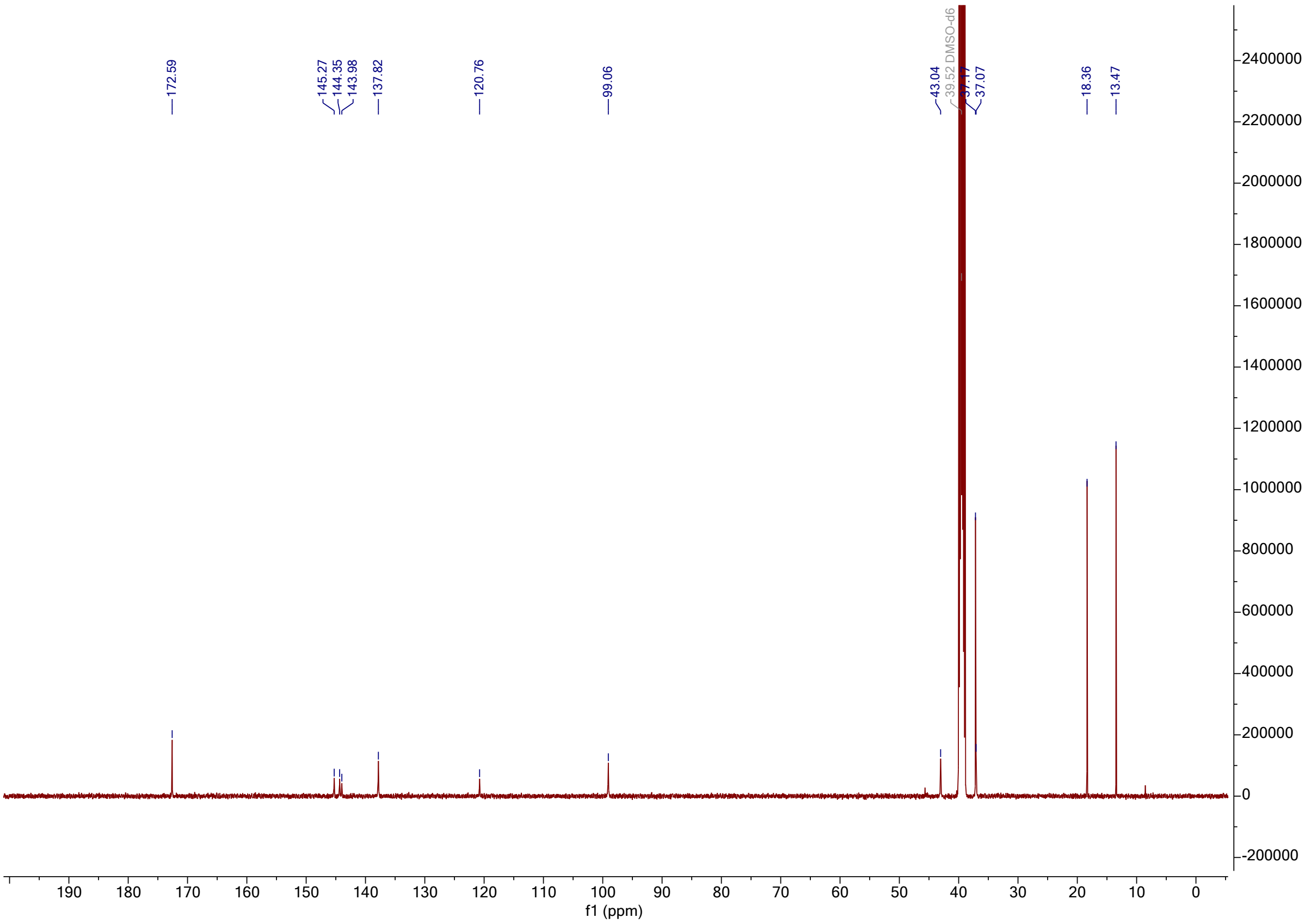

6. Supplementary References

(1) Rossi, A. G. et al. Cyclin-dependent kinase inhibitors enhance the resolution of inflammation by promoting inflammatory cell apoptosis. *Nat. Med.* **2006***, 12*, 1056–1064.

(2) Benson, S. et al. SCOTfluors: Small, Conjugatable, orthogonal, and tunable fluorophores for in vivo imaging of cell metabolism. *Angew. Chem. Int. Ed.* **2019***, 58*, 6911–6915.

(3) Benson, S. et al. Photoactivatable metabolic warheads enable precise and safe ablation of target cells in vivo. *Nat. Commun.* **2021***, 12*, 2369.

(4) Guminski, Y. et al. Synthesis of conjugated spermine derivatives with 7-nitrobenzoxadiazole (NBD), rhodamine and bodipy as new fluorescent probes for the polyamine transport system. *Bioorg. Med. Chem. Lett.* **2009***, 19*, 2474–2477.
